# Danggui Buxue Decoction Ameliorates Idiopathic Pulmonary Fibrosis through MicroRNA and Messenger RNA Regulatory Network

**DOI:** 10.1155/2022/3439656

**Published:** 2022-04-26

**Authors:** Huizhe Zhang, Xue Wang, Yanchen Shi, Mengying Liu, Qingqing Xia, Weilong Jiang, Yufeng Zhang

**Affiliations:** ^1^Nanjing University of Chinese Medicine, Nanjing, Jiangsu 210023, China; ^2^Department of Respiratory Medicine, Yancheng Hospital of Traditional Chinese Medicine, Yancheng Hospital Affiliated to Nanjing University of Chinese Medicine, Yancheng, Jiangsu 224005, China; ^3^Department of Pulmonary and Critical Care Medicine, Nanjing Drum Tower Hospital Clinical College of Nanjing Medical University, Nanjing, Jiangsu 210008, China; ^4^Department of Pulmonary and Critical Care Medicine, Jiangyin Hospital of Traditional Chinese Medicine, Jiangyin Hospital Affiliated to Nanjing University of Chinese Medicine, Jiangyin, Jiangsu 214400, China

## Abstract

Objective. To develop a putative microRNA (miRNA) and messenger RNA (mRNA) regulatory network of Danggui Buxue decoction's (DGBXD) amelioration of idiopathic pulmonary fibrosis (IPF). Methods. The Gene Expression Omnibus (GEO) database was used to identify differentially expressed miRNAs (DE-miRNAs) and differentially expressed mRNAs (DE-mRNAs). Using miRNet, the predicted target genes of identified DE-miRNAs were estimated, and then the target genes of DE-miRNAs in IPF were comprehensively examined. The Enrichr database was used to conduct functional enrichment and pathway enrichment. Traditional Chinese Medicine Systems Pharmacology Database and Analysis Platform (TCMSP) was employed to obtain the target genes of DGBXD as well as active compounds. A putative miRNA-mRNA regulatory network of DGBXD acting on IPF was developed by intersecting the target genes of DGBXD with the DE-miRNA target genes in IPF. A bleomycin-induced mouse model was established and used to perform histopathology as well as real-time quantitative polymerase chain reaction (qRT-PCR) analyses of some miRNA-mRNA pairs. Results. Fourteen upmodulated DE-miRNAs and six downmodulated DE-miRNAs were screened. The downstream target genes of upmodulated and downmodulated DE-miRNAs were predicted. Subsequently, 1160 upmodulated DE-mRNAs and 1427 downmodulated DE-mRNAs were identified. Then, target genes of DE-miRNAs comprising 49 downmodulated and 53 upmodulated target genes were further screened to perform functional enrichment and pathway enrichment analyses. Subsequently, 196 target genes of DGBXD were obtained from TCMSP, with six downregulated target genes and six upregulated target genes of DGBXD acting on IPF being identified. A promising miRNA-mRNA regulatory network of DGBXD acting on IPF was developed in this study. Moreover, mir-493 together with its target gene Olr1 and mir-338 together with Hif1a were further validated by qRT-PCR. Conclusion. This study proposed detailed possible processes of miRNA-mRNA modulatory axis in IPF and constructed a prospective IPF-related miRNA-mRNA modulatory network with the aim of alleviating IPF with DGBXD.

## 1. Introduction

Idiopathic pulmonary fibrosis (IPF) is a chronic and progressive lung condition whose characteristics include pulmonary fibrosis. The etiology of IPF remains unknown, with its pathological manifestation being usual interstitial pneumonia (UIP) [1]. IPF is more common in the elderly, but it is a rare disease with an estimated incidence of approximately 2.8–9.3 per 100,000 people in Europe and North America [2–4]. However, in China, epidemiological data about this condition are scarce, although its incidence has significantly increased in recent years [5]. IPF cannot be cured, so the main goals of the current treatment are to delay disease progression, improve quality of life, and prolong patient survival [6]. Limited drug options are available for IPF, with pirfenidone and nintedanib having obvious curative effects, while traditional Chinese medicine (TCM) can play an integral role in managing IPF [7–10]. Nonetheless, the prognosis of patients with IPF remains dismal, with median survival duration of approximately 2–3 years from the initial diagnosis [11]. There is thus a crucial need to develop a successful treatment for IPF.

MicroRNAs (miRNAs) are endogenous, double-stranded small RNA molecules ranging in length between 20 and 24 nucleotides that do not encode proteins [12]. miRNAs play an integral role in silencing RNA and posttranscriptionally modulating the expression of genes by interacting with messenger RNA (mRNA) via the base pairs of intramolecular complementary sequences [13]. Studies have reported that a single miRNA can regulate numerous genes, while many miRNAs can also coregulate a single gene. Therefore, miRNAs are involved in myriad cellular activities, such as cell differentiation, apoptosis, proliferation, migration, and energy metabolism [14]. Furthermore, miRNAs play significant roles in organ fibrosis, particularly in IPF, potentially being correlated with disease pathogenesis [15–19].

Studies have demonstrated that TCM can modulate miRNA-mRNA networks in the treatment of different diseases [20–22]. However, to the best of our knowledge, no evidence has been reported on the use of TCM for treating IPF through regulating the miRNA-mRNA network. Recently, in a study conducted by the authors of this paper, a putative IPF-related miRNA-mRNA modulatory network was developed, which could aid in the discovery of new miRNA-mRNA axes of IPF [23]. This can also promote further studies on TCM acting on IPF-related miRNA-mRNA regulatory networks.

Danggui Buxue decoction (DGBXD), which is an ancient classical formula among hundreds of thousands of TCM formulae, has been clinically used in China for over 800 years. The two herbs contained in DGBXD are Radix Astragali (RA; Huangqi in Chinese) and Radix Angelicae Sinensis (RAS; Danggui in Chinese) [24]. Recently, a series of studies on the use of DGBXD for treating IPF were conducted. The obtained results confirmed the effectiveness and safety of RA and RAS in IPF patients [25] and clarified the multipathway, multitarget, and multicomponent mechanisms by which RA and RAS act in the treatment of IPF [26, 27]. Therefore, DGBXD can effectively treat IPF by targeting multiple genes. Building on the previous reports on DGBXD- and miRNA-related studies [28, 29] and the miRNA-mRNA regulatory network that we developed earlier, this study further investigates whether DGBXD alleviates IPF by regulating the miRNA-mRNA modulatory network, which can facilitate further research of the relevant miRNA-mRNA pairs experimentally verified and the material basis by which DGBXD acts on IPF through the miRNA-mRNA modulatory network.

In this research, differentially expressed miRNAs (DE-miRNAs) between tissues of patients with IPF and tissues of healthy controls were screened for the first time using miRNA datasets and downstream target genes of the DE-miRNAs and were predicted using a network database. In addition, differentially expressed mRNAs (DE-mRNAs) between IPF and normal tissues were acquired with the aid of an IPF mRNA dataset. Moreover, target genes of DE-miRNAs in IPF were identified to conduct functional enrichment based on Gene Ontology (GO) and pathway enrichment based on the Kyoto Encyclopedia of Genes and Genomes (KEGG). Subsequently, target genes of DGBXD, as well as active compounds, were discovered. After the active target genes of DGBXD intersected with the DE-miRNA target genes in IPF, the target genes of DGBXD acting on IPF were also determined. Ultimately, a putative miRNA-mRNA regulatory network associated with the action of DGBXD on IPF was identified. The experimental verification of relevant miRNA-mRNA pairs was performed, which should facilitate further research.

## 2. Materials and Methods

### 2.1. Searching and Screening of Microarray Datasets

A search for datasets focusing on the miRNAs and mRNAs related to IPF was performed in the National Center for Biotechnology Information (NCBI) Gene Expression Omnibus (GEO) database (https://www.ncbi.nlm.nih.gov/gds/). Taking miRNA expression as an example, the retrieval strategy used in this study was as follows: {[“microRNAs” (MeSH Terms) OR “microRNA” (All Fields)] AND “idiopathic pulmonary fibrosis” (All Fields)}. Datasets derived from IPF clinical patients and containing IPF-related and normal samples were screened for inclusion in this study.

### 2.2. Identification of DE-miRNAs

We employed the GEO2R online tool (https://www.ncbi.nlm.nih.gov/geo/geo2r/) to directly detect DE-miRNAs between IPF and normal samples in the screened miRNA datasets [30, 31]. Differentially expressed miRNAs were identified by GEO2R by comparing the IPF group with the normal group, setting |log2FoldChange (FC)| > 1 and adjusted (adj) *p* value < 0.05 as the cut-off values.

### 2.3. Predicting Downstream Target Genes of DE-miRNAs

The miRNet (https://www.mirnet.ca/) platform, which is an integrative platform integrating miRNAs, functions, and targets, was used for the purpose of estimating the downstream target genes of DE-miRNAs [32–34]. The DE-miRNAs that were downmodulated and upmodulated were entered into the web platform, while the data of target genes of downmodulated and upmodulated DE-miRNAs were downloaded.

### 2.4. Identification of DE-mRNA and DE-miRNA Target Genes in IPF

The screened IPF-related mRNA datasets were analyzed to enhance the validity of our additional analysis of the target genes of DE-miRNAs that had been screened. Series matrix files were downloaded from the GEO database. Setting |log2FC| > 1 and adj *p* value < 0.05 as the cut-off values, DE-mRNAs in IPF were discovered with the aid of the RGui and limma packages [35].

Then, an intersection analysis of DE-mRNAs and predicted target genes of DE-miRNAs was performed for the purpose of identifying the target genes of DE-miRNAs in IPF.

### 2.5. GO Functional Enrichment and KEGG Pathway Enrichment Analyses

The Enrichr database, a reliable online tool for Gene Set Enrichment Analysis (https://amp.pharm.mssm.edu/Enrichr/), was used to conduct analyses of GO functional enrichment and KEGG pathway enrichment for the target genes [36, 37]. Subsequently, we entered target genes into the web platform for the purpose of obtaining data regarding GO functional enrichment and KEGG pathway enrichment. GO functional analysis comprises three classifications: cellular component (CC), molecular function (MF), and biological process (BP). We set a *p* value of <0.05 to indicate a statistically significant difference.

### 2.6. Identification of Active Target Genes of DGBXD and Active Compounds

We used the Traditional Chinese Medicine Systems Pharmacology Database and Analysis Platform (TCMSP; https://old.tcmsp-e.com/tcmsp.php) to obtain the chemical compounds and their corresponding target genes in DGBXD (RA and RAS) [38]. Drug-likeness (DL) ≥ 0.18 and oral bioavailability (OB) ≥ 30% were established as thresholds to identify the active compounds, with reference to previous studies [26, 39]. All human gene symbols were corrected to their official gene symbols with the aid of UniProt Knowledgebase (https://www.uniprot.org/) [40, 41].

### 2.7. Determination of Target Genes of DGBXD Acting on IPF

The target genes of the active compounds of DGBXD that overlapped with the target genes of DE-miRNAs in IPF were selected as target genes of DGBXD acting on IPF. According to their corresponding miRNAs, a potential miRNA-mRNA regulatory network of DGBXD acting on IPF was established.

### 2.8. Animal Experiments

We procured C57BL/6 wild-type mice from the Animal Core Facility of Nanjing Medical University (Nanjing Medical University, Nanjing, China). A total of 30 male mice ranging in age from 6 to 8 weeks, weighing 18 to 22 g, were kept in a controlled setting and fed conventional rodent chow, as well as being provided with free access to water. The mice were classified into three groups at random (n = 10 for each group): bleomycin group (Model), control group (Control), and bleomycin + DGBXD group (Treatment). For the purpose of inducing fibrosis, the mice in the model and treatment groups were administered 50 *μ*l of bleomycin (5 mg/kg) (Nippon Kayaku Co., Ltd., Tokyo, Japan), while those in the control group received normal saline (NS) via intratracheal injection, through an endotracheal quantitative microsprayer aerosolizer (Shanghai Yuyan Instruments Co., Ltd., Shanghai, China) [42]. The day after modeling, the control and model groups were intragastrically administered NS, whereas the animals in the treatment group were administered DGBXD once a day for 3 weeks. DGBXD granules were purchased from Jiangyin Tianjiang Pharmaceutical Co., Ltd. (Jiangyin, China) (Table S1). RA and RAS were dissolved with NS at a 5 : 1 ratio. Dosages of DGBXD were set based on their clinical dosages and previous studies [43–45]. The following dosages were used: 4.68 mg/g; RA: 3.90 mg/g, and RAS: 0.78 mg/g (crude drug), calculated according to a body weight of 70 kg for adults using 30 g of RA and 6 g of RAS. Mice were anesthetized and sacrificed on day 21 for further experiments. All of the procedures were approved by the Ethics Committee for Animal Experiments of Nanjing University of Chinese Medicine (A211201).

### 2.9. Histopathological Analysis

On day 21 following the initial therapy, the mice were euthanized and their lungs were extracted for further examination. A 10% formaldehyde solution was used to fix the lung tissue samples, followed by embedding in paraffin and slicing into 5-*μ*m sections. We applied hematoxylin-eosin (HE) and Masson staining to determine whether the lungs had been injured or changed in terms of their morphological appearance. The Szapiel score and Ashcroft score were used to semiquantify the histopathological changes in a blinded manner [46, 47]. The scoring standards are shown in Tables S2 and S3. The scores were assessed separately by two of the authors.

### 2.10. RNA Extraction, Reverse Transcription, and Real-Time Quantitative Polymerase Chain Reaction (qRT-PCR)

RNAiso Plus (Takara) was used for extracting total RNA from the lung tissues of the mice. PrimeScript™ RT Master Mix (Takara) was employed to reverse-transcribe the isolated RNA into complementary DNA (cDNA). Takara's TB Green® Premix Ex Taq™ II (Takara) was applied to quantify the levels of mRNA expression, with GAPDH expression used as an internal reference. The qRT-PCR was carried out using primers with the following sequences: Olr1: forward: 5′-CAA TTT CCC ATA CCA CCT CCC-3′, reverse: 5′-AGT TCC ATT CTC CCA TAG CCA-3′; Hif1a: forward: 5′-ATT TTG GCA GCG ATG ACA CAG-3′, reverse: 5′-CTT TGG AGT TTC CGA TGA AGG TA-3′; and GAPDH: forward: 5′-GAA CGG GAA GCT CAC TGG-3′, reverse: 5′-GCC TGC TTC ACC ACC TTC T-3′. Guangzhou RiboBio Co., Ltd. (Guangzhou, China), supplied the mir-493 and mir-338 primers, and U6 small nuclear RNA (snRNA) as an internal control. In accordance with the manufacturer's instructions, miRNA qRT-PCR was conducted using the Bulge-loop™ miRNA qRT-PCR Starter Kit (Guangzhou RiboBio Co., Ltd.) [48]. Quantification of data was performed by the comparative 2−ΔΔCt method.

### 2.11. Statistical Analysis

Some statistical analyses were carried out directly using the bioinformatic tools available with the aid of the websites indicated above. Only miRNAs or mRNAs with |log2FC| > 1 and adj *p* value < 0.05 were deemed to have statistical significance when differential expression analysis was performed, whether RGui or GEO2R was used. A *p* value < 0.05 was considered to indicate statistical significance in the GO functional enrichment and KEGG pathway enrichment analyses. GraphPad Prism (version: 8.0.2) and IBM SPSS Statistics (version: 25.0) were employed to conduct the qRT-PCR analyses in this study. One-way ANOVA was performed to conduct statistical analysis on the statistics and data assessment. A *p* value < 0.05 was considered to indicate statistical significance.

## 3. Results

### 3.1. Screened miRNA Datasets and Identified DE-miRNAs

**Figure 1:**
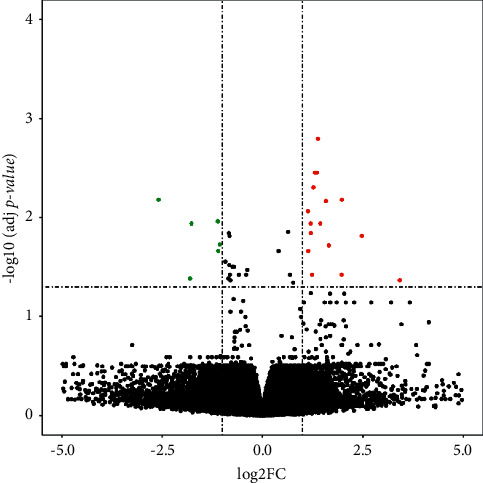
DE-miRNAs between IPF and normal samples. |log2FC| > 1 and adj *p* value < 0.05 were established as the cut-off values in the identification of DE-miRNAs. Six green dots denote downregulated DE-miRNAs and 16 red dots denote upregulated ones; black dots denote miRNAs without differential expression between IPF and normal samples. Finally, six downregulated DE-miRNAs (miR-30b, miR-326, miR-203, miR-338-3p, miR-375, miR-184) and 14 upregulated DE-miRNAs (miR-127-3p, miR-654-3p, miR-409-3p, miR-487b, miR-495, miR-432, miR-369-5p, miR-410, miR-299-5p, miR-382, miR-409-5p, miR-493, miR-154, miR-31) were identified after removing two duplicate DE-miRNAs.

A total of three microarray datasets (GSE13316, GSE27430, and GSE75647) that met the inclusion criteria mentioned above were selected for subsequent analysis. These microarray datasets contained data on IPF and normal samples. The GSE13316 dataset was based on the platform GPL6955, GSE27430 was based on the platform GPL8227, and GSE75647 was based on the platform GPL21199. The three datasets were chosen to filter DE-miRNAs between IPF and normal samples. MiRNAs were discovered directly using GEO2R by designated groups, each of which contained the miRNA expression data. Adj *p* value < 0.05 and |log2FC| > 1 were set as the cut-off values for detecting DE-miRNAs.  Figure 1 shows a volcano plot of the DE-miRNAs.

After removing duplicates, 14 upregulated DE-miRNAs in IPF (miR-127-3p, miR-654-3p, miR-409-3p, miR-487b, miR-495, miR-432, miR-369-5p, miR-410, miR-299-5p, miR-382, miR-409-5p, miR-493, miR-154, miR-31) and six downregulated DE-miRNAs in IPF (miR-30b, miR-326, miR-203, miR-338-3p, miR-375, miR-184) were detected.

### 3.2. Predicted Downstream Target Genes of DE-miRNAs

**Figure 2:**
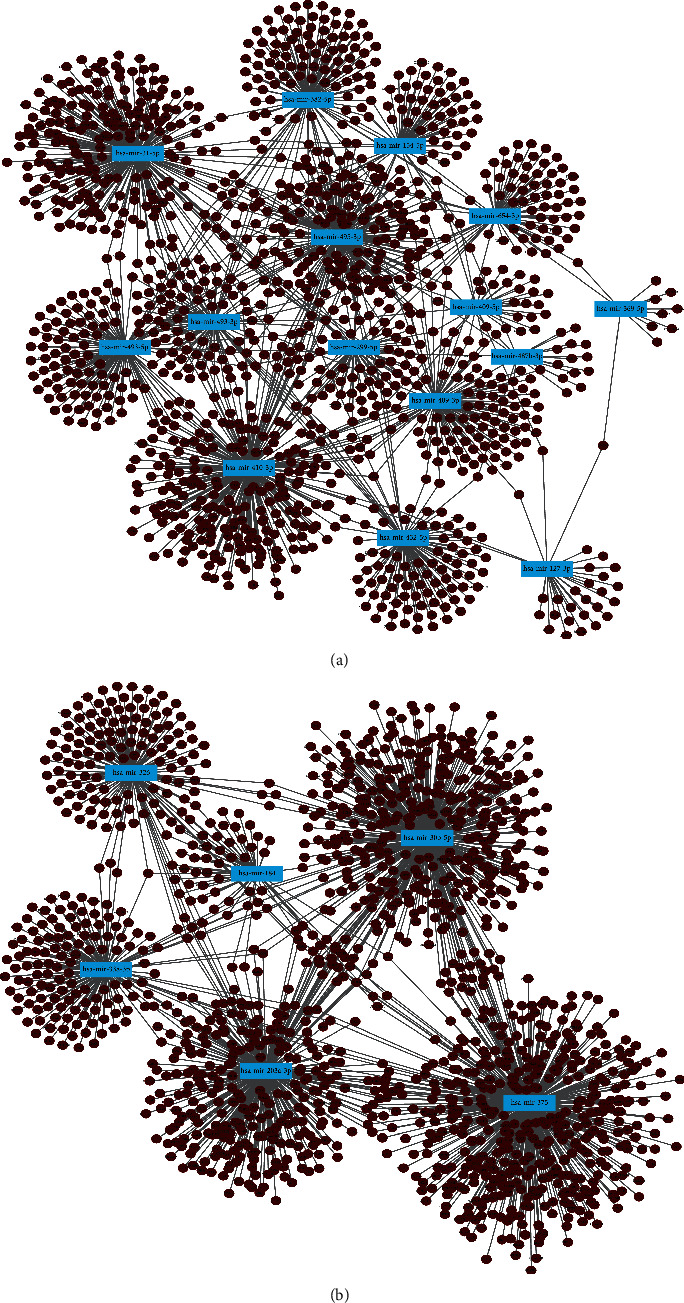
Predicted downstream target genes of DE-miRNAs. (a) miRNet was used for establishing a network of upmodulated DE-miRNAs and their target genes. Blue rectangles represent 15 upmodulated DE-miRNAs (miR-31 is represented in both miR-31-3p and miR-31-5p in miRNet); brown dots represent target genes. (b) miRNet was used for establishing a network of downmodulated DE-miRNAs and their target genes. Blue rectangles represent six downmodulated DE-miRNAs; brown dots represent target genes.

When predicting the downstream target genes of DE-miRNAs, we employed the miRNet database because miRNAs have the greatest potential to exert their biological effects by specifically targeting the 3′ untranslated region of mRNAs. The predicted downstream target genes of the upregulated DE-miRNAs numbered 1285 genes (Table S4), whereas those of the downregulated DE-miRNAs numbered 1411 genes (Table S5). We established a network of the upmodulated DE-miRNA-target genes as depicted in Figure 2(a), and one of the downmodulated DE-miRNA-target genes as shown in Figure 2(b).

### 3.3. Searched mRNA Dataset and Identified DE-mRNAs

The GEO database was searched for the purpose of obtaining datasets containing information about mRNA expression. Datasets based on IPF clinical patients containing data on IPF and normal samples were also included. Finally, one dataset (GSE92592) was chosen for further investigation. The platform GPL11154 served as the foundation for GSE92592.

**Figure 3:**
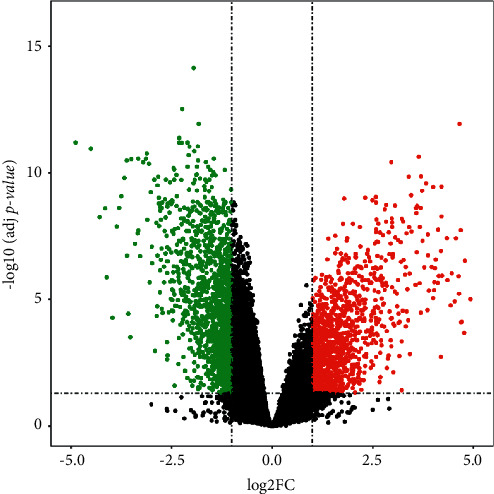
DE-mRNAs between IPF and normal samples. |log2FC| > 1 and adj *p* value < 0.05 were established as cut-off values for the identification of DE-mRNAs. Green and red dots denote the downmodulated and upmodulated mRNAs in IPF samples; black dots denote mRNAs without differential expression between IPF and normal samples.

This dataset was selected for the purpose of determining the DE-mRNAs between IPF and normal samples. A series of matrix files was obtained from the GEO database. Applying RGui and limma package to the analysis of variance, differentially expressed mRNAs were identified. |log2FC| > 1 and adj *p* value < 0.05 were established as the cut-off values for detecting DE-mRNAs. Finally, we identified 1160 upmodulated DE-mRNAs in IPF (Table S6) and 1427 downmodulated ones (Table S7).  Figure 3 shows a volcano plot of the DE-mRNAs.

### 3.4. Identified DE-miRNA Target Genes in IPF

**Figure 4:**
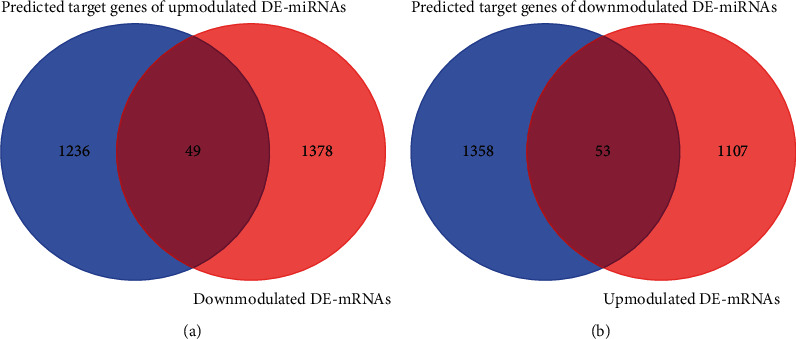
Identified target genes of DE-miRNAs in IPF. (a) The overlap of predicted target genes of upmodulated DE-miRNAs and downmodulated DE-mRNAs. (b) The overlap of predicted target genes of downmodulated DE-miRNAs and upmodulated DE-mRNAs.

There is typically a negative correlation between the level of an miRNA and the level of mRNA expression of its target gene. A combined assessment of 1427 downmodulated DE-mRNAs and 1285 predicted target genes of upmodulated DE-miRNAs was carried out in this study. Subsequently, 49 target genes of the upmodulated DE-miRNAs were discovered (Figure 4(a), Table 1). A combined evaluation of 1160 upmodulated DE-mRNAs and 1411 predicted target genes of downmodulated DE-miRNAs was also performed. Subsequently, 53 target genes of downmodulated DE-miRNAs were discovered (Figure 4(b), Table 2).

### 3.5. GO Functional Enrichment and KEGG Pathway Enrichment

The identified DE-miRNA target genes were subjected to GO functional enrichment and KEGG pathway enrichment analyses, both of which were accomplished using the Enrichr database.

**Figure 5:**
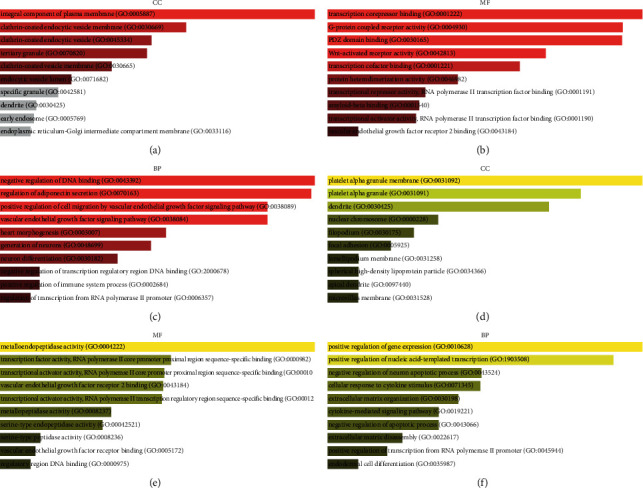
GO functional enrichment of target genes of DE-miRNAs in IPF. (a) The six enriched CC categories of target genes that were downmodulated. (b) The top 10 target genes with high enrichment in MF that were downmodulated. (c) The top 10 enriched BP categories of target genes that were downmodulated. (d) The top 10 target genes with high enrichment in CC that were upmodulated. (e) The top 10 target genes with high enrichment in MF that were upmodulated. (f) The top 10 enriched BP categories of target genes that were upmodulated. Sorted by p value ranking. A darker color reflects a larger *p* value. A gray band indicates *p* value > 0.05. The length indicates combined score.

The findings from GO CC analysis demonstrated that the target genes of the upregulated DE-miRNAs were particularly associated with integral components of the plasma membrane, clathrin-coated endocytic vesicle membrane, clathrin-coated endocytic vesicle, clathrin-coated vesicle membrane, and tertiary granule, among others (Figure 5(a)). Meanwhile, the findings from GO MF analysis demonstrated that the target genes of upregulated DE-miRNAs were particularly related to transcription corepressor binding, PDZ domain binding, transcription cofactor binding, G-protein-coupled receptor activity, and Wnt-activated receptor activity, among others (Figure 5(b)). Finally, the findings from GO BP analysis illustrated that the target genes for the upmodulated DE-miRNAs were strongly associated with negative regulation of DNA binding, regulation of adiponectin secretion, heart morphogenesis, vascular endothelial growth factor (VEGF) signaling pathway, and positive regulation of cell migration by the VEGF signaling pathway, among others (Figure 5(c)).

The results obtained from GO CC analysis showed that the target genes of the downregulated DE-miRNAs were particularly associated with platelet alpha granule, platelet alpha granule membrane, nuclear chromosome, filopodium, and dendrite, among others (Figure 5(d)). Meanwhile, the findings from GO MF analysis demonstrated that the target genes of the downregulated DE-miRNAs were particularly linked with transcription factor activity RNA polymerase II core promoter proximal region sequence-specific binding, transcriptional activator activity RNA polymerase II core promoter proximal region sequence-specific binding, VEGF receptor 2 binding, transcriptional activator activity RNA polymerase II transcription regulatory region sequence-specific binding, and metalloendopeptidase activity, among others (Figure 5(e)). Finally, GO BP analysis showed that the target genes of the downregulated DE-miRNAs demonstrated particular links with extracellular matrix organization, positive regulation of gene expression, positive regulation of nucleic acid-templated transcription, negative regulation of neuron apoptotic process, and cellular response to cytokine stimulus, among others (Figure 5(f)).

**Figure 6:**
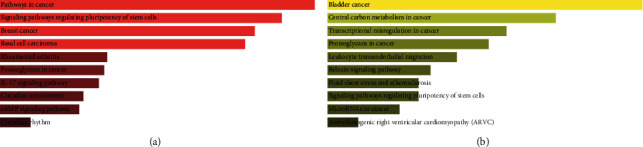
KEGG pathway enrichment of DE-miRNA target genes in IPF. (a) The top 10 target gene pathways with high enrichment that were downmodulated. (b) The top 10 target gene pathways with high enrichment that were upmodulated. Sorted by p value ranking. A darker color reflects a larger *p* value. The length indicates combined score.

KEGG pathway enrichment analysis was also conducted on the genes that were targeted by DE-miRNAs. The results showed that the target genes of the upmodulated DE-miRNAs were particularly associated with pathways in cancer, breast cancer, basal cell carcinoma, proteoglycans in cancer, signaling pathways regulating pluripotency of stem cells, rheumatoid arthritis, cyclic adenosine monophosphate (cAMP), interleukin 17 signaling pathway, circadian entrainment, and circadian rhythm, among others (Figure 6(a)). The target genes for the downmodulated DE-miRNAs were found to be particularly associated with miRNAs, central carbon metabolism, transcriptional misregulation, proteoglycans in cancer, bladder cancer, leukocyte transendothelial migration, relaxin signaling pathway, fluid shear stress and atherosclerosis, signaling pathways regulating pluripotency of stem cells, and arrhythmogenic right ventricular cardiomyopathy, among others (Figure 6(b)).

### 3.6. Identified Active Compounds and Target Genes of DGBXD

This study identified 87 compounds in RA and 125 compounds in RAS, using TCMSP. Among these, 22 active compounds in DGBXD were predicted (20 in RA and 2 in RAS) (Table 3) upon setting the criteria of DL ≥ 0.18 and OB ≥ 30%. TCMSP was also used for determining the target genes for each of these 22 compounds (Table S6). The gene symbols were filtered using UniProt Knowledgebase, with the format “Homo sapiens” being used to identify them. Finally, we acquired a total of 196 active target genes of DGBXD (Table 4).

### 3.7. Identified Target Genes of DGBXD Acting on IPF

**Figure 7:**
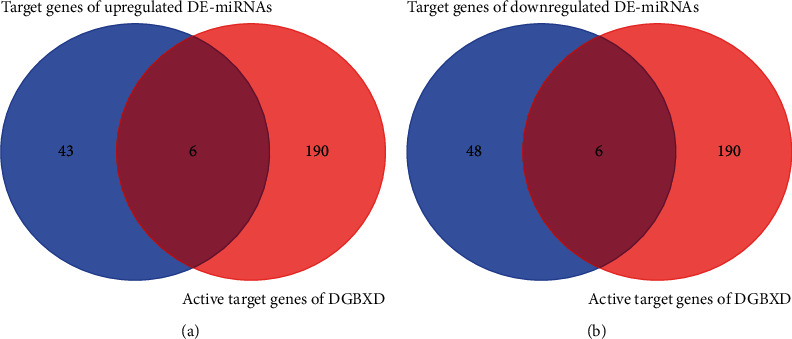
Identified target genes of DGBXD acting on IPF. (a) The overlap of the 196 active target genes of DGBXD with the 49 target genes of upregulated DE-miRNAs was determined, which identified six downregulated target genes of DGBXD acting on IPF. (b) The overlap of the 196 active target genes of DGBXD with the 53 target genes of downregulated DE-miRNAs was determined, which identified six upregulated target genes of DGBXD acting on IPF.

We determined those genes among the 196 active target genes of DGBXD that overlapped with the 49 target genes of upmodulated DE-miRNAs and the 53 target genes of downmodulated DE-miRNAs. This led to identification of the target genes through which DGBXD acts on IPF, namely, six downmodulated genes (Figure 7(a)) and six upmodulated ones (Figure 7(b)).

### 3.8. Identified Putative miRNA-mRNA Regulatory Network of DGBXD Acting on IPF

On the basis of the identified miRNA and target gene pairs (Tables 1 and 2), the relationship between the miRNAs and target genes of DGBXD through which it acts on IPF was characterized (Table 5) and a putative miRNA-mRNA regulatory network involved in the action of DGBXD on IPF was developed. According to the correlations between the active compounds of DGBXD and its target genes (Table S8), further study should be performed on potential compounds in DGBXD acting on IPF through the miRNA-mRNA regulatory network.

### 3.9. Experimental Validation of a Bleomycin-Induced IPF Model

**Figure 8:**
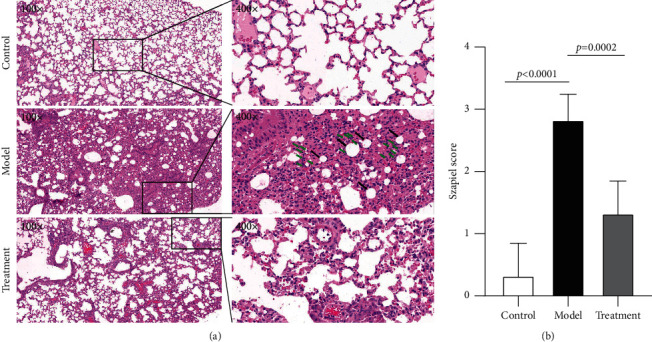
HE staining of lung samples. (a) On day 21, extracellular matrix proliferation and injured alveolar structure emerged visibly (black arrow), while fibroblasts (green arrow) appeared in the model group. The DGBXD treatment group exhibited slight preservation of alveolar structure and suppression of pulmonary interstitial hyperplasia on day 21. (b) Assessment by the Szapiel score. The Szapiel score was significantly higher than in the control group, whereas the DGBXD treatment group was improved.

To duplicate the establishment of IPF models and determine the therapeutic effects of DGBXD, HE staining was carried out to assess the pathological alterations in the various studied groups. After 21 days, extracellular matrix hyperplasia and injured alveolar structure visibly emerged, with fibroblasts appearing in the model group. On day 21, the alveolar structure was somewhat conserved in the DGBXD treatment group and the pulmonary interstitial hyperplasia was attenuated (Figure 8(a)). Additionally, the Szapiel score was significantly higher than in the control group, whereas the DGBXD treatment group was improved (Figure 8(b)). These results indicated that DGBXD could optimize the structure of the alveoli in bleomycin-induced IPF.

**Figure 9:**
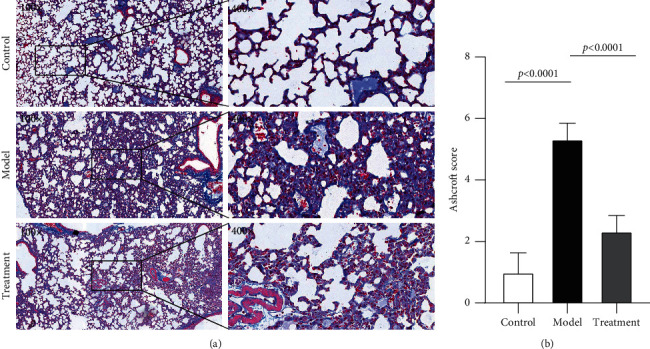
Masson staining of lung samples. (a) On day 21, a large number of blue-dyed collagen fibers were observed in the interstitial lung fluid of the model group. On day 21, mild lung fibrosis was observed in the DGBXD treatment groups. (b) Assessment by the Ashcroft score. The Ashcroft score was significantly higher than in the control group, whereas the DGBXD treatment group was improved.

The extent of pulmonary fibrosis was evaluated among the groups using Masson staining. On day 21, a large number of blue-dyed collagen fibers were found in the lung interstitial fluid of the model group. Moreover, on day 21 following treatment, mild pulmonary fibrosis was observed in the DGBXD-treated group (Figure 9(a)). Additionally, the Ashcroft score was significantly higher than in the control group, whereas the DGBXD treatment group was improved (Figure 9(b)).

### 3.10. Validation of miRNA-mRNA Pairs in the miRNA-mRNA Regulatory Network

**Figure 10:**
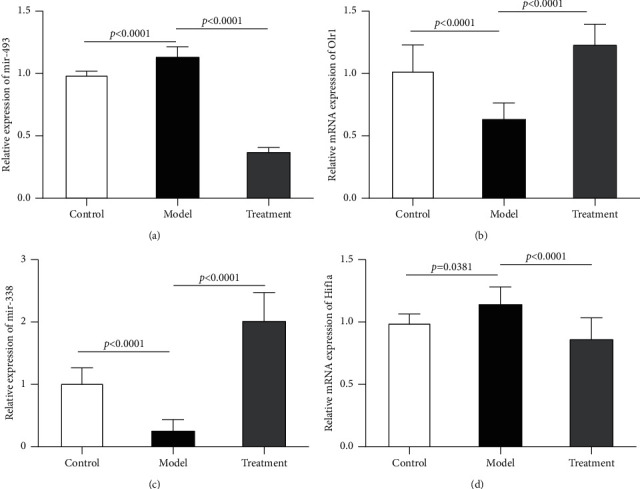
Validation of miRNA-mRNA pairs using qRT-PCR. (a) Substantial elevation in mir-493 expression level was observed in the model group in contrast with that in the control group. Nevertheless, considerable attenuation in mir-493 expression was observed in the treatment group compared with that in the model group. (b) The mRNA expression of *Olr1* was dramatically attenuated in the model group in comparison with that in the control group, whereas it was substantially elevated in the treatment group compared with that in the model group, with a statistically significant difference. (c) mir-338 expression was shown to undergo significant attenuation in the model group compared with that in the control group, while being substantially elevated in the treatment group compared with that in the model group. (d) The Hif1a mRNA expression was found to be substantially elevated in the model group compared with that in the control group, while being substantially reduced in the treatment group compared with that in the model group.

We used qRT-PCR to investigate the expression level of mir-493 along with its target gene Olr1, as well as the expression level of mir-338 along with its target gene Hif1a, in lung tissues. In full agreement with the predicted results, the experimental validation demonstrated that mir-493 expression was remarkably elevated in the IPF model group, compared with that in the control group, and considerably attenuated in the DGBXD treatment group compared with that in the model group (Figure 10(a)). Moreover, the mRNA expression of Olr1 was shown to have significantly decreased in the IPF model group compared with that in the control group and to be remarkably elevated in the DGBXD treatment group compared with that in the model group (Figure 10(b))

mir-338 expression was also significantly attenuated in the IPF model group compared with that in the control group and significantly elevated in the DGBXD treatment group compared with that in the model group (Figure 10(c)). Finally, the mRNA expression of Hif1a was shown to be markedly increased in the IPF model group compared with that in the control group and substantially attenuated in the DGBXD treatment group compared with that in the model group (Figure 10(d)).

## 4. Discussion

IPF is an interstitial disease with UIP as its main pathological manifestation. IPF cannot currently be cured and its prognosis remains poor [49, 50]. Although many related studies have been reported, the mechanism behind the occurrence and development of IPF are still not very clear. Thus far, studies have shown that epithelial-mesenchymal transition (EMT) and myofibroblast differentiation may be involved in the onset and progression of IPF [51–53]. Moreover, mitochondrial dysfunction and metabolic reprogramming are considered drivers of IPF [54].

Several studies explained the expression and function of miRNA have focused on the functions of miRNAs in cell proliferative ability, apoptosis, migratory function, differentiation, and energy metabolism [15–19]. MiRNAs are also closely related to EMT and myofibroblast differentiation [55–58].

Various studies have demonstrated that miRNA-mRNA regulation performs indispensable functions in the respiratory system. According to the results of an integrative investigation of miRNA-mRNA expression in nonsmall cell lung cancer, miRNAs might be a major modulatory factor regulating basic cellular activities and cell differentiation [59, 60]. Moreover, integrative analysis of miRNA-mRNA expression indicated that miRNA-mRNA expression is associated with the influenza virus [61]. Changes in the expression profiles of miRNA-mRNA in lung tissue in a mouse model also revealed the pathophysiology of bronchopulmonary dysplasia [62].

Recently, a putative IPF-related miRNA-mRNA regulatory network was established [23]. A search of the GEO database was carried out in accordance with previously reported methods, and differential expression analysis was carried out with the aid of miRNA and mRNA microarray datasets. As a result, 14 DE-miRNAs were found to be upmodulated in IPF, whereas six DE-miRNAs were downmodulated. The majority of DE-miRNA expression patterns were in line with those observed in earlier studies. For instance, miR-31 was reported to be considerably upregulated in the serum of IPF patients, compared with the level in healthy controls [63], and the levels of miR-31 expression were found to be positively correlated with the levels of SMAD2/AKT and SMAD6 expression in patients with IPF, while transforming growth factor-*β* (TGF-*β*) was found to induce miR-31 levels in A549 cells [64]. The expression levels of miR-410, miR-382, miR-299-5p, miR-369-5p, miR-409-3p, miR-487b, miR-127-3p, miR-493, miR-409-5p, and miR-154 are also higher in IPF [65]. Moreover, the expression levels of miR-410 and TGF-*β*1 are high in lung tissue of fibrosis model rats [66]. Furthermore, the expression of miR-154 is upmodulated in the lungs of individuals experiencing pulmonary fibrosis [67]. TGF-*β* suppresses the level of miR-184 in A549 cells, elevates miR-184, and ameliorates the viability of A549 cells induced by TGF-*β* [64]. MiR-338 reduces EMT and delays the development of pulmonary fibrosis [68–70]. Meanwhile, miR-326 modulates the expression of TGF-*β*1 and alleviates lung fibrosis [71], and miR-326 suppresses the inflammatory response and enhances autophagy in pulmonary fibrosis induced by silica [72]. However, inconsistent results have been reported. One study found that the suppression of miR‐495‐3p could promote sphingosine-1-phosphate receptor 3 (S1PR3) expression in pulmonary epithelia and that overexpressed miR‐495‐3p could inhibit the S1PR3/SMAD2/SMAD3 pathway and suppress EMT [73].

After integrating DE-mRNAs and their anticipated target genes, several target genes of DE-miRNAs involved in IPF were obtained, which included 49 target genes for upmodulated DE-miRNAs and 53 target genes for downmodulated ones. Subsequently, GO functional enrichment and pathway analyses were conducted. Pathway enrichment analysis illustrated that the target genes were particularly associated with cancer-related pathways, rheumatoid arthritis, signaling pathways modulating pluripotency of stem cells, central carbon metabolism, miRNAs, transcriptional misregulation, fluid shear stress, and atherosclerosis. These functions and pathways are related to mitochondrial activity and metabolism. For instance, the gene expression programs that establish and maintain specific cell states are controlled by thousands of transcription factors, cofactors, and chromatin regulators, whose misregulation can cause a broad range of diseases [74]. Metabolic reprogramming in tumors is closely related to autophagy and mitophagy [75]. Intestinal microbiota metabolism is associated with atherosclerosis [76]. The role of cancer stem cell metabolism in carcinogenesis is a major focus in cancer research [77]. Mitochondrial dysfunction is associated with the initiation and progression of atherosclerosis by elevating the production of reactive oxygen species and mitochondrial oxidative stress damage, mitochondrial dynamics dysfunction, and energy supply [78]. MiRNAs involved in mitochondrial metabolism, mitochondrial oxidative phosphorylation, electron transport chain components, lipid metabolism, and metabolic disorders are very important and closely related to mitochondrial dynamics and cancer [79]. The inhibition of dynamin-related protein 1 (DRP1) and mitochondrial fission attenuates inflammatory response in fibroblast-like synoviocytes of rheumatoid arthritis [80]. Mitochondrial fission activity is associated with high proliferation and invasiveness in some cancer cells and with self-renewal and resistance to differentiation in some stem cells [81]. Mitochondrial dynamics and dysfunction are related to mitochondrial DNA defects, excessive fission, mitochondrial retrograde signaling, and cancer progression [82]. These functions and pathways mentioned above are related to mitochondrial activity and metabolism, which suggests that the target genes of DE-miRNAs in IPF are also related to mitochondrial function and metabolism.

Some research reports have demonstrated that TCM can exert effects in regulating miRNA, mitochondrial function, and metabolism. For example, by regulating miRNA, TCM can promote liver regeneration in rat models of acute liver failure, ameliorate cyclosporin A-induced chronic nephrotoxicity, and treat coronary heart disease [20–22]. TCM has also been reported to attenuate myocardial ischemia/reperfusion injury by preserving mitochondrial function [83]. Studies have also shown that TCM can treat cardiovascular disease by protecting mitochondrial function [84]. Furthermore, metabolic reprogramming in TCM treatment of hypertension has received increasing attention [85], and metabolic reprogramming by TCM is also important for effective cancer therapy [86].

DGBXD, a TCM formulation, was first described by Li Dongyuan in the differentiation of endogenous and exogenous disorders (Nei Wai Shang Bian Huo Lun in Chinese) in 1247 CE. This herbal formula contains RA and RAS. DGBXD consists of the combination of herbs of 30 g of RA and 6 g of RAS [24]. According to TCM theory, DGBXD can be used for nourishing qi and enriching blood [87]. DGBXD can improve lung, spleen, and kidney deficiency, strengthen the blood circulation to remove blood stasis, and make qi flourishing stasis, in line with the pathological mechanism of IPF, which is also considered to be related to mitochondria and energy metabolism in modern medicine [88, 89].

A systematic review of RA and RAS in the treatment of IPF was previously performed, which confirmed their effectiveness and safety in IPF patients [25]. Network pharmacology studies were also carried out, clarifying the multipathway, multitarget, and multicomponent mechanism by which RA and RAS act in the treatment of IPF [26, 27]. These studies also provided a basis for in-depth study of the use of DGBXD to treat IPF.

Using a network pharmacology approach, 196 target genes of DGBXD, as well as 22 active compounds, were obtained in this study. The 196 active target genes of DGBXD were intersected with the target genes for DE-miRNAs, out of which 49 were upmodulated and 53 were downmodulated in IPF, resulting in the identification of six downmodulated and six upmodulated target genes of DGBXD that play certain roles in IPF. Some of these genes were associated with mitochondrial function and metabolism. Mitochondrial dysfunction is closely related to nuclear factor-*κ*B and oxidized low-density-lipoprotein receptor 1 (OLR1) in atrial tissue during atrial fibrillation [90]. The association of OLR1 with angiotensin II type 1 receptor (AT1R) plays a crucial role in regulating mitochondrial quality control [91]. Mitochondrial dysfunction and metabolism are also closely related to hypoxia inducible factor-1*α* (HIF1A), mitochondrial dysfunction represses HIF1A protein synthesis, and mitochondrial defect and stabilization of HIF1A act synergistically to activate glycolysis [92, 93].

Following the analysis of the miRNA and target gene pairs mentioned above, it was discovered that there is a correlation between the miRNA and target genes of DGBXD that act on IPF. Thus, in this study, a putative miRNA-mRNA regulatory network of DGBXD acting on IPF was proposed, which includes miR-654-3p-PKIA/ADRB1, miR-493-5p-FOS/OLR1, miR-410-3p/miR-495-3p-VEGFA, and miR-493-3p-MAP2 of upregulated miRNA and downregulated gene modulatory network; miR-203a-3p-TOP2A/MMP1/TP63 and miR-338-3p-MMP2/HIF1A/MMP9 of downregulated miRNA and upregulated gene modulatory network. With regard to regulatory networks, few reports on them in human diseases have been published, and IPF-related issues have received little attention. For instance, the overexpression of secreted protein acidic and rich in cysteine (SPARC) reduces vascular endothelial growth factor-A (VEGFA) expression in NB1691 neuroblastoma cells via miR-410; in addition, the overexpression of SPARC combined with miR-410 was demonstrated to be more effective at alleviating angiogenesis, whereas the administration of miR-410 blockers attenuated the suppression of VEGFA mediated by SPARC in NB1691 cells [94]. A DNA methyltransferase inhibitor, 5-AzaC, has also been shown to increase miR-495 expression, while decreasing the expression of its target gene signal transducer and activator of transcription 3 (STAT3), as well as the expression of its downstream target VEGF. These anticancer characteristics of 5-AzaC have also been confirmed in breast cancer cells [95]. In the initiation and progression of nasopharyngeal carcinoma, miR-338-3p inhibits migratory and proliferative abilities by targeting HIF1A directly, which could represent a novel therapeutic target [96]. These studies may have identified the potential targets for treating related diseases. In particular, the miRNA-mRNA regulatory network of DGBXD acting on IPF warrants further research, with the aim of verifying the related potential mechanism. The majority of the miRNA-mRNA pairs included in the network established in this study and possibly leading to the pathophysiology of IPF have not previously been explored, so studies of them are required to investigate and discover novel disease processes and treatment targets.

On the basis of previous animal model studies of the action of DGBXD on IPF, the dosage of DGBXD used and the effect of DGBXD in bleomycin-induced IPF animal model are corroborative [97–101]. Then, a bleomycin-induced IPF mouse model was duplicated, and the dosage of DGBXD intragastrically administered was calculated according to a body weight of 70 kg for adults using 30 g of RA and 6 g of RAS. Finally, the results of HE staining and Masson staining of lung sections confirmed the therapeutic effects of DGBXD. These results are consistent with previous animal model studies on IPF, and DGBXD clearly has an effect in bleomycin-induced IPF mice, which provides a basis for further mechanistic research.

After we replicated the IPF model for treatment with DGBXD, we primarily performed qRT-PCR validation of the abovementioned miRNA-mRNA pairs. Not unnaturally, genes associated with mitochondrial function and metabolism could be investigated for the first time. The expression level of mir-493 along with its target gene *Olr1* and the expression level of miR-338 along with its target gene Hif1a were explored in lung tissues by qRT-PCR. mir-493 expression was substantially upmodulated in IPF groups, whereas the gene Olr1 was downregulated, and mir-338 expression was substantially downmodulated in the IPF group along with the upregulation of Hif1a. In addition, mir-493 expression was remarkably attenuated in the DGBXD groups along with an increase in Olr1, whereas mir-338 expression was considerably elevated in the DGBXD groups along with a decrease in Hif1a. These validation results are consistent with the regulatory network that we established, which is very important and meaningful. These miRNA-mRNA pairs associated with mitochondrial dysfunction and metabolic reprogramming could thus be prioritized for further investigation in basic research. According to the active compounds previously obtained, an HIF1A-associated compound is quercetin, and an OLR1-associated compound is isorhamnetin, which can provide a reference regarding the mechanism by which DGBXD acts on IPF through the miRNA-mRNA regulatory network.

Although a putative miRNA-mRNA regulatory network of DGBXD acting on IPF has been developed in this study, this work had several limitations. First, the IPF-related miRNA and mRNA microarray datasets used were mainly from the GEO database, whereas the other databases have less-relevant datasets. The number and sample size of the microarray datasets included in this study may thus have been insufficient, although we collected miRNA and mRNA microarray datasets as comprehensively as possible from the GEO database. Nonetheless, each dataset contains raw data from relevant clinical studies and a single dataset can display all of the obtained miRNA- or mRNA-related information, and these microarray datasets are also complete and reliable. Second, employing TCMSP, we successfully identified the active chemicals as well as the target genes in RA and RAS. However, the data collection method was not exhaustive and the criteria for screening for active chemicals could potentially have led to bias, but TCMSP is currently the most-extensive accessible database and most of the common active chemicals and target genes in RA and RAS in the network database can be identified in TCMSP. In fact, part of the active chemicals and target genes in RA and RAS may be missed including some active chemicals through quality control index of RA and RAS in Chinese Pharmacopoeia. Therefore, although we established the potential miRNA-mRNA regulatory network through the network database, some of the miRNA-mRNA pairs may have been missed for the above reasons. However, our purpose in this study was to find potential miRNA-mRNA pairs involved in DGBXD acting on IPF from the predictions and then perform validation. Overall, the established miRNA-mRNA regulatory network of DGBXD acting on IPF in this study is still reliable and significant, especially the experimentally validated miRNA-mRNA pairs. Finally, because we further validated the gene expression and miRNA-mRNA pairs in the regulatory network using an IPF mouse model, the validated miRNA-mRNA pairs for specific deep mechanisms require further investigation. In addition, further clinical research is required to validate the regulatory network, as well as its relationships to clinical efficacy and prognosis.

## 5. Conclusion

This study revealed a potential mechanism of involvement of miRNA-mRNA modulatory axes in the pathogenic mechanisms of IPF. It also developed a putative IPF-related miRNA-mRNA regulatory network through which DGBXD ameliorates IPF. Finally, relevant miRNA-mRNA pairs were experimentally verified to facilitate further research, and potential compounds in DGBXD acting on IPF through the miRNA-mRNA regulatory network can now also be further studied.

## Figures and Tables

**Table 1:** Target genes of upmodulated DE-miRNAs in IPF.

Target genes	Upregulated DE-miRNAs	Target genes	Upregulated DE-miRNAs
ZNF331	miR-31-5p	CABP4	miR-410-3p
CASKIN2	miR-31-5p	HHIP	miR-410-3p
SPRY4	miR-31-5p	RAB11FIP1	miR-410-3p
FOXD4L1	miR-31-5p	CADM2	miR-410-3p
MAPK4	miR-127-3p	FOS	miR-493-5p
PER3	miR-154-5p	OLR1	miR-493-5p
VSIG2	zmiR-154-5p	CITED2	miR-493-5p
			miR-493-3p
PTGDR	miR-382-5p	FREM2	miR-493-5p
FAM105 A	miR-382-5p	ID4	miR-432-5p
SYT13	miR-382-5p	KCNK16	miR-432-5p
FABP4	miR-369-5p	MOGAT1	miR-432-5p
GAB1	miR-369-5p	SEC14L4	miR-432-5p
	miR-409-3p		
IFNG	miR-409-3p	PER2	miR-495-3p
EFR3B	miR-409-3p	DLC1	miR-495-3p
SLC26A7	miR-409-3p	GALNT8	miR-495-3p
CCDC38	miR-409-3p	CCDC141	miR-495-3p
	miR-410-3p		
HCAR2	miR-409-3p	LIFR	miR-493-3p
KLF6	miR-410-3p	MAP2	miR-493-3p
CD55	miR-410-3p	FZD4	miR-493-3p
DUSP8	miR-410-3p	GRM3	miR-487b-3p
VEGFA	miR-410-3p	ADRB1	miR-654-3p
	miR-495-3p		
FZD5	miR-410-3p	PKIA	miR-654-3p
ARHGAP29	miR-410-3p	CDKL2	miR-654-3p
SLC25A27	miR-410-3p	PRSS21	miR-654-3p
HEY1	miR-410-3p		

DE-miRNA: differentially expressed microRNA; IPF: idiopathic pulmonary fibrosis.

**Table 2:** Target genes of downmodulated DE-miRNAs in IPF.

Target genes	Downregulated DE-miRNAs	Target genes	Downregulated DE-miRNAs
DLX5	miR-203a-3p	CRABP2	miR-375
HTR2A	miR-203a-3p	KCNN4	miR-375
IGFBP5	miR-203a-3p	OTX1	miR-375
MMP1	miR-203a-3p	PDK1	miR-375
MMP10	miR-203a-3p	RAB3B	miR-375
SIX1	miR-203a-3p miR-30b-5p	SOX2	miR-375
TOP2A	miR-203a-3p	CLDN1	miR-375
TP63	miR-203a-3p	CRLF1	miR-375
IL24	miR-203a-3p	ARNTL2	miR-375
GREM1	miR-203a-3p	CHPF	miR-375
PSAT1	miR-203a-3p	SAMD11	miR-375
MRO	miR-203a-3p miR-30b-5p	C1R	miR-326
C15orf48	miR-203a-3p	CLU	miR-326
MSI2	miR-203a-3p	HMGA2	miR-326
SPATA18	miR-203a-3p	MTHFD2	miR-326
ACTC1	miR-30b-5p	CAPN5	miR-338-3p
MYBL2	miR-30b-5p	CDH2	miR-338-3p
RRM2	miR-30b-5p	HIF1A	miR-338-3p
SLC7A5	miR-30b-5p miR-184 miR-338-3p	ITGB3	miR-338-3p
SIX4	miR-30b-5p	MAP1A	miR-338-3p
MARCH4	miR-30b-5p	MMP2	miR-338-3p
TXNDC5	miR-30b-5p	MMP9	miR-338-3p
CTHRC1	miR-30b-5p	PCDH7	miR-338-3p
FAM81 B	miR-30b-5p	NCS1	miR-338-3p
IFNE	miR-30b-5p	FJX1	miR-338-3p
TNFRSF13 C	miR-184	SCARA3	miR-338-3p
CALU	miR-375		

DE-miRNA: differentially expressed microRNA; IPF: idiopathic pulmonary fibrosis.

**Table 3:** Active compounds in DGBXD determined by TCMSP.

Herb	Active compound	Compound id	OB (%)	DL
RA	Mairin	MOL000211	55.38	0.78
RA	Jaranol	MOL000239	50.83	0.29
RA	Hederagenin	MOL000296	36.91	0.75
RA	(3 S, 8 S, 9 S, 10 R, 13 R, 14 S, 17 R)-10, 13-Dimethyl-17-[(2 R, 5 S)-5-propan-2-yloctan-2-yl]-2, 3, 4, 7, 8, 9, 11, 12, 14, 15, 16, 17-dodecahydro-1h-cyclopenta [a] phenanthren-3-ol	MOL000033	36.23	0.78
RA	Isorhamnetin	MOL000354	49.60	0.31
RA	3, 9-di-O-Methylnissolin	MOL000371	53.74	0.48
RA	5′-Hydroxyiso-muronulatol-2′, 5′-di-O-glucoside	MOL000374	41.72	0.69
RA	7-O-Methylisomucronulatol	MOL000378	74.69	0.30
RA	9, 10-Dimethoxypterocarpan-3-O-*β*-D-glucoside	MOL000379	36.74	0.92
RA	(6aR, 11aR)-9, 10-Dimethoxy-6a, 11a-dihydro-6h-benzofurano [3, 2-c]chromen-3-ol	MOL000380	64.26	0.42
RA	Bifendate	MOL000387	31.10	0.67
RA	Formononetin	MOL000392	69.67	0.21
RA	Isoflavanone	MOL000398	109.99	0.30
RA	Calycosin	MOL000417	47.75	0.24
RA	Kaempferol	MOL000422	41.88	0.24
RA	FA	MOL000433	68.96	0.71
RA	(3R)-3-(2-Hydroxy-3, 4-dimethoxyphenyl)chroman-7-ol	MOL000438	67.67	0.26
RA	Isomucronulatol-7, 2′-di-O-glucosiole	MOL000439	49.28	0.62
RA	1, 7-Dihydroxy-3, 9-dimethoxy pterocarpene	MOL000442	39.05	0.48
RA	Quercetin	MOL000098	46.43	0.28
RAS	Beta-sitosterol	MOL000358	36.91	0.75
RAS	Stigmasterol	MOL000449	43.83	0.76

DGBXD: Danggui Buxue decoction; TCMSP: Traditional Chinese Medicine Systems Pharmacology database and analysis platform; OB: oral bioavailability; DL: drug-likeness; RA: Radix Astragali; RAS: Radix Angelicae Sinensis.

**Table 4:** Active target genes of DGBXD.

PGR	ADRA2A	OLR1	HAS2	TOP1	TOP2A
NCOA2	SLC6A2	HTR3A	GSTP1	RAF1	ABCG2
PTGS1	SLC6A3	ADRA2C	AHR	SOD1	NFE2L2
PTGS2	AKR1B1	ADRA1D	PSMD3	HIF1A	NQO1
HSP90AA1	PLAU	CHRM5	SLC2A4	RUNX1T1	PARP1
KCNH2	LTA4H	OPRD1	NR1I3	HSPA5	COL3A1
DRD1	MAOB	RXRB	INSRR	ERBB2	CXCL11
CHRM3	MAOA	KDR	DIO1	ACACA	CXCL2
CHRM1	CTRB1	MET	PPP3CA	CAV1	DCAF5
SCN5A	ADRB1	PKIA	GSTM1	MYC	CHEK2
CHRM4	NOS2	IL4	GSTM2	F3	CLDN4
ADRA1A	AR	ATP5F1B	AKR1C3	GJA1	PPARA
CHRM2	ESR2	ND6	SLPI	IL1B	HSF1
ADRA1B	DPP4	HSD3B2	MMP3	CCL2	CRP
ADRB2	CDK2	HSD3B1	EGFR	PTGER3	CXCL10
CHRNA2	CHEK1	IKBKB	VEGFA	IL8RA	CHUK
SLC6A4	PRSS1	AKT1	CCND1	PRKCB	SPP1
OPRM1	CALM1	TNFSF15	BCL2L1	BIRC5	RUNX2
GABRA1	GRIA2	AHSA1	FOS	DUOX2	RASSF1
BCL2	ADH1B	MAPK8	CDKN1A	NOS3	E2F1
BAX	LYZD1	MMP1	EIF6	HSPB1	E2F2
CASP9	ESR1	STAT1	MMP2	SULT1E1	ACPP
JUN	PPARG	CDK1	MMP9	MGAM	CTSD
CASP3	MAPK14	HMOX1	MAPK1	IL2	IGFBP3
CASP8	GSK3B	CYP3A4	IL10RB	CCNB1	IGF2
PRKCA	CCNA2	CYP1A2	EGF	PLAT	CD40LG
PON1	PYGM	CYP1A1	RB1	THBD	IRF1
MAP2	PPARD	ICAM1	IL6	SERPINE1	ERBB3
NR3C2	F7	SELE	TP63	COL1A1	PCOLCE
ADH1C	HTR	VCAM1	ELK1	IFNGR1	NPEPPS
IGHG1	ACHE	NR1I2	NFKBIA	IL1A	HK2
RXRA	RELA	CYP1B1	POR	MPO	RASA1
NCOA1	NCF1	ALOX5	ODC1		

DGBXD: Danggui Buxue decoction.

**Table 5:** Target genes of DGBXD acting on IPF and corresponding miRNA.

Target genes	Corresponding miRNA	
Downregulated target genes	PKIA	miR-654-3p
	FOS	miR-493-5p
	ADRB1	miR-654-3p
	VEGFA	miR-410-3p
		miR-495-3p
	MAP2	miR-493-3p
	OLR1	miR-493-5p

Upregulated target genes	MMP2	miR-338-3p
	TOP2A	miR-203a-3p
	HIF1A	miR-338-3p
	MMP1	miR-203a-3p
	TP63	miR-203a-3p
	MMP9	miR-338-3p

DGBXD: Danggui Buxue decoction; IPF: idiopathic pulmonary fibrosis; miRNA: microRNA.

## Data Availability

Data can be obtained from the corresponding author upon written request.

## Supplementary Materials

Supplementary Materials Table S1: DGBXD granules. Table S2: Szapiel score system. Table S3: Ashcroft score system. Table S4: predicted target genes of upregulated DE-miRNAs (n = 1285). Table S5: predicted target genes of downregulated DE-miRNAs (n = 1411). Table S6: upregulated DE-mRNAs (n = 1160). Table S7: downregulated DE-mRNAs (n = 1427). Table S8: corresponding gene symbols of RA and RAS.
