# Supplementary material for: Danggui Buxue Decoction Ameliorates Idiopathic Pulmonary Fibrosis through MicroRNA and Messenger RNA Regulatory Network
Source: Evid Based Complement Alternat Med. 2022 Apr 26;2022:3439656. doi: 10.1155/2022/3439656 (PMC9064538; doi:10.1155/2022/3439656)
Supplement: Supplementary Materials — Table S1: DGBXD granules. Table S2: Szapiel score system. Table S3: Ashcroft score system. Table S4: predicted target genes of upregulated DE-miRNAs (n = 1285). Table S5: predicted target genes of downregulated DE-miRNAs (n = 1411). Table S6: upregulated DE-mRNAs (n = 1160). Table S7: downregulated DE-mRNAs (n = 1427). Table S8: corresponding gene symbols of RA and RAS. [file 3439656.f1.zip › 3439656.f1/Table S1 DGBXD granules.docx]

**Table S1:** DGBXD granules.

| **Granules** | **Product batch** | **Packing size**  **(g/package)** | **Equivalent to crude drug (g)** |
| --- | --- | --- | --- |
| RA | 20081371 | 1.5 | 10.0 |
| RAS | 21030021 | 4.0 | 10.0 |

DGBXD: Danggui Buxue decoction; RA: *Radix Astragali*; RAS: *Radix Angelicae Sinensis*.
