# Supplementary material for: Danggui Buxue Decoction Ameliorates Idiopathic Pulmonary Fibrosis through MicroRNA and Messenger RNA Regulatory Network
Source: Evid Based Complement Alternat Med. 2022 Apr 26;2022:3439656. doi: 10.1155/2022/3439656 (PMC9064538; doi:10.1155/2022/3439656)
Supplement: Supplementary Materials — Table S1: DGBXD granules. Table S2: Szapiel score system. Table S3: Ashcroft score system. Table S4: predicted target genes of upregulated DE-miRNAs (n = 1285). Table S5: predicted target genes of downregulated DE-miRNAs (n = 1411). Table S6: upregulated DE-mRNAs (n = 1160). Table S7: downregulated DE-mRNAs (n = 1427). Table S8: corresponding gene symbols of RA and RAS. [file 3439656.f1.zip › 3439656.f1/Table S2 Szapiel score system.docx]

**Table S2:** Szapiel score system.

| **Degree of alveolitis** | **Histopathologic features** |
| --- | --- |
| 0 | No alveolitis. |
| 1 | Thickening of the alveolar septum by a mononuclear cell infiltrate, with involvement limited to focal, pleural-based lesions occupying less than 20% of the lung and with good preservation of the alveolar architecture. |
| 2 | A more widespread alveolitis involving 20%–50% of the lung, although still predominantly pleural based. |
| 3 | A diffuse alveolitis involving >50% of the lung, with occasional consolidation of air spaces by the intra-alveolar mononuclear cells and some hemorrhagic areas within the interstitium and/or alveolus. |
