# Supplementary material for: Danggui Buxue Decoction Ameliorates Idiopathic Pulmonary Fibrosis through MicroRNA and Messenger RNA Regulatory Network
Source: Evid Based Complement Alternat Med. 2022 Apr 26;2022:3439656. doi: 10.1155/2022/3439656 (PMC9064538; doi:10.1155/2022/3439656)
Supplement: Supplementary Materials — Table S1: DGBXD granules. Table S2: Szapiel score system. Table S3: Ashcroft score system. Table S4: predicted target genes of upregulated DE-miRNAs (n = 1285). Table S5: predicted target genes of downregulated DE-miRNAs (n = 1411). Table S6: upregulated DE-mRNAs (n = 1160). Table S7: downregulated DE-mRNAs (n = 1427). Table S8: corresponding gene symbols of RA and RAS. [file 3439656.f1.zip › 3439656.f1/Table S3 Ashcroft score system.docx]

**Table S3:** Ashcroft score system.

| **Grade of fibrosis** | **Histologic features** |
| --- | --- |
| 0 | Normal lung. |
| 1 | Minimal fibrous thickening of alveolar or bronchiolar walls. |
| 2 | Between 1 and 3. |
| 3 | Moderate thickening of walls without obvious damage to lung architecture. |
| 4 | Between 3 and 5. |
| 5 | Increased fibrosis with definite damage to lung structure and formation of fibrous bands or small fibrous masses. |
| 6 | Between 5 and 7. |
| 7 | Severe distortion of the structure and large fibrous areas; “honeycomb lung” is placed in this category. |
| 8 | Total fibrous obliteration of the field. |
