# Supplementary material for: Danggui Buxue Decoction Ameliorates Idiopathic Pulmonary Fibrosis through MicroRNA and Messenger RNA Regulatory Network
Source: Evid Based Complement Alternat Med. 2022 Apr 26;2022:3439656. doi: 10.1155/2022/3439656 (PMC9064538; doi:10.1155/2022/3439656)
Supplement: Supplementary Materials — Table S1: DGBXD granules. Table S2: Szapiel score system. Table S3: Ashcroft score system. Table S4: predicted target genes of upregulated DE-miRNAs (n = 1285). Table S5: predicted target genes of downregulated DE-miRNAs (n = 1411). Table S6: upregulated DE-mRNAs (n = 1160). Table S7: downregulated DE-mRNAs (n = 1427). Table S8: corresponding gene symbols of RA and RAS. [file 3439656.f1.zip › 3439656.f1/Table S4 Predicted target genes of upregulated DE-miRNAs (n=1285).docx]

**Table S4:** Predicted target genes of upregulated DE-miRNAs (n=1285).

| **miRNA ID** | **Gene** |
| --- | --- |
| hsa-mir-31-5p | PARP1 |
| hsa-mir-31-5p | AP2B1 |
| hsa-mir-31-5p | AR |
| hsa-mir-31-5p | ARF1 |
| hsa-mir-31-5p | RHOA |
| hsa-mir-31-5p | ATP2A2 |
| hsa-mir-31-5p | ATP5A1 |
| hsa-mir-31-5p | CASR |
| hsa-mir-31-5p | CCNT1 |
| hsa-mir-31-5p | CDK1 |
| hsa-mir-31-5p | CYP27B1 |
| hsa-mir-31-5p | DMD |
| hsa-mir-31-5p | DOCK1 |
| hsa-mir-31-5p | E2F2 |
| hsa-mir-31-5p | EFNB1 |
| hsa-mir-31-5p | ETS1 |
| hsa-mir-31-5p | FOXC1 |
| hsa-mir-31-5p | FOXD4 |
| hsa-mir-31-5p | FOXO3 |
| hsa-mir-31-5p | FLNA |
| hsa-mir-31-5p | FRK |
| hsa-mir-31-5p | GLI2 |
| hsa-mir-31-5p | SFN |
| hsa-mir-31-5p | GTF2E1 |
| hsa-mir-31-5p | GYG1 |
| hsa-mir-31-5p | HOXA7 |
| hsa-mir-31-5p | HOXC13 |
| hsa-mir-31-5p | HOXD3 |
| hsa-mir-31-5p | ICAM1 |
| hsa-mir-31-5p | IL5RA |
| hsa-mir-31-5p | ILF2 |
| hsa-mir-31-5p | IPP |
| hsa-mir-31-5p | ITGA5 |
| hsa-mir-31-5p | JARID2 |
| hsa-mir-31-5p | STMN1 |
| hsa-mir-31-5p | SMAD4 |
| hsa-mir-31-5p | MAGEA3 |
| hsa-mir-31-5p | MAGEA6 |
| hsa-mir-31-5p | MAGEA12 |
| hsa-mir-31-5p | MCM2 |
| hsa-mir-31-5p | MET |
| hsa-mir-31-5p | MLH1 |
| hsa-mir-31-5p | AFF1 |
| hsa-mir-31-5p | MMP16 |
| hsa-mir-31-5p | MYO1D |
| hsa-mir-31-5p | NF2 |
| hsa-mir-31-5p | NFE2L1 |
| hsa-mir-31-5p | NFIC |
| hsa-mir-31-5p | NPM1 |
| hsa-mir-31-5p | PLAGL2 |
| hsa-mir-31-5p | PPP2R2A |
| hsa-mir-31-5p | PRKCE |
| hsa-mir-31-5p | PTPRJ |
| hsa-mir-31-5p | PEX19 |
| hsa-mir-31-5p | RAB5B |
| hsa-mir-31-5p | RAB27A |
| hsa-mir-31-5p | RANGAP1 |
| hsa-mir-31-5p | RASA1 |
| hsa-mir-31-5p | RDX |
| hsa-mir-31-5p | RET |
| hsa-mir-31-5p | RPA1 |
| hsa-mir-31-5p | RPL12 |
| hsa-mir-31-5p | RPL27A |
| hsa-mir-31-5p | RPL35A |
| hsa-mir-31-5p | RPL37A |
| hsa-mir-31-5p | RPS4Y1 |
| hsa-mir-31-5p | RPS7 |
| hsa-mir-31-5p | SDC4 |
| hsa-mir-31-5p | CXCL12 |
| hsa-mir-31-5p | SELE |
| hsa-mir-31-5p | SLC1A2 |
| hsa-mir-31-5p | SOX4 |
| hsa-mir-31-5p | SP1 |
| hsa-mir-31-5p | SRC |
| hsa-mir-31-5p | TAPBP |
| hsa-mir-31-5p | TBXA2R |
| hsa-mir-31-5p | TIAM1 |
| hsa-mir-31-5p | XRCC5 |
| hsa-mir-31-5p | YY1 |
| hsa-mir-31-5p | YWHAE |
| hsa-mir-31-5p | CNBP |
| hsa-mir-31-5p | FZD3 |
| hsa-mir-31-5p | ARID1A |
| hsa-mir-31-5p | TRRAP |
| hsa-mir-31-5p | BAP1 |
| hsa-mir-31-5p | FZD1 |
| hsa-mir-31-5p | HIST1H2BC |
| hsa-mir-31-5p | FAM193A |
| hsa-mir-31-5p | NUMB |
| hsa-mir-31-5p | CREG1 |
| hsa-mir-31-5p | DPM2 |
| hsa-mir-31-5p | HIST1H2BJ |
| hsa-mir-31-5p | LIPG |
| hsa-mir-31-5p | MAP4K4 |
| hsa-mir-31-5p | LAPTM4A |
| hsa-mir-31-5p | KMT2B |
| hsa-mir-31-5p | SERTAD2 |
| hsa-mir-31-5p | C2CD5 |
| hsa-mir-31-5p | ZBTB39 |
| hsa-mir-31-5p | RHOBTB1 |
| hsa-mir-31-5p | MED12 |
| hsa-mir-31-5p | ARPC5 |
| hsa-mir-31-5p | TSPAN1 |
| hsa-mir-31-5p | RASA4 |
| hsa-mir-31-5p | SPRY3 |
| hsa-mir-31-5p | SPRY1 |
| hsa-mir-31-5p | NOP56 |
| hsa-mir-31-5p | GNA13 |
| hsa-mir-31-5p | NFAT5 |
| hsa-mir-31-5p | ZNF460 |
| hsa-mir-31-5p | WASF3 |
| hsa-mir-31-5p | ZNF275 |
| hsa-mir-31-5p | SNRNP27 |
| hsa-mir-31-5p | LILRA2 |
| hsa-mir-31-5p | NUDT3 |
| hsa-mir-31-5p | PHLDA1 |
| hsa-mir-31-5p | FOXJ3 |
| hsa-mir-31-5p | BAHD1 |
| hsa-mir-31-5p | DKK1 |
| hsa-mir-31-5p | KLHDC10 |
| hsa-mir-31-5p | SMG1 |
| hsa-mir-31-5p | TNRC6B |
| hsa-mir-31-5p | MPRIP |
| hsa-mir-31-5p | XPO6 |
| hsa-mir-31-5p | SATB2 |
| hsa-mir-31-5p | ABCB9 |
| hsa-mir-31-5p | NUP188 |
| hsa-mir-31-5p | SRRM2 |
| hsa-mir-31-5p | PPIL2 |
| hsa-mir-31-5p | REXO2 |
| hsa-mir-31-5p | TOR1AIP1 |
| hsa-mir-31-5p | AGO1 |
| hsa-mir-31-5p | LATS2 |
| hsa-mir-31-5p | AKAP8L |
| hsa-mir-31-5p | GHITM |
| hsa-mir-31-5p | INTU |
| hsa-mir-31-5p | SRPX2 |
| hsa-mir-31-5p | PCSK1N |
| hsa-mir-31-5p | STOML2 |
| hsa-mir-31-5p | FOXP3 |
| hsa-mir-31-5p | RSRC1 |
| hsa-mir-31-5p | KLF13 |
| hsa-mir-31-5p | EXOC6 |
| hsa-mir-31-5p | NDFIP2 |
| hsa-mir-31-5p | OTUD4 |
| hsa-mir-31-5p | RNF111 |
| hsa-mir-31-5p | DNAAF5 |
| hsa-mir-31-5p | UBA6 |
| hsa-mir-31-5p | DDX19A |
| hsa-mir-31-5p | LRRC59 |
| hsa-mir-31-5p | ZNF331 |
| hsa-mir-31-5p | BCAS4 |
| hsa-mir-31-5p | HIF1AN |
| hsa-mir-31-5p | ECHDC1 |
| hsa-mir-31-5p | CDC42SE1 |
| hsa-mir-31-5p | DPYSL5 |
| hsa-mir-31-5p | EXOSC5 |
| hsa-mir-31-5p | EMSY |
| hsa-mir-31-5p | PARP11 |
| hsa-mir-31-5p | PPP4R3B |
| hsa-mir-31-5p | CASKIN2 |
| hsa-mir-31-5p | PHF12 |
| hsa-mir-31-5p | TRIB3 |
| hsa-mir-31-5p | GUF1 |
| hsa-mir-31-5p | ZDHHC6 |
| hsa-mir-31-5p | GIGYF1 |
| hsa-mir-31-5p | IL25 |
| hsa-mir-31-5p | PAPOLG |
| hsa-mir-31-5p | TMEM109 |
| hsa-mir-31-5p | NOL9 |
| hsa-mir-31-5p | QSER1 |
| hsa-mir-31-5p | MCMBP |
| hsa-mir-31-5p | EDC3 |
| hsa-mir-31-5p | KLHL15 |
| hsa-mir-31-5p | DNAJC5 |
| hsa-mir-31-5p | AKNA |
| hsa-mir-31-5p | TXNDC5 |
| hsa-mir-31-5p | SPRY4 |
| hsa-mir-31-5p | RAB1B |
| hsa-mir-31-5p | C19orf12 |
| hsa-mir-31-5p | SESN2 |
| hsa-mir-31-5p | STK40 |
| hsa-mir-31-5p | SPRTN |
| hsa-mir-31-5p | SYDE2 |
| hsa-mir-31-5p | PRRC2B |
| hsa-mir-31-5p | NFATC2IP |
| hsa-mir-31-5p | ZNF587 |
| hsa-mir-31-5p | HIST1H2BK |
| hsa-mir-31-5p | ZC3H12C |
| hsa-mir-31-5p | MTSS1L |
| hsa-mir-31-5p | SFXN1 |
| hsa-mir-31-5p | VPS26B |
| hsa-mir-31-5p | SLC18B1 |
| hsa-mir-31-5p | ZNF641 |
| hsa-mir-31-5p | SP7 |
| hsa-mir-31-5p | ZC3H18 |
| hsa-mir-31-5p | SGPP2 |
| hsa-mir-31-5p | TMEM182 |
| hsa-mir-31-5p | CCDC127 |
| hsa-mir-31-5p | DACT3 |
| hsa-mir-31-5p | CKAP2L |
| hsa-mir-31-5p | PPM1L |
| hsa-mir-31-5p | SPRED1 |
| hsa-mir-31-5p | RETREG3 |
| hsa-mir-31-5p | FOXD4L1 |
| hsa-mir-31-5p | SPRED2 |
| hsa-mir-31-5p | JAZF1 |
| hsa-mir-31-5p | TMEM9 |
| hsa-mir-31-5p | ANKRD52 |
| hsa-mir-31-5p | ZIK1 |
| hsa-mir-31-5p | RPL7L1 |
| hsa-mir-31-5p | C1QTNF9 |
| hsa-mir-31-5p | ZNF678 |
| hsa-mir-31-5p | FOXD4L4 |
| hsa-mir-31-5p | SFT2D2 |
| hsa-mir-31-5p | C15orf52 |
| hsa-mir-31-5p | ZNF805 |
| hsa-mir-31-5p | ACBD7 |
| hsa-mir-31-5p | MXRA7 |
| hsa-mir-31-5p | MZT1 |
| hsa-mir-31-5p | FOXD4L5 |
| hsa-mir-31-5p | C17orf99 |
| hsa-mir-31-5p | MICA |
| hsa-mir-31-5p | PYURF |
| hsa-mir-127-3p | GRK2 |
| hsa-mir-127-3p | BCL6 |
| hsa-mir-127-3p | PRDM1 |
| hsa-mir-127-3p | DAGLA |
| hsa-mir-127-3p | SEPT7 |
| hsa-mir-127-3p | SLC29A1 |
| hsa-mir-127-3p | KCNA6 |
| hsa-mir-127-3p | LGALS8 |
| hsa-mir-127-3p | MGMT |
| hsa-mir-127-3p | MMP13 |
| hsa-mir-127-3p | SERPINB9 |
| hsa-mir-127-3p | PIK3CG |
| hsa-mir-127-3p | MAPK4 |
| hsa-mir-127-3p | SFRP1 |
| hsa-mir-127-3p | SKI |
| hsa-mir-127-3p | TGM2 |
| hsa-mir-127-3p | XBP1 |
| hsa-mir-127-3p | XRCC3 |
| hsa-mir-127-3p | BAG5 |
| hsa-mir-127-3p | ZWINT |
| hsa-mir-127-3p | SEC31A |
| hsa-mir-127-3p | COTL1 |
| hsa-mir-127-3p | NBEA |
| hsa-mir-127-3p | BOLA1 |
| hsa-mir-127-3p | RGMA |
| hsa-mir-127-3p | ACTR3B |
| hsa-mir-127-3p | USP35 |
| hsa-mir-127-3p | KMT5A |
| hsa-mir-127-3p | ACTR3C |
| hsa-mir-154-5p | TMEM50B |
| hsa-mir-154-5p | CCND2 |
| hsa-mir-154-5p | E2F5 |
| hsa-mir-154-5p | ACSL1 |
| hsa-mir-154-5p | GATA6 |
| hsa-mir-154-5p | LMNB1 |
| hsa-mir-154-5p | SMAD4 |
| hsa-mir-154-5p | MYO9B |
| hsa-mir-154-5p | PPP1CC |
| hsa-mir-154-5p | PPP2R2A |
| hsa-mir-154-5p | RAB5B |
| hsa-mir-154-5p | RPS4X |
| hsa-mir-154-5p | AURKA |
| hsa-mir-154-5p | TLR2 |
| hsa-mir-154-5p | WNT5A |
| hsa-mir-154-5p | ZXDA |
| hsa-mir-154-5p | HMGA2 |
| hsa-mir-154-5p | FZD9 |
| hsa-mir-154-5p | PER3 |
| hsa-mir-154-5p | TAF1B |
| hsa-mir-154-5p | ZEB2 |
| hsa-mir-154-5p | UST |
| hsa-mir-154-5p | ZNF267 |
| hsa-mir-154-5p | ATG7 |
| hsa-mir-154-5p | MYL12A |
| hsa-mir-154-5p | CIT |
| hsa-mir-154-5p | CBX3 |
| hsa-mir-154-5p | ZFP30 |
| hsa-mir-154-5p | DICER1 |
| hsa-mir-154-5p | ACAP2 |
| hsa-mir-154-5p | VSIG2 |
| hsa-mir-154-5p | ULK3 |
| hsa-mir-154-5p | SESN1 |
| hsa-mir-154-5p | NRBF2 |
| hsa-mir-154-5p | SAR1B |
| hsa-mir-154-5p | WAC |
| hsa-mir-154-5p | GALNT7 |
| hsa-mir-154-5p | SNRK |
| hsa-mir-154-5p | ATG2B |
| hsa-mir-154-5p | SLC25A36 |
| hsa-mir-154-5p | C11orf57 |
| hsa-mir-154-5p | ASH1L |
| hsa-mir-154-5p | CLDND1 |
| hsa-mir-154-5p | OTUD7B |
| hsa-mir-154-5p | KIAA1191 |
| hsa-mir-154-5p | SLAIN2 |
| hsa-mir-154-5p | ANKEF1 |
| hsa-mir-154-5p | C8orf33 |
| hsa-mir-154-5p | PLEKHF2 |
| hsa-mir-154-5p | PCNX2 |
| hsa-mir-154-5p | SLC25A28 |
| hsa-mir-154-5p | DNAL1 |
| hsa-mir-154-5p | TMEM79 |
| hsa-mir-154-5p | LZIC |
| hsa-mir-154-5p | PCGF5 |
| hsa-mir-154-5p | FAM126A |
| hsa-mir-154-5p | ZC3HAV1L |
| hsa-mir-154-5p | ZXDB |
| hsa-mir-154-5p | TET3 |
| hsa-mir-154-5p | MMS22L |
| hsa-mir-154-5p | PLA2G4D |
| hsa-mir-154-5p | ZNF678 |
| hsa-mir-154-5p | MTRNR2L9 |
| hsa-mir-382-5p | ADM |
| hsa-mir-382-5p | BBS4 |
| hsa-mir-382-5p | ZFP36L1 |
| hsa-mir-382-5p | CALM1 |
| hsa-mir-382-5p | CASP3 |
| hsa-mir-382-5p | CCND2 |
| hsa-mir-382-5p | CLTC |
| hsa-mir-382-5p | DAD1 |
| hsa-mir-382-5p | DDOST |
| hsa-mir-382-5p | DYNC1H1 |
| hsa-mir-382-5p | DRD1 |
| hsa-mir-382-5p | EIF1AX |
| hsa-mir-382-5p | CTTN |
| hsa-mir-382-5p | FRAXA |
| hsa-mir-382-5p | STT3A |
| hsa-mir-382-5p | KIF5C |
| hsa-mir-382-5p | TM4SF1 |
| hsa-mir-382-5p | MXD1 |
| hsa-mir-382-5p | ATXN3 |
| hsa-mir-382-5p | NFIA |
| hsa-mir-382-5p | NHS |
| hsa-mir-382-5p | YBX1 |
| hsa-mir-382-5p | PCBP1 |
| hsa-mir-382-5p | PFKFB2 |
| hsa-mir-382-5p | PPM1A |
| hsa-mir-382-5p | PRNP |
| hsa-mir-382-5p | PTEN |
| hsa-mir-382-5p | PTGDR |
| hsa-mir-382-5p | RAB6A |
| hsa-mir-382-5p | RPLP0 |
| hsa-mir-382-5p | ATXN1 |
| hsa-mir-382-5p | SLC3A1 |
| hsa-mir-382-5p | SLC6A8 |
| hsa-mir-382-5p | TSPYL1 |
| hsa-mir-382-5p | UBB |
| hsa-mir-382-5p | XPO1 |
| hsa-mir-382-5p | VEZF1 |
| hsa-mir-382-5p | PTP4A2 |
| hsa-mir-382-5p | HIST1H3G |
| hsa-mir-382-5p | DYRK2 |
| hsa-mir-382-5p | SAP30 |
| hsa-mir-382-5p | SYNJ2 |
| hsa-mir-382-5p | HERC2 |
| hsa-mir-382-5p | HIST1H3F |
| hsa-mir-382-5p | SMC3 |
| hsa-mir-382-5p | XPR1 |
| hsa-mir-382-5p | VAMP3 |
| hsa-mir-382-5p | ZNF264 |
| hsa-mir-382-5p | RBM39 |
| hsa-mir-382-5p | G3BP2 |
| hsa-mir-382-5p | ARL4A |
| hsa-mir-382-5p | YAF2 |
| hsa-mir-382-5p | PNMA2 |
| hsa-mir-382-5p | MTHFD2 |
| hsa-mir-382-5p | GPR176 |
| hsa-mir-382-5p | B4GALT7 |
| hsa-mir-382-5p | ZNF652 |
| hsa-mir-382-5p | KLHDC10 |
| hsa-mir-382-5p | ZCCHC14 |
| hsa-mir-382-5p | MTCL1 |
| hsa-mir-382-5p | UFL1 |
| hsa-mir-382-5p | EXOSC2 |
| hsa-mir-382-5p | DICER1 |
| hsa-mir-382-5p | SF3B1 |
| hsa-mir-382-5p | ZKSCAN5 |
| hsa-mir-382-5p | UBBP4 |
| hsa-mir-382-5p | ZNF318 |
| hsa-mir-382-5p | PARM1 |
| hsa-mir-382-5p | KLHL3 |
| hsa-mir-382-5p | SERGEF |
| hsa-mir-382-5p | MRPS18C |
| hsa-mir-382-5p | SAR1B |
| hsa-mir-382-5p | COPS4 |
| hsa-mir-382-5p | TRPV2 |
| hsa-mir-382-5p | GSKIP |
| hsa-mir-382-5p | ARMCX3 |
| hsa-mir-382-5p | FAM105A |
| hsa-mir-382-5p | GNL3L |
| hsa-mir-382-5p | PNPO |
| hsa-mir-382-5p | FAM46A |
| hsa-mir-382-5p | PLSCR4 |
| hsa-mir-382-5p | SYT13 |
| hsa-mir-382-5p | NYAP2 |
| hsa-mir-382-5p | SMURF2 |
| hsa-mir-382-5p | RAPH1 |
| hsa-mir-382-5p | TBL1XR1 |
| hsa-mir-382-5p | RPAP2 |
| hsa-mir-382-5p | DSN1 |
| hsa-mir-382-5p | PCNX2 |
| hsa-mir-382-5p | NETO2 |
| hsa-mir-382-5p | ATG10 |
| hsa-mir-382-5p | SLC10A7 |
| hsa-mir-382-5p | PLEKHA8 |
| hsa-mir-382-5p | ADO |
| hsa-mir-382-5p | TMEM209 |
| hsa-mir-382-5p | SLC25A46 |
| hsa-mir-382-5p | MSANTD3 |
| hsa-mir-382-5p | FNIP1 |
| hsa-mir-382-5p | SCAMP4 |
| hsa-mir-382-5p | SPIC |
| hsa-mir-382-5p | MTPN |
| hsa-mir-382-5p | FAM199X |
| hsa-mir-382-5p | APCDD1 |
| hsa-mir-382-5p | NEXMIF |
| hsa-mir-382-5p | ZNF860 |
| hsa-mir-382-5p | DGAT2L6 |
| hsa-mir-382-5p | NHLRC3 |
| hsa-mir-382-5p | LUZP6 |
| hsa-mir-382-5p | MTRNR2L1 |
| hsa-mir-382-5p | MTRNR2L2 |
| hsa-mir-382-5p | MTRNR2L8 |
| hsa-mir-369-5p | DNMT3A |
| hsa-mir-369-5p | DNMT3B |
| hsa-mir-369-5p | FABP4 |
| hsa-mir-369-5p | HLA-DQB2 |
| hsa-mir-369-5p | PAFAH1B2 |
| hsa-mir-369-5p | TGFBR3 |
| hsa-mir-369-5p | NR4A3 |
| hsa-mir-369-5p | RBM39 |
| hsa-mir-369-5p | ZWINT |
| hsa-mir-369-5p | ANKRD42 |
| hsa-mir-369-5p | HIST2H3D |
| hsa-mir-409-5p | ABCA3 |
| hsa-mir-409-5p | AIRE |
| hsa-mir-409-5p | DYNC1H1 |
| hsa-mir-409-5p | EIF4EBP2 |
| hsa-mir-409-5p | FDXR |
| hsa-mir-409-5p | GNAI1 |
| hsa-mir-409-5p | GSK3B |
| hsa-mir-409-5p | KPNA3 |
| hsa-mir-409-5p | MC2R |
| hsa-mir-409-5p | RGL2 |
| hsa-mir-409-5p | RPS4X |
| hsa-mir-409-5p | RSU1 |
| hsa-mir-409-5p | SOD2 |
| hsa-mir-409-5p | VLDLR |
| hsa-mir-409-5p | ZNF12 |
| hsa-mir-409-5p | AKAP1 |
| hsa-mir-409-5p | PDXK |
| hsa-mir-409-5p | STAG2 |
| hsa-mir-409-5p | CCT5 |
| hsa-mir-409-5p | RPRD2 |
| hsa-mir-409-5p | ACAP2 |
| hsa-mir-409-5p | NBEA |
| hsa-mir-409-5p | ZDHHC9 |
| hsa-mir-409-5p | RAB14 |
| hsa-mir-409-5p | RUFY2 |
| hsa-mir-409-5p | ZNF512B |
| hsa-mir-409-5p | GPBP1L1 |
| hsa-mir-409-5p | QSER1 |
| hsa-mir-409-5p | FAM163A |
| hsa-mir-409-5p | SRRD |
| hsa-mir-409-5p | ZBTB34 |
| hsa-mir-409-3p | AKT1 |
| hsa-mir-409-3p | ANG |
| hsa-mir-409-3p | ATP5G3 |
| hsa-mir-409-3p | FOXN3 |
| hsa-mir-409-3p | CTNND1 |
| hsa-mir-409-3p | ELF2 |
| hsa-mir-409-3p | FGA |
| hsa-mir-409-3p | FGB |
| hsa-mir-409-3p | FGG |
| hsa-mir-409-3p | GAB1 |
| hsa-mir-409-3p | GCK |
| hsa-mir-409-3p | GDNF |
| hsa-mir-409-3p | GNAL |
| hsa-mir-409-3p | GSK3B |
| hsa-mir-409-3p | MSH6 |
| hsa-mir-409-3p | IFNG |
| hsa-mir-409-3p | KPNA4 |
| hsa-mir-409-3p | MET |
| hsa-mir-409-3p | MGMT |
| hsa-mir-409-3p | ATXN3 |
| hsa-mir-409-3p | PDE7A |
| hsa-mir-409-3p | PPP2R2A |
| hsa-mir-409-3p | PPP3R1 |
| hsa-mir-409-3p | PRNP |
| hsa-mir-409-3p | RDX |
| hsa-mir-409-3p | RECQL |
| hsa-mir-409-3p | RNASEL |
| hsa-mir-409-3p | RPS24 |
| hsa-mir-409-3p | RSU1 |
| hsa-mir-409-3p | SGCD |
| hsa-mir-409-3p | SRP54 |
| hsa-mir-409-3p | HSPA13 |
| hsa-mir-409-3p | ZEB1 |
| hsa-mir-409-3p | NR2F2 |
| hsa-mir-409-3p | TGFBR2 |
| hsa-mir-409-3p | SEC62 |
| hsa-mir-409-3p | TMPO |
| hsa-mir-409-3p | TXNRD1 |
| hsa-mir-409-3p | UBE2D1 |
| hsa-mir-409-3p | UGT2B17 |
| hsa-mir-409-3p | UQCRFS1 |
| hsa-mir-409-3p | YWHAE |
| hsa-mir-409-3p | ZFX |
| hsa-mir-409-3p | ZNF207 |
| hsa-mir-409-3p | ZNF224 |
| hsa-mir-409-3p | CSDE1 |
| hsa-mir-409-3p | RECK |
| hsa-mir-409-3p | DCAF5 |
| hsa-mir-409-3p | MTMR7 |
| hsa-mir-409-3p | VGLL4 |
| hsa-mir-409-3p | SECISBP2L |
| hsa-mir-409-3p | FRAT1 |
| hsa-mir-409-3p | HNRNPR |
| hsa-mir-409-3p | PRPF8 |
| hsa-mir-409-3p | DCTN6 |
| hsa-mir-409-3p | STAG2 |
| hsa-mir-409-3p | MALT1 |
| hsa-mir-409-3p | DIDO1 |
| hsa-mir-409-3p | MGAT4A |
| hsa-mir-409-3p | MTF2 |
| hsa-mir-409-3p | ELL2 |
| hsa-mir-409-3p | EFR3B |
| hsa-mir-409-3p | UNC13A |
| hsa-mir-409-3p | KDM4C |
| hsa-mir-409-3p | RASGRP3 |
| hsa-mir-409-3p | MOB4 |
| hsa-mir-409-3p | ZBTB20 |
| hsa-mir-409-3p | TNRC6A |
| hsa-mir-409-3p | RNF141 |
| hsa-mir-409-3p | PPHLN1 |
| hsa-mir-409-3p | NLK |
| hsa-mir-409-3p | MYO3A |
| hsa-mir-409-3p | TRMT13 |
| hsa-mir-409-3p | MINDY2 |
| hsa-mir-409-3p | MSL2 |
| hsa-mir-409-3p | PHF10 |
| hsa-mir-409-3p | DDX19A |
| hsa-mir-409-3p | C20orf24 |
| hsa-mir-409-3p | C5orf15 |
| hsa-mir-409-3p | NUFIP2 |
| hsa-mir-409-3p | SLAIN2 |
| hsa-mir-409-3p | ZFP14 |
| hsa-mir-409-3p | G6PC2 |
| hsa-mir-409-3p | AASDHPPT |
| hsa-mir-409-3p | ZMAT3 |
| hsa-mir-409-3p | CDCP1 |
| hsa-mir-409-3p | GRAMD2B |
| hsa-mir-409-3p | ZFHX4 |
| hsa-mir-409-3p | MOB3B |
| hsa-mir-409-3p | ATAD5 |
| hsa-mir-409-3p | CPEB4 |
| hsa-mir-409-3p | GUCD1 |
| hsa-mir-409-3p | MED10 |
| hsa-mir-409-3p | XKR4 |
| hsa-mir-409-3p | C1QTNF3 |
| hsa-mir-409-3p | SLC26A7 |
| hsa-mir-409-3p | FCHO2 |
| hsa-mir-409-3p | CCDC38 |
| hsa-mir-409-3p | MPLKIP |
| hsa-mir-409-3p | SESN3 |
| hsa-mir-409-3p | CCDC117 |
| hsa-mir-409-3p | CCDC80 |
| hsa-mir-409-3p | RNF38 |
| hsa-mir-409-3p | CAMSAP1 |
| hsa-mir-409-3p | ZNF367 |
| hsa-mir-409-3p | FLCN |
| hsa-mir-409-3p | PDE12 |
| hsa-mir-409-3p | LVRN |
| hsa-mir-409-3p | C17orf105 |
| hsa-mir-409-3p | HCAR2 |
| hsa-mir-409-3p | HSPE1-MOB4 |
| hsa-mir-410-3p | ABCF1 |
| hsa-mir-410-3p | ADCY9 |
| hsa-mir-410-3p | ADD2 |
| hsa-mir-410-3p | JAG1 |
| hsa-mir-410-3p | AGTR1 |
| hsa-mir-410-3p | ANXA5 |
| hsa-mir-410-3p | CA8 |
| hsa-mir-410-3p | CCNB1 |
| hsa-mir-410-3p | CDK1 |
| hsa-mir-410-3p | CHEK1 |
| hsa-mir-410-3p | CHML |
| hsa-mir-410-3p | KLF6 |
| hsa-mir-410-3p | CRK |
| hsa-mir-410-3p | CD55 |
| hsa-mir-410-3p | DGKG |
| hsa-mir-410-3p | DDX3X |
| hsa-mir-410-3p | DUSP7 |
| hsa-mir-410-3p | DUSP8 |
| hsa-mir-410-3p | EIF2B1 |
| hsa-mir-410-3p | ELF2 |
| hsa-mir-410-3p | ETS2 |
| hsa-mir-410-3p | ETV6 |
| hsa-mir-410-3p | F2RL1 |
| hsa-mir-410-3p | ACSL4 |
| hsa-mir-410-3p | FKBP1A |
| hsa-mir-410-3p | FOXL1 |
| hsa-mir-410-3p | GDNF |
| hsa-mir-410-3p | GRID1 |
| hsa-mir-410-3p | GSK3B |
| hsa-mir-410-3p | GTF2A1 |
| hsa-mir-410-3p | GTF2B |
| hsa-mir-410-3p | HIVEP1 |
| hsa-mir-410-3p | HNRNPU |
| hsa-mir-410-3p | HOXA11 |
| hsa-mir-410-3p | HES1 |
| hsa-mir-410-3p | NDST1 |
| hsa-mir-410-3p | IFNAR1 |
| hsa-mir-410-3p | IGFBP1 |
| hsa-mir-410-3p | ITPKB |
| hsa-mir-410-3p | JAG2 |
| hsa-mir-410-3p | TNPO1 |
| hsa-mir-410-3p | LBR |
| hsa-mir-410-3p | LDHA |
| hsa-mir-410-3p | LDLR |
| hsa-mir-410-3p | LRP6 |
| hsa-mir-410-3p | MCAM |
| hsa-mir-410-3p | DNAJB9 |
| hsa-mir-410-3p | MDM2 |
| hsa-mir-410-3p | MET |
| hsa-mir-410-3p | MLLT3 |
| hsa-mir-410-3p | MYBL1 |
| hsa-mir-410-3p | MYBPC1 |
| hsa-mir-410-3p | NOTCH1 |
| hsa-mir-410-3p | NTRK3 |
| hsa-mir-410-3p | OCRL |
| hsa-mir-410-3p | PIK3CG |
| hsa-mir-410-3p | POU2F1 |
| hsa-mir-410-3p | POU2F2 |
| hsa-mir-410-3p | PPP2R5E |
| hsa-mir-410-3p | PRKCD |
| hsa-mir-410-3p | RAB3B |
| hsa-mir-410-3p | RANBP1 |
| hsa-mir-410-3p | REST |
| hsa-mir-410-3p | RREB1 |
| hsa-mir-410-3p | SC5D |
| hsa-mir-410-3p | CXCL5 |
| hsa-mir-410-3p | SLC7A2 |
| hsa-mir-410-3p | SLC8A1 |
| hsa-mir-410-3p | SNAI1 |
| hsa-mir-410-3p | SNRPD3 |
| hsa-mir-410-3p | SNX2 |
| hsa-mir-410-3p | SOD2 |
| hsa-mir-410-3p | SP1 |
| hsa-mir-410-3p | SRP72 |
| hsa-mir-410-3p | HSPA13 |
| hsa-mir-410-3p | SYP |
| hsa-mir-410-3p | TFDP1 |
| hsa-mir-410-3p | TGIF1 |
| hsa-mir-410-3p | THBS1 |
| hsa-mir-410-3p | TPD52 |
| hsa-mir-410-3p | TRAF6 |
| hsa-mir-410-3p | VEGFA |
| hsa-mir-410-3p | XRCC3 |
| hsa-mir-410-3p | YWHAZ |
| hsa-mir-410-3p | MAP3K12 |
| hsa-mir-410-3p | ZXDA |
| hsa-mir-410-3p | CSDE1 |
| hsa-mir-410-3p | FZD5 |
| hsa-mir-410-3p | PDHX |
| hsa-mir-410-3p | MFAP5 |
| hsa-mir-410-3p | NRIP1 |
| hsa-mir-410-3p | HIST1H3B |
| hsa-mir-410-3p | STX7 |
| hsa-mir-410-3p | RECK |
| hsa-mir-410-3p | CUL2 |
| hsa-mir-410-3p | KHSRP |
| hsa-mir-410-3p | KLF7 |
| hsa-mir-410-3p | TNFSF9 |
| hsa-mir-410-3p | TRIP10 |
| hsa-mir-410-3p | KIF3B |
| hsa-mir-410-3p | ARHGAP29 |
| hsa-mir-410-3p | SLC25A27 |
| hsa-mir-410-3p | ATP6V1G1 |
| hsa-mir-410-3p | ZNF516 |
| hsa-mir-410-3p | USP6NL |
| hsa-mir-410-3p | LAPTM4A |
| hsa-mir-410-3p | PHACTR2 |
| hsa-mir-410-3p | HUWE1 |
| hsa-mir-410-3p | MBNL2 |
| hsa-mir-410-3p | RBM7 |
| hsa-mir-410-3p | ZNF267 |
| hsa-mir-410-3p | ZER1 |
| hsa-mir-410-3p | NUP50 |
| hsa-mir-410-3p | ZNF275 |
| hsa-mir-410-3p | TCERG1 |
| hsa-mir-410-3p | COPS8 |
| hsa-mir-410-3p | HNRNPA0 |
| hsa-mir-410-3p | BTG3 |
| hsa-mir-410-3p | DDX52 |
| hsa-mir-410-3p | TPPP |
| hsa-mir-410-3p | DUSP10 |
| hsa-mir-410-3p | XPOT |
| hsa-mir-410-3p | ZFP30 |
| hsa-mir-410-3p | ZNF507 |
| hsa-mir-410-3p | PLEKHA6 |
| hsa-mir-410-3p | ZHX3 |
| hsa-mir-410-3p | ZNF609 |
| hsa-mir-410-3p | PRRC2C |
| hsa-mir-410-3p | TRIM2 |
| hsa-mir-410-3p | LARP1 |
| hsa-mir-410-3p | SIK3 |
| hsa-mir-410-3p | HEY1 |
| hsa-mir-410-3p | SLC7A11 |
| hsa-mir-410-3p | PRKD3 |
| hsa-mir-410-3p | OSBP2 |
| hsa-mir-410-3p | ZNF318 |
| hsa-mir-410-3p | SLC24A2 |
| hsa-mir-410-3p | SLC39A6 |
| hsa-mir-410-3p | MGAT4C |
| hsa-mir-410-3p | POT1 |
| hsa-mir-410-3p | NSL1 |
| hsa-mir-410-3p | ZBTB20 |
| hsa-mir-410-3p | SENP3 |
| hsa-mir-410-3p | FBXL3 |
| hsa-mir-410-3p | RGS17 |
| hsa-mir-410-3p | SNX5 |
| hsa-mir-410-3p | SULT1B1 |
| hsa-mir-410-3p | TNRC18P2 |
| hsa-mir-410-3p | OSTM1 |
| hsa-mir-410-3p | CLEC2D |
| hsa-mir-410-3p | USP25 |
| hsa-mir-410-3p | C6orf48 |
| hsa-mir-410-3p | GLRX2 |
| hsa-mir-410-3p | APIP |
| hsa-mir-410-3p | CERCAM |
| hsa-mir-410-3p | HSPA14 |
| hsa-mir-410-3p | ZBTB7A |
| hsa-mir-410-3p | TRIAP1 |
| hsa-mir-410-3p | TRMT112 |
| hsa-mir-410-3p | RSF1 |
| hsa-mir-410-3p | SIX4 |
| hsa-mir-410-3p | XRN1 |
| hsa-mir-410-3p | TMCO1 |
| hsa-mir-410-3p | RSBN1 |
| hsa-mir-410-3p | ZNHIT6 |
| hsa-mir-410-3p | LEPROT |
| hsa-mir-410-3p | ZRANB1 |
| hsa-mir-410-3p | BNC2 |
| hsa-mir-410-3p | BCOR |
| hsa-mir-410-3p | ACER3 |
| hsa-mir-410-3p | KLHL9 |
| hsa-mir-410-3p | AJAP1 |
| hsa-mir-410-3p | EMC7 |
| hsa-mir-410-3p | TM9SF3 |
| hsa-mir-410-3p | PMEPA1 |
| hsa-mir-410-3p | STOX2 |
| hsa-mir-410-3p | CABP4 |
| hsa-mir-410-3p | CNOT6 |
| hsa-mir-410-3p | ZNF608 |
| hsa-mir-410-3p | NUFIP2 |
| hsa-mir-410-3p | ZNF319 |
| hsa-mir-410-3p | DDX55 |
| hsa-mir-410-3p | MIER1 |
| hsa-mir-410-3p | CYP4F11 |
| hsa-mir-410-3p | CREBZF |
| hsa-mir-410-3p | BACH2 |
| hsa-mir-410-3p | PAPD5 |
| hsa-mir-410-3p | HHIP |
| hsa-mir-410-3p | DCLRE1C |
| hsa-mir-410-3p | MEAF6 |
| hsa-mir-410-3p | NABP1 |
| hsa-mir-410-3p | PLEKHA3 |
| hsa-mir-410-3p | C1orf50 |
| hsa-mir-410-3p | ARSJ |
| hsa-mir-410-3p | ZFAND1 |
| hsa-mir-410-3p | ZNF556 |
| hsa-mir-410-3p | ABHD18 |
| hsa-mir-410-3p | RAB11FIP1 |
| hsa-mir-410-3p | KLHL15 |
| hsa-mir-410-3p | TTYH3 |
| hsa-mir-410-3p | APOL6 |
| hsa-mir-410-3p | KIAA1109 |
| hsa-mir-410-3p | POLR1B |
| hsa-mir-410-3p | PHF6 |
| hsa-mir-410-3p | ZBED3 |
| hsa-mir-410-3p | HPS3 |
| hsa-mir-410-3p | PPP1R15B |
| hsa-mir-410-3p | SCIN |
| hsa-mir-410-3p | TRIM4 |
| hsa-mir-410-3p | ZNF551 |
| hsa-mir-410-3p | MCFD2 |
| hsa-mir-410-3p | HAUS8 |
| hsa-mir-410-3p | MMGT1 |
| hsa-mir-410-3p | LYSMD3 |
| hsa-mir-410-3p | GINM1 |
| hsa-mir-410-3p | CCDC38 |
| hsa-mir-410-3p | MSI2 |
| hsa-mir-410-3p | UHMK1 |
| hsa-mir-410-3p | SGPP2 |
| hsa-mir-410-3p | CPEB2 |
| hsa-mir-410-3p | GRPEL2 |
| hsa-mir-410-3p | AMER1 |
| hsa-mir-410-3p | TRPM6 |
| hsa-mir-410-3p | C18orf25 |
| hsa-mir-410-3p | RC3H1 |
| hsa-mir-410-3p | EXOC8 |
| hsa-mir-410-3p | CKAP2L |
| hsa-mir-410-3p | ITPRIPL1 |
| hsa-mir-410-3p | KLHL23 |
| hsa-mir-410-3p | CCDC80 |
| hsa-mir-410-3p | ITPRIPL2 |
| hsa-mir-410-3p | AGO3 |
| hsa-mir-410-3p | FAM76A |
| hsa-mir-410-3p | MPEG1 |
| hsa-mir-410-3p | DOK6 |
| hsa-mir-410-3p | LRRC63 |
| hsa-mir-410-3p | VGLL2 |
| hsa-mir-410-3p | PRR14L |
| hsa-mir-410-3p | CADM2 |
| hsa-mir-410-3p | GPATCH11 |
| hsa-mir-410-3p | SVIP |
| hsa-mir-410-3p | SLC46A3 |
| hsa-mir-410-3p | EOGT |
| hsa-mir-410-3p | RGMB |
| hsa-mir-410-3p | ZNF850 |
| hsa-mir-410-3p | GPR141 |
| hsa-mir-410-3p | RPSAP58 |
| hsa-mir-410-3p | LIN28B |
| hsa-mir-410-3p | ANKRD33B |
| hsa-mir-410-3p | C16orf52 |
| hsa-mir-493-5p | ACTB |
| hsa-mir-493-5p | RHOC |
| hsa-mir-493-5p | ARHGAP5 |
| hsa-mir-493-5p | ATP7A |
| hsa-mir-493-5p | BDH1 |
| hsa-mir-493-5p | CALM2 |
| hsa-mir-493-5p | CDKN1A |
| hsa-mir-493-5p | CKS2 |
| hsa-mir-493-5p | CTGF |
| hsa-mir-493-5p | EIF2B1 |
| hsa-mir-493-5p | FHL2 |
| hsa-mir-493-5p | FOXL1 |
| hsa-mir-493-5p | FOS |
| hsa-mir-493-5p | FUT4 |
| hsa-mir-493-5p | GNAT1 |
| hsa-mir-493-5p | GRIK3 |
| hsa-mir-493-5p | GTF2H1 |
| hsa-mir-493-5p | IL12RB2 |
| hsa-mir-493-5p | JAG2 |
| hsa-mir-493-5p | KCNJ6 |
| hsa-mir-493-5p | L1CAM |
| hsa-mir-493-5p | DNAJB9 |
| hsa-mir-493-5p | MT2A |
| hsa-mir-493-5p | MYO10 |
| hsa-mir-493-5p | NCL |
| hsa-mir-493-5p | NDUFS1 |
| hsa-mir-493-5p | OLR1 |
| hsa-mir-493-5p | PCSK1 |
| hsa-mir-493-5p | PDPK1 |
| hsa-mir-493-5p | CDK14 |
| hsa-mir-493-5p | PTPRF |
| hsa-mir-493-5p | RAB5C |
| hsa-mir-493-5p | RAD23B |
| hsa-mir-493-5p | RPS3 |
| hsa-mir-493-5p | SCD |
| hsa-mir-493-5p | TRA2B |
| hsa-mir-493-5p | SP4 |
| hsa-mir-493-5p | SYT4 |
| hsa-mir-493-5p | TDG |
| hsa-mir-493-5p | UBE2G1 |
| hsa-mir-493-5p | PCGF2 |
| hsa-mir-493-5p | VEZF1 |
| hsa-mir-493-5p | EVI5 |
| hsa-mir-493-5p | HMGA2 |
| hsa-mir-493-5p | SMARCA5 |
| hsa-mir-493-5p | HAT1 |
| hsa-mir-493-5p | SLC25A12 |
| hsa-mir-493-5p | HIP1R |
| hsa-mir-493-5p | MAP7 |
| hsa-mir-493-5p | USP2 |
| hsa-mir-493-5p | SMC3 |
| hsa-mir-493-5p | TBRG4 |
| hsa-mir-493-5p | HMGN3 |
| hsa-mir-493-5p | SOCS5 |
| hsa-mir-493-5p | JADE3 |
| hsa-mir-493-5p | TBC1D5 |
| hsa-mir-493-5p | TSC22D2 |
| hsa-mir-493-5p | HMGXB4 |
| hsa-mir-493-5p | SCAMP2 |
| hsa-mir-493-5p | LHFPL6 |
| hsa-mir-493-5p | TRIM13 |
| hsa-mir-493-5p | PCGF3 |
| hsa-mir-493-5p | CITED2 |
| hsa-mir-493-5p | ANP32B |
| hsa-mir-493-5p | SRSF10 |
| hsa-mir-493-5p | WDR3 |
| hsa-mir-493-5p | NUDT3 |
| hsa-mir-493-5p | RPH3A |
| hsa-mir-493-5p | RAB21 |
| hsa-mir-493-5p | PRRC2C |
| hsa-mir-493-5p | AGTPBP1 |
| hsa-mir-493-5p | TRIM2 |
| hsa-mir-493-5p | SLC44A1 |
| hsa-mir-493-5p | SF3B1 |
| hsa-mir-493-5p | CES3 |
| hsa-mir-493-5p | CHMP2B |
| hsa-mir-493-5p | LSM14A |
| hsa-mir-493-5p | RTL8A |
| hsa-mir-493-5p | FBXL3 |
| hsa-mir-493-5p | SULT1B1 |
| hsa-mir-493-5p | GOLM1 |
| hsa-mir-493-5p | WAC |
| hsa-mir-493-5p | ZNF107 |
| hsa-mir-493-5p | SARAF |
| hsa-mir-493-5p | RSF1 |
| hsa-mir-493-5p | MRPS21 |
| hsa-mir-493-5p | RBM23 |
| hsa-mir-493-5p | DEPDC1 |
| hsa-mir-493-5p | MBNL3 |
| hsa-mir-493-5p | ZMIZ1 |
| hsa-mir-493-5p | USP28 |
| hsa-mir-493-5p | ZDBF2 |
| hsa-mir-493-5p | DDX55 |
| hsa-mir-493-5p | C12orf49 |
| hsa-mir-493-5p | GEMIN6 |
| hsa-mir-493-5p | STN1 |
| hsa-mir-493-5p | KLHL15 |
| hsa-mir-493-5p | CFHR5 |
| hsa-mir-493-5p | LMNB2 |
| hsa-mir-493-5p | USMG5 |
| hsa-mir-493-5p | FNIP1 |
| hsa-mir-493-5p | EFHC1 |
| hsa-mir-493-5p | PWWP2A |
| hsa-mir-493-5p | TOP1MT |
| hsa-mir-493-5p | RAB3IP |
| hsa-mir-493-5p | ZDHHC15 |
| hsa-mir-493-5p | ZNF550 |
| hsa-mir-493-5p | EPHX4 |
| hsa-mir-493-5p | ANKRD52 |
| hsa-mir-493-5p | TMPRSS12 |
| hsa-mir-493-5p | SPOPL |
| hsa-mir-493-5p | FREM2 |
| hsa-mir-493-5p | GPR141 |
| hsa-mir-493-5p | C16orf52 |
| hsa-mir-493-5p | MTRNR2L2 |
| hsa-mir-432-5p | ADAR |
| hsa-mir-432-5p | ATM |
| hsa-mir-432-5p | BBS4 |
| hsa-mir-432-5p | DST |
| hsa-mir-432-5p | CENPA |
| hsa-mir-432-5p | CPD |
| hsa-mir-432-5p | CSNK1G2 |
| hsa-mir-432-5p | MEGF9 |
| hsa-mir-432-5p | GALNT2 |
| hsa-mir-432-5p | GNA12 |
| hsa-mir-432-5p | GNAS |
| hsa-mir-432-5p | IAPP |
| hsa-mir-432-5p | ID4 |
| hsa-mir-432-5p | INS |
| hsa-mir-432-5p | KCNMB1 |
| hsa-mir-432-5p | LIF |
| hsa-mir-432-5p | MDM2 |
| hsa-mir-432-5p | MECP2 |
| hsa-mir-432-5p | MGAT1 |
| hsa-mir-432-5p | NFIC |
| hsa-mir-432-5p | NUCB2 |
| hsa-mir-432-5p | PRKAB1 |
| hsa-mir-432-5p | PRKCH |
| hsa-mir-432-5p | PTPN3 |
| hsa-mir-432-5p | TRAPPC2 |
| hsa-mir-432-5p | SPARC |
| hsa-mir-432-5p | SURF4 |
| hsa-mir-432-5p | TFPI |
| hsa-mir-432-5p | TUBB2A |
| hsa-mir-432-5p | CSDE1 |
| hsa-mir-432-5p | DHX16 |
| hsa-mir-432-5p | OGT |
| hsa-mir-432-5p | OR6A2 |
| hsa-mir-432-5p | DLG5 |
| hsa-mir-432-5p | PITPNM1 |
| hsa-mir-432-5p | PDE4DIP |
| hsa-mir-432-5p | ESPL1 |
| hsa-mir-432-5p | DNAJB6 |
| hsa-mir-432-5p | LRPPRC |
| hsa-mir-432-5p | STAMBP |
| hsa-mir-432-5p | NES |
| hsa-mir-432-5p | BLCAP |
| hsa-mir-432-5p | AKAP2 |
| hsa-mir-432-5p | RHOBTB3 |
| hsa-mir-432-5p | UNC13A |
| hsa-mir-432-5p | SETX |
| hsa-mir-432-5p | KDM4C |
| hsa-mir-432-5p | ZCCHC14 |
| hsa-mir-432-5p | RCOR1 |
| hsa-mir-432-5p | RBFOX2 |
| hsa-mir-432-5p | DSTYK |
| hsa-mir-432-5p | HEATR5A |
| hsa-mir-432-5p | PNISR |
| hsa-mir-432-5p | SETBP1 |
| hsa-mir-432-5p | ZBTB20 |
| hsa-mir-432-5p | PCLO |
| hsa-mir-432-5p | DNTTIP2 |
| hsa-mir-432-5p | SOCS7 |
| hsa-mir-432-5p | ASAP1 |
| hsa-mir-432-5p | NBAS |
| hsa-mir-432-5p | CHMP3 |
| hsa-mir-432-5p | SOBP |
| hsa-mir-432-5p | LRRC1 |
| hsa-mir-432-5p | KIF21A |
| hsa-mir-432-5p | ERO1B |
| hsa-mir-432-5p | CASKIN1 |
| hsa-mir-432-5p | P3H1 |
| hsa-mir-432-5p | MRPL34 |
| hsa-mir-432-5p | RAPH1 |
| hsa-mir-432-5p | KIAA0319L |
| hsa-mir-432-5p | OR2C3 |
| hsa-mir-432-5p | KCNK16 |
| hsa-mir-432-5p | ARID5B |
| hsa-mir-432-5p | ING5 |
| hsa-mir-432-5p | PHLDB2 |
| hsa-mir-432-5p | LYRM7 |
| hsa-mir-432-5p | IGSF8 |
| hsa-mir-432-5p | MOGAT1 |
| hsa-mir-432-5p | ZNF784 |
| hsa-mir-432-5p | ITPRIPL1 |
| hsa-mir-432-5p | ADAMTS17 |
| hsa-mir-432-5p | DENND6A |
| hsa-mir-432-5p | TUBB |
| hsa-mir-432-5p | ANO5 |
| hsa-mir-432-5p | UNC5B |
| hsa-mir-432-5p | SEC14L4 |
| hsa-mir-432-5p | FMN1 |
| hsa-mir-432-5p | IRF2BP2 |
| hsa-mir-432-5p | C10orf105 |
| hsa-mir-432-5p | PALM2-AKAP2 |
| hsa-mir-432-5p | SPANXN1 |
| hsa-mir-432-5p | SPANXN5 |
| hsa-mir-432-5p | GDF5OS |
| hsa-mir-432-5p | RNF103-CHMP3 |
| hsa-mir-495-3p | ACTA1 |
| hsa-mir-495-3p | ACTC1 |
| hsa-mir-495-3p | AKT1 |
| hsa-mir-495-3p | ATP7A |
| hsa-mir-495-3p | BMI1 |
| hsa-mir-495-3p | CASP8 |
| hsa-mir-495-3p | RUNX3 |
| hsa-mir-495-3p | CD9 |
| hsa-mir-495-3p | CDK1 |
| hsa-mir-495-3p | CDC5L |
| hsa-mir-495-3p | COL4A1 |
| hsa-mir-495-3p | DGKB |
| hsa-mir-495-3p | FGF2 |
| hsa-mir-495-3p | FOXC1 |
| hsa-mir-495-3p | B4GALT1 |
| hsa-mir-495-3p | HMGN1 |
| hsa-mir-495-3p | HNRNPC |
| hsa-mir-495-3p | HOXC8 |
| hsa-mir-495-3p | HOXD12 |
| hsa-mir-495-3p | HSPA1B |
| hsa-mir-495-3p | HSPA5 |
| hsa-mir-495-3p | HSP90AA1 |
| hsa-mir-495-3p | IL6R |
| hsa-mir-495-3p | IL15 |
| hsa-mir-495-3p | INHBA |
| hsa-mir-495-3p | KPNA2 |
| hsa-mir-495-3p | TNPO1 |
| hsa-mir-495-3p | LDLR |
| hsa-mir-495-3p | LMAN1 |
| hsa-mir-495-3p | LRP6 |
| hsa-mir-495-3p | MARCKS |
| hsa-mir-495-3p | MAK |
| hsa-mir-495-3p | MAT1A |
| hsa-mir-495-3p | MCL1 |
| hsa-mir-495-3p | MEIS1 |
| hsa-mir-495-3p | MFAP3 |
| hsa-mir-495-3p | MOCS2 |
| hsa-mir-495-3p | MTAP |
| hsa-mir-495-3p | TRIM37 |
| hsa-mir-495-3p | MYO10 |
| hsa-mir-495-3p | NCAM2 |
| hsa-mir-495-3p | NDUFB6 |
| hsa-mir-495-3p | OCRL |
| hsa-mir-495-3p | PBX3 |
| hsa-mir-495-3p | PCDH1 |
| hsa-mir-495-3p | PCMT1 |
| hsa-mir-495-3p | PFDN2 |
| hsa-mir-495-3p | ABCB1 |
| hsa-mir-495-3p | PRNP |
| hsa-mir-495-3p | SC5D |
| hsa-mir-495-3p | SCO1 |
| hsa-mir-495-3p | CCL2 |
| hsa-mir-495-3p | CXCL5 |
| hsa-mir-495-3p | SDC2 |
| hsa-mir-495-3p | SIX3 |
| hsa-mir-495-3p | SOX9 |
| hsa-mir-495-3p | SREBF1 |
| hsa-mir-495-3p | STX4 |
| hsa-mir-495-3p | TGFBR2 |
| hsa-mir-495-3p | TGIF1 |
| hsa-mir-495-3p | TOP2A |
| hsa-mir-495-3p | VEGFA |
| hsa-mir-495-3p | XRCC2 |
| hsa-mir-495-3p | SF1 |
| hsa-mir-495-3p | CNBP |
| hsa-mir-495-3p | MLLT10 |
| hsa-mir-495-3p | HMGA2 |
| hsa-mir-495-3p | COIL |
| hsa-mir-495-3p | NCK2 |
| hsa-mir-495-3p | CBX4 |
| hsa-mir-495-3p | TMEFF1 |
| hsa-mir-495-3p | CD164 |
| hsa-mir-495-3p | PER2 |
| hsa-mir-495-3p | WASL |
| hsa-mir-495-3p | VGLL4 |
| hsa-mir-495-3p | MLEC |
| hsa-mir-495-3p | AREL1 |
| hsa-mir-495-3p | MED13 |
| hsa-mir-495-3p | RNF41 |
| hsa-mir-495-3p | NPM3 |
| hsa-mir-495-3p | DLC1 |
| hsa-mir-495-3p | LYPLA1 |
| hsa-mir-495-3p | ZBTB18 |
| hsa-mir-495-3p | HEXIM1 |
| hsa-mir-495-3p | TXNIP |
| hsa-mir-495-3p | KDM5B |
| hsa-mir-495-3p | ARPP19 |
| hsa-mir-495-3p | ZNF460 |
| hsa-mir-495-3p | SMR3B |
| hsa-mir-495-3p | RAB10 |
| hsa-mir-495-3p | RAB31 |
| hsa-mir-495-3p | PTP4A3 |
| hsa-mir-495-3p | PCNX1 |
| hsa-mir-495-3p | TAB2 |
| hsa-mir-495-3p | TBC1D9 |
| hsa-mir-495-3p | MPRIP |
| hsa-mir-495-3p | PHLPP1 |
| hsa-mir-495-3p | NUP62 |
| hsa-mir-495-3p | TMEM2 |
| hsa-mir-495-3p | KIAA1549L |
| hsa-mir-495-3p | PNISR |
| hsa-mir-495-3p | NGDN |
| hsa-mir-495-3p | ZZZ3 |
| hsa-mir-495-3p | TOR1AIP1 |
| hsa-mir-495-3p | GALNT8 |
| hsa-mir-495-3p | TIMM10 |
| hsa-mir-495-3p | AGO2 |
| hsa-mir-495-3p | SNX24 |
| hsa-mir-495-3p | EEF2K |
| hsa-mir-495-3p | TNPO2 |
| hsa-mir-495-3p | ZNF354C |
| hsa-mir-495-3p | RNF141 |
| hsa-mir-495-3p | MRPL35 |
| hsa-mir-495-3p | CRLF3 |
| hsa-mir-495-3p | RNF138 |
| hsa-mir-495-3p | MIEF1 |
| hsa-mir-495-3p | DDIT4 |
| hsa-mir-495-3p | CRLS1 |
| hsa-mir-495-3p | RFK |
| hsa-mir-495-3p | EIF5A2 |
| hsa-mir-495-3p | ADAMTS9 |
| hsa-mir-495-3p | MTA3 |
| hsa-mir-495-3p | SMOC1 |
| hsa-mir-495-3p | LRRC4 |
| hsa-mir-495-3p | COPS7B |
| hsa-mir-495-3p | C6orf106 |
| hsa-mir-495-3p | FNDC3B |
| hsa-mir-495-3p | UBE2Z |
| hsa-mir-495-3p | MTMR9 |
| hsa-mir-495-3p | CDC73 |
| hsa-mir-495-3p | HMBOX1 |
| hsa-mir-495-3p | QSER1 |
| hsa-mir-495-3p | GEMIN6 |
| hsa-mir-495-3p | ZNF703 |
| hsa-mir-495-3p | KLHL15 |
| hsa-mir-495-3p | TTYH3 |
| hsa-mir-495-3p | SPNS1 |
| hsa-mir-495-3p | ZBTB37 |
| hsa-mir-495-3p | UTP4 |
| hsa-mir-495-3p | CEP19 |
| hsa-mir-495-3p | SLC25A46 |
| hsa-mir-495-3p | ANKRD40 |
| hsa-mir-495-3p | BTF3L4 |
| hsa-mir-495-3p | MYADM |
| hsa-mir-495-3p | MRRF |
| hsa-mir-495-3p | ZBTB47 |
| hsa-mir-495-3p | TP53INP1 |
| hsa-mir-495-3p | PRRT2 |
| hsa-mir-495-3p | ZNF573 |
| hsa-mir-495-3p | WTIP |
| hsa-mir-495-3p | UHMK1 |
| hsa-mir-495-3p | EMB |
| hsa-mir-495-3p | DNAJC21 |
| hsa-mir-495-3p | C5orf24 |
| hsa-mir-495-3p | TMEM68 |
| hsa-mir-495-3p | SAMD8 |
| hsa-mir-495-3p | EIF5AL1 |
| hsa-mir-495-3p | PHF13 |
| hsa-mir-495-3p | CDCA2 |
| hsa-mir-495-3p | ZNF431 |
| hsa-mir-495-3p | CASP16P |
| hsa-mir-495-3p | C4orf46 |
| hsa-mir-495-3p | CALHM5 |
| hsa-mir-495-3p | ZNF740 |
| hsa-mir-495-3p | CCDC141 |
| hsa-mir-495-3p | ARL10 |
| hsa-mir-495-3p | SREK1IP1 |
| hsa-mir-495-3p | SFT2D2 |
| hsa-mir-495-3p | NHLRC3 |
| hsa-mir-495-3p | SCIMP |
| hsa-mir-495-3p | ZNF324B |
| hsa-mir-495-3p | ACBD7 |
| hsa-mir-495-3p | ZNF724 |
| hsa-mir-495-3p | TRIM67 |
| hsa-mir-495-3p | ZBTB8B |
| hsa-mir-495-3p | C16orf52 |
| hsa-mir-495-3p | SIGLEC14 |
| hsa-mir-495-3p | TMEM170B |
| hsa-mir-495-3p | MTRNR2L7 |
| hsa-mir-495-3p | MTRNR2L1 |
| hsa-mir-495-3p | MTRNR2L3 |
| hsa-mir-495-3p | MTRNR2L10 |
| hsa-mir-495-3p | MTRNR2L11 |
| hsa-mir-495-3p | MSANTD3-TMEFF1 |
| hsa-mir-299-5p | ACTC1 |
| hsa-mir-299-5p | ATP6V1A |
| hsa-mir-299-5p | CDKN1A |
| hsa-mir-299-5p | FOLR1 |
| hsa-mir-299-5p | H3F3B |
| hsa-mir-299-5p | HIF1A |
| hsa-mir-299-5p | LDLR |
| hsa-mir-299-5p | NEUROD1 |
| hsa-mir-299-5p | PBX2P1 |
| hsa-mir-299-5p | RGL2 |
| hsa-mir-299-5p | ROBO1 |
| hsa-mir-299-5p | SOX4 |
| hsa-mir-299-5p | SPP1 |
| hsa-mir-299-5p | STRN |
| hsa-mir-299-5p | HIST1H2BE |
| hsa-mir-299-5p | RECK |
| hsa-mir-299-5p | STX16 |
| hsa-mir-299-5p | HIST1H3F |
| hsa-mir-299-5p | ATG5 |
| hsa-mir-299-5p | SAE1 |
| hsa-mir-299-5p | PLXNC1 |
| hsa-mir-299-5p | ZNF256 |
| hsa-mir-299-5p | HAX1 |
| hsa-mir-299-5p | HOXB13 |
| hsa-mir-299-5p | SLC35A1 |
| hsa-mir-299-5p | POLQ |
| hsa-mir-299-5p | RAB10 |
| hsa-mir-299-5p | TCERG1 |
| hsa-mir-299-5p | PAPD7 |
| hsa-mir-299-5p | SEC63 |
| hsa-mir-299-5p | RAB21 |
| hsa-mir-299-5p | TNRC6B |
| hsa-mir-299-5p | TBC1D1 |
| hsa-mir-299-5p | CTDNEP1 |
| hsa-mir-299-5p | ARIH1 |
| hsa-mir-299-5p | TNRC6A |
| hsa-mir-299-5p | CERS2 |
| hsa-mir-299-5p | HDGFL3 |
| hsa-mir-299-5p | DCTN4 |
| hsa-mir-299-5p | KLF3 |
| hsa-mir-299-5p | LIMA1 |
| hsa-mir-299-5p | PHAX |
| hsa-mir-299-5p | POLE3 |
| hsa-mir-299-5p | DCAF16 |
| hsa-mir-299-5p | PSPC1 |
| hsa-mir-299-5p | KLHL42 |
| hsa-mir-299-5p | CREBZF |
| hsa-mir-299-5p | ZBTB10 |
| hsa-mir-299-5p | EDEM3 |
| hsa-mir-299-5p | CLPB |
| hsa-mir-299-5p | CDCA7 |
| hsa-mir-299-5p | DCUN1D5 |
| hsa-mir-299-5p | EIF1AD |
| hsa-mir-299-5p | SLX4 |
| hsa-mir-299-5p | TSLP |
| hsa-mir-299-5p | H2AFV |
| hsa-mir-299-5p | LRRC15 |
| hsa-mir-299-5p | FAM84A |
| hsa-mir-299-5p | CNKSR3 |
| hsa-mir-299-5p | TVP23C |
| hsa-mir-299-5p | ARL5B |
| hsa-mir-299-5p | SFT2D2 |
| hsa-mir-299-5p | MTRNR2L2 |
| hsa-mir-493-3p | ACTB |
| hsa-mir-493-3p | ALDOA |
| hsa-mir-493-3p | RHOC |
| hsa-mir-493-3p | ARHGAP5 |
| hsa-mir-493-3p | KIF1A |
| hsa-mir-493-3p | CALM1 |
| hsa-mir-493-3p | DDB1 |
| hsa-mir-493-3p | EIF4A1 |
| hsa-mir-493-3p | GNB1 |
| hsa-mir-493-3p | HNRNPU |
| hsa-mir-493-3p | HSPA4 |
| hsa-mir-493-3p | IARS |
| hsa-mir-493-3p | KCNJ4 |
| hsa-mir-493-3p | LGALS3BP |
| hsa-mir-493-3p | LIFR |
| hsa-mir-493-3p | MAP2 |
| hsa-mir-493-3p | MXI1 |
| hsa-mir-493-3p | NFATC3 |
| hsa-mir-493-3p | NKX6-1 |
| hsa-mir-493-3p | PAWR |
| hsa-mir-493-3p | PPP1CC |
| hsa-mir-493-3p | PRKCH |
| hsa-mir-493-3p | MAP2K7 |
| hsa-mir-493-3p | PSMD7 |
| hsa-mir-493-3p | PEX2 |
| hsa-mir-493-3p | RAD51 |
| hsa-mir-493-3p | TRIM27 |
| hsa-mir-493-3p | RPL3 |
| hsa-mir-493-3p | SIAH2 |
| hsa-mir-493-3p | SKP1 |
| hsa-mir-493-3p | SP100 |
| hsa-mir-493-3p | UBE2D3 |
| hsa-mir-493-3p | VLDLR |
| hsa-mir-493-3p | YWHAZ |
| hsa-mir-493-3p | CSDE1 |
| hsa-mir-493-3p | SLC7A5 |
| hsa-mir-493-3p | FZD4 |
| hsa-mir-493-3p | PEA15 |
| hsa-mir-493-3p | BTRC |
| hsa-mir-493-3p | XPR1 |
| hsa-mir-493-3p | SRSF11 |
| hsa-mir-493-3p | KCNB2 |
| hsa-mir-493-3p | PDE4DIP |
| hsa-mir-493-3p | DAZAP2 |
| hsa-mir-493-3p | SMG7 |
| hsa-mir-493-3p | HS3ST1 |
| hsa-mir-493-3p | CITED2 |
| hsa-mir-493-3p | CRTAP |
| hsa-mir-493-3p | CELF1 |
| hsa-mir-493-3p | TMED10 |
| hsa-mir-493-3p | RAB31 |
| hsa-mir-493-3p | ABHD2 |
| hsa-mir-493-3p | NXPH3 |
| hsa-mir-493-3p | COPG1 |
| hsa-mir-493-3p | ZNF507 |
| hsa-mir-493-3p | DKK1 |
| hsa-mir-493-3p | MYCBP2 |
| hsa-mir-493-3p | ZCCHC14 |
| hsa-mir-493-3p | SSBP2 |
| hsa-mir-493-3p | PLXNB2 |
| hsa-mir-493-3p | CLIC4 |
| hsa-mir-493-3p | RTL8A |
| hsa-mir-493-3p | ASAP1 |
| hsa-mir-493-3p | SH3GLB1 |
| hsa-mir-493-3p | GOLPH3L |
| hsa-mir-493-3p | FEM1A |
| hsa-mir-493-3p | VPS35 |
| hsa-mir-493-3p | CNDP2 |
| hsa-mir-493-3p | NKRF |
| hsa-mir-493-3p | ATP10D |
| hsa-mir-493-3p | CBX8 |
| hsa-mir-493-3p | TRIB3 |
| hsa-mir-493-3p | TMEM109 |
| hsa-mir-493-3p | PPP1R3B |
| hsa-mir-493-3p | BRIP1 |
| hsa-mir-493-3p | MAP3K21 |
| hsa-mir-493-3p | LMNB2 |
| hsa-mir-493-3p | ZNF670 |
| hsa-mir-493-3p | ZNF689 |
| hsa-mir-493-3p | FOXP4 |
| hsa-mir-493-3p | SLC43A2 |
| hsa-mir-493-3p | AADACL3 |
| hsa-mir-493-3p | ZNF645 |
| hsa-mir-493-3p | DENND5B |
| hsa-mir-493-3p | ZNF678 |
| hsa-mir-493-3p | FMN1 |
| hsa-mir-493-3p | WDR82P1 |
| hsa-mir-493-3p | SYCE1L |
| hsa-mir-487b-3p | BMI1 |
| hsa-mir-487b-3p | GRM3 |
| hsa-mir-487b-3p | KRAS |
| hsa-mir-487b-3p | MYC |
| hsa-mir-487b-3p | THBS1 |
| hsa-mir-487b-3p | WNT5A |
| hsa-mir-487b-3p | EVI5 |
| hsa-mir-487b-3p | MAGI2 |
| hsa-mir-487b-3p | NXF1 |
| hsa-mir-487b-3p | ARPP19 |
| hsa-mir-487b-3p | SUZ12 |
| hsa-mir-487b-3p | MINK1 |
| hsa-mir-487b-3p | PRELID3B |
| hsa-mir-487b-3p | UFC1 |
| hsa-mir-487b-3p | SOBP |
| hsa-mir-487b-3p | TMEM40 |
| hsa-mir-487b-3p | L2HGDH |
| hsa-mir-487b-3p | CPEB4 |
| hsa-mir-487b-3p | KCTD10 |
| hsa-mir-487b-3p | FRRS1 |
| hsa-mir-654-3p | ACTN4 |
| hsa-mir-654-3p | ADRB1 |
| hsa-mir-654-3p | AKT1 |
| hsa-mir-654-3p | ENTPD1 |
| hsa-mir-654-3p | CDKN1A |
| hsa-mir-654-3p | CYP24A1 |
| hsa-mir-654-3p | DECR1 |
| hsa-mir-654-3p | ELK4 |
| hsa-mir-654-3p | CLN8 |
| hsa-mir-654-3p | ETS1 |
| hsa-mir-654-3p | EXT1 |
| hsa-mir-654-3p | FOXG1 |
| hsa-mir-654-3p | GABPB1 |
| hsa-mir-654-3p | GABRG1 |
| hsa-mir-654-3p | NR6A1 |
| hsa-mir-654-3p | GMDS |
| hsa-mir-654-3p | H2AFZ |
| hsa-mir-654-3p | HDGF |
| hsa-mir-654-3p | IFNGR2 |
| hsa-mir-654-3p | PDPK1 |
| hsa-mir-654-3p | POU2F1 |
| hsa-mir-654-3p | PKIA |
| hsa-mir-654-3p | PRKCD |
| hsa-mir-654-3p | TMPRSS15 |
| hsa-mir-654-3p | TFDP2 |
| hsa-mir-654-3p | SUMO1 |
| hsa-mir-654-3p | NR4A3 |
| hsa-mir-654-3p | CDKL2 |
| hsa-mir-654-3p | SDC3 |
| hsa-mir-654-3p | EXOG |
| hsa-mir-654-3p | ARL4C |
| hsa-mir-654-3p | EMC8 |
| hsa-mir-654-3p | PRSS21 |
| hsa-mir-654-3p | HHLA2 |
| hsa-mir-654-3p | ZNF652 |
| hsa-mir-654-3p | RALY |
| hsa-mir-654-3p | LRCH1 |
| hsa-mir-654-3p | KIAA1549L |
| hsa-mir-654-3p | PLEKHG4 |
| hsa-mir-654-3p | TMEM158 |
| hsa-mir-654-3p | LSM3 |
| hsa-mir-654-3p | MYLIP |
| hsa-mir-654-3p | ERGIC2 |
| hsa-mir-654-3p | KCNK10 |
| hsa-mir-654-3p | ZNF562 |
| hsa-mir-654-3p | BSDC1 |
| hsa-mir-654-3p | MAP7D1 |
| hsa-mir-654-3p | PMEPA1 |
| hsa-mir-654-3p | SCYL3 |
| hsa-mir-654-3p | DDX55 |
| hsa-mir-654-3p | PTBP2 |
| hsa-mir-654-3p | ZMAT3 |
| hsa-mir-654-3p | NUCKS1 |
| hsa-mir-654-3p | DEPTOR |
| hsa-mir-654-3p | RPF2 |
| hsa-mir-654-3p | ZC3H12C |
| hsa-mir-654-3p | BTF3L4 |
| hsa-mir-654-3p | CD300LB |
| hsa-mir-654-3p | GJD3 |
| hsa-mir-654-3p | KRT222 |
| hsa-mir-654-3p | KIF18B |
| hsa-mir-654-3p | ZNF578 |
| hsa-mir-654-3p | ZNF610 |
| hsa-mir-654-3p | CCDC83 |
| hsa-mir-654-3p | TIPRL |
| hsa-mir-654-3p | DCAF4L1 |
| hsa-mir-654-3p | TRIM42 |
| hsa-mir-654-3p | NHSL2 |
| hsa-mir-654-3p | FBXO47 |
| hsa-mir-654-3p | ARGFX |
