# Supplementary material for: Danggui Buxue Decoction Ameliorates Idiopathic Pulmonary Fibrosis through MicroRNA and Messenger RNA Regulatory Network
Source: Evid Based Complement Alternat Med. 2022 Apr 26;2022:3439656. doi: 10.1155/2022/3439656 (PMC9064538; doi:10.1155/2022/3439656)
Supplement: Supplementary Materials — Table S1: DGBXD granules. Table S2: Szapiel score system. Table S3: Ashcroft score system. Table S4: predicted target genes of upregulated DE-miRNAs (n = 1285). Table S5: predicted target genes of downregulated DE-miRNAs (n = 1411). Table S6: upregulated DE-mRNAs (n = 1160). Table S7: downregulated DE-mRNAs (n = 1427). Table S8: corresponding gene symbols of RA and RAS. [file 3439656.f1.zip › 3439656.f1/Table S5 Predicted target genes of downregulated DE-miRNAs (n=1411).docx]

**Table S5:** Predicted target genes of downregulated DE-miRNAs (n=1411).

| **miRNA ID** | **Gene** |
| --- | --- |
| hsa-mir-203a-3p | ABL1 |
| hsa-mir-203a-3p | ACVR2B |
| hsa-mir-203a-3p | AKT2 |
| hsa-mir-203a-3p | ALOX15 |
| hsa-mir-203a-3p | BIRC5 |
| hsa-mir-203a-3p | ASNA1 |
| hsa-mir-203a-3p | ASPA |
| hsa-mir-203a-3p | ATM |
| hsa-mir-203a-3p | BCL2L2 |
| hsa-mir-203a-3p | BCL7A |
| hsa-mir-203a-3p | BMI1 |
| hsa-mir-203a-3p | BMPR1A |
| hsa-mir-203a-3p | BTG1 |
| hsa-mir-203a-3p | CALR |
| hsa-mir-203a-3p | CAV1 |
| hsa-mir-203a-3p | RUNX2 |
| hsa-mir-203a-3p | CDH1 |
| hsa-mir-203a-3p | CDH7 |
| hsa-mir-203a-3p | CDK6 |
| hsa-mir-203a-3p | FOXN3 |
| hsa-mir-203a-3p | CCR5 |
| hsa-mir-203a-3p | CREB1 |
| hsa-mir-203a-3p | DLX5 |
| hsa-mir-203a-3p | DUSP5 |
| hsa-mir-203a-3p | E2F1 |
| hsa-mir-203a-3p | E2F3 |
| hsa-mir-203a-3p | EDNRA |
| hsa-mir-203a-3p | EN2 |
| hsa-mir-203a-3p | STOM |
| hsa-mir-203a-3p | EYA4 |
| hsa-mir-203a-3p | EXT1 |
| hsa-mir-203a-3p | FGF2 |
| hsa-mir-203a-3p | FRK |
| hsa-mir-203a-3p | FZD2 |
| hsa-mir-203a-3p | G6PC |
| hsa-mir-203a-3p | GABRB1 |
| hsa-mir-203a-3p | GAS1 |
| hsa-mir-203a-3p | GATA6 |
| hsa-mir-203a-3p | GNAS |
| hsa-mir-203a-3p | RAPGEF1 |
| hsa-mir-203a-3p | GSK3B |
| hsa-mir-203a-3p | HOXA1 |
| hsa-mir-203a-3p | HOXD3 |
| hsa-mir-203a-3p | HTR2A |
| hsa-mir-203a-3p | IFIT1 |
| hsa-mir-203a-3p | IGF1R |
| hsa-mir-203a-3p | IGFBP5 |
| hsa-mir-203a-3p | IL6 |
| hsa-mir-203a-3p | IL7 |
| hsa-mir-203a-3p | CXCL8 |
| hsa-mir-203a-3p | TNFRSF9 |
| hsa-mir-203a-3p | FOXK2 |
| hsa-mir-203a-3p | JUN |
| hsa-mir-203a-3p | KCNJ2 |
| hsa-mir-203a-3p | KIF2A |
| hsa-mir-203a-3p | KIF5B |
| hsa-mir-203a-3p | LASP1 |
| hsa-mir-203a-3p | LDHA |
| hsa-mir-203a-3p | LIFR |
| hsa-mir-203a-3p | SMAD2 |
| hsa-mir-203a-3p | SMAD4 |
| hsa-mir-203a-3p | SMAD9 |
| hsa-mir-203a-3p | MBNL1 |
| hsa-mir-203a-3p | MMP1 |
| hsa-mir-203a-3p | MMP10 |
| hsa-mir-203a-3p | MYD88 |
| hsa-mir-203a-3p | NARS |
| hsa-mir-203a-3p | NCL |
| hsa-mir-203a-3p | NEK3 |
| hsa-mir-203a-3p | NFYA |
| hsa-mir-203a-3p | NPPC |
| hsa-mir-203a-3p | OSBP |
| hsa-mir-203a-3p | REG3A |
| hsa-mir-203a-3p | PAX6 |
| hsa-mir-203a-3p | PDE7A |
| hsa-mir-203a-3p | PIK3CA |
| hsa-mir-203a-3p | PLAGL2 |
| hsa-mir-203a-3p | PLD2 |
| hsa-mir-203a-3p | PPP1CB |
| hsa-mir-203a-3p | PRKACB |
| hsa-mir-203a-3p | PRKCA |
| hsa-mir-203a-3p | MAPK8 |
| hsa-mir-203a-3p | MAPK9 |
| hsa-mir-203a-3p | PRNP |
| hsa-mir-203a-3p | RGL2 |
| hsa-mir-203a-3p | RAN |
| hsa-mir-203a-3p | RAP1A |
| hsa-mir-203a-3p | RAP2B |
| hsa-mir-203a-3p | RASA2 |
| hsa-mir-203a-3p | ABCE1 |
| hsa-mir-203a-3p | SCO1 |
| hsa-mir-203a-3p | SIX1 |
| hsa-mir-203a-3p | SNAI2 |
| hsa-mir-203a-3p | SNAI1 |
| hsa-mir-203a-3p | SOD2 |
| hsa-mir-203a-3p | SON |
| hsa-mir-203a-3p | SRC |
| hsa-mir-203a-3p | STAT1 |
| hsa-mir-203a-3p | SYK |
| hsa-mir-203a-3p | TCF4 |
| hsa-mir-203a-3p | ZEB1 |
| hsa-mir-203a-3p | DYNLT1 |
| hsa-mir-203a-3p | NR2F2 |
| hsa-mir-203a-3p | NKX2-1 |
| hsa-mir-203a-3p | TNF |
| hsa-mir-203a-3p | TOP2A |
| hsa-mir-203a-3p | TPD52L1 |
| hsa-mir-203a-3p | NR2C2 |
| hsa-mir-203a-3p | TRPS1 |
| hsa-mir-203a-3p | TYMS |
| hsa-mir-203a-3p | SUMO1 |
| hsa-mir-203a-3p | UVRAG |
| hsa-mir-203a-3p | VEGFA |
| hsa-mir-203a-3p | ZNF24 |
| hsa-mir-203a-3p | ZNF148 |
| hsa-mir-203a-3p | ZMYM2 |
| hsa-mir-203a-3p | ZNF200 |
| hsa-mir-203a-3p | MAFK |
| hsa-mir-203a-3p | NCOA4 |
| hsa-mir-203a-3p | ANP32A |
| hsa-mir-203a-3p | ARID1A |
| hsa-mir-203a-3p | PIP5K1A |
| hsa-mir-203a-3p | DYRK3 |
| hsa-mir-203a-3p | CUL3 |
| hsa-mir-203a-3p | PPM1D |
| hsa-mir-203a-3p | CASK |
| hsa-mir-203a-3p | TP63 |
| hsa-mir-203a-3p | DDX3Y |
| hsa-mir-203a-3p | SNX4 |
| hsa-mir-203a-3p | BANF1 |
| hsa-mir-203a-3p | CDKL2 |
| hsa-mir-203a-3p | SOCS3 |
| hsa-mir-203a-3p | MAP3K13 |
| hsa-mir-203a-3p | SOCS6 |
| hsa-mir-203a-3p | CIAO1 |
| hsa-mir-203a-3p | TJP2 |
| hsa-mir-203a-3p | RASAL2 |
| hsa-mir-203a-3p | ROCK2 |
| hsa-mir-203a-3p | CLOCK |
| hsa-mir-203a-3p | VGLL4 |
| hsa-mir-203a-3p | KIAA0408 |
| hsa-mir-203a-3p | DAZAP2 |
| hsa-mir-203a-3p | TSC22D2 |
| hsa-mir-203a-3p | ZEB2 |
| hsa-mir-203a-3p | HELZ |
| hsa-mir-203a-3p | SLC23A1 |
| hsa-mir-203a-3p | THRAP3 |
| hsa-mir-203a-3p | HNRNPDL |
| hsa-mir-203a-3p | DNAJB6 |
| hsa-mir-203a-3p | RNF41 |
| hsa-mir-203a-3p | HNRNPR |
| hsa-mir-203a-3p | DSCR3 |
| hsa-mir-203a-3p | DLC1 |
| hsa-mir-203a-3p | IPO7 |
| hsa-mir-203a-3p | HEXIM1 |
| hsa-mir-203a-3p | ARID3B |
| hsa-mir-203a-3p | KHDRBS1 |
| hsa-mir-203a-3p | CELF2 |
| hsa-mir-203a-3p | KIF1C |
| hsa-mir-203a-3p | NUP50 |
| hsa-mir-203a-3p | ARPP19 |
| hsa-mir-203a-3p | ZNF268 |
| hsa-mir-203a-3p | IL24 |
| hsa-mir-203a-3p | CIT |
| hsa-mir-203a-3p | FGFR1OP |
| hsa-mir-203a-3p | ZWINT |
| hsa-mir-203a-3p | WIF1 |
| hsa-mir-203a-3p | POLI |
| hsa-mir-203a-3p | SEC63 |
| hsa-mir-203a-3p | PDCD10 |
| hsa-mir-203a-3p | PARK7 |
| hsa-mir-203a-3p | ZNF652 |
| hsa-mir-203a-3p | FAM208A |
| hsa-mir-203a-3p | SATB2 |
| hsa-mir-203a-3p | ZCCHC11 |
| hsa-mir-203a-3p | SYNM |
| hsa-mir-203a-3p | SMCHD1 |
| hsa-mir-203a-3p | UFL1 |
| hsa-mir-203a-3p | SLC44A1 |
| hsa-mir-203a-3p | SH3BP4 |
| hsa-mir-203a-3p | HECTD1 |
| hsa-mir-203a-3p | ZNF451 |
| hsa-mir-203a-3p | SZRD1 |
| hsa-mir-203a-3p | FBXL3 |
| hsa-mir-203a-3p | FBXL5 |
| hsa-mir-203a-3p | CNNM4 |
| hsa-mir-203a-3p | CNNM3 |
| hsa-mir-203a-3p | RGS17 |
| hsa-mir-203a-3p | GREM1 |
| hsa-mir-203a-3p | TMEM97 |
| hsa-mir-203a-3p | TBK1 |
| hsa-mir-203a-3p | SERTAD3 |
| hsa-mir-203a-3p | PSAT1 |
| hsa-mir-203a-3p | DNTTIP2 |
| hsa-mir-203a-3p | ASAP1 |
| hsa-mir-203a-3p | RNF141 |
| hsa-mir-203a-3p | GLRX2 |
| hsa-mir-203a-3p | TRNT1 |
| hsa-mir-203a-3p | SH3GLB1 |
| hsa-mir-203a-3p | RLIM |
| hsa-mir-203a-3p | GDAP1 |
| hsa-mir-203a-3p | EGLN1 |
| hsa-mir-203a-3p | KLHL28 |
| hsa-mir-203a-3p | NSD3 |
| hsa-mir-203a-3p | TMEM70 |
| hsa-mir-203a-3p | DNAAF2 |
| hsa-mir-203a-3p | ZNF654 |
| hsa-mir-203a-3p | FBXW7 |
| hsa-mir-203a-3p | PI4K2B |
| hsa-mir-203a-3p | SLC39A9 |
| hsa-mir-203a-3p | TSR1 |
| hsa-mir-203a-3p | ENAH |
| hsa-mir-203a-3p | EXOC2 |
| hsa-mir-203a-3p | CAND1 |
| hsa-mir-203a-3p | C20orf24 |
| hsa-mir-203a-3p | GPCPD1 |
| hsa-mir-203a-3p | DHX33 |
| hsa-mir-203a-3p | CDC42SE2 |
| hsa-mir-203a-3p | SLC45A4 |
| hsa-mir-203a-3p | RAB22A |
| hsa-mir-203a-3p | CYP20A1 |
| hsa-mir-203a-3p | SLC12A5 |
| hsa-mir-203a-3p | SERINC1 |
| hsa-mir-203a-3p | SRGAP1 |
| hsa-mir-203a-3p | NUFIP2 |
| hsa-mir-203a-3p | TRMT5 |
| hsa-mir-203a-3p | HOMEZ |
| hsa-mir-203a-3p | SLAIN2 |
| hsa-mir-203a-3p | KIF13A |
| hsa-mir-203a-3p | ARHGEF28 |
| hsa-mir-203a-3p | SMURF2 |
| hsa-mir-203a-3p | RMND5A |
| hsa-mir-203a-3p | BCL11B |
| hsa-mir-203a-3p | RAPH1 |
| hsa-mir-203a-3p | WDR77 |
| hsa-mir-203a-3p | FYCO1 |
| hsa-mir-203a-3p | MCTP1 |
| hsa-mir-203a-3p | CBLL1 |
| hsa-mir-203a-3p | KLHL15 |
| hsa-mir-203a-3p | CPEB4 |
| hsa-mir-203a-3p | CXXC4 |
| hsa-mir-203a-3p | CLPTM1L |
| hsa-mir-203a-3p | NETO2 |
| hsa-mir-203a-3p | MRO |
| hsa-mir-203a-3p | NCALD |
| hsa-mir-203a-3p | PCGF6 |
| hsa-mir-203a-3p | POLR1B |
| hsa-mir-203a-3p | GINS4 |
| hsa-mir-203a-3p | ZBED3 |
| hsa-mir-203a-3p | C15orf48 |
| hsa-mir-203a-3p | LCOR |
| hsa-mir-203a-3p | PARD6B |
| hsa-mir-203a-3p | DGAT2 |
| hsa-mir-203a-3p | NUDCD1 |
| hsa-mir-203a-3p | TRIM4 |
| hsa-mir-203a-3p | MIDN |
| hsa-mir-203a-3p | MCFD2 |
| hsa-mir-203a-3p | FMNL2 |
| hsa-mir-203a-3p | FLYWCH2 |
| hsa-mir-203a-3p | MARCH3 |
| hsa-mir-203a-3p | COX20 |
| hsa-mir-203a-3p | GSTO2 |
| hsa-mir-203a-3p | NAA30 |
| hsa-mir-203a-3p | MSI2 |
| hsa-mir-203a-3p | ANKRD13B |
| hsa-mir-203a-3p | CCSAP |
| hsa-mir-203a-3p | TRIM71 |
| hsa-mir-203a-3p | SPATA18 |
| hsa-mir-203a-3p | JMY |
| hsa-mir-203a-3p | PM20D2 |
| hsa-mir-203a-3p | MTPN |
| hsa-mir-203a-3p | TOR1AIP2 |
| hsa-mir-203a-3p | UBXN2A |
| hsa-mir-203a-3p | GPR156 |
| hsa-mir-203a-3p | PRICKLE2 |
| hsa-mir-203a-3p | GLIS3 |
| hsa-mir-203a-3p | OIT3 |
| hsa-mir-203a-3p | ZNF367 |
| hsa-mir-203a-3p | ARID2 |
| hsa-mir-203a-3p | TRIML2 |
| hsa-mir-203a-3p | TBCEL |
| hsa-mir-203a-3p | SPATA13 |
| hsa-mir-203a-3p | ZUFSP |
| hsa-mir-203a-3p | C6orf223 |
| hsa-mir-203a-3p | FOXK1 |
| hsa-mir-203a-3p | SEMA3D |
| hsa-mir-203a-3p | PRR14L |
| hsa-mir-203a-3p | IPMK |
| hsa-mir-203a-3p | LCLAT1 |
| hsa-mir-203a-3p | CADM2 |
| hsa-mir-203a-3p | MCM9 |
| hsa-mir-203a-3p | GXYLT1 |
| hsa-mir-203a-3p | HEPHL1 |
| hsa-mir-203a-3p | LCE1A |
| hsa-mir-203a-3p | PEAR1 |
| hsa-mir-203a-3p | CERKL |
| hsa-mir-203a-3p | BOLA3 |
| hsa-mir-203a-3p | LIN28B |
| hsa-mir-203a-3p | PRAMEF8 |
| hsa-mir-203a-3p | PIM3 |
| hsa-mir-203a-3p | MXRA7 |
| hsa-mir-203a-3p | PRAMEF7 |
| hsa-mir-203a-3p | LOH12CR2 |
| hsa-mir-203a-3p | ZNF704 |
| hsa-mir-203a-3p | TMPPE |
| hsa-mir-203a-3p | LINC00598 |
| hsa-mir-203a-3p | STMP1 |
| hsa-mir-203a-3p | GXYLT2 |
| hsa-mir-30b-5p | ACTC1 |
| hsa-mir-30b-5p | PARP1 |
| hsa-mir-30b-5p | AP2A1 |
| hsa-mir-30b-5p | APLP2 |
| hsa-mir-30b-5p | TRIM23 |
| hsa-mir-30b-5p | ARF1 |
| hsa-mir-30b-5p | SERPINC1 |
| hsa-mir-30b-5p | ATM |
| hsa-mir-30b-5p | ATP2A2 |
| hsa-mir-30b-5p | ATP5C1 |
| hsa-mir-30b-5p | BCL2 |
| hsa-mir-30b-5p | BCL6 |
| hsa-mir-30b-5p | BCL9 |
| hsa-mir-30b-5p | PRDM1 |
| hsa-mir-30b-5p | ZFP36L1 |
| hsa-mir-30b-5p | C1QBP |
| hsa-mir-30b-5p | CASP3 |
| hsa-mir-30b-5p | CAT |
| hsa-mir-30b-5p | RUNX2 |
| hsa-mir-30b-5p | CCNF |
| hsa-mir-30b-5p | ENTPD1 |
| hsa-mir-30b-5p | CHAT |
| hsa-mir-30b-5p | CHD1 |
| hsa-mir-30b-5p | CREM |
| hsa-mir-30b-5p | CSF1 |
| hsa-mir-30b-5p | DNMT1 |
| hsa-mir-30b-5p | ARID3A |
| hsa-mir-30b-5p | CELSR3 |
| hsa-mir-30b-5p | EIF2S1 |
| hsa-mir-30b-5p | EIF2B1 |
| hsa-mir-30b-5p | EPB41 |
| hsa-mir-30b-5p | ERG |
| hsa-mir-30b-5p | EYA3 |
| hsa-mir-30b-5p | FANCF |
| hsa-mir-30b-5p | FOXG1 |
| hsa-mir-30b-5p | FRZB |
| hsa-mir-30b-5p | FUCA1 |
| hsa-mir-30b-5p | GAB1 |
| hsa-mir-30b-5p | GALNT1 |
| hsa-mir-30b-5p | B4GALT1 |
| hsa-mir-30b-5p | GCLC |
| hsa-mir-30b-5p | GNAI2 |
| hsa-mir-30b-5p | GNAZ |
| hsa-mir-30b-5p | GOLGA1 |
| hsa-mir-30b-5p | GTF2E2 |
| hsa-mir-30b-5p | HMGN2 |
| hsa-mir-30b-5p | FOXA1 |
| hsa-mir-30b-5p | HOXA1 |
| hsa-mir-30b-5p | FOXN2 |
| hsa-mir-30b-5p | IFNAR2 |
| hsa-mir-30b-5p | IL1A |
| hsa-mir-30b-5p | IMPDH2 |
| hsa-mir-30b-5p | IREB2 |
| hsa-mir-30b-5p | JAK1 |
| hsa-mir-30b-5p | KIF5B |
| hsa-mir-30b-5p | KIF11 |
| hsa-mir-30b-5p | LCP1 |
| hsa-mir-30b-5p | LDLR |
| hsa-mir-30b-5p | LIFR |
| hsa-mir-30b-5p | SMAD1 |
| hsa-mir-30b-5p | MBNL1 |
| hsa-mir-30b-5p | MFAP3 |
| hsa-mir-30b-5p | MTR |
| hsa-mir-30b-5p | MYBL2 |
| hsa-mir-30b-5p | MYO1E |
| hsa-mir-30b-5p | PPP1R12A |
| hsa-mir-30b-5p | PPP1R12B |
| hsa-mir-30b-5p | NAP1L1 |
| hsa-mir-30b-5p | NOTCH1 |
| hsa-mir-30b-5p | OPHN1 |
| hsa-mir-30b-5p | SERPINE1 |
| hsa-mir-30b-5p | PAWR |
| hsa-mir-30b-5p | PCNT |
| hsa-mir-30b-5p | PDGFRB |
| hsa-mir-30b-5p | PGGT1B |
| hsa-mir-30b-5p | PGM3 |
| hsa-mir-30b-5p | PIK3C2B |
| hsa-mir-30b-5p | PIP4K2A |
| hsa-mir-30b-5p | PLAGL2 |
| hsa-mir-30b-5p | PLSCR1 |
| hsa-mir-30b-5p | PLXNA1 |
| hsa-mir-30b-5p | PNN |
| hsa-mir-30b-5p | POLRMT |
| hsa-mir-30b-5p | POU4F1 |
| hsa-mir-30b-5p | PPP1R2 |
| hsa-mir-30b-5p | PPP2R1B |
| hsa-mir-30b-5p | PPP3CB |
| hsa-mir-30b-5p | PRKAR1A |
| hsa-mir-30b-5p | MAPK8 |
| hsa-mir-30b-5p | PSMD7 |
| hsa-mir-30b-5p | TWF1 |
| hsa-mir-30b-5p | RAD23B |
| hsa-mir-30b-5p | RAP1B |
| hsa-mir-30b-5p | RBBP7 |
| hsa-mir-30b-5p | REV3L |
| hsa-mir-30b-5p | RPA2 |
| hsa-mir-30b-5p | RPS4X |
| hsa-mir-30b-5p | RPS27A |
| hsa-mir-30b-5p | RRM2 |
| hsa-mir-30b-5p | SBF1 |
| hsa-mir-30b-5p | CXCL11 |
| hsa-mir-30b-5p | SRSF7 |
| hsa-mir-30b-5p | SH3GL1 |
| hsa-mir-30b-5p | SHC1 |
| hsa-mir-30b-5p | SIX1 |
| hsa-mir-30b-5p | SKIL |
| hsa-mir-30b-5p | SNAI1 |
| hsa-mir-30b-5p | SNTB2 |
| hsa-mir-30b-5p | SOX4 |
| hsa-mir-30b-5p | SOX12 |
| hsa-mir-30b-5p | SP4 |
| hsa-mir-30b-5p | SRPRA |
| hsa-mir-30b-5p | STAU1 |
| hsa-mir-30b-5p | ELOVL4 |
| hsa-mir-30b-5p | STRN |
| hsa-mir-30b-5p | SYPL1 |
| hsa-mir-30b-5p | TAF4B |
| hsa-mir-30b-5p | DYNLT3 |
| hsa-mir-30b-5p | TFDP1 |
| hsa-mir-30b-5p | TGFA |
| hsa-mir-30b-5p | KLF10 |
| hsa-mir-30b-5p | TP53 |
| hsa-mir-30b-5p | TSPYL1 |
| hsa-mir-30b-5p | UBE2D3 |
| hsa-mir-30b-5p | XPO1 |
| hsa-mir-30b-5p | MKRN3 |
| hsa-mir-30b-5p | ZNF200 |
| hsa-mir-30b-5p | ZFAND5 |
| hsa-mir-30b-5p | PTP4A1 |
| hsa-mir-30b-5p | LRP8 |
| hsa-mir-30b-5p | SLC7A5 |
| hsa-mir-30b-5p | NCOA3 |
| hsa-mir-30b-5p | PICALM |
| hsa-mir-30b-5p | CDC7 |
| hsa-mir-30b-5p | SOCS1 |
| hsa-mir-30b-5p | STX16 |
| hsa-mir-30b-5p | BECN1 |
| hsa-mir-30b-5p | EED |
| hsa-mir-30b-5p | ADAM9 |
| hsa-mir-30b-5p | NAPG |
| hsa-mir-30b-5p | TNFRSF10B |
| hsa-mir-30b-5p | CREG1 |
| hsa-mir-30b-5p | APLN |
| hsa-mir-30b-5p | PER2 |
| hsa-mir-30b-5p | ST3GAL5 |
| hsa-mir-30b-5p | SOCS3 |
| hsa-mir-30b-5p | BAZ1B |
| hsa-mir-30b-5p | CLDN12 |
| hsa-mir-30b-5p | CCNE2 |
| hsa-mir-30b-5p | ATG12 |
| hsa-mir-30b-5p | VAPA |
| hsa-mir-30b-5p | PNMA1 |
| hsa-mir-30b-5p | KLF4 |
| hsa-mir-30b-5p | B4GALT5 |
| hsa-mir-30b-5p | ZNF264 |
| hsa-mir-30b-5p | RASAL2 |
| hsa-mir-30b-5p | ROCK2 |
| hsa-mir-30b-5p | SLC4A7 |
| hsa-mir-30b-5p | TBPL1 |
| hsa-mir-30b-5p | BAG4 |
| hsa-mir-30b-5p | H6PD |
| hsa-mir-30b-5p | PREPL |
| hsa-mir-30b-5p | SH3PXD2A |
| hsa-mir-30b-5p | IQCB1 |
| hsa-mir-30b-5p | UBE3C |
| hsa-mir-30b-5p | ZNF646 |
| hsa-mir-30b-5p | JADE3 |
| hsa-mir-30b-5p | MATR3 |
| hsa-mir-30b-5p | CEP350 |
| hsa-mir-30b-5p | ZBTB39 |
| hsa-mir-30b-5p | JOSD1 |
| hsa-mir-30b-5p | GFPT2 |
| hsa-mir-30b-5p | GNPDA1 |
| hsa-mir-30b-5p | TANK |
| hsa-mir-30b-5p | SH2B3 |
| hsa-mir-30b-5p | SAE1 |
| hsa-mir-30b-5p | FEM1B |
| hsa-mir-30b-5p | G3BP1 |
| hsa-mir-30b-5p | MBNL2 |
| hsa-mir-30b-5p | LHFPL2 |
| hsa-mir-30b-5p | PLIN3 |
| hsa-mir-30b-5p | ZMPSTE24 |
| hsa-mir-30b-5p | MARCH6 |
| hsa-mir-30b-5p | SCML2 |
| hsa-mir-30b-5p | CFDP1 |
| hsa-mir-30b-5p | RBM14 |
| hsa-mir-30b-5p | ERLIN1 |
| hsa-mir-30b-5p | CELF1 |
| hsa-mir-30b-5p | GNA13 |
| hsa-mir-30b-5p | NFAT5 |
| hsa-mir-30b-5p | ARPP19 |
| hsa-mir-30b-5p | ZNF460 |
| hsa-mir-30b-5p | SEC24A |
| hsa-mir-30b-5p | FRS2 |
| hsa-mir-30b-5p | RAB10 |
| hsa-mir-30b-5p | DBF4 |
| hsa-mir-30b-5p | TMED2 |
| hsa-mir-30b-5p | FERMT2 |
| hsa-mir-30b-5p | PDCD10 |
| hsa-mir-30b-5p | CBX3 |
| hsa-mir-30b-5p | IKZF2 |
| hsa-mir-30b-5p | MTF2 |
| hsa-mir-30b-5p | VASH1 |
| hsa-mir-30b-5p | ZNF507 |
| hsa-mir-30b-5p | ENPP4 |
| hsa-mir-30b-5p | MLXIP |
| hsa-mir-30b-5p | WDR37 |
| hsa-mir-30b-5p | BAHD1 |
| hsa-mir-30b-5p | HABP4 |
| hsa-mir-30b-5p | RAB18 |
| hsa-mir-30b-5p | CEP152 |
| hsa-mir-30b-5p | KLHDC10 |
| hsa-mir-30b-5p | SNRNP200 |
| hsa-mir-30b-5p | MAST3 |
| hsa-mir-30b-5p | NCOA6 |
| hsa-mir-30b-5p | AVL9 |
| hsa-mir-30b-5p | PEG10 |
| hsa-mir-30b-5p | HIC2 |
| hsa-mir-30b-5p | CLCC1 |
| hsa-mir-30b-5p | WDR43 |
| hsa-mir-30b-5p | TTLL12 |
| hsa-mir-30b-5p | UBXN4 |
| hsa-mir-30b-5p | JADE2 |
| hsa-mir-30b-5p | LARP1 |
| hsa-mir-30b-5p | ZDHHC17 |
| hsa-mir-30b-5p | HEY1 |
| hsa-mir-30b-5p | LRRC8B |
| hsa-mir-30b-5p | DDAH1 |
| hsa-mir-30b-5p | CD2AP |
| hsa-mir-30b-5p | ZMYND8 |
| hsa-mir-30b-5p | KPNA6 |
| hsa-mir-30b-5p | STX12 |
| hsa-mir-30b-5p | RASGRP3 |
| hsa-mir-30b-5p | NIPBL |
| hsa-mir-30b-5p | DCAF12 |
| hsa-mir-30b-5p | OLFML2B |
| hsa-mir-30b-5p | TBC1D10B |
| hsa-mir-30b-5p | GLCE |
| hsa-mir-30b-5p | SERBP1 |
| hsa-mir-30b-5p | BLOC1S6 |
| hsa-mir-30b-5p | FBXO3 |
| hsa-mir-30b-5p | SACS |
| hsa-mir-30b-5p | VPS41 |
| hsa-mir-30b-5p | AFF4 |
| hsa-mir-30b-5p | TNRC6A |
| hsa-mir-30b-5p | EML4 |
| hsa-mir-30b-5p | MYLIP |
| hsa-mir-30b-5p | UBN1 |
| hsa-mir-30b-5p | NRBP1 |
| hsa-mir-30b-5p | IL21R |
| hsa-mir-30b-5p | TMED5 |
| hsa-mir-30b-5p | ASB3 |
| hsa-mir-30b-5p | DCTN4 |
| hsa-mir-30b-5p | IER5 |
| hsa-mir-30b-5p | CHST15 |
| hsa-mir-30b-5p | ZNF589 |
| hsa-mir-30b-5p | FAM8A1 |
| hsa-mir-30b-5p | RNF138 |
| hsa-mir-30b-5p | AZIN1 |
| hsa-mir-30b-5p | SIX4 |
| hsa-mir-30b-5p | BTBD1 |
| hsa-mir-30b-5p | CSNK1G1 |
| hsa-mir-30b-5p | DCUN1D1 |
| hsa-mir-30b-5p | KCTD5 |
| hsa-mir-30b-5p | PRMT7 |
| hsa-mir-30b-5p | USP53 |
| hsa-mir-30b-5p | DLL4 |
| hsa-mir-30b-5p | TMEM106B |
| hsa-mir-30b-5p | OTUD4 |
| hsa-mir-30b-5p | KLHL28 |
| hsa-mir-30b-5p | PGPEP1 |
| hsa-mir-30b-5p | UHRF1BP1 |
| hsa-mir-30b-5p | ZNF770 |
| hsa-mir-30b-5p | STX17 |
| hsa-mir-30b-5p | SOBP |
| hsa-mir-30b-5p | FANCL |
| hsa-mir-30b-5p | LRRC8D |
| hsa-mir-30b-5p | SEC61A2 |
| hsa-mir-30b-5p | RNF220 |
| hsa-mir-30b-5p | RIF1 |
| hsa-mir-30b-5p | PBRM1 |
| hsa-mir-30b-5p | SETD5 |
| hsa-mir-30b-5p | SLC38A7 |
| hsa-mir-30b-5p | PCMTD2 |
| hsa-mir-30b-5p | C7orf43 |
| hsa-mir-30b-5p | LIN7C |
| hsa-mir-30b-5p | ACER3 |
| hsa-mir-30b-5p | SLC35C1 |
| hsa-mir-30b-5p | YOD1 |
| hsa-mir-30b-5p | MED29 |
| hsa-mir-30b-5p | CDC37L1 |
| hsa-mir-30b-5p | LMBR1L |
| hsa-mir-30b-5p | POLR3E |
| hsa-mir-30b-5p | BTBD7 |
| hsa-mir-30b-5p | N4BP2 |
| hsa-mir-30b-5p | MBNL3 |
| hsa-mir-30b-5p | TRERF1 |
| hsa-mir-30b-5p | BDP1 |
| hsa-mir-30b-5p | KDM3A |
| hsa-mir-30b-5p | CAND1 |
| hsa-mir-30b-5p | ACTR10 |
| hsa-mir-30b-5p | NDUFA12 |
| hsa-mir-30b-5p | EIF5A2 |
| hsa-mir-30b-5p | PHTF2 |
| hsa-mir-30b-5p | RAB22A |
| hsa-mir-30b-5p | MIB1 |
| hsa-mir-30b-5p | TAOK1 |
| hsa-mir-30b-5p | SEMA6A |
| hsa-mir-30b-5p | MARCH4 |
| hsa-mir-30b-5p | PCDH10 |
| hsa-mir-30b-5p | TSHZ3 |
| hsa-mir-30b-5p | SHROOM3 |
| hsa-mir-30b-5p | TNRC6C |
| hsa-mir-30b-5p | USP37 |
| hsa-mir-30b-5p | EPG5 |
| hsa-mir-30b-5p | ANKRA2 |
| hsa-mir-30b-5p | RRAGD |
| hsa-mir-30b-5p | ELOVL5 |
| hsa-mir-30b-5p | RBSN |
| hsa-mir-30b-5p | PAPD5 |
| hsa-mir-30b-5p | IKZF4 |
| hsa-mir-30b-5p | GIGYF1 |
| hsa-mir-30b-5p | COPS7B |
| hsa-mir-30b-5p | S100PBP |
| hsa-mir-30b-5p | RFX7 |
| hsa-mir-30b-5p | VPS33A |
| hsa-mir-30b-5p | MARCKSL1 |
| hsa-mir-30b-5p | CAMKV |
| hsa-mir-30b-5p | FYCO1 |
| hsa-mir-30b-5p | VCPKMT |
| hsa-mir-30b-5p | AKIRIN1 |
| hsa-mir-30b-5p | DHX40 |
| hsa-mir-30b-5p | ALG9 |
| hsa-mir-30b-5p | RNF122 |
| hsa-mir-30b-5p | LPCAT1 |
| hsa-mir-30b-5p | NAA25 |
| hsa-mir-30b-5p | SIKE1 |
| hsa-mir-30b-5p | EDC3 |
| hsa-mir-30b-5p | RUBCNL |
| hsa-mir-30b-5p | RNF34 |
| hsa-mir-30b-5p | PLEKHO2 |
| hsa-mir-30b-5p | KLHL15 |
| hsa-mir-30b-5p | CPEB4 |
| hsa-mir-30b-5p | WDR82 |
| hsa-mir-30b-5p | SLC35G2 |
| hsa-mir-30b-5p | CYB5B |
| hsa-mir-30b-5p | NDEL1 |
| hsa-mir-30b-5p | TXNDC5 |
| hsa-mir-30b-5p | MRO |
| hsa-mir-30b-5p | CDCA7 |
| hsa-mir-30b-5p | KREMEN1 |
| hsa-mir-30b-5p | B3GNT5 |
| hsa-mir-30b-5p | QRFPR |
| hsa-mir-30b-5p | SETD3 |
| hsa-mir-30b-5p | FYTTD1 |
| hsa-mir-30b-5p | RNF135 |
| hsa-mir-30b-5p | PCGF5 |
| hsa-mir-30b-5p | MSANTD4 |
| hsa-mir-30b-5p | HHIPL1 |
| hsa-mir-30b-5p | LCOR |
| hsa-mir-30b-5p | CBX2 |
| hsa-mir-30b-5p | ADO |
| hsa-mir-30b-5p | PPP1R15B |
| hsa-mir-30b-5p | FAM104A |
| hsa-mir-30b-5p | C8orf76 |
| hsa-mir-30b-5p | ZNRF1 |
| hsa-mir-30b-5p | STRIP1 |
| hsa-mir-30b-5p | ZCRB1 |
| hsa-mir-30b-5p | KBTBD6 |
| hsa-mir-30b-5p | MCFD2 |
| hsa-mir-30b-5p | MTDH |
| hsa-mir-30b-5p | SFXN1 |
| hsa-mir-30b-5p | WDR89 |
| hsa-mir-30b-5p | ADPRHL1 |
| hsa-mir-30b-5p | WDFY2 |
| hsa-mir-30b-5p | CTHRC1 |
| hsa-mir-30b-5p | MOGAT1 |
| hsa-mir-30b-5p | IP6K3 |
| hsa-mir-30b-5p | JDP2 |
| hsa-mir-30b-5p | ZNF543 |
| hsa-mir-30b-5p | LYPLAL1 |
| hsa-mir-30b-5p | TPRG1L |
| hsa-mir-30b-5p | PPARGC1B |
| hsa-mir-30b-5p | NACC2 |
| hsa-mir-30b-5p | ZSCAN29 |
| hsa-mir-30b-5p | DPY19L3 |
| hsa-mir-30b-5p | PHF13 |
| hsa-mir-30b-5p | FAM81B |
| hsa-mir-30b-5p | BMT2 |
| hsa-mir-30b-5p | FAM91A1 |
| hsa-mir-30b-5p | ZXDB |
| hsa-mir-30b-5p | PPTC7 |
| hsa-mir-30b-5p | SLFN5 |
| hsa-mir-30b-5p | RETREG3 |
| hsa-mir-30b-5p | ZNF567 |
| hsa-mir-30b-5p | MIER3 |
| hsa-mir-30b-5p | CCDC71L |
| hsa-mir-30b-5p | FBXO45 |
| hsa-mir-30b-5p | ZBTB38 |
| hsa-mir-30b-5p | LCLAT1 |
| hsa-mir-30b-5p | ZDHHC20 |
| hsa-mir-30b-5p | GCSAM |
| hsa-mir-30b-5p | GXYLT1 |
| hsa-mir-30b-5p | RGMB |
| hsa-mir-30b-5p | TRIM59 |
| hsa-mir-30b-5p | IFNE |
| hsa-mir-30b-5p | MIA3 |
| hsa-mir-30b-5p | PTAR1 |
| hsa-mir-30b-5p | SKIDA1 |
| hsa-mir-30b-5p | LIN28B |
| hsa-mir-30b-5p | TOMM5 |
| hsa-mir-30b-5p | MZT1 |
| hsa-mir-30b-5p | GOLGA8B |
| hsa-mir-30b-5p | GPR75-ASB3 |
| hsa-mir-30b-5p | MTRNR2L10 |
| hsa-mir-30b-5p | LRRC3C |
| hsa-mir-184 | AKT1 |
| hsa-mir-184 | AKT2 |
| hsa-mir-184 | ARHGDIA |
| hsa-mir-184 | BCL2 |
| hsa-mir-184 | BCL2L1 |
| hsa-mir-184 | TPP1 |
| hsa-mir-184 | CSF1 |
| hsa-mir-184 | GAS1 |
| hsa-mir-184 | INPPL1 |
| hsa-mir-184 | LIFR |
| hsa-mir-184 | MEIS3P1 |
| hsa-mir-184 | MYC |
| hsa-mir-184 | NFATC2 |
| hsa-mir-184 | NFIC |
| hsa-mir-184 | OPRD1 |
| hsa-mir-184 | PDGFB |
| hsa-mir-184 | PKM |
| hsa-mir-184 | PLAGL2 |
| hsa-mir-184 | PTPA |
| hsa-mir-184 | PRKCB |
| hsa-mir-184 | FSCN1 |
| hsa-mir-184 | SURF6 |
| hsa-mir-184 | TNFAIP2 |
| hsa-mir-184 | EZR |
| hsa-mir-184 | CNBP |
| hsa-mir-184 | SLC7A5 |
| hsa-mir-184 | PLPP3 |
| hsa-mir-184 | TM9SF4 |
| hsa-mir-184 | CARM1 |
| hsa-mir-184 | RAI1 |
| hsa-mir-184 | PPP1R13L |
| hsa-mir-184 | PPP6R1 |
| hsa-mir-184 | ZFPM2 |
| hsa-mir-184 | SND1 |
| hsa-mir-184 | TJP3 |
| hsa-mir-184 | AGO2 |
| hsa-mir-184 | DESI1 |
| hsa-mir-184 | TNPO2 |
| hsa-mir-184 | TACO1 |
| hsa-mir-184 | GNL3L |
| hsa-mir-184 | SELENOS |
| hsa-mir-184 | BIN3 |
| hsa-mir-184 | LRRC8A |
| hsa-mir-184 | CBX8 |
| hsa-mir-184 | FN3K |
| hsa-mir-184 | KLC2 |
| hsa-mir-184 | SOX7 |
| hsa-mir-184 | GPRIN1 |
| hsa-mir-184 | TNFRSF13C |
| hsa-mir-184 | USH1G |
| hsa-mir-184 | IFFO2 |
| hsa-mir-184 | ZSCAN25 |
| hsa-mir-184 | CEP170B |
| hsa-mir-184 | PEAK3 |
| hsa-mir-184 | POM121C |
| hsa-mir-375 | PARP1 |
| hsa-mir-375 | JAG1 |
| hsa-mir-375 | BIRC3 |
| hsa-mir-375 | XIAP |
| hsa-mir-375 | RHOA |
| hsa-mir-375 | ARHGDIA |
| hsa-mir-375 | AXL |
| hsa-mir-375 | BAK1 |
| hsa-mir-375 | BCL2 |
| hsa-mir-375 | ZFP36L2 |
| hsa-mir-375 | C1QBP |
| hsa-mir-375 | CALU |
| hsa-mir-375 | CASP3 |
| hsa-mir-375 | RUNX3 |
| hsa-mir-375 | CDC42 |
| hsa-mir-375 | CDKN2B |
| hsa-mir-375 | CDR2 |
| hsa-mir-375 | CENPF |
| hsa-mir-375 | CFL2 |
| hsa-mir-375 | CTSC |
| hsa-mir-375 | CHGB |
| hsa-mir-375 | CNN3 |
| hsa-mir-375 | COL12A1 |
| hsa-mir-375 | MAP3K8 |
| hsa-mir-375 | CPOX |
| hsa-mir-375 | CRABP2 |
| hsa-mir-375 | CSTF2 |
| hsa-mir-375 | CTGF |
| hsa-mir-375 | CTNNB1 |
| hsa-mir-375 | CXADR |
| hsa-mir-375 | TIMM8A |
| hsa-mir-375 | DLG4 |
| hsa-mir-375 | DNMT3B |
| hsa-mir-375 | DPYSL3 |
| hsa-mir-375 | DR1 |
| hsa-mir-375 | DUSP6 |
| hsa-mir-375 | DUT |
| hsa-mir-375 | EFNB2 |
| hsa-mir-375 | EIF2S1 |
| hsa-mir-375 | EIF4EBP2 |
| hsa-mir-375 | ELAVL2 |
| hsa-mir-375 | ELAVL4 |
| hsa-mir-375 | EMP1 |
| hsa-mir-375 | ERBB2 |
| hsa-mir-375 | EXT1 |
| hsa-mir-375 | F3 |
| hsa-mir-375 | ACSL3 |
| hsa-mir-375 | ACSL4 |
| hsa-mir-375 | FAH |
| hsa-mir-375 | FKBP1A |
| hsa-mir-375 | FKBP1B |
| hsa-mir-375 | FOLR1 |
| hsa-mir-375 | KDSR |
| hsa-mir-375 | IFI6 |
| hsa-mir-375 | GAS8 |
| hsa-mir-375 | GATA6 |
| hsa-mir-375 | GOLGA4 |
| hsa-mir-375 | RAPGEF1 |
| hsa-mir-375 | HK2 |
| hsa-mir-375 | MR1 |
| hsa-mir-375 | HOXB3 |
| hsa-mir-375 | HSP90AA1 |
| hsa-mir-375 | NDST1 |
| hsa-mir-375 | SP110 |
| hsa-mir-375 | IFIT1 |
| hsa-mir-375 | IGF1R |
| hsa-mir-375 | IGFBP3 |
| hsa-mir-375 | RBPJ |
| hsa-mir-375 | IL1RAP |
| hsa-mir-375 | ITGB1 |
| hsa-mir-375 | ITIH4 |
| hsa-mir-375 | JAK2 |
| hsa-mir-375 | KCNN4 |
| hsa-mir-375 | KCNQ2 |
| hsa-mir-375 | KHK |
| hsa-mir-375 | KPNA4 |
| hsa-mir-375 | KRT8 |
| hsa-mir-375 | LDHB |
| hsa-mir-375 | LRP5 |
| hsa-mir-375 | MRE11 |
| hsa-mir-375 | MST1R |
| hsa-mir-375 | MYBL1 |
| hsa-mir-375 | MYC |
| hsa-mir-375 | MYCN |
| hsa-mir-375 | NCAM1 |
| hsa-mir-375 | NPPB |
| hsa-mir-375 | ODF2 |
| hsa-mir-375 | OPA1 |
| hsa-mir-375 | OTX1 |
| hsa-mir-375 | PEBP1 |
| hsa-mir-375 | PAFAH1B1 |
| hsa-mir-375 | PAFAH2 |
| hsa-mir-375 | PRDX1 |
| hsa-mir-375 | PDK1 |
| hsa-mir-375 | PIK3CA |
| hsa-mir-375 | PLAG1 |
| hsa-mir-375 | PPP1R2 |
| hsa-mir-375 | PRKCA |
| hsa-mir-375 | MAPK3 |
| hsa-mir-375 | EIF2AK2 |
| hsa-mir-375 | PRKX |
| hsa-mir-375 | KLK10 |
| hsa-mir-375 | PTPN1 |
| hsa-mir-375 | PTPN12 |
| hsa-mir-375 | RAB3B |
| hsa-mir-375 | RAB6A |
| hsa-mir-375 | RBBP8 |
| hsa-mir-375 | RCN2 |
| hsa-mir-375 | RLF |
| hsa-mir-375 | RPGR |
| hsa-mir-375 | RPN1 |
| hsa-mir-375 | RPN2 |
| hsa-mir-375 | RTN2 |
| hsa-mir-375 | SDC1 |
| hsa-mir-375 | MAP2K4 |
| hsa-mir-375 | SET |
| hsa-mir-375 | SLC6A6 |
| hsa-mir-375 | SLC7A1 |
| hsa-mir-375 | SNAPC1 |
| hsa-mir-375 | SNRPA1 |
| hsa-mir-375 | SON |
| hsa-mir-375 | SOX2 |
| hsa-mir-375 | SP1 |
| hsa-mir-375 | STAT3 |
| hsa-mir-375 | STX3 |
| hsa-mir-375 | TAF2 |
| hsa-mir-375 | TCF12 |
| hsa-mir-375 | TFRC |
| hsa-mir-375 | TGFB2 |
| hsa-mir-375 | TGM2 |
| hsa-mir-375 | THRA |
| hsa-mir-375 | TNNI3 |
| hsa-mir-375 | TNS1 |
| hsa-mir-375 | TP53 |
| hsa-mir-375 | TPR |
| hsa-mir-375 | TRPS1 |
| hsa-mir-375 | UBE3A |
| hsa-mir-375 | UCHL3 |
| hsa-mir-375 | UGCG |
| hsa-mir-375 | USP1 |
| hsa-mir-375 | YWHAZ |
| hsa-mir-375 | ALMS1 |
| hsa-mir-375 | REEP5 |
| hsa-mir-375 | DEK |
| hsa-mir-375 | CUL5 |
| hsa-mir-375 | PTP4A2 |
| hsa-mir-375 | PUDP |
| hsa-mir-375 | FZD4 |
| hsa-mir-375 | FZD8 |
| hsa-mir-375 | SPOP |
| hsa-mir-375 | EEA1 |
| hsa-mir-375 | DGKD |
| hsa-mir-375 | AP3B1 |
| hsa-mir-375 | NUMB |
| hsa-mir-375 | EIF4G3 |
| hsa-mir-375 | DNAH11 |
| hsa-mir-375 | B3GALNT1 |
| hsa-mir-375 | TNFSF13 |
| hsa-mir-375 | PEX11B |
| hsa-mir-375 | ASAP2 |
| hsa-mir-375 | BCL10 |
| hsa-mir-375 | BSN |
| hsa-mir-375 | MBD2 |
| hsa-mir-375 | SPAG9 |
| hsa-mir-375 | SLC7A6 |
| hsa-mir-375 | CLDN1 |
| hsa-mir-375 | TBX19 |
| hsa-mir-375 | USP10 |
| hsa-mir-375 | EXO1 |
| hsa-mir-375 | EBAG9 |
| hsa-mir-375 | ZBED1 |
| hsa-mir-375 | XPR1 |
| hsa-mir-375 | NOLC1 |
| hsa-mir-375 | CRLF1 |
| hsa-mir-375 | KLF4 |
| hsa-mir-375 | EFTUD2 |
| hsa-mir-375 | RAB28 |
| hsa-mir-375 | HOMER2 |
| hsa-mir-375 | AKAP7 |
| hsa-mir-375 | PIGB |
| hsa-mir-375 | LITAF |
| hsa-mir-375 | RAB3D |
| hsa-mir-375 | WTAP |
| hsa-mir-375 | SEC24C |
| hsa-mir-375 | VGLL4 |
| hsa-mir-375 | HERPUD1 |
| hsa-mir-375 | PHACTR2 |
| hsa-mir-375 | SNPH |
| hsa-mir-375 | MLEC |
| hsa-mir-375 | KIAA0513 |
| hsa-mir-375 | KIAA0232 |
| hsa-mir-375 | TBC1D5 |
| hsa-mir-375 | RIMS3 |
| hsa-mir-375 | SERTAD2 |
| hsa-mir-375 | DAZAP2 |
| hsa-mir-375 | NUP58 |
| hsa-mir-375 | TSC22D2 |
| hsa-mir-375 | RB1CC1 |
| hsa-mir-375 | ARHGAP11A |
| hsa-mir-375 | TRIM66 |
| hsa-mir-375 | WDR1 |
| hsa-mir-375 | AMMECR1 |
| hsa-mir-375 | BCL2L11 |
| hsa-mir-375 | CTDSP2 |
| hsa-mir-375 | ABI2 |
| hsa-mir-375 | SLC25A15 |
| hsa-mir-375 | LHFPL2 |
| hsa-mir-375 | STX6 |
| hsa-mir-375 | GPHN |
| hsa-mir-375 | RIDA |
| hsa-mir-375 | FSTL3 |
| hsa-mir-375 | BCAS2 |
| hsa-mir-375 | RRAGB |
| hsa-mir-375 | YAP1 |
| hsa-mir-375 | TIMM17A |
| hsa-mir-375 | FAM3C |
| hsa-mir-375 | HOXB13 |
| hsa-mir-375 | SEC23A |
| hsa-mir-375 | CREB3 |
| hsa-mir-375 | SYNCRIP |
| hsa-mir-375 | UNC13B |
| hsa-mir-375 | SEMA3C |
| hsa-mir-375 | ATG7 |
| hsa-mir-375 | PROCR |
| hsa-mir-375 | CELF2 |
| hsa-mir-375 | AHCYL1 |
| hsa-mir-375 | SEC24A |
| hsa-mir-375 | SDCCAG3 |
| hsa-mir-375 | RAB10 |
| hsa-mir-375 | SUB1 |
| hsa-mir-375 | PRDX3 |
| hsa-mir-375 | LMAN2 |
| hsa-mir-375 | NUDT21 |
| hsa-mir-375 | PSIP1 |
| hsa-mir-375 | BAZ1A |
| hsa-mir-375 | RASSF8 |
| hsa-mir-375 | CBX3 |
| hsa-mir-375 | COG2 |
| hsa-mir-375 | PHLDA1 |
| hsa-mir-375 | DNAJC8 |
| hsa-mir-375 | CHSY1 |
| hsa-mir-375 | CARD8 |
| hsa-mir-375 | ELL2 |
| hsa-mir-375 | SLC4A1AP |
| hsa-mir-375 | NT5C2 |
| hsa-mir-375 | DIP2C |
| hsa-mir-375 | STK38L |
| hsa-mir-375 | SAMD4A |
| hsa-mir-375 | ERP44 |
| hsa-mir-375 | SIPA1L3 |
| hsa-mir-375 | DCUN1D4 |
| hsa-mir-375 | PSME4 |
| hsa-mir-375 | PHLPP1 |
| hsa-mir-375 | WWC1 |
| hsa-mir-375 | AGTPBP1 |
| hsa-mir-375 | IQCE |
| hsa-mir-375 | ICOSLG |
| hsa-mir-375 | RPGRIP1L |
| hsa-mir-375 | DPY19L1 |
| hsa-mir-375 | RHOQ |
| hsa-mir-375 | SLC35A3 |
| hsa-mir-375 | HEY1 |
| hsa-mir-375 | PHF3 |
| hsa-mir-375 | LEPROTL1 |
| hsa-mir-375 | SUZ12 |
| hsa-mir-375 | TNPO3 |
| hsa-mir-375 | GTPBP4 |
| hsa-mir-375 | ARL2BP |
| hsa-mir-375 | CORO1C |
| hsa-mir-375 | MKRN1 |
| hsa-mir-375 | SH3BP4 |
| hsa-mir-375 | IL17RA |
| hsa-mir-375 | FBXO8 |
| hsa-mir-375 | CKAP2 |
| hsa-mir-375 | DNAJC2 |
| hsa-mir-375 | ANKRD1 |
| hsa-mir-375 | SERP1 |
| hsa-mir-375 | SESN1 |
| hsa-mir-375 | DESI1 |
| hsa-mir-375 | MAT2B |
| hsa-mir-375 | C11orf54 |
| hsa-mir-375 | CCDC59 |
| hsa-mir-375 | SENP1 |
| hsa-mir-375 | NRBP1 |
| hsa-mir-375 | TMED5 |
| hsa-mir-375 | PHF20L1 |
| hsa-mir-375 | RDH11 |
| hsa-mir-375 | INSIG2 |
| hsa-mir-375 | JPT1 |
| hsa-mir-375 | NAGPA |
| hsa-mir-375 | NIN |
| hsa-mir-375 | CRIM1 |
| hsa-mir-375 | ZNF571 |
| hsa-mir-375 | CRLF3 |
| hsa-mir-375 | NELFCD |
| hsa-mir-375 | SCLY |
| hsa-mir-375 | RASD1 |
| hsa-mir-375 | CAB39 |
| hsa-mir-375 | NUP54 |
| hsa-mir-375 | GDAP1 |
| hsa-mir-375 | NLE1 |
| hsa-mir-375 | TMX3 |
| hsa-mir-375 | EXOC6 |
| hsa-mir-375 | MINDY2 |
| hsa-mir-375 | TMEM106B |
| hsa-mir-375 | NCAPG2 |
| hsa-mir-375 | NSD3 |
| hsa-mir-375 | PPP2R3C |
| hsa-mir-375 | RMDN3 |
| hsa-mir-375 | APPL2 |
| hsa-mir-375 | NAT10 |
| hsa-mir-375 | OGFOD1 |
| hsa-mir-375 | YY1AP1 |
| hsa-mir-375 | PGM2 |
| hsa-mir-375 | TMEM74B |
| hsa-mir-375 | AGPAT5 |
| hsa-mir-375 | MNS1 |
| hsa-mir-375 | STRBP |
| hsa-mir-375 | PIP4P2 |
| hsa-mir-375 | CDCA7L |
| hsa-mir-375 | VEZT |
| hsa-mir-375 | STAP2 |
| hsa-mir-375 | G2E3 |
| hsa-mir-375 | MREG |
| hsa-mir-375 | CCDC88A |
| hsa-mir-375 | ENAH |
| hsa-mir-375 | METTL2B |
| hsa-mir-375 | EMC3 |
| hsa-mir-375 | CMAS |
| hsa-mir-375 | NXT2 |
| hsa-mir-375 | NSFL1C |
| hsa-mir-375 | KLHL7 |
| hsa-mir-375 | PDGFC |
| hsa-mir-375 | ZC3HAV1 |
| hsa-mir-375 | BDH2 |
| hsa-mir-375 | IFT46 |
| hsa-mir-375 | C1GALT1 |
| hsa-mir-375 | ARNTL2 |
| hsa-mir-375 | CHPT1 |
| hsa-mir-375 | PHTF2 |
| hsa-mir-375 | KIAA1191 |
| hsa-mir-375 | PBXIP1 |
| hsa-mir-375 | CYP20A1 |
| hsa-mir-375 | USP31 |
| hsa-mir-375 | NUFIP2 |
| hsa-mir-375 | IFT80 |
| hsa-mir-375 | CIP2A |
| hsa-mir-375 | GATAD1 |
| hsa-mir-375 | RAP2C |
| hsa-mir-375 | ENOPH1 |
| hsa-mir-375 | ABHD4 |
| hsa-mir-375 | RRAGC |
| hsa-mir-375 | AZI2 |
| hsa-mir-375 | MPP5 |
| hsa-mir-375 | MOSPD3 |
| hsa-mir-375 | TNS3 |
| hsa-mir-375 | DEPTOR |
| hsa-mir-375 | USP46 |
| hsa-mir-375 | PLEKHA3 |
| hsa-mir-375 | MAPKAP1 |
| hsa-mir-375 | CHPF |
| hsa-mir-375 | ADIPOR2 |
| hsa-mir-375 | PPCS |
| hsa-mir-375 | BBS10 |
| hsa-mir-375 | ZDHHC11 |
| hsa-mir-375 | LPCAT1 |
| hsa-mir-375 | WWC2 |
| hsa-mir-375 | DGLUCY |
| hsa-mir-375 | GPR157 |
| hsa-mir-375 | ZNF614 |
| hsa-mir-375 | UXS1 |
| hsa-mir-375 | GKAP1 |
| hsa-mir-375 | CEP70 |
| hsa-mir-375 | LIMD2 |
| hsa-mir-375 | CEP44 |
| hsa-mir-375 | ZFP91 |
| hsa-mir-375 | SETD7 |
| hsa-mir-375 | NETO2 |
| hsa-mir-375 | APH1B |
| hsa-mir-375 | SYT15 |
| hsa-mir-375 | TAF3 |
| hsa-mir-375 | SNX25 |
| hsa-mir-375 | KCTD10 |
| hsa-mir-375 | FAM96A |
| hsa-mir-375 | FYTTD1 |
| hsa-mir-375 | CCDC115 |
| hsa-mir-375 | PCGF5 |
| hsa-mir-375 | GFM2 |
| hsa-mir-375 | MAP3K21 |
| hsa-mir-375 | DCTN5 |
| hsa-mir-375 | C15orf41 |
| hsa-mir-375 | EBPL |
| hsa-mir-375 | TMEM25 |
| hsa-mir-375 | RPUSD4 |
| hsa-mir-375 | TMTC4 |
| hsa-mir-375 | SLC35B4 |
| hsa-mir-375 | ATOH8 |
| hsa-mir-375 | CEP19 |
| hsa-mir-375 | PAQR8 |
| hsa-mir-375 | ZFHX2 |
| hsa-mir-375 | DISP2 |
| hsa-mir-375 | CCDC32 |
| hsa-mir-375 | STPG1 |
| hsa-mir-375 | ZNF799 |
| hsa-mir-375 | C12orf29 |
| hsa-mir-375 | OXNAD1 |
| hsa-mir-375 | MTDH |
| hsa-mir-375 | TRMT10A |
| hsa-mir-375 | MED8 |
| hsa-mir-375 | CYP2U1 |
| hsa-mir-375 | PIK3IP1 |
| hsa-mir-375 | MAL2 |
| hsa-mir-375 | OSBPL8 |
| hsa-mir-375 | PTPMT1 |
| hsa-mir-375 | VASN |
| hsa-mir-375 | BATF2 |
| hsa-mir-375 | NUS1 |
| hsa-mir-375 | COX20 |
| hsa-mir-375 | AEBP2 |
| hsa-mir-375 | TMEM170A |
| hsa-mir-375 | LSM12 |
| hsa-mir-375 | TPRG1L |
| hsa-mir-375 | GALM |
| hsa-mir-375 | GRPEL2 |
| hsa-mir-375 | UBLCP1 |
| hsa-mir-375 | ADAT2 |
| hsa-mir-375 | NCOA7 |
| hsa-mir-375 | MTPN |
| hsa-mir-375 | WFDC6 |
| hsa-mir-375 | GSC |
| hsa-mir-375 | CEP128 |
| hsa-mir-375 | TTC7B |
| hsa-mir-375 | CMTM4 |
| hsa-mir-375 | ZNF785 |
| hsa-mir-375 | SAMD11 |
| hsa-mir-375 | CBWD2 |
| hsa-mir-375 | SGO2 |
| hsa-mir-375 | SH3D19 |
| hsa-mir-375 | VPS37D |
| hsa-mir-375 | ANKRD46 |
| hsa-mir-375 | SPRED1 |
| hsa-mir-375 | ZNF540 |
| hsa-mir-375 | TOR1AIP2 |
| hsa-mir-375 | SGMS2 |
| hsa-mir-375 | PAPD4 |
| hsa-mir-375 | KLHDC8B |
| hsa-mir-375 | SLC39A11 |
| hsa-mir-375 | REEP3 |
| hsa-mir-375 | UBN2 |
| hsa-mir-375 | BOD1L1 |
| hsa-mir-375 | AQP11 |
| hsa-mir-375 | POC1B |
| hsa-mir-375 | CCDC88B |
| hsa-mir-375 | TMED8 |
| hsa-mir-375 | TMEM145 |
| hsa-mir-375 | LYPD5 |
| hsa-mir-375 | SLC25A42 |
| hsa-mir-375 | FAM126B |
| hsa-mir-375 | PRRT3 |
| hsa-mir-375 | DOK7 |
| hsa-mir-375 | FAM200B |
| hsa-mir-375 | CEP57L1 |
| hsa-mir-375 | METTL2A |
| hsa-mir-375 | ESPNL |
| hsa-mir-375 | ZNF404 |
| hsa-mir-375 | SOX2-OT |
| hsa-mir-375 | SKA2 |
| hsa-mir-375 | SFT2D2 |
| hsa-mir-375 | FAM89A |
| hsa-mir-375 | MALAT1 |
| hsa-mir-375 | KMT5A |
| hsa-mir-375 | ZNF470 |
| hsa-mir-375 | MIR17HG |
| hsa-mir-375 | IDNK |
| hsa-mir-375 | CBWD3 |
| hsa-mir-375 | ZBTB8A |
| hsa-mir-375 | FAM196B |
| hsa-mir-326 | ACTB |
| hsa-mir-326 | ACTG1 |
| hsa-mir-326 | AKT1 |
| hsa-mir-326 | ANXA6 |
| hsa-mir-326 | AR |
| hsa-mir-326 | ABCC6 |
| hsa-mir-326 | CCND1 |
| hsa-mir-326 | BCL2L1 |
| hsa-mir-326 | BMP7 |
| hsa-mir-326 | C1R |
| hsa-mir-326 | CD9 |
| hsa-mir-326 | CLU |
| hsa-mir-326 | EPHB3 |
| hsa-mir-326 | ERBB2 |
| hsa-mir-326 | F9 |
| hsa-mir-326 | FASN |
| hsa-mir-326 | FGF1 |
| hsa-mir-326 | FPR1 |
| hsa-mir-326 | XRCC6 |
| hsa-mir-326 | GLI1 |
| hsa-mir-326 | GRK6 |
| hsa-mir-326 | GYS1 |
| hsa-mir-326 | HLA-C |
| hsa-mir-326 | HNRNPA1 |
| hsa-mir-326 | HSPA1B |
| hsa-mir-326 | IHH |
| hsa-mir-326 | INPPL1 |
| hsa-mir-326 | KLK2 |
| hsa-mir-326 | KPNA2 |
| hsa-mir-326 | KRAS |
| hsa-mir-326 | MAGEB4 |
| hsa-mir-326 | MAT2A |
| hsa-mir-326 | MAZ |
| hsa-mir-326 | MSH3 |
| hsa-mir-326 | NF2 |
| hsa-mir-326 | NOTCH1 |
| hsa-mir-326 | NOTCH2 |
| hsa-mir-326 | NUP98 |
| hsa-mir-326 | PBX2 |
| hsa-mir-326 | PDE3A |
| hsa-mir-326 | PDE1B |
| hsa-mir-326 | PDHA1 |
| hsa-mir-326 | PIGA |
| hsa-mir-326 | PKM |
| hsa-mir-326 | CTSA |
| hsa-mir-326 | PTPA |
| hsa-mir-326 | SMO |
| hsa-mir-326 | FSCN1 |
| hsa-mir-326 | SP1 |
| hsa-mir-326 | DYNLT1 |
| hsa-mir-326 | ZNF154 |
| hsa-mir-326 | CSDE1 |
| hsa-mir-326 | MAFK |
| hsa-mir-326 | HMGA2 |
| hsa-mir-326 | FAM193A |
| hsa-mir-326 | UBE4A |
| hsa-mir-326 | GOSR2 |
| hsa-mir-326 | THRAP3 |
| hsa-mir-326 | ABCF2 |
| hsa-mir-326 | DCAF7 |
| hsa-mir-326 | SRRM1 |
| hsa-mir-326 | PCGF3 |
| hsa-mir-326 | SPTLC1 |
| hsa-mir-326 | MTHFD2 |
| hsa-mir-326 | SLC27A4 |
| hsa-mir-326 | RPP14 |
| hsa-mir-326 | BVES |
| hsa-mir-326 | ERLIN2 |
| hsa-mir-326 | ATXN2L |
| hsa-mir-326 | SYNRG |
| hsa-mir-326 | MYH15 |
| hsa-mir-326 | PHF8 |
| hsa-mir-326 | PSD3 |
| hsa-mir-326 | SF3B3 |
| hsa-mir-326 | POFUT1 |
| hsa-mir-326 | ACAP2 |
| hsa-mir-326 | PATZ1 |
| hsa-mir-326 | SSBP2 |
| hsa-mir-326 | CIZ1 |
| hsa-mir-326 | RTL8A |
| hsa-mir-326 | TRPC4AP |
| hsa-mir-326 | CNNM4 |
| hsa-mir-326 | AGO1 |
| hsa-mir-326 | MOCS3 |
| hsa-mir-326 | NOB1 |
| hsa-mir-326 | CD274 |
| hsa-mir-326 | PEX5L |
| hsa-mir-326 | GDE1 |
| hsa-mir-326 | A4GALT |
| hsa-mir-326 | RBM47 |
| hsa-mir-326 | MED18 |
| hsa-mir-326 | BCAS3 |
| hsa-mir-326 | VPS13C |
| hsa-mir-326 | SLC47A1 |
| hsa-mir-326 | ASXL2 |
| hsa-mir-326 | NKRF |
| hsa-mir-326 | SORCS2 |
| hsa-mir-326 | EPG5 |
| hsa-mir-326 | BCL11B |
| hsa-mir-326 | VKORC1 |
| hsa-mir-326 | ULBP3 |
| hsa-mir-326 | DHX40 |
| hsa-mir-326 | TBL1XR1 |
| hsa-mir-326 | UCK1 |
| hsa-mir-326 | GRWD1 |
| hsa-mir-326 | ZNF394 |
| hsa-mir-326 | HOOK3 |
| hsa-mir-326 | FAM167B |
| hsa-mir-326 | LSM10 |
| hsa-mir-326 | PAQR8 |
| hsa-mir-326 | KIAA1671 |
| hsa-mir-326 | ARRDC1 |
| hsa-mir-326 | ZNF689 |
| hsa-mir-326 | NAA30 |
| hsa-mir-326 | USH1G |
| hsa-mir-326 | SEZ6 |
| hsa-mir-326 | RPTN |
| hsa-mir-326 | FAM216B |
| hsa-mir-326 | HNRNPA1L2 |
| hsa-mir-326 | FBXL16 |
| hsa-mir-326 | CCDC12 |
| hsa-mir-326 | DAB2IP |
| hsa-mir-326 | ZNF746 |
| hsa-mir-326 | METTL27 |
| hsa-mir-326 | JMJD1C |
| hsa-mir-326 | ARL5B |
| hsa-mir-326 | HNRNPUL2 |
| hsa-mir-326 | ZNRF2 |
| hsa-mir-326 | CALHM5 |
| hsa-mir-326 | PLA2G4F |
| hsa-mir-326 | RBM20 |
| hsa-mir-326 | STUM |
| hsa-mir-326 | ZNF772 |
| hsa-mir-326 | TOMM5 |
| hsa-mir-326 | KIAA0754 |
| hsa-mir-326 | CASTOR2 |
| hsa-mir-326 | HOTAIR |
| hsa-mir-326 | C8orf17 |
| hsa-mir-338-3p | ACVR1 |
| hsa-mir-338-3p | ADCY7 |
| hsa-mir-338-3p | GRK2 |
| hsa-mir-338-3p | ALOX5AP |
| hsa-mir-338-3p | CCND1 |
| hsa-mir-338-3p | CAPN5 |
| hsa-mir-338-3p | C8A |
| hsa-mir-338-3p | RUNX2 |
| hsa-mir-338-3p | CDH2 |
| hsa-mir-338-3p | EIF4EBP2 |
| hsa-mir-338-3p | FOS |
| hsa-mir-338-3p | FUT4 |
| hsa-mir-338-3p | GATA6 |
| hsa-mir-338-3p | GRIK3 |
| hsa-mir-338-3p | HIF1A |
| hsa-mir-338-3p | MNX1 |
| hsa-mir-338-3p | HRH1 |
| hsa-mir-338-3p | DNAJB2 |
| hsa-mir-338-3p | ID3 |
| hsa-mir-338-3p | ITGB3 |
| hsa-mir-338-3p | KIFC1 |
| hsa-mir-338-3p | MAK |
| hsa-mir-338-3p | MAP1A |
| hsa-mir-338-3p | MID1 |
| hsa-mir-338-3p | MMP2 |
| hsa-mir-338-3p | MMP9 |
| hsa-mir-338-3p | MSN |
| hsa-mir-338-3p | MVK |
| hsa-mir-338-3p | MYH9 |
| hsa-mir-338-3p | NOVA1 |
| hsa-mir-338-3p | ORC4 |
| hsa-mir-338-3p | PCDH7 |
| hsa-mir-338-3p | PFAS |
| hsa-mir-338-3p | PKLR |
| hsa-mir-338-3p | PKM |
| hsa-mir-338-3p | PLXNA2 |
| hsa-mir-338-3p | RPL35A |
| hsa-mir-338-3p | RPL41 |
| hsa-mir-338-3p | VPS52 |
| hsa-mir-338-3p | SALL1 |
| hsa-mir-338-3p | SMO |
| hsa-mir-338-3p | SOX4 |
| hsa-mir-338-3p | SSR3 |
| hsa-mir-338-3p | ADAM17 |
| hsa-mir-338-3p | TAPBP |
| hsa-mir-338-3p | TAZ |
| hsa-mir-338-3p | WNT9A |
| hsa-mir-338-3p | XRCC2 |
| hsa-mir-338-3p | ZNF208 |
| hsa-mir-338-3p | SLC7A5 |
| hsa-mir-338-3p | IRS2 |
| hsa-mir-338-3p | TRADD |
| hsa-mir-338-3p | NRP1 |
| hsa-mir-338-3p | HIST1H2BJ |
| hsa-mir-338-3p | RNF8 |
| hsa-mir-338-3p | GPRC5A |
| hsa-mir-338-3p | SLC7A7 |
| hsa-mir-338-3p | EMC2 |
| hsa-mir-338-3p | FAM131B |
| hsa-mir-338-3p | KIAA0513 |
| hsa-mir-338-3p | ZEB2 |
| hsa-mir-338-3p | LRIG2 |
| hsa-mir-338-3p | ZBTB39 |
| hsa-mir-338-3p | TOM1 |
| hsa-mir-338-3p | ARL4C |
| hsa-mir-338-3p | SCML2 |
| hsa-mir-338-3p | ZBTB18 |
| hsa-mir-338-3p | STK25 |
| hsa-mir-338-3p | MORF4L1 |
| hsa-mir-338-3p | ZWINT |
| hsa-mir-338-3p | KIAA0895 |
| hsa-mir-338-3p | NCS1 |
| hsa-mir-338-3p | PHF3 |
| hsa-mir-338-3p | ACOT9 |
| hsa-mir-338-3p | FJX1 |
| hsa-mir-338-3p | ANAPC13 |
| hsa-mir-338-3p | GPR132 |
| hsa-mir-338-3p | ZBTB7B |
| hsa-mir-338-3p | RLIM |
| hsa-mir-338-3p | RSRC1 |
| hsa-mir-338-3p | SCARA3 |
| hsa-mir-338-3p | PHF7 |
| hsa-mir-338-3p | MRPS23 |
| hsa-mir-338-3p | C9orf78 |
| hsa-mir-338-3p | MTMR12 |
| hsa-mir-338-3p | SGTB |
| hsa-mir-338-3p | WDR55 |
| hsa-mir-338-3p | RPP25 |
| hsa-mir-338-3p | SDHAF2 |
| hsa-mir-338-3p | TMEM74B |
| hsa-mir-338-3p | UBE2Q1 |
| hsa-mir-338-3p | TENM3 |
| hsa-mir-338-3p | CDC42SE1 |
| hsa-mir-338-3p | LRRC47 |
| hsa-mir-338-3p | KIAA1210 |
| hsa-mir-338-3p | SHROOM3 |
| hsa-mir-338-3p | MIER1 |
| hsa-mir-338-3p | HES4 |
| hsa-mir-338-3p | PLEKHA1 |
| hsa-mir-338-3p | ARHGEF28 |
| hsa-mir-338-3p | CCDC14 |
| hsa-mir-338-3p | ALG9 |
| hsa-mir-338-3p | ATAT1 |
| hsa-mir-338-3p | PREX2 |
| hsa-mir-338-3p | ZKSCAN3 |
| hsa-mir-338-3p | ZFP91 |
| hsa-mir-338-3p | TRAF7 |
| hsa-mir-338-3p | ZDHHC18 |
| hsa-mir-338-3p | MRPL45 |
| hsa-mir-338-3p | LCOR |
| hsa-mir-338-3p | LMNB2 |
| hsa-mir-338-3p | ZNF566 |
| hsa-mir-338-3p | DSEL |
| hsa-mir-338-3p | BTBD9 |
| hsa-mir-338-3p | C1QTNF6 |
| hsa-mir-338-3p | GPR146 |
| hsa-mir-338-3p | SSX2IP |
| hsa-mir-338-3p | RFTN2 |
| hsa-mir-338-3p | B3GAT2 |
| hsa-mir-338-3p | BEST3 |
| hsa-mir-338-3p | TMEM199 |
| hsa-mir-338-3p | ZNF582 |
| hsa-mir-338-3p | DAB2IP |
| hsa-mir-338-3p | RNF217 |
| hsa-mir-338-3p | ZNF626 |
| hsa-mir-338-3p | TET3 |
| hsa-mir-338-3p | SLC25A34 |
| hsa-mir-338-3p | NLRP9 |
| hsa-mir-338-3p | MYLK4 |
| hsa-mir-338-3p | MACC1 |
| hsa-mir-338-3p | NUP43 |
| hsa-mir-338-3p | CATSPER4 |
| hsa-mir-338-3p | C15orf52 |
| hsa-mir-338-3p | SCIMP |
| hsa-mir-338-3p | C2orf71 |
| hsa-mir-338-3p | PLA2G2C |
| hsa-mir-338-3p | VHLL |
| hsa-mir-338-3p | POLR2J3 |
| hsa-mir-338-3p | ISPD |
| hsa-mir-338-3p | UPK3BL1 |
| hsa-mir-338-3p | PLA2G4B |
| hsa-mir-338-3p | SLFN12L |
