# Supplementary material for: Danggui Buxue Decoction Ameliorates Idiopathic Pulmonary Fibrosis through MicroRNA and Messenger RNA Regulatory Network
Source: Evid Based Complement Alternat Med. 2022 Apr 26;2022:3439656. doi: 10.1155/2022/3439656 (PMC9064538; doi:10.1155/2022/3439656)
Supplement: Supplementary Materials — Table S1: DGBXD granules. Table S2: Szapiel score system. Table S3: Ashcroft score system. Table S4: predicted target genes of upregulated DE-miRNAs (n = 1285). Table S5: predicted target genes of downregulated DE-miRNAs (n = 1411). Table S6: upregulated DE-mRNAs (n = 1160). Table S7: downregulated DE-mRNAs (n = 1427). Table S8: corresponding gene symbols of RA and RAS. [file 3439656.f1.zip › 3439656.f1/Table S6 Upregulated DE-mRNAs (n=1160).docx]

**Table S6:** Upregulated DE-mRNAs (n=1160).

|  | **logFC** | ***p-value*** | **adj *p-value*** |
| --- | --- | --- | --- |
| MMP1 | 7.702035 | 2.52E-13 | 1.27E-10 |
| KRT6A | 6.885773 | 4.73E-11 | 5.93E-09 |
| MMP10 | 6.136902 | 4.82E-13 | 1.88E-10 |
| KRT14 | 5.917626 | 3.79E-09 | 1.88E-07 |
| BPIFA1 | 5.862133 | 1.56E-06 | 2.51E-05 |
| MMP13 | 5.848486 | 7.56E-12 | 1.56E-09 |
| GREM1 | 5.717778 | 6.27E-12 | 1.43E-09 |
| COMP | 5.319064 | 4.13E-12 | 1.07E-09 |
| SERPINB4 | 4.936635 | 4.82E-07 | 9.83E-06 |
| CYP24A1 | 4.798529 | 6.57E-09 | 2.93E-07 |
| SERPINB3 | 4.78048 | 2.2E-05 | 0.000211 |
| BPIFB1 | 4.72194 | 6.36E-06 | 7.71E-05 |
| LY6D | 4.700063 | 6.63E-06 | 7.95E-05 |
| SPP1 | 4.694325 | 2.08E-10 | 1.85E-08 |
| COL17A1 | 4.661463 | 2.55E-16 | 1.18E-12 |
| KRT5 | 4.652137 | 2.65E-07 | 6.01E-06 |
| CXCL13 | 4.643006 | 3.64E-08 | 1.21E-06 |
| IGFL2 | 4.57212 | 5.2E-10 | 3.88E-08 |
| KRT16 | 4.543153 | 6.27E-07 | 1.21E-05 |
| SERPINB5 | 4.468365 | 2.65E-08 | 9.31E-07 |
| FDCSP | 4.441239 | 9.83E-07 | 1.74E-05 |
| ELK2AP | 4.359652 | 5.67E-08 | 1.74E-06 |
| CST2 | 4.34868 | 5.08E-10 | 3.82E-08 |
| CXCL6 | 4.226977 | 3.42E-08 | 1.16E-06 |
| TUBB3 | 4.215158 | 4.09E-11 | 5.25E-09 |
| IL13RA2 | 4.21388 | 1.06E-12 | 3.55E-10 |
| CILP2 | 4.200223 | 2.24E-09 | 1.24E-07 |
| MSMB | 4.195883 | 0.000318 | 0.001887 |
| SAA1 | 4.14469 | 3.52E-08 | 1.19E-06 |
| UGT1A6 | 4.12976 | 1.94E-10 | 1.74E-08 |
| PRSS1 | 4.121742 | 7.87E-08 | 2.23E-06 |
| GPR87 | 4.067992 | 5.81E-09 | 2.65E-07 |
| PLA2G2A | 4.05101 | 1.51E-08 | 5.79E-07 |
| TMPRSS4 | 4.033356 | 7.16E-08 | 2.09E-06 |
| DSC3 | 4.028085 | 1.8E-07 | 4.38E-06 |
| MMP11 | 4.007583 | 1.1E-12 | 3.64E-10 |
| MUC5B | 4.004812 | 4.28E-07 | 8.9E-06 |
| SAA2 | 3.969456 | 1.33E-08 | 5.23E-07 |
| CLCA2 | 3.898911 | 1.89E-07 | 4.53E-06 |
| FAM83A | 3.878067 | 3.6E-09 | 1.8E-07 |
| PCP4 | 3.872828 | 3.47E-10 | 2.73E-08 |
| CR2 | 3.861486 | 1.67E-06 | 2.65E-05 |
| GJB2 | 3.828773 | 7.29E-13 | 2.6E-10 |
| ADAMTS16 | 3.783741 | 1.4E-08 | 5.41E-07 |
| S100A2 | 3.740874 | 6.96E-10 | 4.88E-08 |
| COL10A1 | 3.728037 | 1.7E-12 | 5.15E-10 |
| CRLF1 | 3.701916 | 2.89E-13 | 1.38E-10 |
| LBP | 3.694626 | 2.25E-06 | 3.36E-05 |
| MMP7 | 3.691807 | 1.49E-11 | 2.41E-09 |
| KRT17 | 3.673967 | 1.46E-10 | 1.38E-08 |
| DPEP1 | 3.670049 | 8.36E-08 | 2.32E-06 |
| TMEM59L | 3.645836 | 1.94E-14 | 2.31E-11 |
| ABCA12 | 3.604705 | 1.12E-11 | 2.01E-09 |
| DIO2 | 3.59565 | 2.58E-09 | 1.39E-07 |
| SYT12 | 3.588954 | 2.9E-11 | 3.87E-09 |
| STRA6 | 3.585127 | 1.54E-11 | 2.44E-09 |
| PI15 | 3.570922 | 9.93E-08 | 2.67E-06 |
| ATP10B | 3.469522 | 2.48E-10 | 2.09E-08 |
| CXCL14 | 3.461254 | 2.63E-12 | 7.62E-10 |
| MUC16 | 3.432273 | 0.000233 | 0.001454 |
| B3GNT3 | 3.42389 | 2.74E-08 | 9.58E-07 |
| TNS4 | 3.412615 | 1.5E-10 | 1.4E-08 |
| BAAT | 3.404376 | 3.13E-13 | 1.43E-10 |
| MC4R | 3.388156 | 5.24E-06 | 6.62E-05 |
| SFRP2 | 3.368841 | 1.59E-07 | 3.98E-06 |
| FLJ41200 | 3.350644 | 1.98E-06 | 3.01E-05 |
| COL1A1 | 3.344216 | 3.04E-10 | 2.47E-08 |
| PSCA | 3.328824 | 1.07E-05 | 0.000116 |
| GLB1L3 | 3.328137 | 1.33E-08 | 5.23E-07 |
| CPXM1 | 3.311035 | 3.08E-08 | 1.05E-06 |
| FAT2 | 3.275534 | 6.05E-09 | 2.75E-07 |
| COL3A1 | 3.262927 | 7.58E-11 | 8.43E-09 |
| TCN1 | 3.250105 | 1.81E-06 | 2.81E-05 |
| CTHRC1 | 3.229875 | 5.46E-11 | 6.57E-09 |
| TRY6 | 3.2255 | 0.0132 | 0.038636 |
| ATP12A | 3.225042 | 5.7E-05 | 0.000456 |
| KRT15 | 3.208868 | 7.51E-09 | 3.26E-07 |
| SERPINA5 | 3.202571 | 8.26E-10 | 5.55E-08 |
| C13orf33 | 3.174768 | 6.33E-06 | 7.68E-05 |
| BPIFB2 | 3.167065 | 0.00029 | 0.001744 |
| ADH7 | 3.151992 | 7.2E-06 | 8.49E-05 |
| IVL | 3.148959 | 2.48E-06 | 3.62E-05 |
| GCNT3 | 3.138399 | 6.42E-10 | 4.57E-08 |
| LCN2 | 3.134655 | 5.58E-08 | 1.72E-06 |
| SLC5A5 | 3.113232 | 1.09E-06 | 1.89E-05 |
| MMP3 | 3.103957 | 2.61E-07 | 5.96E-06 |
| SCRG1 | 3.066479 | 4.7E-10 | 3.58E-08 |
| KIF26B | 3.060056 | 1.08E-11 | 1.96E-09 |
| CYP19A1 | 3.050978 | 2.09E-07 | 4.92E-06 |
| BNC1 | 3.04204 | 9.61E-06 | 0.000107 |
| COL11A1 | 3.019348 | 7.98E-06 | 9.21E-05 |
| SERPINB13 | 3.01699 | 0.001742 | 0.007486 |
| TDO2 | 3.005934 | 3.28E-09 | 1.66E-07 |
| ACAN | 2.997172 | 4.56E-09 | 2.17E-07 |
| P4HA3 | 2.988465 | 1.45E-10 | 1.38E-08 |
| CHRDL2 | 2.988057 | 8.54E-11 | 9.16E-09 |
| FAM83B | 2.985996 | 1.28E-07 | 3.33E-06 |
| CCL7 | 2.981237 | 4.08E-06 | 5.44E-05 |
| GAP43 | 2.976775 | 5.16E-11 | 6.33E-09 |
| C10orf81 | 2.967456 | 9.89E-05 | 0.000716 |
| CDH3 | 2.966124 | 4.59E-14 | 3.79E-11 |
| RHOV | 2.958208 | 4.35E-06 | 5.73E-05 |
| MUC13 | 2.950838 | 5.06E-05 | 0.000414 |
| CLDN10 | 2.949245 | 9.59E-10 | 6.27E-08 |
| CHST6 | 2.922565 | 1.97E-06 | 3E-05 |
| THY1 | 2.917746 | 3.29E-09 | 1.66E-07 |
| PCSK1 | 2.903921 | 2.74E-10 | 2.26E-08 |
| WT1 | 2.899119 | 0.000272 | 0.001659 |
| IGFL1 | 2.881617 | 0.000391 | 0.002229 |
| CHP2 | 2.85851 | 0.001107 | 0.005182 |
| CRABP1 | 2.855394 | 1.8E-05 | 0.000179 |
| KCNS2 | 2.854443 | 1.1E-07 | 2.9E-06 |
| PLXNA4 | 2.839236 | 9.71E-12 | 1.84E-09 |
| ADAMTS14 | 2.83721 | 7.74E-11 | 8.51E-09 |
| TMEM215 | 2.833643 | 7.91E-08 | 2.23E-06 |
| FCRL5 | 2.824241 | 6.36E-09 | 2.87E-07 |
| XPNPEP2 | 2.801582 | 1.79E-11 | 2.74E-09 |
| DSG3 | 2.799716 | 1.7E-07 | 4.19E-06 |
| VTCN1 | 2.787578 | 1.1E-05 | 0.000118 |
| IL11 | 2.778562 | 7.96E-07 | 1.47E-05 |
| NGFR | 2.75989 | 3.78E-10 | 2.94E-08 |
| MIR31HG | 2.759238 | 8.9E-07 | 1.61E-05 |
| TWIST1 | 2.748673 | 1.08E-07 | 2.86E-06 |
| POSTN | 2.747578 | 4.49E-08 | 1.43E-06 |
| PAPPA | 2.743388 | 8.23E-08 | 2.3E-06 |
| ADAMTS18 | 2.733757 | 1E-05 | 0.00011 |
| CD177 | 2.727219 | 4.52E-07 | 9.3E-06 |
| LGALS7B | 2.722697 | 8.85E-05 | 0.000655 |
| NPTX2 | 2.720615 | 4.6E-09 | 2.19E-07 |
| POU2AF1 | 2.717468 | 6.11E-08 | 1.82E-06 |
| FLJ16779 | 2.712996 | 3.54E-08 | 1.19E-06 |
| PROM1 | 2.712909 | 5.18E-05 | 0.000422 |
| GABBR2 | 2.710582 | 2.09E-06 | 3.15E-05 |
| GDA | 2.707598 | 1.39E-08 | 5.4E-07 |
| CACNA1G | 2.697676 | 5.9E-08 | 1.78E-06 |
| KRT6B | 2.693849 | 0.003021 | 0.011709 |
| PITX1 | 2.691156 | 0.000204 | 0.0013 |
| CILP | 2.685633 | 2.81E-10 | 2.3E-08 |
| GSTA2 | 2.67239 | 0.005195 | 0.01826 |
| KRT13 | 2.664424 | 4.75E-06 | 6.12E-05 |
| CLEC4G | 2.655542 | 5.08E-08 | 1.59E-06 |
| TFAP2A | 2.645863 | 2.22E-07 | 5.15E-06 |
| IGLL5 | 2.644604 | 1.32E-06 | 2.2E-05 |
| TIMP4 | 2.64455 | 9.82E-11 | 1.02E-08 |
| THBS2 | 2.63818 | 8.03E-10 | 5.45E-08 |
| GJB5 | 2.633869 | 6.44E-06 | 7.78E-05 |
| LOC100505989 | 2.620817 | 2.85E-05 | 0.00026 |
| HS6ST2 | 2.620455 | 7.72E-10 | 5.31E-08 |
| ADAM12 | 2.617502 | 3.88E-09 | 1.91E-07 |
| ZNF385D | 2.615689 | 1.63E-08 | 6.17E-07 |
| CD79A | 2.606772 | 1.14E-06 | 1.95E-05 |
| COL14A1 | 2.603623 | 7.76E-10 | 5.31E-08 |
| KRT23 | 2.601352 | 2.38E-05 | 0.000224 |
| MYO3B | 2.59837 | 5.79E-11 | 6.93E-09 |
| UCN2 | 2.596595 | 5.36E-08 | 1.66E-06 |
| LRRC26 | 2.59181 | 8.08E-06 | 9.3E-05 |
| UPK1B | 2.587857 | 0.000318 | 0.001887 |
| STEAP1 | 2.587685 | 1.91E-10 | 1.72E-08 |
| MIR205HG | 2.587393 | 6.65E-05 | 0.000516 |
| VPREB3 | 2.586799 | 5.89E-06 | 7.26E-05 |
| LGI2 | 2.585714 | 6.74E-12 | 1.47E-09 |
| CPNE4 | 2.584401 | 2.64E-10 | 2.2E-08 |
| PSAT1 | 2.581496 | 3.23E-12 | 8.99E-10 |
| ECEL1 | 2.577692 | 4.01E-06 | 5.37E-05 |
| DNAJC22 | 2.571693 | 1.38E-08 | 5.39E-07 |
| MUC4 | 2.570485 | 5.47E-07 | 1.08E-05 |
| HAMP | 2.569769 | 4.87E-08 | 1.54E-06 |
| IGF1 | 2.553948 | 9.55E-10 | 6.26E-08 |
| SCG5 | 2.546063 | 2.49E-08 | 8.9E-07 |
| TNFRSF13C | 2.544637 | 1.86E-07 | 4.48E-06 |
| MZB1 | 2.544494 | 1.31E-06 | 2.19E-05 |
| FCRLA | 2.541953 | 2.37E-06 | 3.49E-05 |
| MCHR1 | 2.539102 | 4.08E-08 | 1.34E-06 |
| FNDC1 | 2.536665 | 2.11E-06 | 3.18E-05 |
| MMP12 | 2.523319 | 4.92E-05 | 0.000404 |
| GLYATL2 | 2.521238 | 2.6E-05 | 0.000241 |
| TMEM229A | 2.51538 | 1.53E-08 | 5.84E-07 |
| CCNO | 2.51433 | 0.000799 | 0.003983 |
| CDH2 | 2.50543 | 1.97E-06 | 3E-05 |
| FRMD5 | 2.495961 | 4.83E-12 | 1.2E-09 |
| GJB4 | 2.483829 | 1.23E-05 | 0.00013 |
| NETO1 | 2.478466 | 1.59E-05 | 0.000162 |
| PADI1 | 2.475648 | 7.07E-05 | 0.000544 |
| FAM83F | 2.470101 | 3.23E-06 | 4.5E-05 |
| ADAM23 | 2.469384 | 7.65E-08 | 2.17E-06 |
| ERN2 | 2.468946 | 0.00052 | 0.002823 |
| FOXE1 | 2.465495 | 2.99E-06 | 4.21E-05 |
| CBLN4 | 2.458218 | 5.31E-06 | 6.69E-05 |
| PTPRZ1 | 2.454182 | 2.87E-08 | 9.91E-07 |
| MMP8 | 2.453521 | 0.001085 | 0.005098 |
| LTF | 2.449719 | 2.19E-05 | 0.000209 |
| F2RL2 | 2.437665 | 7.18E-09 | 3.15E-07 |
| ANKRD18B | 2.437292 | 3.1E-05 | 0.000278 |
| BHLHA15 | 2.424675 | 8.01E-08 | 2.25E-06 |
| JSRP1 | 2.420554 | 1.71E-05 | 0.000172 |
| FUT2 | 2.412386 | 4.51E-09 | 2.16E-07 |
| ABP1 | 2.392178 | 5.71E-08 | 1.75E-06 |
| COL15A1 | 2.382922 | 1.99E-09 | 1.13E-07 |
| SERPIND1 | 2.376327 | 7.94E-05 | 0.0006 |
| OTX1 | 2.371 | 1.7E-05 | 0.000171 |
| HTR2A | 2.368859 | 1.6E-07 | 4E-06 |
| CRABP2 | 2.362555 | 3.09E-09 | 1.59E-07 |
| AKR1B10 | 2.344343 | 0.000229 | 0.001433 |
| SAA2-SAA4 | 2.339943 | 0.000322 | 0.001903 |
| SIX2 | 2.33841 | 3.94E-05 | 0.000338 |
| DOK5 | 2.335541 | 1.18E-08 | 4.8E-07 |
| C1QL2 | 2.334164 | 1.92E-05 | 0.000188 |
| CP | 2.319084 | 1.35E-06 | 2.24E-05 |
| OGDHL | 2.317791 | 5.11E-07 | 1.03E-05 |
| LYPD1 | 2.315752 | 3.59E-12 | 9.62E-10 |
| MIR650 | 2.314858 | 0.000884 | 0.004317 |
| WISP1 | 2.314086 | 5.76E-08 | 1.75E-06 |
| LOC646862 | 2.310113 | 6.48E-05 | 0.000507 |
| ADM2 | 2.308764 | 7.07E-09 | 3.11E-07 |
| SERPINA3 | 2.304391 | 7.87E-07 | 1.46E-05 |
| ACTG2 | 2.300033 | 1.34E-06 | 2.22E-05 |
| DRD5 | 2.298251 | 5.58E-06 | 6.97E-05 |
| BBOX1 | 2.297652 | 4.96E-06 | 6.35E-05 |
| DEFB4A | 2.295976 | 0.001809 | 0.007702 |
| LOC100131726 | 2.295905 | 4.31E-06 | 5.7E-05 |
| ALPK2 | 2.289971 | 8.03E-07 | 1.48E-05 |
| SPRR2D | 2.288247 | 0.000294 | 0.001763 |
| SERPINB2 | 2.288163 | 0.000293 | 0.00176 |
| KIAA1644 | 2.282738 | 1.54E-07 | 3.87E-06 |
| TSPAN19 | 2.278172 | 0.004212 | 0.015401 |
| MMP9 | 2.269669 | 1.65E-06 | 2.62E-05 |
| KLK12 | 2.261649 | 3.96E-05 | 0.000339 |
| FUT6 | 2.259169 | 5.74E-06 | 7.11E-05 |
| CYP1B1 | 2.256871 | 5.24E-07 | 1.05E-05 |
| ITGA11 | 2.251523 | 1.19E-07 | 3.11E-06 |
| MYEOV | 2.242389 | 5.83E-08 | 1.77E-06 |
| SHOX2 | 2.241931 | 2.7E-06 | 3.87E-05 |
| ACTBL2 | 2.237717 | 5.28E-07 | 1.05E-05 |
| GALNTL2 | 2.235051 | 4.94E-06 | 6.33E-05 |
| ADAMTS4 | 2.226104 | 0.004165 | 0.015247 |
| PDLIM4 | 2.225817 | 3.88E-08 | 1.28E-06 |
| SLC2A5 | 2.223204 | 1.51E-08 | 5.77E-07 |
| ADIPOQ | 2.220557 | 0.01254 | 0.037121 |
| FLNC | 2.213828 | 1.7E-06 | 2.68E-05 |
| HABP2 | 2.211827 | 0.000127 | 0.000881 |
| TMEM200C | 2.197276 | 1.32E-08 | 5.21E-07 |
| BHLHE22 | 2.194655 | 1.34E-09 | 8.14E-08 |
| FHL2 | 2.184101 | 1.47E-10 | 1.38E-08 |
| SHISA8 | 2.183541 | 1.89E-05 | 0.000186 |
| SPINK1 | 2.182379 | 3.93E-06 | 5.29E-05 |
| GSTA1 | 2.18156 | 0.001629 | 0.007072 |
| CHI3L1 | 2.177064 | 3.4E-07 | 7.3E-06 |
| CGREF1 | 2.175346 | 8.5E-07 | 1.55E-05 |
| AMTN | 2.174748 | 0.006247 | 0.02119 |
| PTX3 | 2.173785 | 0.001133 | 0.005281 |
| DERL3 | 2.171219 | 7.13E-07 | 1.34E-05 |
| SULF1 | 2.167198 | 3.54E-08 | 1.19E-06 |
| CYP2A13 | 2.164676 | 0.000416 | 0.002337 |
| KRT75 | 2.156144 | 0.000184 | 0.00119 |
| TUBB2B | 2.155424 | 1.28E-08 | 5.09E-07 |
| CALML3 | 2.14577 | 0.002152 | 0.008898 |
| CKMT1B | 2.145601 | 2.23E-05 | 0.000213 |
| CACNA1E | 2.14439 | 4.76E-06 | 6.12E-05 |
| SERPINB7 | 2.143051 | 0.003187 | 0.012233 |
| MB | 2.137155 | 0.000692 | 0.003551 |
| FBLN2 | 2.134388 | 9.4E-08 | 2.55E-06 |
| ANKUB1 | 2.13216 | 0.005194 | 0.01826 |
| RAB3C | 2.132123 | 1.2E-08 | 4.83E-07 |
| FAM83D | 2.130844 | 5.36E-07 | 1.07E-05 |
| CYP2F1 | 2.128917 | 0.000937 | 0.00453 |
| TXNDC5 | 2.119449 | 1.09E-05 | 0.000118 |
| FAM150A | 2.117799 | 2.28E-06 | 3.4E-05 |
| DIRAS1 | 2.117291 | 5.35E-06 | 6.73E-05 |
| SDS | 2.115075 | 1.38E-06 | 2.27E-05 |
| C2CD4A | 2.113167 | 5.04E-05 | 0.000412 |
| CCL13 | 2.103166 | 3.62E-07 | 7.68E-06 |
| CD19 | 2.09958 | 4.96E-06 | 6.35E-05 |
| COL5A2 | 2.089429 | 4.54E-09 | 2.17E-07 |
| COL6A3 | 2.085288 | 3.17E-09 | 1.61E-07 |
| SLN | 2.082219 | 0.000156 | 0.001039 |
| PROM2 | 2.081147 | 4.39E-09 | 2.11E-07 |
| PNOC | 2.080661 | 1.16E-05 | 0.000124 |
| GJB6 | 2.079537 | 0.000321 | 0.001899 |
| CLMP | 2.079074 | 2.45E-08 | 8.77E-07 |
| PLA2G7 | 2.074264 | 6.89E-08 | 2.03E-06 |
| CLDN2 | 2.071301 | 4.45E-05 | 0.000375 |
| CLCA4 | 2.07046 | 0.000771 | 0.003865 |
| LOC401463 | 2.069295 | 2.15E-07 | 5.03E-06 |
| GPR115 | 2.068632 | 1.89E-05 | 0.000186 |
| CDC20B | 2.067588 | 0.017049 | 0.047456 |
| EPHA10 | 2.063287 | 1.61E-08 | 6.08E-07 |
| PAX9 | 2.060457 | 2.82E-05 | 0.000258 |
| KRT16P2 | 2.060229 | 0.005159 | 0.01816 |
| LOC100287814 | 2.055636 | 0.000144 | 0.000976 |
| ZPLD1 | 2.053696 | 0.003761 | 0.014058 |
| KCNG2 | 2.053143 | 9.9E-07 | 1.74E-05 |
| HECW1 | 2.044278 | 7.78E-07 | 1.44E-05 |
| STAC2 | 2.03956 | 1.77E-05 | 0.000177 |
| CYP27C1 | 2.039339 | 2.68E-07 | 6.06E-06 |
| EDN2 | 2.037182 | 0.001056 | 0.004977 |
| TEX26 | 2.036086 | 0.006378 | 0.021537 |
| C12orf74 | 2.033141 | 0.00076 | 0.003827 |
| PTPRT | 2.031113 | 0.001444 | 0.006426 |
| SERPINE2 | 2.022212 | 2.07E-07 | 4.89E-06 |
| VCAN | 2.017645 | 2.43E-08 | 8.72E-07 |
| PAX5 | 2.017186 | 0.000246 | 0.001523 |
| WDR38 | 2.015425 | 0.005356 | 0.018717 |
| HHLA2 | 2.014634 | 0.00013 | 0.000894 |
| SERPINF1 | 2.013548 | 1E-10 | 1.04E-08 |
| RAB3B | 2.012893 | 6.78E-06 | 8.09E-05 |
| CHST9 | 2.008477 | 0.010379 | 0.031899 |
| FAM155B | 2.006944 | 8.38E-06 | 9.56E-05 |
| C8orf47 | 2.00647 | 0.002188 | 0.009018 |
| MS4A1 | 2.005326 | 4.89E-05 | 0.000403 |
| TNC | 2.002107 | 2.56E-08 | 9.05E-07 |
| HMGA2 | 2.001261 | 0.000134 | 0.00092 |
| DNAH3 | 1.996986 | 0.003824 | 0.01424 |
| SLC5A8 | 1.996285 | 0.00032 | 0.001892 |
| MLIP | 1.994027 | 6.02E-06 | 7.36E-05 |
| COL1A2 | 1.991712 | 5.87E-08 | 1.77E-06 |
| ABCA13 | 1.987352 | 0.000784 | 0.003918 |
| CYMP | 1.98689 | 7.35E-06 | 8.62E-05 |
| VSIG1 | 1.983811 | 0.001882 | 0.007957 |
| PRG4 | 1.982382 | 1.65E-05 | 0.000168 |
| FRMPD2 | 1.980774 | 0.001174 | 0.00543 |
| LOC100499467 | 1.977601 | 1.68E-06 | 2.66E-05 |
| SLC22A16 | 1.976556 | 0.00215 | 0.008892 |
| ADAMDEC1 | 1.975632 | 0.000113 | 0.000799 |
| ALDH3A1 | 1.975279 | 1.01E-05 | 0.00011 |
| IGDCC4 | 1.974437 | 2.54E-07 | 5.82E-06 |
| MRO | 1.969757 | 1.02E-06 | 1.79E-05 |
| MEOX1 | 1.966424 | 0.000335 | 0.001966 |
| GBP6 | 1.966175 | 0.000122 | 0.00085 |
| SSTR2 | 1.964382 | 2.88E-05 | 0.000261 |
| ELOVL2 | 1.960507 | 2.88E-05 | 0.000262 |
| CCDC80 | 1.953839 | 6.73E-05 | 0.000521 |
| TP53AIP1 | 1.95036 | 0.00222 | 0.009127 |
| MMP16 | 1.949329 | 1.38E-06 | 2.27E-05 |
| TMEM190 | 1.948578 | 0.004911 | 0.017455 |
| TM4SF19 | 1.945374 | 0.00016 | 0.001058 |
| EYA2 | 1.939249 | 7.61E-08 | 2.16E-06 |
| CTSE | 1.93778 | 2.49E-07 | 5.72E-06 |
| LOC100288077 | 1.933766 | 2.86E-05 | 0.000261 |
| FAM43B | 1.933286 | 1.16E-05 | 0.000124 |
| HNF4G | 1.931665 | 0.000285 | 0.001718 |
| COL5A1 | 1.926029 | 4.61E-08 | 1.47E-06 |
| DNAH9 | 1.925217 | 0.004144 | 0.015193 |
| MEF2B | 1.924212 | 1.06E-05 | 0.000116 |
| TMEM45A | 1.923847 | 4.62E-08 | 1.47E-06 |
| BOC | 1.921254 | 6.62E-07 | 1.26E-05 |
| ST6GALNAC1 | 1.917471 | 4.23E-06 | 5.62E-05 |
| COL22A1 | 1.914788 | 4.94E-09 | 2.33E-07 |
| EPN3 | 1.912543 | 0.000192 | 0.001234 |
| VSTM2L | 1.912116 | 6.74E-07 | 1.27E-05 |
| CSMD2 | 1.907243 | 6.9E-08 | 2.03E-06 |
| MUCL1 | 1.906536 | 0.000499 | 0.002726 |
| KCND3 | 1.904006 | 4.55E-06 | 5.95E-05 |
| GPX2 | 1.902139 | 9.88E-07 | 1.74E-05 |
| LOC283710 | 1.901837 | 3.99E-06 | 5.35E-05 |
| DES | 1.899313 | 9.26E-06 | 0.000104 |
| UGT2A1 | 1.897994 | 0.006764 | 0.022583 |
| PPAPDC1A | 1.89762 | 2.12E-05 | 0.000204 |
| SPDEF | 1.895996 | 4.59E-06 | 5.97E-05 |
| TTR | 1.895159 | 0.003033 | 0.01174 |
| IGFBP5 | 1.891451 | 2.62E-08 | 9.23E-07 |
| PIP | 1.889451 | 0.007479 | 0.024509 |
| RARRES1 | 1.88924 | 7.94E-09 | 3.43E-07 |
| GPR110 | 1.885161 | 1.56E-06 | 2.51E-05 |
| WNK2 | 1.882582 | 2.49E-05 | 0.000233 |
| MAP1A | 1.880703 | 6.83E-07 | 1.29E-05 |
| STMN2 | 1.880528 | 8.15E-05 | 0.000614 |
| TSPAN1 | 1.878154 | 0.000217 | 0.001372 |
| SCGB3A1 | 1.873593 | 0.001396 | 0.006244 |
| TNFRSF6B | 1.87346 | 3.62E-05 | 0.000317 |
| PYCR1 | 1.871448 | 5.07E-10 | 3.82E-08 |
| ALDH1A3 | 1.870117 | 5.73E-08 | 1.75E-06 |
| VWA3B | 1.869622 | 0.008869 | 0.028163 |
| MIR3189 | 1.868661 | 0.000129 | 0.000893 |
| LOC100130899 | 1.867488 | 4.76E-05 | 0.000396 |
| EPHB2 | 1.860653 | 6.83E-10 | 4.81E-08 |
| ADCYAP1 | 1.856501 | 0.001493 | 0.006595 |
| UCN3 | 1.853939 | 0.001391 | 0.006228 |
| HCAR1 | 1.853875 | 0.000361 | 0.002091 |
| GDF15 | 1.849983 | 3.97E-07 | 8.35E-06 |
| ROR2 | 1.849861 | 8.29E-08 | 2.31E-06 |
| DGKI | 1.849341 | 8.01E-06 | 9.23E-05 |
| CCDC164 | 1.846775 | 0.010269 | 0.031649 |
| IL2RA | 1.846481 | 1.45E-06 | 2.37E-05 |
| ASPN | 1.84623 | 6.28E-07 | 1.21E-05 |
| CPNE5 | 1.845998 | 2E-07 | 4.73E-06 |
| SLCO5A1 | 1.845005 | 1.04E-06 | 1.82E-05 |
| LOC96610 | 1.841214 | 6.27E-06 | 7.63E-05 |
| ASB5 | 1.839912 | 0.000546 | 0.00293 |
| SPTBN2 | 1.839433 | 3.57E-09 | 1.79E-07 |
| HTR3A | 1.839343 | 0.000329 | 0.001934 |
| SCG2 | 1.839118 | 1.67E-06 | 2.65E-05 |
| PRSS3 | 1.838553 | 2.16E-05 | 0.000207 |
| BLK | 1.838251 | 1.05E-05 | 0.000115 |
| DRD2 | 1.833105 | 0.000541 | 0.002906 |
| GLIS1 | 1.830849 | 4.46E-05 | 0.000375 |
| USH1C | 1.829499 | 0.001529 | 0.006726 |
| SAA4 | 1.828855 | 0.002574 | 0.010299 |
| FAM159B | 1.823493 | 0.001113 | 0.005207 |
| TMEM132E | 1.820366 | 2.21E-06 | 3.3E-05 |
| CCR8 | 1.819372 | 6.83E-06 | 8.13E-05 |
| P2RY6 | 1.817373 | 5.72E-09 | 2.63E-07 |
| NCS1 | 1.814195 | 1.17E-07 | 3.07E-06 |
| PLD5 | 1.812756 | 1.87E-06 | 2.89E-05 |
| SH3PXD2B | 1.81049 | 2.78E-07 | 6.25E-06 |
| C19orf26 | 1.805486 | 0.000664 | 0.003441 |
| MTTP | 1.805431 | 9.43E-06 | 0.000105 |
| ARNTL2 | 1.804919 | 5.7E-09 | 2.63E-07 |
| LRRC15 | 1.804341 | 0.000105 | 0.000751 |
| MMP2 | 1.803476 | 1.82E-07 | 4.4E-06 |
| FGF11 | 1.797479 | 3.95E-12 | 1.03E-09 |
| IDAS | 1.797181 | 0.00116 | 0.005382 |
| SLC27A2 | 1.796577 | 5.93E-05 | 0.00047 |
| NKAIN4 | 1.794253 | 7.78E-06 | 9.05E-05 |
| TMEM158 | 1.793084 | 3.18E-06 | 4.44E-05 |
| DUSP9 | 1.789878 | 6.85E-05 | 0.00053 |
| SOX2 | 1.789696 | 0.010512 | 0.032227 |
| TNFRSF17 | 1.781764 | 0.000387 | 0.002213 |
| LUZP2 | 1.77843 | 0.00017 | 0.001117 |
| KIAA0125 | 1.775206 | 0.000223 | 0.001402 |
| TNFSF11 | 1.772952 | 1.08E-06 | 1.87E-05 |
| ADH4 | 1.771746 | 0.000142 | 0.000968 |
| MUC21 | 1.7694 | 2.29E-05 | 0.000217 |
| XIRP1 | 1.767511 | 0.006404 | 0.021611 |
| C9orf135 | 1.765461 | 0.012756 | 0.037622 |
| MEP1A | 1.765075 | 2.02E-06 | 3.06E-05 |
| PTGFRN | 1.764992 | 1.02E-10 | 1.05E-08 |
| C7orf10 | 1.7617 | 5.75E-09 | 2.63E-07 |
| MARCH4 | 1.761687 | 0.000844 | 0.004163 |
| LRRN1 | 1.756846 | 5.14E-09 | 2.41E-07 |
| WFDC2 | 1.754355 | 2.43E-06 | 3.56E-05 |
| C12orf36 | 1.753633 | 0.009517 | 0.029841 |
| KLK13 | 1.751308 | 0.001769 | 0.007573 |
| TSHR | 1.750706 | 3.63E-05 | 0.000317 |
| IL1R2 | 1.749762 | 0.000401 | 0.002275 |
| MS4A8B | 1.743965 | 0.015169 | 0.043257 |
| NTN1 | 1.741784 | 5.27E-07 | 1.05E-05 |
| C1orf189 | 1.741555 | 0.002903 | 0.011312 |
| COL7A1 | 1.738954 | 4.94E-08 | 1.56E-06 |
| LIPG | 1.737253 | 0.000592 | 0.003121 |
| HPSE2 | 1.737156 | 5.17E-07 | 1.04E-05 |
| LRRC10B | 1.73388 | 0.004153 | 0.015215 |
| PKP1 | 1.733601 | 0.000173 | 0.001129 |
| PCSK1N | 1.731207 | 6.9E-06 | 8.2E-05 |
| GABRA3 | 1.730935 | 0.003731 | 0.013977 |
| LOC100507127 | 1.728164 | 9.43E-05 | 0.000689 |
| SRD5A2 | 1.72651 | 0.011814 | 0.035399 |
| C1orf173 | 1.726194 | 0.010228 | 0.031568 |
| FER1L4 | 1.725228 | 5.69E-06 | 7.07E-05 |
| C9orf171 | 1.724015 | 0.006797 | 0.022667 |
| SSC5D | 1.723717 | 1.44E-07 | 3.66E-06 |
| TRIM29 | 1.722218 | 0.000183 | 0.001185 |
| SNAP25 | 1.722174 | 0.000107 | 0.000764 |
| PPAP2C | 1.720928 | 6.99E-05 | 0.00054 |
| SLC38A11 | 1.716919 | 1.59E-06 | 2.54E-05 |
| BFSP2 | 1.715652 | 8.42E-05 | 0.000629 |
| PODNL1 | 1.715352 | 4.43E-08 | 1.42E-06 |
| LINC00520 | 1.715122 | 0.00028 | 0.001696 |
| ISM1 | 1.713426 | 2.96E-09 | 1.55E-07 |
| MIR3687 | 1.712523 | 4.45E-05 | 0.000375 |
| SLC4A11 | 1.708882 | 5.56E-06 | 6.94E-05 |
| GABRP | 1.70837 | 0.000769 | 0.003861 |
| FERMT1 | 1.707946 | 7.87E-09 | 3.4E-07 |
| SAMD11 | 1.707566 | 3.55E-07 | 7.57E-06 |
| TIMP1 | 1.706756 | 7.11E-06 | 8.4E-05 |
| LOC100507254 | 1.706437 | 0.000481 | 0.002636 |
| ARMC3 | 1.706079 | 0.014613 | 0.041938 |
| MEX3A | 1.706056 | 1.02E-08 | 4.22E-07 |
| TGM5 | 1.705701 | 2.51E-05 | 0.000235 |
| SYT8 | 1.705115 | 0.000127 | 0.000881 |
| NEK10 | 1.70315 | 0.00399 | 0.014739 |
| IGSF9 | 1.701976 | 6.24E-06 | 7.6E-05 |
| CNN1 | 1.700191 | 2.52E-05 | 0.000235 |
| ODZ3 | 1.696865 | 9.84E-06 | 0.000109 |
| PALM2-AKAP2 | 1.695342 | 0.000654 | 0.003396 |
| GAL | 1.693422 | 0.005327 | 0.018633 |
| FNDC4 | 1.692917 | 2.85E-06 | 4.06E-05 |
| CTXN1 | 1.692114 | 0.000257 | 0.00158 |
| LDLRAD1 | 1.69075 | 0.012754 | 0.037621 |
| BAI2 | 1.690404 | 5.46E-08 | 1.69E-06 |
| ZNF469 | 1.690357 | 8.15E-09 | 3.49E-07 |
| GDNF | 1.688481 | 0.000508 | 0.002769 |
| RASAL1 | 1.687416 | 1.54E-07 | 3.87E-06 |
| HR | 1.684678 | 5.35E-08 | 1.66E-06 |
| NELL1 | 1.682426 | 0.008149 | 0.026251 |
| ANKRD36BP2 | 1.68221 | 0.000337 | 0.001976 |
| GRM7 | 1.681808 | 0.010162 | 0.031409 |
| FBN1 | 1.68139 | 1.65E-07 | 4.08E-06 |
| LOC100287846 | 1.681083 | 3.68E-06 | 5.03E-05 |
| ARHGAP40 | 1.678937 | 6.7E-05 | 0.000519 |
| SIX1 | 1.678915 | 0.001486 | 0.00657 |
| CD180 | 1.676334 | 4.24E-07 | 8.85E-06 |
| EPYC | 1.676091 | 0.000362 | 0.002095 |
| SLC7A5 | 1.674044 | 3.13E-05 | 0.00028 |
| TSHZ2 | 1.669501 | 1.14E-08 | 4.67E-07 |
| PGLYRP4 | 1.668201 | 0.00165 | 0.007141 |
| SLC38A8 | 1.660665 | 0.003154 | 0.012126 |
| SIX4 | 1.660649 | 7.04E-05 | 0.000543 |
| GRIA3 | 1.660315 | 8.12E-06 | 9.33E-05 |
| CFH | 1.659823 | 2.59E-08 | 9.15E-07 |
| PCSK2 | 1.658265 | 0.00033 | 0.001937 |
| KRT40 | 1.657054 | 0.002126 | 0.008803 |
| SPOCK1 | 1.651341 | 0.000231 | 0.001442 |
| LAMP5 | 1.649939 | 2.02E-08 | 7.45E-07 |
| TP63 | 1.649433 | 1.34E-05 | 0.00014 |
| PCDH7 | 1.648398 | 4.14E-07 | 8.67E-06 |
| GRM4 | 1.648089 | 0.000858 | 0.004219 |
| CAPN13 | 1.644773 | 7.25E-06 | 8.53E-05 |
| PHLDA2 | 1.639574 | 4.53E-05 | 0.00038 |
| CCNA1 | 1.639505 | 0.004306 | 0.015676 |
| PAPPA2 | 1.638433 | 0.000381 | 0.002187 |
| LOC100131208 | 1.638124 | 1.37E-05 | 0.000142 |
| FAM92B | 1.636585 | 0.016798 | 0.046897 |
| CERCAM | 1.636401 | 2.67E-07 | 6.06E-06 |
| FAIM2 | 1.632604 | 4.29E-06 | 5.68E-05 |
| MAP1B | 1.632436 | 1.27E-06 | 2.14E-05 |
| HIF1A | 1.629968 | 4.31E-06 | 5.7E-05 |
| INHBB | 1.629014 | 4.6E-06 | 5.99E-05 |
| GJB3 | 1.627704 | 0.000171 | 0.001121 |
| SEMA6B | 1.627378 | 3.05E-05 | 0.000274 |
| CD248 | 1.626913 | 8.31E-06 | 9.49E-05 |
| SRRM3 | 1.626302 | 2.71E-07 | 6.11E-06 |
| CNR2 | 1.625604 | 0.000662 | 0.003431 |
| BDKRB2 | 1.625413 | 2.69E-05 | 0.000248 |
| IFNE | 1.624881 | 0.000676 | 0.003484 |
| HS6ST3 | 1.624721 | 0.000729 | 0.003694 |
| BARX2 | 1.624574 | 0.000619 | 0.003243 |
| GSDMC | 1.624311 | 6.31E-06 | 7.67E-05 |
| ACCN2 | 1.623418 | 3.44E-07 | 7.37E-06 |
| HCN4 | 1.62208 | 0.002342 | 0.009547 |
| SLC24A2 | 1.622014 | 2.3E-05 | 0.000218 |
| C6orf222 | 1.62034 | 0.000113 | 0.0008 |
| CEACAM5 | 1.618119 | 0.002988 | 0.011595 |
| C5orf65 | 1.617807 | 1.76E-09 | 1.02E-07 |
| SALL4 | 1.614873 | 1.27E-05 | 0.000134 |
| KRT42P | 1.613358 | 0.0023 | 0.009407 |
| MIXL1 | 1.613192 | 0.000831 | 0.004106 |
| MOXD1 | 1.613038 | 2.27E-07 | 5.24E-06 |
| FOXJ1 | 1.612378 | 0.00199 | 0.008324 |
| MXRA5 | 1.609451 | 1.2E-06 | 2.03E-05 |
| ESPN | 1.609162 | 0.000183 | 0.001183 |
| IBSP | 1.607351 | 0.004094 | 0.015032 |
| SYT5 | 1.607327 | 0.001967 | 0.008257 |
| BCAS1 | 1.603436 | 0.000385 | 0.0022 |
| FER1L6 | 1.602315 | 0.014744 | 0.042229 |
| LOC100506305 | 1.600314 | 0.000364 | 0.002102 |
| LINC00469 | 1.599718 | 5.92E-05 | 0.00047 |
| HNF4A | 1.59809 | 0.000738 | 0.003729 |
| LAX1 | 1.597851 | 1.05E-06 | 1.83E-05 |
| FMO1 | 1.596539 | 0.000307 | 0.00183 |
| CD209 | 1.59276 | 5.82E-07 | 1.13E-05 |
| CDHR4 | 1.590118 | 0.014592 | 0.041897 |
| FAM55D | 1.589972 | 0.001186 | 0.005476 |
| LDLRAD3 | 1.588596 | 2.02E-05 | 0.000197 |
| CNGA4 | 1.587711 | 0.011303 | 0.034177 |
| CD1A | 1.586961 | 2.87E-06 | 4.08E-05 |
| EMILIN1 | 1.586354 | 1.15E-06 | 1.96E-05 |
| CRISPLD2 | 1.58502 | 3.76E-05 | 0.000326 |
| MIR4311 | 1.584963 | 0.001017 | 0.004828 |
| APOBEC4 | 1.584635 | 0.013948 | 0.040403 |
| SLC6A2 | 1.584236 | 0.00099 | 0.004739 |
| FABP7 | 1.583667 | 0.007049 | 0.023338 |
| CHIT1 | 1.580775 | 0.017458 | 0.048419 |
| TACR1 | 1.5793 | 5.06E-06 | 6.46E-05 |
| DCLK1 | 1.578546 | 1.06E-06 | 1.85E-05 |
| LOC100506422 | 1.578396 | 0.009538 | 0.029892 |
| FOXN4 | 1.577785 | 0.00848 | 0.027166 |
| SCAMP5 | 1.576237 | 9.53E-08 | 2.59E-06 |
| HIST1H1D | 1.576055 | 0.000395 | 0.002243 |
| GGT5 | 1.575675 | 2.83E-09 | 1.5E-07 |
| TTC29 | 1.575494 | 0.017831 | 0.049261 |
| CCDC135 | 1.574914 | 0.005715 | 0.019754 |
| NWD1 | 1.574854 | 0.012585 | 0.037224 |
| NPAS2 | 1.574777 | 3.97E-10 | 3.08E-08 |
| SPRED3 | 1.574658 | 3.41E-05 | 0.0003 |
| PTGES | 1.573478 | 8.42E-07 | 1.54E-05 |
| C20orf26 | 1.57241 | 0.004581 | 0.016498 |
| ZNF474 | 1.570545 | 0.002901 | 0.011308 |
| LHCGR | 1.570446 | 1.61E-05 | 0.000165 |
| KISS1R | 1.567392 | 0.000175 | 0.001141 |
| SOX11 | 1.565721 | 6.05E-05 | 0.000478 |
| PRR15 | 1.561861 | 0.001431 | 0.006381 |
| CCDC108 | 1.560598 | 0.015048 | 0.042984 |
| LOC100506013 | 1.558507 | 0.002301 | 0.00941 |
| C1QTNF8 | 1.558207 | 0.00453 | 0.016355 |
| LRRTM1 | 1.553664 | 0.000597 | 0.003146 |
| BEND6 | 1.553079 | 5.34E-07 | 1.06E-05 |
| ODZ4 | 1.551535 | 2.4E-07 | 5.53E-06 |
| SDK2 | 1.551273 | 1.86E-07 | 4.48E-06 |
| CCDC129 | 1.55075 | 7.7E-05 | 0.000585 |
| THSD7B | 1.546367 | 0.000157 | 0.001042 |
| FGFBP1 | 1.540989 | 0.006965 | 0.02312 |
| REEP2 | 1.5391 | 1.68E-06 | 2.66E-05 |
| CTSK | 1.535347 | 3.3E-07 | 7.12E-06 |
| KCNK2 | 1.530616 | 0.001129 | 0.005272 |
| C1orf110 | 1.530609 | 0.009573 | 0.029985 |
| KIAA1199 | 1.530358 | 2.3E-05 | 0.000218 |
| C8orf75 | 1.52899 | 0.004961 | 0.017597 |
| OSR2 | 1.52829 | 1.71E-06 | 2.7E-05 |
| RIMS2 | 1.528162 | 0.000666 | 0.003448 |
| TRPV6 | 1.526818 | 1.33E-06 | 2.22E-05 |
| DNAJC12 | 1.526323 | 1.65E-05 | 0.000167 |
| AQP5 | 1.524934 | 5.84E-05 | 0.000466 |
| SPEF1 | 1.523745 | 0.007305 | 0.024071 |
| FBN2 | 1.522927 | 9.3E-05 | 0.000681 |
| CCL19 | 1.521338 | 0.000434 | 0.002418 |
| MS4A6E | 1.520229 | 0.000373 | 0.002143 |
| LINC00525 | 1.518784 | 0.00876 | 0.027867 |
| CCDC113 | 1.517592 | 0.002562 | 0.01026 |
| FLJ43390 | 1.517464 | 0.004874 | 0.017352 |
| SSTR3 | 1.516788 | 0.000118 | 0.000827 |
| IL24 | 1.516614 | 0.000995 | 0.00475 |
| RMRP | 1.516371 | 2.26E-05 | 0.000215 |
| FCGBP | 1.509867 | 2.37E-05 | 0.000224 |
| NIPAL4 | 1.508805 | 0.000327 | 0.001925 |
| LTBP1 | 1.502604 | 1.76E-07 | 4.32E-06 |
| SYNDIG1 | 1.50149 | 2.35E-06 | 3.47E-05 |
| YSK4 | 1.500167 | 0.017876 | 0.049329 |
| FAM5C | 1.500076 | 0.00014 | 0.000956 |
| DNAJB8-AS1 | 1.5 | 0.011554 | 0.034774 |
| SHANK1 | 1.499709 | 9.94E-06 | 0.000109 |
| XCR1 | 1.499511 | 0.000818 | 0.004057 |
| ANKFN1 | 1.494077 | 0.002491 | 0.010022 |
| B3GNT6 | 1.492331 | 0.016277 | 0.045771 |
| CHST4 | 1.492157 | 0.000146 | 0.000985 |
| ZNF215 | 1.491923 | 2.61E-05 | 0.000242 |
| PIM2 | 1.491555 | 4.32E-06 | 5.71E-05 |
| VWCE | 1.491422 | 5.21E-06 | 6.59E-05 |
| TMEM155 | 1.489704 | 0.000366 | 0.002114 |
| ALDH1L2 | 1.489663 | 1.41E-07 | 3.61E-06 |
| SLC2A1 | 1.489295 | 6.58E-07 | 1.25E-05 |
| HMGB3 | 1.48884 | 5.1E-08 | 1.6E-06 |
| C6 | 1.488403 | 0.006709 | 0.022428 |
| GRHL3 | 1.488222 | 4.33E-06 | 5.71E-05 |
| ITGA7 | 1.486893 | 1.32E-07 | 3.41E-06 |
| F5 | 1.482396 | 6.8E-06 | 8.11E-05 |
| CLDN14 | 1.482309 | 0.000621 | 0.003254 |
| SPAG4 | 1.481062 | 1.01E-05 | 0.000111 |
| STEAP2 | 1.479197 | 1.99E-06 | 3.03E-05 |
| ACTC1 | 1.479129 | 0.002935 | 0.011424 |
| IL10 | 1.47698 | 0.000441 | 0.002451 |
| RET | 1.476872 | 3.82E-07 | 8.06E-06 |
| SLAMF9 | 1.476638 | 0.001783 | 0.007617 |
| DARC | 1.476139 | 0.00019 | 0.001224 |
| FBLIM1 | 1.475387 | 2.09E-07 | 4.92E-06 |
| FAM196A | 1.473381 | 0.000122 | 0.000851 |
| S1PR3 | 1.473134 | 1.91E-06 | 2.94E-05 |
| MYH11 | 1.472147 | 3.29E-05 | 0.000292 |
| PPP2R2C | 1.471506 | 0.000981 | 0.004704 |
| LOC402160 | 1.471055 | 0.011462 | 0.034559 |
| EFNB3 | 1.470548 | 0.003923 | 0.014532 |
| C22orf15 | 1.470189 | 0.008267 | 0.026583 |
| IL1RL2 | 1.468546 | 0.000122 | 0.000853 |
| LOC100287314 | 1.468136 | 7.53E-05 | 0.000576 |
| MRAP | 1.466019 | 1.19E-05 | 0.000127 |
| ARMC4 | 1.46439 | 0.014334 | 0.041298 |
| NCCRP1 | 1.464317 | 0.001889 | 0.007977 |
| LOC388630 | 1.46422 | 7.12E-08 | 2.08E-06 |
| FAP | 1.463713 | 9.19E-06 | 0.000103 |
| CA3 | 1.462506 | 2.04E-05 | 0.000198 |
| ADAMTS9 | 1.462493 | 9.81E-05 | 0.000711 |
| PGF | 1.461863 | 1.91E-05 | 0.000188 |
| DLX5 | 1.461854 | 8.37E-05 | 0.000626 |
| CYP7B1 | 1.460525 | 2.76E-05 | 0.000253 |
| PTPRN | 1.460194 | 0.005091 | 0.017968 |
| TCTE1 | 1.460187 | 0.007279 | 0.023989 |
| IGFBPL1 | 1.45737 | 6.16E-05 | 0.000486 |
| CCDC60 | 1.45472 | 0.005722 | 0.019774 |
| DMRTA2 | 1.453964 | 0.000996 | 0.004753 |
| PODN | 1.453479 | 3.59E-06 | 4.91E-05 |
| KERA | 1.451047 | 0.000149 | 0.001004 |
| HES2 | 1.449286 | 2.95E-06 | 4.16E-05 |
| TNFAIP6 | 1.447694 | 0.000815 | 0.004049 |
| DQX1 | 1.446835 | 0.000476 | 0.00261 |
| SFN | 1.445606 | 0.001784 | 0.007617 |
| PI3 | 1.445564 | 0.001832 | 0.007777 |
| PTPRH | 1.445309 | 0.002284 | 0.009355 |
| MFAP2 | 1.444803 | 7.01E-06 | 8.3E-05 |
| KCNG1 | 1.443823 | 1.88E-05 | 0.000186 |
| TLR10 | 1.443786 | 3.33E-05 | 0.000294 |
| CD27 | 1.441429 | 5.81E-05 | 0.000463 |
| VAT1L | 1.439555 | 0.007229 | 0.023847 |
| FOXI2 | 1.439449 | 0.000186 | 0.001199 |
| NRK | 1.438977 | 0.00033 | 0.001937 |
| CFHR3 | 1.436164 | 1.75E-05 | 0.000176 |
| AVPR1A | 1.435721 | 0.003916 | 0.014515 |
| KRT80 | 1.433442 | 2.92E-05 | 0.000264 |
| AGT | 1.432966 | 0.000172 | 0.001125 |
| ROPN1L | 1.432856 | 0.013928 | 0.040354 |
| DNAH5 | 1.431146 | 0.000588 | 0.003106 |
| GCKR | 1.430855 | 0.000243 | 0.001509 |
| SPON1 | 1.430757 | 3.74E-08 | 1.24E-06 |
| PMCH | 1.428742 | 0.000367 | 0.002115 |
| KLHDC7A | 1.428721 | 4.48E-06 | 5.87E-05 |
| RAMP1 | 1.427964 | 7.63E-06 | 8.9E-05 |
| CCDC160 | 1.427635 | 0.003543 | 0.013384 |
| F13A1 | 1.426438 | 0.0002 | 0.001278 |
| MMRN1 | 1.425149 | 0.000527 | 0.002853 |
| LOC100422737 | 1.424036 | 0.010013 | 0.03105 |
| GPX8 | 1.420247 | 1.79E-07 | 4.36E-06 |
| CD24 | 1.419899 | 4.27E-06 | 5.66E-05 |
| TOX3 | 1.418156 | 3.28E-05 | 0.000291 |
| ACY3 | 1.41615 | 6.59E-05 | 0.000513 |
| DNAH2 | 1.412961 | 0.004994 | 0.017689 |
| C6orf165 | 1.412446 | 0.011957 | 0.035737 |
| EPHA3 | 1.411383 | 9.94E-06 | 0.000109 |
| KLHL13 | 1.408861 | 0.000151 | 0.001016 |
| D4S234E | 1.406089 | 2.77E-06 | 3.96E-05 |
| HSPA4L | 1.405856 | 6.87E-06 | 8.17E-05 |
| FOXP3 | 1.405141 | 1.33E-07 | 3.44E-06 |
| LRRC4C | 1.404855 | 7.9E-05 | 0.000597 |
| FAM198A | 1.402978 | 3.32E-06 | 4.58E-05 |
| CPZ | 1.402733 | 6.59E-05 | 0.000513 |
| CRISP2 | 1.40174 | 0.014703 | 0.04214 |
| FUT3 | 1.401699 | 5.51E-10 | 4.02E-08 |
| S100P | 1.399926 | 0.000237 | 0.001479 |
| CKMT1A | 1.39915 | 0.000991 | 0.004742 |
| ST8SIA2 | 1.39875 | 0.001467 | 0.006502 |
| NGEF | 1.397228 | 0.001329 | 0.006005 |
| BDKRB1 | 1.396296 | 0.013432 | 0.039189 |
| ABRA | 1.396212 | 0.000124 | 0.000862 |
| SBSN | 1.39618 | 0.011084 | 0.033656 |
| PLEKHG7 | 1.395974 | 0.003141 | 0.012082 |
| LRRC43 | 1.395299 | 0.005015 | 0.017743 |
| IGLL1 | 1.388333 | 0.001723 | 0.007415 |
| FJX1 | 1.384728 | 6.81E-06 | 8.11E-05 |
| PRUNE2 | 1.384718 | 8.38E-05 | 0.000626 |
| C14orf37 | 1.384576 | 1.75E-05 | 0.000175 |
| ARL9 | 1.38288 | 9.48E-05 | 0.000692 |
| TUBBP5 | 1.377979 | 0.000597 | 0.003146 |
| B4GALNT2 | 1.377409 | 0.009395 | 0.029537 |
| CAPN5 | 1.376037 | 5.6E-09 | 2.6E-07 |
| SIX3 | 1.375837 | 0.001743 | 0.007489 |
| RGMA | 1.375828 | 3.72E-06 | 5.05E-05 |
| BASP1 | 1.375321 | 1.34E-06 | 2.22E-05 |
| ZNF648 | 1.374626 | 0.001266 | 0.005771 |
| TGM3 | 1.372342 | 0.007035 | 0.023299 |
| R3HDML | 1.371001 | 0.010336 | 0.031813 |
| HTR2B | 1.367025 | 0.000268 | 0.001635 |
| PF4V1 | 1.366361 | 0.003292 | 0.012552 |
| GPR37 | 1.365709 | 2.19E-05 | 0.00021 |
| AK4 | 1.365265 | 2.19E-05 | 0.000209 |
| TRIM55 | 1.364306 | 0.000691 | 0.003544 |
| CBLC | 1.362794 | 4.59E-07 | 9.41E-06 |
| FBXO16 | 1.362392 | 5.91E-06 | 7.27E-05 |
| ARX | 1.362225 | 0.014009 | 0.040563 |
| PTCHD4 | 1.360082 | 3.13E-05 | 0.00028 |
| NME1-NME2 | 1.359608 | 0.002721 | 0.010742 |
| FKBP11 | 1.359565 | 8.86E-07 | 1.6E-05 |
| UCHL1 | 1.359481 | 1.6E-05 | 0.000163 |
| BPIFA2 | 1.359102 | 0.004595 | 0.016538 |
| HYDIN | 1.353265 | 0.005507 | 0.019132 |
| ABCG4 | 1.353142 | 0.000197 | 0.001261 |
| PVRL1 | 1.353134 | 4.45E-08 | 1.42E-06 |
| LRRIQ1 | 1.352871 | 0.011217 | 0.033995 |
| SGPP2 | 1.352497 | 9.78E-05 | 0.00071 |
| ITGBL1 | 1.352099 | 9.55E-05 | 0.000696 |
| FGF7 | 1.350988 | 4.8E-05 | 0.000398 |
| WDR63 | 1.35092 | 0.008042 | 0.026009 |
| LOC100507050 | 1.350552 | 4.57E-05 | 0.000383 |
| CD164L2 | 1.35029 | 0.009031 | 0.028589 |
| FLG | 1.34968 | 0.004083 | 0.015001 |
| DLG2 | 1.348981 | 4.02E-08 | 1.32E-06 |
| CFB | 1.348598 | 3.19E-06 | 4.45E-05 |
| ATP1A4 | 1.348174 | 0.000156 | 0.001039 |
| FOXI1 | 1.347552 | 0.003371 | 0.012822 |
| SRPX2 | 1.347091 | 4.14E-08 | 1.35E-06 |
| LOC728978 | 1.346904 | 0.000163 | 0.001078 |
| OAF | 1.345901 | 8.5E-07 | 1.55E-05 |
| C1S | 1.345094 | 5.76E-08 | 1.75E-06 |
| CD38 | 1.344399 | 0.000106 | 0.00076 |
| PNCK | 1.343484 | 0.003083 | 0.011899 |
| ECT2L | 1.342932 | 0.011262 | 0.034101 |
| DIO3 | 1.342818 | 0.000162 | 0.00107 |
| OLIG2 | 1.34055 | 0.008863 | 0.028149 |
| NEK2 | 1.33773 | 0.000443 | 0.002458 |
| PTGER1 | 1.335415 | 0.000419 | 0.00235 |
| KIF19 | 1.333014 | 0.00796 | 0.025802 |
| ALDH1L1 | 1.332484 | 0.001498 | 0.006617 |
| CDH17 | 1.332129 | 0.000173 | 0.001129 |
| CHST8 | 1.3292 | 0.000276 | 0.001673 |
| ESR2 | 1.32841 | 2.94E-06 | 4.15E-05 |
| CCL21 | 1.327793 | 0.000644 | 0.003355 |
| BCL2L15 | 1.327675 | 8.86E-08 | 2.43E-06 |
| KCNMA1 | 1.32739 | 9.83E-06 | 0.000109 |
| DHRS9 | 1.327105 | 4.6E-05 | 0.000385 |
| TSPAN11 | 1.324061 | 9.63E-07 | 1.71E-05 |
| MOBP | 1.321203 | 0.003264 | 0.012464 |
| SAMD15 | 1.321029 | 0.001492 | 0.006593 |
| FAM20C | 1.318866 | 8.39E-07 | 1.53E-05 |
| TMEM40 | 1.317063 | 0.001444 | 0.006426 |
| FBXO32 | 1.316392 | 2.3E-06 | 3.41E-05 |
| ADAM28 | 1.315281 | 4.31E-07 | 8.95E-06 |
| SLC28A3 | 1.31407 | 0.000644 | 0.003353 |
| C3orf67 | 1.313779 | 0.008067 | 0.026071 |
| IGJ | 1.313529 | 0.001793 | 0.007651 |
| CPXM2 | 1.313065 | 3.46E-05 | 0.000304 |
| AGR2 | 1.312416 | 1.37E-05 | 0.000143 |
| HIST1H2BH | 1.311645 | 0.000537 | 0.002893 |
| PRRX2 | 1.309982 | 2.16E-05 | 0.000207 |
| WDR65 | 1.308953 | 0.015575 | 0.044215 |
| CCDC78 | 1.306149 | 0.017337 | 0.048146 |
| SOWAHA | 1.305684 | 0.00969 | 0.030261 |
| PLTP | 1.303979 | 7.78E-06 | 9.05E-05 |
| FRMPD2P1 | 1.303741 | 0.012268 | 0.036471 |
| IGSF1 | 1.303063 | 0.000208 | 0.001323 |
| SLC44A4 | 1.302007 | 0.000458 | 0.002521 |
| CD79B | 1.30197 | 4.49E-05 | 0.000377 |
| PLVAP | 1.3019 | 0.00036 | 0.002084 |
| MYBPC2 | 1.30025 | 0.00058 | 0.003069 |
| SLC30A2 | 1.300064 | 0.000729 | 0.003694 |
| KCNE4 | 1.299415 | 0.000716 | 0.003645 |
| CATSPERB | 1.298634 | 2.77E-05 | 0.000254 |
| LOC728175 | 1.298272 | 8.45E-05 | 0.00063 |
| LOC653786 | 1.295022 | 0.002814 | 0.011031 |
| COL25A1 | 1.294872 | 0.001466 | 0.006497 |
| MIA | 1.294589 | 0.012049 | 0.035955 |
| LOXL1 | 1.293237 | 6.05E-06 | 7.4E-05 |
| RSPH9 | 1.292699 | 0.010153 | 0.03139 |
| LOC338579 | 1.292481 | 9.47E-05 | 0.000691 |
| PRR7 | 1.291481 | 1.88E-05 | 0.000186 |
| HRH2 | 1.289276 | 8.2E-05 | 0.000616 |
| CDR1 | 1.289036 | 0.004591 | 0.016525 |
| MTHFD2 | 1.288427 | 2.16E-06 | 3.24E-05 |
| FAM81B | 1.28765 | 0.010616 | 0.032508 |
| RNASE2 | 1.287486 | 0.000258 | 0.001585 |
| CLDN1 | 1.285366 | 0.000123 | 0.000853 |
| CCDC151 | 1.284935 | 0.014728 | 0.042199 |
| ANKRD34B | 1.280363 | 0.000324 | 0.001911 |
| HOXC6 | 1.280146 | 0.007652 | 0.024957 |
| C9orf116 | 1.279467 | 0.008305 | 0.026694 |
| PKIB | 1.277793 | 0.000179 | 0.001164 |
| DGCR5 | 1.277364 | 0.000292 | 0.001753 |
| IER5L | 1.277097 | 7.79E-06 | 9.05E-05 |
| HS3ST3A1 | 1.276692 | 0.000796 | 0.003965 |
| LOC729966 | 1.276411 | 0.008787 | 0.027936 |
| COL8A1 | 1.276402 | 0.00053 | 0.002867 |
| WNT10A | 1.276264 | 7.35E-05 | 0.000563 |
| DDIT4 | 1.274378 | 0.000261 | 0.001599 |
| CLIC6 | 1.274347 | 3.51E-05 | 0.000308 |
| RIBC2 | 1.274221 | 0.011083 | 0.033656 |
| C11orf70 | 1.273573 | 0.0031 | 0.011951 |
| MRVI1 | 1.272059 | 9.76E-07 | 1.72E-05 |
| KIAA1211 | 1.271689 | 1.45E-06 | 2.37E-05 |
| ADRA2A | 1.270525 | 2.58E-06 | 3.73E-05 |
| GYG2 | 1.269435 | 2.58E-05 | 0.00024 |
| E2F8 | 1.269186 | 2.02E-05 | 0.000197 |
| C21orf88 | 1.268824 | 0.004473 | 0.016184 |
| C9orf174 | 1.268437 | 0.001759 | 0.007538 |
| SLC2A4 | 1.265822 | 1.28E-05 | 0.000135 |
| ANLN | 1.264119 | 7.33E-05 | 0.000562 |
| ITGB3 | 1.261245 | 5.19E-06 | 6.58E-05 |
| METTL24 | 1.261171 | 0.000507 | 0.002765 |
| COCH | 1.257312 | 8.86E-07 | 1.6E-05 |
| C1R | 1.257072 | 3.81E-07 | 8.05E-06 |
| KCNK15 | 1.253856 | 0.000359 | 0.00208 |
| RPRML | 1.2533 | 0.002719 | 0.010737 |
| ZMYND10 | 1.252386 | 0.014548 | 0.041818 |
| LEPREL2 | 1.251734 | 1.5E-06 | 2.44E-05 |
| THBS4 | 1.250223 | 1.29E-06 | 2.16E-05 |
| KCNJ16 | 1.249524 | 0.017384 | 0.04825 |
| SCARA5 | 1.249346 | 0.000752 | 0.003789 |
| SLC1A3 | 1.249208 | 0.000143 | 0.000973 |
| C16orf71 | 1.248818 | 0.007742 | 0.025215 |
| MGC45800 | 1.247246 | 0.006707 | 0.022423 |
| C8orf80 | 1.245032 | 3.19E-05 | 0.000285 |
| CAPS | 1.244245 | 0.015524 | 0.044122 |
| KIF4A | 1.244228 | 0.000383 | 0.002196 |
| AIM2 | 1.243808 | 1.52E-05 | 0.000156 |
| BACE2 | 1.242799 | 3.13E-08 | 1.07E-06 |
| LRRC17 | 1.241511 | 3.81E-06 | 5.16E-05 |
| LOC154860 | 1.239422 | 0.001547 | 0.006795 |
| P2RX5 | 1.239134 | 0.000106 | 0.000759 |
| CUX2 | 1.237434 | 0.000719 | 0.003655 |
| PACRG | 1.234998 | 0.014133 | 0.040807 |
| SDK1 | 1.234854 | 2.99E-07 | 6.57E-06 |
| KCTD1 | 1.234124 | 5.87E-06 | 7.25E-05 |
| GPHA2 | 1.231098 | 0.000142 | 0.000965 |
| ABCB11 | 1.230207 | 0.010475 | 0.032134 |
| PKP2 | 1.229576 | 0.007058 | 0.023362 |
| KIF1A | 1.229567 | 0.00803 | 0.025981 |
| LINGO1 | 1.229233 | 5.06E-05 | 0.000414 |
| TLL2 | 1.22813 | 0.000269 | 0.001641 |
| CCL18 | 1.227529 | 0.008012 | 0.025943 |
| CCDC17 | 1.227073 | 0.01769 | 0.048961 |
| C12orf68 | 1.226252 | 0.003113 | 0.011993 |
| MMP14 | 1.225716 | 2.62E-06 | 3.79E-05 |
| WSCD2 | 1.225387 | 1.95E-05 | 0.000191 |
| PLCH2 | 1.225257 | 6.32E-05 | 0.000496 |
| LOC100507421 | 1.222909 | 1.94E-05 | 0.00019 |
| GPR84 | 1.222353 | 0.001471 | 0.006517 |
| KCNN4 | 1.222002 | 3.61E-07 | 7.66E-06 |
| TNFRSF21 | 1.220451 | 5.28E-08 | 1.65E-06 |
| WDR66 | 1.21923 | 0.012051 | 0.035958 |
| GAD1 | 1.218418 | 0.006313 | 0.021374 |
| SMOC1 | 1.21753 | 0.000274 | 0.001668 |
| MYBL2 | 1.216701 | 0.000575 | 0.003048 |
| PVT1 | 1.215934 | 1.47E-06 | 2.39E-05 |
| TMEM232 | 1.215468 | 0.010898 | 0.033196 |
| DPP10 | 1.215366 | 0.001041 | 0.004918 |
| CAPN6 | 1.214986 | 0.00131 | 0.005938 |
| AQP2 | 1.213478 | 0.000986 | 0.004722 |
| KCNQ3 | 1.210625 | 0.000185 | 0.001195 |
| ADCY2 | 1.210316 | 6.24E-05 | 0.000491 |
| GNG4 | 1.208649 | 0.003934 | 0.014559 |
| IRF4 | 1.208386 | 0.00167 | 0.007216 |
| LOC100499227 | 1.207221 | 0.007 | 0.023211 |
| CHPF | 1.207024 | 1.27E-06 | 2.13E-05 |
| PROK1 | 1.206259 | 0.007546 | 0.024663 |
| ST6GAL1 | 1.206237 | 1.27E-07 | 3.31E-06 |
| SMPX | 1.206101 | 0.002629 | 0.010463 |
| DNAH7 | 1.204626 | 0.010413 | 0.031987 |
| CDHR2 | 1.204133 | 0.000405 | 0.00229 |
| PCBP3 | 1.203963 | 2.37E-05 | 0.000224 |
| HOXC5 | 1.20293 | 0.004064 | 0.014958 |
| HIST1H2AG | 1.201108 | 0.00085 | 0.004184 |
| HS3ST2 | 1.20039 | 0.000332 | 0.001951 |
| ADAMTS2 | 1.200245 | 4.71E-06 | 6.07E-05 |
| DAPL1 | 1.199821 | 0.004345 | 0.015802 |
| TRPV4 | 1.199262 | 0.000736 | 0.003721 |
| CLEC11A | 1.198232 | 1.31E-05 | 0.000138 |
| FAM46C | 1.197806 | 1.2E-05 | 0.000128 |
| CNFN | 1.197486 | 0.000169 | 0.001111 |
| SLC10A6 | 1.196385 | 1.72E-05 | 0.000173 |
| SLC1A4 | 1.194916 | 3.2E-07 | 6.96E-06 |
| SGK110 | 1.193848 | 0.000507 | 0.002765 |
| HIST1H2AE | 1.191894 | 0.000583 | 0.003085 |
| COL18A1 | 1.19098 | 5.56E-07 | 1.09E-05 |
| C7 | 1.18991 | 0.000715 | 0.00364 |
| ITGB4 | 1.189875 | 6.44E-06 | 7.78E-05 |
| SLC39A14 | 1.18859 | 0.000104 | 0.000747 |
| ATP1A2 | 1.188263 | 0.00166 | 0.007179 |
| LOC100506385 | 1.188122 | 4.57E-05 | 0.000383 |
| SPATA17 | 1.184747 | 0.011583 | 0.034837 |
| PDK1 | 1.184627 | 4.9E-05 | 0.000403 |
| COL19A1 | 1.183586 | 0.000557 | 0.002974 |
| ANKRD45 | 1.180951 | 0.009217 | 0.02907 |
| INA | 1.180671 | 0.002627 | 0.010459 |
| FAM81A | 1.180511 | 0.002632 | 0.010473 |
| ZSCAN4 | 1.179751 | 0.008101 | 0.026154 |
| IGFBP4 | 1.178438 | 8.89E-07 | 1.61E-05 |
| SYNPO2 | 1.17843 | 0.000124 | 0.000862 |
| AK7 | 1.176975 | 0.013546 | 0.039436 |
| RAET1E | 1.176396 | 0.006756 | 0.022559 |
| GNAO1 | 1.175441 | 2E-07 | 4.74E-06 |
| HEPH | 1.175294 | 1.65E-06 | 2.62E-05 |
| COL9A3 | 1.173343 | 0.001252 | 0.005724 |
| C2orf70 | 1.172391 | 0.009854 | 0.030665 |
| CHL1 | 1.17169 | 3.78E-06 | 5.12E-05 |
| PDE1A | 1.170376 | 2.55E-05 | 0.000238 |
| LOC728739 | 1.16926 | 0.001001 | 0.004771 |
| EFHC2 | 1.169104 | 0.006469 | 0.021778 |
| TNFRSF10C | 1.164465 | 0.000391 | 0.002226 |
| UMODL1 | 1.163309 | 0.006917 | 0.022994 |
| ASPHD1 | 1.163289 | 0.000824 | 0.004079 |
| MIR3648 | 1.161993 | 0.004218 | 0.015416 |
| COLEC11 | 1.161147 | 0.002629 | 0.010463 |
| TG | 1.160764 | 4.69E-06 | 6.05E-05 |
| STEAP3 | 1.160337 | 1.15E-06 | 1.96E-05 |
| KIF18B | 1.16031 | 0.00012 | 0.000839 |
| ACTA2 | 1.160255 | 0.000377 | 0.002161 |
| SNORA14B | 1.157942 | 0.001163 | 0.00539 |
| PLN | 1.157337 | 0.00584 | 0.020083 |
| HIST3H2A | 1.15713 | 0.000666 | 0.003448 |
| CCL22 | 1.15634 | 0.000117 | 0.000826 |
| LOC100130417 | 1.155692 | 7.85E-05 | 0.000594 |
| CHAC1 | 1.15473 | 0.009773 | 0.030455 |
| LRRN2 | 1.153858 | 4.15E-05 | 0.000353 |
| KIF20A | 1.153425 | 8.66E-05 | 0.000643 |
| RASD2 | 1.149527 | 0.000103 | 0.000739 |
| ATP6V0A4 | 1.149148 | 0.00651 | 0.021884 |
| SLAMF7 | 1.149041 | 0.000234 | 0.00146 |
| HIST1H3C | 1.149036 | 0.014568 | 0.041847 |
| TMPRSS7 | 1.148995 | 0.00536 | 0.018725 |
| SPATA18 | 1.148607 | 0.005337 | 0.018659 |
| CLDN16 | 1.147976 | 0.017454 | 0.048414 |
| ASTN1 | 1.14756 | 0.010423 | 0.032007 |
| TP73 | 1.144785 | 0.003944 | 0.014585 |
| ASB2 | 1.144664 | 4.71E-05 | 0.000392 |
| GOLM1 | 1.142196 | 9.86E-07 | 1.74E-05 |
| IGLON5 | 1.141538 | 0.002888 | 0.011266 |
| ADAMTS19 | 1.141001 | 0.004496 | 0.016247 |
| SULF2 | 1.139589 | 4.15E-06 | 5.52E-05 |
| DNAJB13 | 1.138598 | 0.012249 | 0.036421 |
| ECE2 | 1.137995 | 5.72E-06 | 7.1E-05 |
| TOP2A | 1.137819 | 0.000426 | 0.002384 |
| TAGLN | 1.137473 | 1.64E-05 | 0.000166 |
| SERINC2 | 1.137288 | 2.08E-05 | 0.000201 |
| SLC17A9 | 1.136478 | 1.88E-06 | 2.9E-05 |
| METTL7B | 1.136412 | 0.000297 | 0.00178 |
| ALDH1B1 | 1.136246 | 8.73E-06 | 9.9E-05 |
| CLU | 1.136068 | 9.14E-06 | 0.000103 |
| TMC3 | 1.134645 | 0.000407 | 0.0023 |
| GPR12 | 1.134612 | 0.009489 | 0.029779 |
| MYOCD | 1.134365 | 0.003088 | 0.011913 |
| SMOC2 | 1.13352 | 7.02E-05 | 0.000542 |
| OR52N5 | 1.133333 | 0.01246 | 0.036931 |
| KCNF1 | 1.133131 | 0.000284 | 0.001717 |
| LAMA2 | 1.132749 | 3.28E-05 | 0.000292 |
| SPSB1 | 1.13269 | 0.001074 | 0.005048 |
| SLC16A7 | 1.130237 | 0.001483 | 0.00656 |
| LOC100131176 | 1.129258 | 0.015521 | 0.044122 |
| SLC25A21 | 1.129031 | 0.001598 | 0.006966 |
| SFRP4 | 1.129028 | 0.006702 | 0.022415 |
| HRASLS2 | 1.128161 | 0.00228 | 0.009343 |
| TPX2 | 1.127995 | 0.000673 | 0.003473 |
| TNFRSF11B | 1.12791 | 0.007816 | 0.025395 |
| FOXA3 | 1.127156 | 0.000555 | 0.002969 |
| RGS22 | 1.126833 | 0.007267 | 0.023951 |
| LINC00494 | 1.126685 | 0.000992 | 0.004742 |
| RSPH4A | 1.124626 | 0.016494 | 0.046266 |
| CDC6 | 1.124282 | 2.45E-06 | 3.58E-05 |
| DLGAP5 | 1.122417 | 0.000395 | 0.002245 |
| AK8 | 1.122316 | 0.011278 | 0.034113 |
| DFNA5 | 1.121557 | 1.68E-05 | 0.00017 |
| UBXN10 | 1.121091 | 0.006237 | 0.021163 |
| ZG16B | 1.117558 | 0.01004 | 0.031113 |
| IQUB | 1.116359 | 0.017102 | 0.047597 |
| PLEKHA4 | 1.11563 | 4.63E-06 | 6.01E-05 |
| TMEM156 | 1.115031 | 0.00056 | 0.002987 |
| S100G | 1.114991 | 0.006111 | 0.020838 |
| S100B | 1.112949 | 0.0001 | 0.000725 |
| GPR1 | 1.112619 | 0.008705 | 0.02775 |
| FAM171B | 1.112129 | 0.003774 | 0.01409 |
| IL31RA | 1.110535 | 0.000735 | 0.003717 |
| LRRN4CL | 1.109573 | 0.001352 | 0.006087 |
| MIR1204 | 1.108589 | 0.002491 | 0.010024 |
| RHBDL3 | 1.108134 | 3.53E-05 | 0.00031 |
| LILRB4 | 1.108075 | 0.000135 | 0.000925 |
| PAMR1 | 1.108068 | 0.000571 | 0.003033 |
| CENPM | 1.10536 | 0.002489 | 0.010019 |
| UHRF1 | 1.104656 | 7.66E-06 | 8.92E-05 |
| GPR176 | 1.104547 | 0.000771 | 0.003865 |
| C3orf32 | 1.102416 | 0.015801 | 0.044705 |
| APCDD1L | 1.102404 | 0.004282 | 0.015606 |
| SEL1L3 | 1.100885 | 3.01E-07 | 6.61E-06 |
| CCDC103 | 1.10062 | 0.009535 | 0.029888 |
| MSC | 1.09994 | 0.004708 | 0.016859 |
| MKI67 | 1.099523 | 0.000145 | 0.000984 |
| MEIS3 | 1.099398 | 1.64E-05 | 0.000167 |
| FKBP10 | 1.099391 | 9.92E-06 | 0.000109 |
| SRGAP3 | 1.098053 | 0.001137 | 0.005292 |
| FSTL1 | 1.097551 | 4.34E-05 | 0.000367 |
| RALGPS2 | 1.096667 | 6.43E-06 | 7.78E-05 |
| LRRC73 | 1.095412 | 0.008737 | 0.027815 |
| TRIP13 | 1.095236 | 0.003635 | 0.013671 |
| ELN | 1.094244 | 0.000221 | 0.001391 |
| MND1 | 1.091836 | 4.91E-05 | 0.000403 |
| KIAA1024 | 1.088267 | 3.26E-06 | 4.52E-05 |
| PBK | 1.08526 | 0.004141 | 0.015186 |
| ENTHD1 | 1.084281 | 0.001707 | 0.00735 |
| GAS7 | 1.083548 | 7.89E-06 | 9.15E-05 |
| ADORA3 | 1.081747 | 0.001395 | 0.006243 |
| GPR88 | 1.080677 | 0.006374 | 0.021531 |
| HS3ST1 | 1.080548 | 0.000178 | 0.001158 |
| INSL3 | 1.079444 | 0.000875 | 0.004287 |
| URB1 | 1.078541 | 4.41E-08 | 1.42E-06 |
| CEACAM7 | 1.078355 | 0.016048 | 0.045273 |
| MEG8 | 1.076089 | 0.002309 | 0.009436 |
| PRDX4 | 1.075961 | 5.84E-06 | 7.21E-05 |
| AHNAK2 | 1.075493 | 0.000336 | 0.001971 |
| PRRX1 | 1.074189 | 2.62E-05 | 0.000243 |
| NRXN2 | 1.072825 | 0.000122 | 0.000851 |
| LOC154822 | 1.072119 | 0.005735 | 0.019806 |
| BICC1 | 1.071078 | 0.001641 | 0.007117 |
| NTS | 1.070387 | 0.004213 | 0.015403 |
| MIR4260 | 1.070267 | 0.003709 | 0.013904 |
| HMCN1 | 1.069802 | 5.31E-05 | 0.00043 |
| FOXC2 | 1.069303 | 0.001455 | 0.006457 |
| TTYH3 | 1.0674 | 6.63E-07 | 1.26E-05 |
| LOC153469 | 1.067037 | 0.009021 | 0.028574 |
| PLAU | 1.066757 | 6.29E-05 | 0.000494 |
| ADRB3 | 1.066648 | 0.011106 | 0.033713 |
| LPAR4 | 1.066571 | 0.003736 | 0.013996 |
| CASQ1 | 1.066166 | 0.000142 | 0.000965 |
| STK32A | 1.065139 | 6.47E-06 | 7.8E-05 |
| NME1 | 1.064657 | 1.85E-05 | 0.000183 |
| KCNH3 | 1.06456 | 0.011439 | 0.034507 |
| GREB1 | 1.064414 | 4.59E-05 | 0.000384 |
| DEPDC1 | 1.064199 | 0.00039 | 0.002225 |
| NDP | 1.063976 | 0.012956 | 0.0381 |
| TEX9 | 1.062657 | 0.006733 | 0.0225 |
| GRIK5 | 1.062489 | 0.000176 | 0.001146 |
| ITM2C | 1.061767 | 3.64E-05 | 0.000318 |
| RRM2 | 1.060838 | 0.000939 | 0.004539 |
| CDKN3 | 1.060794 | 0.000454 | 0.002507 |
| SLC16A10 | 1.059387 | 0.010139 | 0.031352 |
| UGT1A3 | 1.055533 | 0.008378 | 0.02689 |
| MESP1 | 1.054776 | 4.78E-05 | 0.000397 |
| LOX | 1.054613 | 3.2E-05 | 0.000285 |
| SPIB | 1.05276 | 0.00232 | 0.009467 |
| CHRNA4 | 1.051742 | 0.004597 | 0.01654 |
| MFI2 | 1.051538 | 2.77E-05 | 0.000253 |
| RAB36 | 1.051461 | 0.001976 | 0.008281 |
| AOX1 | 1.051341 | 0.00088 | 0.004304 |
| KDELR3 | 1.051211 | 6E-06 | 7.36E-05 |
| TRIM31 | 1.051149 | 0.009026 | 0.02858 |
| HIST1H2BG | 1.050609 | 0.00199 | 0.008324 |
| GPR172B | 1.049881 | 0.001025 | 0.004861 |
| CDKN2A | 1.04937 | 0.000633 | 0.003307 |
| UNC13A | 1.049291 | 0.000232 | 0.001453 |
| LRRC55 | 1.04846 | 0.012835 | 0.037818 |
| TNN | 1.048364 | 0.005427 | 0.018929 |
| LRFN5 | 1.047397 | 0.000995 | 0.004752 |
| LOC645431 | 1.046629 | 0.001727 | 0.00743 |
| RAB3IL1 | 1.042366 | 7.87E-05 | 0.000596 |
| APOD | 1.040843 | 0.001529 | 0.006727 |
| E2F7 | 1.039989 | 0.001255 | 0.005732 |
| CRLF2 | 1.039663 | 0.001455 | 0.006457 |
| CRLF2 | 1.039663 | 0.001455 | 0.006457 |
| C15orf48 | 1.039015 | 0.000287 | 0.001727 |
| GPC1 | 1.038266 | 1.78E-05 | 0.000177 |
| PZP | 1.038062 | 0.000589 | 0.00311 |
| FAT1 | 1.037643 | 1.16E-05 | 0.000124 |
| CDC20 | 1.03665 | 0.000613 | 0.003218 |
| CPA3 | 1.035328 | 0.000365 | 0.002106 |
| POM121L9P | 1.034442 | 0.000319 | 0.001888 |
| CCDC3 | 1.033944 | 0.000156 | 0.001039 |
| C18orf56 | 1.03277 | 0.000537 | 0.002891 |
| HORMAD1 | 1.032744 | 0.004912 | 0.017456 |
| VCAM1 | 1.032069 | 0.013595 | 0.039545 |
| HSPB7 | 1.031955 | 0.001617 | 0.007033 |
| ALDH18A1 | 1.030244 | 5.85E-08 | 1.77E-06 |
| ITGA2 | 1.030052 | 0.001075 | 0.005052 |
| LINC00511 | 1.030051 | 0.005337 | 0.018659 |
| C1QTNF1 | 1.029224 | 0.002592 | 0.010349 |
| KCNE1 | 1.029191 | 0.005336 | 0.018659 |
| MSI2 | 1.029189 | 7.58E-06 | 8.84E-05 |
| ZNF703 | 1.029054 | 0.000547 | 0.002931 |
| KCNMB1 | 1.028026 | 0.000319 | 0.001888 |
| SSR4 | 1.027468 | 3.75E-05 | 0.000326 |
| C9orf129 | 1.025167 | 0.00067 | 0.003464 |
| PC | 1.025034 | 2.63E-06 | 3.79E-05 |
| TMEM132C | 1.023933 | 0.002892 | 0.011279 |
| LOC154092 | 1.023314 | 0.016486 | 0.04625 |
| RBM24 | 1.021629 | 0.011702 | 0.035121 |
| LINGO3 | 1.021478 | 0.000168 | 0.001106 |
| LOC400958 | 1.019833 | 0.013271 | 0.038804 |
| SLC4A3 | 1.017998 | 1.17E-05 | 0.000125 |
| CSPG5 | 1.016945 | 0.008681 | 0.027686 |
| ESCO2 | 1.016933 | 0.002456 | 0.009911 |
| LXN | 1.015538 | 6.99E-05 | 0.00054 |
| LPPR4 | 1.014529 | 0.00064 | 0.003338 |
| CRYBB1 | 1.012644 | 0.000694 | 0.003556 |
| ELANE | 1.012563 | 0.003211 | 0.012309 |
| SAPCD2 | 1.011615 | 9.94E-06 | 0.000109 |
| PNMAL1 | 1.010772 | 0.00719 | 0.023734 |
| NCAPH | 1.01052 | 0.000172 | 0.001123 |
| CALU | 1.009878 | 3.85E-06 | 5.2E-05 |
| TPBG | 1.009694 | 4.34E-07 | 8.99E-06 |
| RHBDL2 | 1.009634 | 5.81E-05 | 0.000464 |
| NXPH4 | 1.006577 | 0.00642 | 0.021655 |
| SYNJ2 | 1.00563 | 5.16E-06 | 6.55E-05 |
| LUM | 1.005423 | 0.000299 | 0.00179 |
| SCARA3 | 1.00502 | 0.000118 | 0.00083 |
| LOC100507351 | 1.004745 | 0.002439 | 0.009858 |
| PGM2L1 | 1.004071 | 4.97E-06 | 6.35E-05 |
| TNFRSF18 | 1.002718 | 0.000182 | 0.001179 |
| MAT1A | 1.001653 | 0.012343 | 0.036639 |
| WBSCR17 | 1.001397 | 0.000186 | 0.001199 |
| KIRREL | 1.000752 | 0.000403 | 0.002282 |
| LOC100507086 | 1.000609 | 0.003594 | 0.013553 |
