# Supplementary material for: Danggui Buxue Decoction Ameliorates Idiopathic Pulmonary Fibrosis through MicroRNA and Messenger RNA Regulatory Network
Source: Evid Based Complement Alternat Med. 2022 Apr 26;2022:3439656. doi: 10.1155/2022/3439656 (PMC9064538; doi:10.1155/2022/3439656)
Supplement: Supplementary Materials — Table S1: DGBXD granules. Table S2: Szapiel score system. Table S3: Ashcroft score system. Table S4: predicted target genes of upregulated DE-miRNAs (n = 1285). Table S5: predicted target genes of downregulated DE-miRNAs (n = 1411). Table S6: upregulated DE-mRNAs (n = 1160). Table S7: downregulated DE-mRNAs (n = 1427). Table S8: corresponding gene symbols of RA and RAS. [file 3439656.f1.zip › 3439656.f1/Table S7 Downregulated DE-mRNAs (n=1427).docx]

**Table S7:** Downregulated DE-mRNAs (n=1427).

|  | **logFC** | ***p-value*** | **Adj *p-value*** |
| --- | --- | --- | --- |
| SLCO1A2 | -6.43645 | 8.88E-14 | 5.81E-11 |
| ITLN2 | -5.82047 | 2.12E-16 | 1.18E-12 |
| SERTM1 | -5.32355 | 1.09E-14 | 1.56E-11 |
| RTKN2 | -4.88609 | 2.31E-15 | 6.35E-12 |
| GALNT13 | -4.50348 | 6.74E-15 | 1.11E-11 |
| CA4 | -4.28482 | 4.4E-11 | 5.59E-09 |
| ADAM29 | -4.14588 | 1.6E-11 | 2.52E-09 |
| RP11-165H20 | -4.10801 | 4.11E-08 | 1.34E-06 |
| SLC6A4 | -3.96184 | 3.96E-06 | 5.31E-05 |
| SOSTDC1 | -3.85525 | 1.36E-10 | 1.32E-08 |
| FLJ34503 | -3.79388 | 1.49E-11 | 2.41E-09 |
| C2orf91 | -3.74358 | 2.94E-12 | 8.39E-10 |
| AGER | -3.66591 | 3.58E-13 | 1.6E-10 |
| MAP3K15 | -3.60855 | 3.6E-14 | 3.26E-11 |
| ADCY8 | -3.60515 | 3.74E-09 | 1.86E-07 |
| FOSB | -3.57198 | 2.49E-06 | 3.63E-05 |
| FAM71A | -3.52298 | 3.56E-05 | 0.000311 |
| BTNL9 | -3.49774 | 2.91E-14 | 2.84E-11 |
| FGFBP2 | -3.40604 | 9.84E-10 | 6.41E-08 |
| SLC14A1 | -3.34211 | 3.08E-10 | 2.48E-08 |
| C19orf69 | -3.32703 | 2.59E-14 | 2.75E-11 |
| GRM8 | -3.32232 | 2.13E-10 | 1.89E-08 |
| ASPG | -3.27964 | 3.99E-09 | 1.96E-07 |
| ECEL1P2 | -3.20378 | 4.79E-14 | 3.8E-11 |
| C11orf9 | -3.13271 | 2.69E-14 | 2.75E-11 |
| PTPRQ | -3.1131 | 1.28E-14 | 1.72E-11 |
| RXFP1 | -3.10042 | 6.14E-11 | 7.15E-09 |
| GPM6A | -3.0585 | 5.77E-14 | 4.42E-11 |
| CHRM1 | -3.04064 | 7.39E-08 | 2.13E-06 |
| GRIA1 | -3.01296 | 1.99E-12 | 5.85E-10 |
| F11 | -2.98774 | 1.39E-09 | 8.32E-08 |
| DPP6 | -2.98304 | 8.69E-09 | 3.68E-07 |
| CYP1A2 | -2.97623 | 8.8E-09 | 3.72E-07 |
| GPIHBP1 | -2.92654 | 4.7E-13 | 1.88E-10 |
| HTR3C | -2.90714 | 0.000165 | 0.001088 |
| CTNND2 | -2.87544 | 8.95E-11 | 9.5E-09 |
| LOC150622 | -2.86579 | 3.23E-06 | 4.49E-05 |
| RPL23AP32 | -2.85231 | 1.1E-06 | 1.89E-05 |
| UPK3B | -2.84655 | 3.71E-09 | 1.85E-07 |
| KLRG2 | -2.84575 | 9.18E-13 | 3.17E-10 |
| LOC285758 | -2.82642 | 3.1E-13 | 1.43E-10 |
| MS4A15 | -2.81358 | 7.97E-12 | 1.62E-09 |
| RGS9BP | -2.81118 | 3.54E-12 | 9.62E-10 |
| LOC554223 | -2.80629 | 1.27E-10 | 1.26E-08 |
| CACNA1S | -2.78472 | 2.3E-07 | 5.3E-06 |
| ZBED2 | -2.7829 | 1.3E-11 | 2.23E-09 |
| VIPR1 | -2.77939 | 1.9E-08 | 7.1E-07 |
| GGTLC1 | -2.77887 | 2.15E-11 | 3.09E-09 |
| AGBL1 | -2.76205 | 5.91E-09 | 2.69E-07 |
| SH3GL3 | -2.75987 | 6.06E-11 | 7.15E-09 |
| CLDN18 | -2.7449 | 1.69E-09 | 9.87E-08 |
| NXF3 | -2.73956 | 1.5E-11 | 2.41E-09 |
| C8B | -2.73956 | 1.45E-06 | 2.37E-05 |
| SLC5A9 | -2.7285 | 7.04E-12 | 1.48E-09 |
| HBA2 | -2.7174 | 2.29E-06 | 3.41E-05 |
| SGCG | -2.68282 | 1.28E-08 | 5.09E-07 |
| CD5L | -2.68127 | 7.43E-08 | 2.13E-06 |
| MYZAP | -2.67021 | 2.3E-10 | 1.99E-08 |
| TNNC1 | -2.65713 | 4.3E-12 | 1.1E-09 |
| GPR158 | -2.64448 | 3E-09 | 1.55E-07 |
| LINC00032 | -2.64269 | 4.56E-11 | 5.76E-09 |
| NMUR1 | -2.63703 | 4.78E-13 | 1.88E-10 |
| CYP3A5 | -2.61778 | 4.02E-10 | 3.1E-08 |
| NR1H4 | -2.60952 | 7.04E-09 | 3.1E-07 |
| CDH10 | -2.60718 | 6.27E-05 | 0.000493 |
| HMGCS2 | -2.60419 | 0.000424 | 0.002373 |
| LHFPL3 | -2.60246 | 1.5E-09 | 8.89E-08 |
| LINC00162 | -2.60219 | 2.52E-08 | 8.98E-07 |
| LRRC36 | -2.59921 | 1.25E-11 | 2.16E-09 |
| ANKRD1 | -2.5945 | 0.000267 | 0.00163 |
| RS1 | -2.59062 | 8.07E-12 | 1.62E-09 |
| LOC400550 | -2.58871 | 7.04E-09 | 3.1E-07 |
| LINC00487 | -2.56727 | 2.16E-06 | 3.24E-05 |
| GNLY | -2.55594 | 1.83E-09 | 1.04E-07 |
| NCKAP5 | -2.54692 | 2.74E-11 | 3.71E-09 |
| CNTN6 | -2.53506 | 6.8E-12 | 1.47E-09 |
| PLAC2 | -2.53267 | 6.64E-13 | 2.41E-10 |
| CHRNA2 | -2.52722 | 6.72E-08 | 1.99E-06 |
| IL1A | -2.50968 | 4.76E-07 | 9.73E-06 |
| KLRF1 | -2.50564 | 8.61E-12 | 1.68E-09 |
| KIAA1683 | -2.50103 | 2.68E-09 | 1.43E-07 |
| HIF3A | -2.49186 | 5.18E-12 | 1.25E-09 |
| EMR1 | -2.48659 | 5.7E-08 | 1.75E-06 |
| TKTL1 | -2.48623 | 1.76E-06 | 2.75E-05 |
| ARC | -2.48266 | 1.63E-07 | 4.05E-06 |
| KRT72 | -2.4794 | 1.75E-06 | 2.74E-05 |
| COL4A3 | -2.4774 | 6.32E-12 | 1.43E-09 |
| S100A3 | -2.47614 | 2.21E-10 | 1.94E-08 |
| RXFP2 | -2.4746 | 5.4E-07 | 1.07E-05 |
| LOC100507632 | -2.47396 | 5.09E-12 | 1.25E-09 |
| CASP12 | -2.46637 | 2.61E-10 | 2.19E-08 |
| COLEC10 | -2.46352 | 5.27E-06 | 6.65E-05 |
| CADM2 | -2.4593 | 1.91E-06 | 2.94E-05 |
| MIR4530 | -2.4577 | 1.89E-07 | 4.52E-06 |
| LOC645591 | -2.4551 | 2.35E-08 | 8.47E-07 |
| PRX | -2.44735 | 3.27E-10 | 2.6E-08 |
| CYS1 | -2.44635 | 1.03E-13 | 6.47E-11 |
| DSCR6 | -2.44264 | 1.24E-08 | 4.96E-07 |
| CAV3 | -2.43492 | 6.58E-08 | 1.95E-06 |
| FAM189A2 | -2.4309 | 1.19E-13 | 7.3E-11 |
| KHDRBS2 | -2.42815 | 4.78E-13 | 1.88E-10 |
| CTSL1P8 | -2.42512 | 2.88E-07 | 6.4E-06 |
| KLK5 | -2.42056 | 0.00787 | 0.025554 |
| WNT3A | -2.41216 | 1.84E-10 | 1.66E-08 |
| FOLR3 | -2.40591 | 1.05E-06 | 1.83E-05 |
| INSC | -2.38072 | 2.31E-09 | 1.27E-07 |
| RAB40A | -2.37542 | 2.3E-13 | 1.2E-10 |
| MME | -2.37224 | 1.15E-11 | 2.02E-09 |
| TMEM100 | -2.37171 | 1.07E-06 | 1.85E-05 |
| HSD17B6 | -2.3645 | 2.97E-12 | 8.39E-10 |
| DHRS2 | -2.36218 | 1.3E-08 | 5.16E-07 |
| IHH | -2.35793 | 1.09E-09 | 6.93E-08 |
| TTPA | -2.35077 | 2.16E-08 | 7.89E-07 |
| FRMPD1 | -2.33747 | 1.69E-10 | 1.55E-08 |
| PNMT | -2.33683 | 5.44E-07 | 1.07E-05 |
| SLC19A3 | -2.32898 | 3E-09 | 1.55E-07 |
| FAM123A | -2.3256 | 0.000708 | 0.003613 |
| LOC100128098 | -2.32505 | 1.65E-08 | 6.24E-07 |
| OVCH1 | -2.32203 | 3.88E-08 | 1.28E-06 |
| KIR2DL3 | -2.32135 | 5.94E-08 | 1.79E-06 |
| SLC22A10 | -2.31783 | 2.79E-08 | 9.68E-07 |
| CCK | -2.31428 | 3.91E-05 | 0.000336 |
| LOC375010 | -2.31411 | 7.04E-10 | 4.9E-08 |
| ANKRD29 | -2.30471 | 2.67E-15 | 6.35E-12 |
| DAPK2 | -2.30309 | 1.17E-15 | 4.19E-12 |
| NRG3 | -2.29452 | 7.62E-08 | 2.16E-06 |
| UNC80 | -2.28588 | 1.89E-11 | 2.84E-09 |
| PTPN5 | -2.2803 | 1.19E-06 | 2.01E-05 |
| EDN3 | -2.27604 | 8.88E-06 | 0.0001 |
| LOC283089 | -2.26818 | 7.39E-10 | 5.11E-08 |
| SH2D1B | -2.26651 | 1.68E-07 | 4.16E-06 |
| O3FAR1 | -2.26004 | 2.88E-09 | 1.52E-07 |
| OVCH2 | -2.25737 | 2.44E-09 | 1.33E-07 |
| CX3CR1 | -2.25639 | 6.9E-09 | 3.06E-07 |
| SYCP2L | -2.25569 | 5.24E-11 | 6.34E-09 |
| RBP2 | -2.25435 | 2.3E-07 | 5.3E-06 |
| ACADL | -2.25161 | 5.1E-13 | 1.92E-10 |
| FAM189A1 | -2.24422 | 1.05E-10 | 1.07E-08 |
| CYP3A7 | -2.24197 | 2.88E-07 | 6.4E-06 |
| EMP2 | -2.24197 | 3.03E-15 | 6.51E-12 |
| HS3ST5 | -2.22899 | 7.21E-06 | 8.49E-05 |
| CCDC48 | -2.22729 | 2.81E-17 | 3.02E-13 |
| ANGPTL7 | -2.21426 | 7.73E-09 | 3.35E-07 |
| GPA33 | -2.21259 | 2.59E-05 | 0.000241 |
| MYO7B | -2.21111 | 5.4E-10 | 3.99E-08 |
| CXCL2 | -2.21007 | 4.63E-06 | 6.02E-05 |
| CRTAC1 | -2.20802 | 6.23E-10 | 4.49E-08 |
| EXD1 | -2.20738 | 2.53E-06 | 3.67E-05 |
| HPGD | -2.2038 | 1.68E-08 | 6.35E-07 |
| MAG | -2.20117 | 0.000321 | 0.0019 |
| C19orf59 | -2.19155 | 2.15E-07 | 5.03E-06 |
| CLIC3 | -2.19134 | 5.09E-11 | 6.29E-09 |
| RXRG | -2.19036 | 5.22E-11 | 6.34E-09 |
| SSTR1 | -2.19015 | 2.29E-08 | 8.31E-07 |
| CELA2A | -2.18565 | 3.07E-07 | 6.72E-06 |
| ABCC13 | -2.18557 | 2.81E-07 | 6.31E-06 |
| KCTD19 | -2.18489 | 6.63E-11 | 7.65E-09 |
| ADRA1A | -2.18461 | 5.8E-07 | 1.13E-05 |
| ODAM | -2.18308 | 9.67E-09 | 4.03E-07 |
| FAM167A | -2.18022 | 1.4E-11 | 2.34E-09 |
| MST1P9 | -2.17917 | 1.72E-09 | 1E-07 |
| HELT | -2.16661 | 3.25E-06 | 4.51E-05 |
| FOS | -2.16379 | 1.32E-07 | 3.42E-06 |
| BTNL3 | -2.1634 | 0.001513 | 0.006672 |
| FLJ35282 | -2.16219 | 1.3E-07 | 3.37E-06 |
| CD300LG | -2.15384 | 1.82E-06 | 2.82E-05 |
| ADRB1 | -2.14423 | 6.39E-09 | 2.88E-07 |
| POPDC3 | -2.13817 | 6.04E-07 | 1.17E-05 |
| ENHO | -2.13726 | 3.74E-07 | 7.91E-06 |
| AGRP | -2.13391 | 9.13E-09 | 3.84E-07 |
| NCR1 | -2.13223 | 9.88E-10 | 6.42E-08 |
| LOC285441 | -2.12851 | 9.55E-09 | 3.99E-07 |
| CA2 | -2.12348 | 2.32E-10 | 1.99E-08 |
| GPR17 | -2.11429 | 2.77E-08 | 9.64E-07 |
| BDNF | -2.11379 | 2.85E-09 | 1.5E-07 |
| LOC200261 | -2.11302 | 6.62E-06 | 7.93E-05 |
| LTK | -2.11207 | 3.42E-09 | 1.72E-07 |
| ALPPL2 | -2.11139 | 2.06E-06 | 3.11E-05 |
| BTNL8 | -2.10721 | 2.81E-09 | 1.49E-07 |
| LOC441177 | -2.10438 | 0.003338 | 0.012714 |
| LRRN3 | -2.1023 | 7.92E-11 | 8.66E-09 |
| C10orf116 | -2.1005 | 2.59E-15 | 6.35E-12 |
| CLIC5 | -2.09884 | 7.23E-11 | 8.07E-09 |
| USHBP1 | -2.09791 | 8.04E-14 | 5.75E-11 |
| CLEC4F | -2.09589 | 5.5E-07 | 1.08E-05 |
| SLITRK2 | -2.09394 | 2.66E-06 | 3.83E-05 |
| ANXA3 | -2.0939 | 1.71E-11 | 2.64E-09 |
| LRRTM4 | -2.0919 | 1.39E-07 | 3.57E-06 |
| LOC283299 | -2.09094 | 4.09E-09 | 1.99E-07 |
| ANGPT4 | -2.08984 | 4.33E-08 | 1.4E-06 |
| ZNF683 | -2.08676 | 1.51E-05 | 0.000156 |
| LOC390660 | -2.08209 | 5.33E-07 | 1.06E-05 |
| KLRC3 | -2.08119 | 2.18E-07 | 5.09E-06 |
| SNX22 | -2.0763 | 3.8E-11 | 4.91E-09 |
| AGAP11 | -2.0727 | 1.57E-14 | 1.98E-11 |
| TMIE | -2.06515 | 1.51E-12 | 4.69E-10 |
| LOC100507091 | -2.06425 | 2.35E-10 | 2.01E-08 |
| C21orf90 | -2.06223 | 2.98E-08 | 1.02E-06 |
| MIR23A | -2.06209 | 1.01E-05 | 0.00011 |
| GUCA2A | -2.06041 | 0.000127 | 0.000878 |
| SFTPC | -2.059 | 2.13E-08 | 7.81E-07 |
| AFF3 | -2.05543 | 5.11E-15 | 9.13E-12 |
| VIP | -2.05154 | 1.16E-06 | 1.97E-05 |
| LOC723809 | -2.04981 | 6.26E-08 | 1.86E-06 |
| GPD1 | -2.04925 | 7.09E-07 | 1.33E-05 |
| PRKG2 | -2.04897 | 1.07E-08 | 4.4E-07 |
| SLC5A4 | -2.04518 | 9.66E-11 | 1.02E-08 |
| HULC | -2.03844 | 4.95E-05 | 0.000406 |
| CAMP | -2.0318 | 6.47E-07 | 1.24E-05 |
| LOC643733 | -2.02983 | 2.59E-11 | 3.55E-09 |
| AGTR2 | -2.0246 | 0.004449 | 0.016121 |
| LRRC19 | -2.02181 | 2.43E-09 | 1.32E-07 |
| NDRG4 | -2.01905 | 4.15E-09 | 2.01E-07 |
| GRID2 | -2.01215 | 0.00012 | 0.000842 |
| SLC10A4 | -2.00697 | 2.08E-05 | 0.0002 |
| STXBP6 | -2.00187 | 1.34E-08 | 5.25E-07 |
| C12orf39 | -1.9994 | 1.45E-06 | 2.37E-05 |
| TMEM88 | -1.99922 | 1.28E-09 | 7.84E-08 |
| GYPE | -1.99917 | 5.2E-10 | 3.88E-08 |
| TCAP | -1.99535 | 6.55E-10 | 4.65E-08 |
| PLLP | -1.9923 | 2.08E-11 | 3.06E-09 |
| BEX1 | -1.98635 | 1.54E-08 | 5.85E-07 |
| TMSB15A | -1.98322 | 6.68E-06 | 7.99E-05 |
| LOC400940 | -1.98321 | 0.007864 | 0.025542 |
| SPOCK2 | -1.983 | 4.4E-09 | 2.11E-07 |
| PRSS21 | -1.97922 | 0.000174 | 0.001137 |
| TNF | -1.97858 | 1.33E-05 | 0.000139 |
| LOC147646 | -1.97794 | 7.38E-08 | 2.12E-06 |
| TMEM74B | -1.97711 | 1.41E-11 | 2.34E-09 |
| SRRM4 | -1.97155 | 3.11E-07 | 6.79E-06 |
| FGF17 | -1.97135 | 3.94E-06 | 5.29E-05 |
| KLF4 | -1.97003 | 1.31E-10 | 1.29E-08 |
| LIFR | -1.96614 | 5.72E-12 | 1.36E-09 |
| SDPR | -1.96119 | 7.08E-11 | 7.99E-09 |
| LOC646324 | -1.95208 | 7.4E-13 | 2.6E-10 |
| UPB1 | -1.95046 | 9.98E-10 | 6.46E-08 |
| DAO | -1.94956 | 7.6E-06 | 8.87E-05 |
| EPB41L5 | -1.94858 | 3.39E-19 | 7.27E-15 |
| ESM1 | -1.94193 | 0.004468 | 0.01617 |
| NKG7 | -1.94016 | 1.19E-09 | 7.42E-08 |
| CLEC6A | -1.93648 | 0.000593 | 0.003125 |
| LOC643650 | -1.93567 | 9E-15 | 1.38E-11 |
| KIR2DS4 | -1.93387 | 0.001465 | 0.006494 |
| SCEL | -1.93331 | 1.81E-07 | 4.39E-06 |
| HRCT1 | -1.93182 | 2.55E-05 | 0.000238 |
| GZMH | -1.93145 | 1.07E-06 | 1.86E-05 |
| AATK | -1.93049 | 1.21E-11 | 2.11E-09 |
| PBOV1 | -1.92862 | 1.66E-06 | 2.63E-05 |
| RNASE13 | -1.92634 | 3.28E-07 | 7.09E-06 |
| EFR3B | -1.92346 | 4.98E-13 | 1.91E-10 |
| FREM3 | -1.92216 | 0.000247 | 0.001527 |
| KCNA4 | -1.92075 | 2.41E-06 | 3.55E-05 |
| DCC | -1.91896 | 3.08E-07 | 6.75E-06 |
| MATN3 | -1.91748 | 6.37E-10 | 4.55E-08 |
| HBEGF | -1.91493 | 3.84E-06 | 5.18E-05 |
| ATP8B5P | -1.9124 | 7.89E-10 | 5.37E-08 |
| CD244 | -1.90713 | 2.44E-10 | 2.07E-08 |
| KIR2DL1 | -1.89857 | 3.91E-05 | 0.000336 |
| LOC100144604 | -1.89697 | 6.18E-07 | 1.19E-05 |
| C6orf174 | -1.88912 | 1.14E-08 | 4.67E-07 |
| SEMA6D | -1.8891 | 2.67E-10 | 2.22E-08 |
| ST8SIA6 | -1.88829 | 7.38E-07 | 1.38E-05 |
| KIAA0087 | -1.88802 | 2.23E-07 | 5.17E-06 |
| CCDC85A | -1.8863 | 3.7E-10 | 2.89E-08 |
| SEMA6A | -1.88514 | 2.22E-13 | 1.19E-10 |
| KLRD1 | -1.88428 | 1.24E-08 | 4.96E-07 |
| RFX6 | -1.88127 | 3.83E-05 | 0.00033 |
| FABP4 | -1.8751 | 3.82E-06 | 5.17E-05 |
| ARHGEF26 | -1.87358 | 2.12E-11 | 3.09E-09 |
| SLC27A6 | -1.87306 | 2.31E-05 | 0.000218 |
| VTRNA1-3 | -1.87303 | 2.16E-06 | 3.24E-05 |
| TRPC3 | -1.8703 | 3.16E-10 | 2.54E-08 |
| C11orf35 | -1.86986 | 2.1E-13 | 1.15E-10 |
| C10orf67 | -1.86906 | 1.63E-07 | 4.05E-06 |
| MIR30C2 | -1.86615 | 1.45E-07 | 3.69E-06 |
| CAV1 | -1.86513 | 1.65E-12 | 5.05E-10 |
| SYT15 | -1.86478 | 7.75E-14 | 5.73E-11 |
| CXorf65 | -1.86396 | 1.22E-05 | 0.00013 |
| SULT1A2 | -1.86308 | 1.33E-10 | 1.3E-08 |
| GNG8 | -1.86261 | 5.06E-06 | 6.45E-05 |
| OASL | -1.86249 | 2.88E-07 | 6.4E-06 |
| SLC13A2 | -1.86019 | 0.003034 | 0.01174 |
| MPP3 | -1.85948 | 8.16E-10 | 5.5E-08 |
| UNC5D | -1.8569 | 5.91E-05 | 0.00047 |
| FCRL6 | -1.85565 | 6.06E-12 | 1.43E-09 |
| RFPL3 | -1.85459 | 1.38E-08 | 5.39E-07 |
| LGALS4 | -1.85285 | 1.53E-09 | 9.05E-08 |
| WNT7A | -1.85036 | 4.21E-09 | 2.04E-07 |
| NOS2 | -1.84985 | 2.21E-05 | 0.000211 |
| CXorf1 | -1.84948 | 0.000914 | 0.00444 |
| C20orf160 | -1.84618 | 4.59E-15 | 8.96E-12 |
| NLRP9 | -1.84541 | 4.43E-06 | 5.82E-05 |
| LOC254312 | -1.84531 | 8.29E-06 | 9.48E-05 |
| ZNF541 | -1.84441 | 1.56E-10 | 1.44E-08 |
| SERPINA10 | -1.84345 | 3.18E-07 | 6.92E-06 |
| PLA2G12B | -1.84042 | 0.000208 | 0.001323 |
| APOA1 | -1.83991 | 1.05E-06 | 1.84E-05 |
| NECAB1 | -1.83948 | 3.65E-14 | 3.26E-11 |
| FABP12 | -1.83741 | 0.009483 | 0.029777 |
| PSAPL1 | -1.83606 | 8.13E-06 | 9.34E-05 |
| CSF3 | -1.83603 | 0.011567 | 0.034794 |
| SYNPO2L | -1.83494 | 2.68E-07 | 6.07E-06 |
| IZUMO1 | -1.83311 | 5.32E-06 | 6.69E-05 |
| PF4 | -1.83222 | 0.003094 | 0.011929 |
| CCDC68 | -1.83109 | 2.32E-10 | 1.99E-08 |
| CLEC3B | -1.83097 | 1.36E-09 | 8.24E-08 |
| MIR126 | -1.83032 | 6.4E-07 | 1.23E-05 |
| ZNF385B | -1.82914 | 6.92E-10 | 4.87E-08 |
| FLJ33360 | -1.8275 | 4.85E-06 | 6.23E-05 |
| LOC644242 | -1.82435 | 8.91E-08 | 2.44E-06 |
| LOC283867 | -1.82246 | 4.46E-08 | 1.42E-06 |
| VSIG10 | -1.82199 | 2.74E-16 | 1.18E-12 |
| FCN3 | -1.82187 | 0.000139 | 0.000949 |
| KCNK17 | -1.82183 | 1.08E-07 | 2.87E-06 |
| FIGF | -1.81978 | 7.38E-05 | 0.000565 |
| CXCL3 | -1.81445 | 1.33E-05 | 0.000139 |
| LOC158376 | -1.81353 | 5.8E-09 | 2.65E-07 |
| RXFP4 | -1.81314 | 5.27E-05 | 0.000428 |
| SHISA3 | -1.8129 | 8.84E-07 | 1.6E-05 |
| KANK4 | -1.81262 | 3.25E-07 | 7.03E-06 |
| HIGD1B | -1.80805 | 1.17E-09 | 7.33E-08 |
| CPB2 | -1.80743 | 0.000148 | 0.001 |
| TDRD10 | -1.80539 | 2.86E-11 | 3.83E-09 |
| RGS9 | -1.80416 | 1.49E-07 | 3.76E-06 |
| KLKB1 | -1.80407 | 6.91E-06 | 8.2E-05 |
| RETN | -1.8011 | 0.000213 | 0.001347 |
| PLA2G1B | -1.79857 | 6.66E-05 | 0.000517 |
| IGFALS | -1.7975 | 9.16E-12 | 1.76E-09 |
| PRSS57 | -1.79731 | 7.52E-06 | 8.8E-05 |
| CHRM2 | -1.79559 | 0.009718 | 0.030321 |
| PDE6A | -1.79275 | 9.04E-09 | 3.81E-07 |
| LOC284080 | -1.7909 | 3.84E-13 | 1.68E-10 |
| AVPR2 | -1.78973 | 5.4E-09 | 2.52E-07 |
| PRKAG3 | -1.78968 | 0.00032 | 0.001892 |
| LOC643669 | -1.78777 | 1.16E-06 | 1.98E-05 |
| EGR2 | -1.78473 | 5.46E-07 | 1.08E-05 |
| KLF2 | -1.7827 | 3.02E-11 | 4E-09 |
| TNNT1 | -1.78267 | 9.5E-05 | 0.000692 |
| PCDH12 | -1.78023 | 2.3E-09 | 1.27E-07 |
| CYP4A11 | -1.77757 | 3.33E-07 | 7.18E-06 |
| LINC00312 | -1.77696 | 9.82E-09 | 4.09E-07 |
| LEPREL1 | -1.77516 | 1.14E-10 | 1.15E-08 |
| CRH | -1.7742 | 0.005923 | 0.020326 |
| RGS16 | -1.77413 | 1.45E-07 | 3.69E-06 |
| KIAA0408 | -1.77389 | 1.67E-05 | 0.000169 |
| PTCRA | -1.771 | 2.11E-05 | 0.000203 |
| XCL1 | -1.77098 | 4.15E-08 | 1.35E-06 |
| NIM1 | -1.76885 | 1.1E-11 | 1.98E-09 |
| SLC39A8 | -1.7676 | 1.42E-08 | 5.47E-07 |
| GSG1L | -1.76695 | 6.14E-05 | 0.000484 |
| APLN | -1.76339 | 0.003413 | 0.01297 |
| LOC729041 | -1.76246 | 5.71E-06 | 7.08E-05 |
| CCDC54 | -1.76189 | 5.77E-06 | 7.15E-05 |
| SCN1A | -1.76129 | 5.39E-07 | 1.07E-05 |
| C11orf21 | -1.75686 | 1.74E-08 | 6.52E-07 |
| KRT73 | -1.75399 | 3.82E-05 | 0.00033 |
| FUT1 | -1.75345 | 2.55E-10 | 2.15E-08 |
| OLR1 | -1.74939 | 7.31E-06 | 8.58E-05 |
| C1orf140 | -1.74681 | 2.32E-07 | 5.34E-06 |
| RAMP2 | -1.74515 | 2.23E-10 | 1.95E-08 |
| MOGAT1 | -1.74421 | 3.32E-05 | 0.000294 |
| C3orf45 | -1.73968 | 3.23E-06 | 4.5E-05 |
| SH3GL2 | -1.73896 | 3.33E-06 | 4.59E-05 |
| CCRL1 | -1.73774 | 6.73E-11 | 7.72E-09 |
| ADARB2 | -1.73658 | 1.11E-06 | 1.91E-05 |
| EMILIN3 | -1.73537 | 9.92E-08 | 2.67E-06 |
| HEY1 | -1.73488 | 1.07E-08 | 4.41E-07 |
| SH2D4B | -1.73351 | 7.43E-08 | 2.13E-06 |
| KLRC2 | -1.73225 | 9.2E-06 | 0.000103 |
| HHIP | -1.72678 | 4.71E-06 | 6.07E-05 |
| CXCR2P1 | -1.72391 | 0.000191 | 0.001229 |
| KAL1 | -1.72338 | 7.31E-09 | 3.19E-07 |
| ABCA10 | -1.72196 | 1.96E-08 | 7.27E-07 |
| LOC283683 | -1.72152 | 7.49E-08 | 2.14E-06 |
| FXYD4 | -1.71915 | 3.06E-05 | 0.000274 |
| LOC645434 | -1.71714 | 5.39E-07 | 1.07E-05 |
| CYP4A22 | -1.71592 | 1.03E-06 | 1.81E-05 |
| LOC284454 | -1.71403 | 2.63E-05 | 0.000244 |
| BMP2 | -1.7132 | 1.77E-07 | 4.33E-06 |
| MYO1A | -1.7105 | 5.25E-08 | 1.64E-06 |
| PER3 | -1.71044 | 1.94E-07 | 4.65E-06 |
| LOC91450 | -1.70739 | 3.94E-08 | 1.3E-06 |
| SLC1A1 | -1.70036 | 1.82E-11 | 2.77E-09 |
| SLC30A8 | -1.6999 | 0.007309 | 0.024077 |
| LY6G6C | -1.69988 | 0.00033 | 0.001937 |
| FAM13A-AS1 | -1.69668 | 7.04E-10 | 4.9E-08 |
| WFDC12 | -1.69336 | 0.000283 | 0.001709 |
| FAM107A | -1.69318 | 2.61E-05 | 0.000242 |
| FGF12 | -1.69192 | 9.7E-08 | 2.62E-06 |
| CALCR | -1.69159 | 9.46E-07 | 1.69E-05 |
| PEBP4 | -1.68878 | 4.43E-08 | 1.42E-06 |
| IFNG | -1.68867 | 0.000979 | 0.004696 |
| MFAP3L | -1.68301 | 1.02E-12 | 3.46E-10 |
| GPRC5D | -1.68097 | 5.08E-07 | 1.02E-05 |
| SEMA3E | -1.6792 | 1.96E-07 | 4.68E-06 |
| FBN3 | -1.67454 | 1.12E-05 | 0.000121 |
| FLJ42875 | -1.67295 | 3.31E-12 | 9.11E-10 |
| OLFML2A | -1.67263 | 1.13E-09 | 7.17E-08 |
| NR4A1 | -1.67093 | 0.000334 | 0.001961 |
| NOTCH4 | -1.67091 | 1.5E-12 | 4.69E-10 |
| HYAL1 | -1.66828 | 1.63E-11 | 2.55E-09 |
| HPCAL4 | -1.66708 | 1.09E-05 | 0.000118 |
| MIR29B2 | -1.66546 | 9.6E-05 | 0.000698 |
| WWC2 | -1.66473 | 7.97E-11 | 8.67E-09 |
| SYT1 | -1.66165 | 6.2E-08 | 1.85E-06 |
| TAL1 | -1.66118 | 4.22E-11 | 5.38E-09 |
| STARD9 | -1.66047 | 4.39E-13 | 1.88E-10 |
| EDNRB | -1.6597 | 2.41E-09 | 1.32E-07 |
| LOC100652759 | -1.65667 | 8.17E-06 | 9.37E-05 |
| MIR657 | -1.6563 | 1.15E-05 | 0.000124 |
| WFIKKN1 | -1.65541 | 1.29E-07 | 3.36E-06 |
| VEGFA | -1.65202 | 6.85E-11 | 7.82E-09 |
| MIR3677 | -1.65119 | 0.000296 | 0.001773 |
| LOC90246 | -1.65087 | 3.44E-10 | 2.72E-08 |
| ACE | -1.64962 | 4.17E-10 | 3.2E-08 |
| DKFZP434K028 | -1.64883 | 0.000906 | 0.004404 |
| LOXHD1 | -1.64855 | 1.3E-09 | 7.91E-08 |
| EXOC3L1 | -1.64834 | 1.48E-10 | 1.39E-08 |
| EMR3 | -1.64829 | 2.46E-05 | 0.000231 |
| C2orf72 | -1.64731 | 9.66E-08 | 2.61E-06 |
| LOC100190938 | -1.64695 | 1.43E-10 | 1.38E-08 |
| GPRIN2 | -1.64623 | 3.79E-09 | 1.88E-07 |
| HECW2 | -1.64506 | 5.8E-06 | 7.18E-05 |
| LGI3 | -1.64502 | 3.72E-06 | 5.05E-05 |
| JPH1 | -1.64429 | 4.36E-14 | 3.74E-11 |
| CD160 | -1.64343 | 1.46E-11 | 2.41E-09 |
| MGAT3 | -1.64264 | 1.49E-10 | 1.4E-08 |
| TMEM139 | -1.64246 | 1.32E-08 | 5.22E-07 |
| OTOF | -1.64041 | 0.002111 | 0.008748 |
| MCOLN3 | -1.63399 | 1.37E-09 | 8.24E-08 |
| ASB11 | -1.63284 | 4.65E-07 | 9.52E-06 |
| CAV2 | -1.63183 | 2.02E-11 | 3.01E-09 |
| FATE1 | -1.63013 | 1.19E-06 | 2.01E-05 |
| RMST | -1.63 | 0.00014 | 0.000952 |
| LOC100506497 | -1.62945 | 5.12E-07 | 1.03E-05 |
| ADRB2 | -1.62859 | 6.1E-13 | 2.26E-10 |
| C2orf71 | -1.62632 | 8.71E-05 | 0.000646 |
| RNU4ATAC | -1.62476 | 2.41E-05 | 0.000227 |
| KCNMB4 | -1.62464 | 2.53E-08 | 9E-07 |
| AMH | -1.62378 | 9.15E-06 | 0.000103 |
| PLA2G4F | -1.62265 | 8.07E-07 | 1.49E-05 |
| C8orf85 | -1.62186 | 2.7E-05 | 0.000249 |
| LOC284578 | -1.621 | 1.17E-09 | 7.35E-08 |
| GDPD3 | -1.61867 | 2.21E-07 | 5.14E-06 |
| KLB | -1.61652 | 1.94E-05 | 0.00019 |
| FXYD6-FXYD2 | -1.61626 | 8.66E-09 | 3.68E-07 |
| HSPA12B | -1.61614 | 6.35E-12 | 1.43E-09 |
| CSRNP1 | -1.61543 | 7.93E-06 | 9.18E-05 |
| GATA3 | -1.61304 | 5.01E-10 | 3.8E-08 |
| PCDH10 | -1.6115 | 2.59E-05 | 0.000241 |
| B3GALNT1 | -1.61013 | 1.86E-11 | 2.81E-09 |
| IFITM5 | -1.60617 | 6.74E-06 | 8.06E-05 |
| PRSS30P | -1.60505 | 1.81E-06 | 2.81E-05 |
| S1PR5 | -1.60436 | 1.45E-10 | 1.38E-08 |
| CDH12 | -1.6041 | 3.06E-06 | 4.28E-05 |
| ODF3L1 | -1.6018 | 0.000347 | 0.002023 |
| MIR142 | -1.5995 | 2.27E-05 | 0.000216 |
| NOVA2 | -1.5988 | 1.59E-10 | 1.47E-08 |
| TBX21 | -1.59851 | 7.21E-11 | 8.07E-09 |
| DPCR1 | -1.597 | 5.69E-05 | 0.000456 |
| RNF128 | -1.59651 | 6.14E-11 | 7.15E-09 |
| CYP3A4 | -1.59603 | 1.19E-06 | 2.01E-05 |
| PRF1 | -1.59448 | 6.68E-08 | 1.97E-06 |
| SLC17A3 | -1.59413 | 5.32E-05 | 0.000431 |
| KIR3DL1 | -1.59367 | 0.0014 | 0.006263 |
| ST6GALNAC5 | -1.59343 | 1.11E-07 | 2.93E-06 |
| ACR | -1.59318 | 6.19E-09 | 2.8E-07 |
| CABP5 | -1.59271 | 0.000533 | 0.002876 |
| PARD6B | -1.59232 | 1.66E-10 | 1.53E-08 |
| TSPAN12 | -1.59049 | 3.07E-10 | 2.48E-08 |
| CD101 | -1.59047 | 3.72E-06 | 5.05E-05 |
| APOA5 | -1.59039 | 5.24E-05 | 0.000426 |
| NRN1L | -1.58969 | 1.65E-06 | 2.62E-05 |
| LIMS2 | -1.58871 | 6.14E-11 | 7.15E-09 |
| LIM2 | -1.58829 | 0.002735 | 0.01078 |
| LOC399940 | -1.58496 | 0.000208 | 0.00132 |
| EGFL7 | -1.5846 | 1.82E-09 | 1.04E-07 |
| CPAMD8 | -1.58414 | 8.95E-14 | 5.81E-11 |
| PTPN21 | -1.58038 | 8.8E-14 | 5.81E-11 |
| C12orf71 | -1.57886 | 1.37E-07 | 3.51E-06 |
| DSCAM | -1.57849 | 0.000414 | 0.00233 |
| RGS6 | -1.57797 | 7.44E-08 | 2.13E-06 |
| GPER | -1.57738 | 1.54E-06 | 2.49E-05 |
| FAM69B | -1.57582 | 5.1E-11 | 6.29E-09 |
| LOC285954 | -1.57479 | 2.6E-05 | 0.000241 |
| PDZD2 | -1.57424 | 6.41E-12 | 1.43E-09 |
| ZNF385C | -1.57021 | 1.19E-09 | 7.42E-08 |
| PPP1R17 | -1.56963 | 4.05E-05 | 0.000346 |
| TMEFF2 | -1.56825 | 3.8E-05 | 0.000329 |
| PTPRB | -1.56818 | 1.22E-09 | 7.61E-08 |
| KIAA1804 | -1.56527 | 1.79E-12 | 5.32E-10 |
| GBP4 | -1.56463 | 2.67E-06 | 3.84E-05 |
| SPATA21 | -1.56199 | 5.7E-05 | 0.000456 |
| CLEC1B | -1.56188 | 0.001759 | 0.007538 |
| ACOXL | -1.5616 | 2.57E-09 | 1.38E-07 |
| TSPAN32 | -1.56156 | 6.04E-08 | 1.81E-06 |
| C19orf80 | -1.56048 | 1.74E-05 | 0.000174 |
| RORC | -1.55896 | 8.56E-07 | 1.55E-05 |
| DGKK | -1.55747 | 0.009889 | 0.030755 |
| ARRB1 | -1.55743 | 4.46E-13 | 1.88E-10 |
| SMAD6 | -1.5574 | 3.58E-05 | 0.000313 |
| JUN | -1.55671 | 5.03E-09 | 2.37E-07 |
| LOC285972 | -1.55396 | 8.15E-07 | 1.5E-05 |
| SEMA5A | -1.55361 | 8.94E-09 | 3.77E-07 |
| TRPC2 | -1.55136 | 4.42E-07 | 9.12E-06 |
| CACNB4 | -1.55037 | 1.37E-10 | 1.32E-08 |
| CLDN24 | -1.54802 | 1.76E-05 | 0.000176 |
| BFSP1 | -1.54614 | 2.74E-10 | 2.26E-08 |
| MGAT5B | -1.54507 | 9.52E-07 | 1.69E-05 |
| TRIM58 | -1.54385 | 0.000135 | 0.000923 |
| C17orf66 | -1.54315 | 3.88E-05 | 0.000334 |
| CECR2 | -1.54096 | 7.78E-10 | 5.31E-08 |
| A2MP1 | -1.5408 | 3.38E-06 | 4.65E-05 |
| LRRC3B | -1.5407 | 0.003844 | 0.0143 |
| NPFF | -1.54025 | 3.14E-05 | 0.00028 |
| STX11 | -1.53875 | 2.13E-09 | 1.19E-07 |
| RIMKLA | -1.53532 | 3.2E-10 | 2.56E-08 |
| LOC100507156 | -1.53406 | 9.5E-10 | 6.25E-08 |
| CLDND2 | -1.53314 | 4.74E-09 | 2.24E-07 |
| LINC00472 | -1.53173 | 8.61E-09 | 3.67E-07 |
| QRICH2 | -1.5303 | 2.57E-09 | 1.38E-07 |
| PLAG1 | -1.53022 | 1.32E-13 | 7.69E-11 |
| GRAMD2 | -1.52874 | 8E-12 | 1.62E-09 |
| LOC154449 | -1.52832 | 0.010955 | 0.033351 |
| LOC643201 | -1.5264 | 0.000134 | 0.000917 |
| STC1 | -1.52621 | 0.001982 | 0.008302 |
| DISP1 | -1.5228 | 3.35E-11 | 4.41E-09 |
| SLC9A3R2 | -1.52205 | 1.03E-11 | 1.9E-09 |
| MBL1P | -1.52164 | 1.28E-06 | 2.15E-05 |
| DACH1 | -1.51857 | 4.76E-10 | 3.62E-08 |
| LMO7 | -1.51772 | 1.33E-10 | 1.3E-08 |
| DNASE1L3 | -1.51735 | 2.62E-05 | 0.000243 |
| TSPEAR | -1.51711 | 9.85E-07 | 1.74E-05 |
| SLC24A4 | -1.51643 | 5.47E-10 | 4.01E-08 |
| ALS2CL | -1.51523 | 8.54E-12 | 1.68E-09 |
| CRYBB2 | -1.51298 | 0.000171 | 0.001118 |
| PALM3 | -1.51213 | 0.000566 | 0.003009 |
| LAMA3 | -1.51179 | 2.51E-11 | 3.52E-09 |
| HOXD-AS1 | -1.51173 | 9.15E-10 | 6.08E-08 |
| KL | -1.50686 | 6.56E-07 | 1.25E-05 |
| SUSD2 | -1.50628 | 2.45E-09 | 1.33E-07 |
| OTUD1 | -1.5052 | 8.94E-11 | 9.5E-09 |
| FAM105A | -1.50476 | 1.89E-08 | 7.04E-07 |
| CIB4 | -1.50151 | 4.38E-05 | 0.000369 |
| CD83 | -1.5013 | 2.28E-05 | 0.000217 |
| LOC732275 | -1.5 | 0.011554 | 0.034774 |
| CLEC12B | -1.49852 | 3.9E-06 | 5.25E-05 |
| RFX4 | -1.49695 | 1.23E-05 | 0.00013 |
| OBSCN | -1.49614 | 1.14E-12 | 3.7E-10 |
| MAFA | -1.49542 | 5.82E-05 | 0.000464 |
| MIR940 | -1.4938 | 0.000133 | 0.000914 |
| AQP4 | -1.49346 | 1.26E-06 | 2.11E-05 |
| SH2D3C | -1.49325 | 3.74E-10 | 2.91E-08 |
| SLC22A8 | -1.49325 | 0.00153 | 0.006728 |
| C12orf54 | -1.49277 | 1.76E-06 | 2.75E-05 |
| CARD16 | -1.49245 | 1.24E-09 | 7.68E-08 |
| FOXD4L1 | -1.492 | 0.000294 | 0.001765 |
| C10orf129 | -1.49151 | 0.000962 | 0.004625 |
| PION | -1.49099 | 2.65E-13 | 1.29E-10 |
| SNORA20 | -1.48863 | 2.1E-05 | 0.000202 |
| XAF1 | -1.48827 | 6.8E-09 | 3.02E-07 |
| SFTA1P | -1.48727 | 4.37E-08 | 1.41E-06 |
| KANK3 | -1.48517 | 2.72E-11 | 3.71E-09 |
| APOH | -1.48493 | 0.000343 | 0.002007 |
| OPRK1 | -1.48422 | 0.000951 | 0.004584 |
| CYP4Z1 | -1.48411 | 1.7E-05 | 0.000171 |
| GOLGA8B | -1.48351 | 4.18E-06 | 5.56E-05 |
| TREM1 | -1.48229 | 9.99E-06 | 0.00011 |
| LOC731424 | -1.47996 | 0.002125 | 0.008799 |
| HTR1D | -1.47971 | 1.74E-05 | 0.000174 |
| LHX9 | -1.47953 | 0.013144 | 0.038512 |
| HBB | -1.47867 | 0.006208 | 0.021076 |
| LOC400238 | -1.47814 | 0.000931 | 0.004511 |
| PCP2 | -1.47753 | 2.7E-06 | 3.87E-05 |
| DDC | -1.47011 | 0.000114 | 0.000805 |
| FGF9 | -1.4701 | 6.08E-06 | 7.43E-05 |
| KIF12 | -1.46836 | 2.7E-07 | 6.1E-06 |
| ATF3 | -1.46833 | 0.000687 | 0.003528 |
| TACR3 | -1.46745 | 0.003812 | 0.014208 |
| ARHGEF15 | -1.46722 | 3.74E-12 | 9.91E-10 |
| LOC100652999 | -1.4648 | 3.74E-07 | 7.92E-06 |
| ZMAT1 | -1.46255 | 6.75E-10 | 4.78E-08 |
| LINC00310 | -1.4618 | 1.26E-07 | 3.3E-06 |
| FAM162B | -1.46122 | 3.05E-09 | 1.57E-07 |
| CACNG4 | -1.46064 | 1.64E-06 | 2.62E-05 |
| TARP | -1.45798 | 1.44E-06 | 2.36E-05 |
| CEACAM19 | -1.45738 | 1.29E-06 | 2.16E-05 |
| LOC100505865 | -1.45582 | 2.25E-10 | 1.96E-08 |
| DDX43 | -1.45539 | 0.000416 | 0.002338 |
| SERPINA9 | -1.45537 | 0.008766 | 0.027883 |
| MIR29C | -1.45356 | 3.72E-06 | 5.05E-05 |
| SELENBP1 | -1.45341 | 1.74E-13 | 9.81E-11 |
| USP32P2 | -1.45203 | 0.001326 | 0.005994 |
| TMEM26 | -1.44582 | 2.84E-05 | 0.000259 |
| SCAI | -1.44518 | 2.7E-14 | 2.75E-11 |
| NTNG1 | -1.44378 | 2.76E-06 | 3.95E-05 |
| LOC284950 | -1.44321 | 7.92E-06 | 9.17E-05 |
| SYN2 | -1.44309 | 5.14E-07 | 1.03E-05 |
| FIBIN | -1.44261 | 3.09E-05 | 0.000277 |
| HPCAL1 | -1.44189 | 5.09E-11 | 6.29E-09 |
| GALNT8 | -1.44084 | 1.43E-06 | 2.35E-05 |
| CACNA2D2 | -1.44034 | 3.48E-08 | 1.18E-06 |
| MRS2P2 | -1.43991 | 9.21E-05 | 0.000676 |
| VSIG2 | -1.43938 | 7.73E-11 | 8.51E-09 |
| GRB14 | -1.43806 | 3.71E-06 | 5.05E-05 |
| LPPR1 | -1.43389 | 3.49E-06 | 4.77E-05 |
| SFTA2 | -1.43261 | 6.07E-07 | 1.17E-05 |
| NOSTRIN | -1.43259 | 5.31E-10 | 3.94E-08 |
| C5orf4 | -1.43151 | 3.77E-11 | 4.9E-09 |
| SULT1A1 | -1.43088 | 7.31E-09 | 3.19E-07 |
| C13orf15 | -1.43083 | 1.49E-07 | 3.76E-06 |
| LOC100128420 | -1.42962 | 8.86E-06 | 0.0001 |
| EMCN | -1.42858 | 2.78E-08 | 9.67E-07 |
| PNPLA7 | -1.42775 | 4.04E-09 | 1.97E-07 |
| TMEM191B | -1.42658 | 0.000649 | 0.003375 |
| LOC100127888 | -1.42604 | 0.002642 | 0.0105 |
| TMCO2 | -1.42601 | 0.003966 | 0.014663 |
| SHANK3 | -1.42512 | 5.12E-12 | 1.25E-09 |
| SIRPB1 | -1.4226 | 0.000112 | 0.000794 |
| KLF14 | -1.42016 | 2.79E-05 | 0.000255 |
| GUSBP3 | -1.41965 | 5.85E-07 | 1.14E-05 |
| SULT2B1 | -1.41916 | 2.82E-06 | 4.01E-05 |
| LST1 | -1.41772 | 7.33E-08 | 2.12E-06 |
| IL17RE | -1.41704 | 1.14E-11 | 2.02E-09 |
| C6orf25 | -1.41475 | 2.7E-05 | 0.000248 |
| WFDC10B | -1.41414 | 0.00018 | 0.001167 |
| ART1 | -1.4135 | 0.001045 | 0.004931 |
| EGR1 | -1.41258 | 0.000239 | 0.00149 |
| CACNA1F | -1.41077 | 2.66E-09 | 1.43E-07 |
| C4orf38 | -1.40983 | 1.41E-06 | 2.32E-05 |
| RAMP3 | -1.40973 | 1.56E-06 | 2.51E-05 |
| SOX17 | -1.40925 | 3.52E-07 | 7.51E-06 |
| GRASP | -1.40919 | 4.32E-07 | 8.97E-06 |
| KCNG3 | -1.40818 | 3.34E-05 | 0.000295 |
| NRADDP | -1.40736 | 5.86E-05 | 0.000466 |
| PTGDR | -1.4068 | 1.44E-06 | 2.35E-05 |
| KCNK16 | -1.40666 | 0.000189 | 0.001215 |
| TNNT2 | -1.40644 | 1E-05 | 0.00011 |
| GABRE | -1.40635 | 5.24E-07 | 1.05E-05 |
| SFTPD | -1.40596 | 4.96E-07 | 1E-05 |
| IL1B | -1.40473 | 0.000558 | 0.002978 |
| ADCYAP1R1 | -1.40462 | 1.5E-05 | 0.000155 |
| C15orf5 | -1.40459 | 3.98E-05 | 0.000341 |
| MIRLET7D | -1.40383 | 2.45E-05 | 0.00023 |
| DENND3 | -1.40272 | 2.54E-13 | 1.27E-10 |
| MYO15B | -1.40109 | 3.14E-09 | 1.61E-07 |
| RNF182 | -1.39847 | 4.39E-08 | 1.42E-06 |
| IER2 | -1.39803 | 1.52E-06 | 2.46E-05 |
| ARAP3 | -1.3971 | 8.26E-12 | 1.64E-09 |
| TMEM178 | -1.39691 | 2.26E-06 | 3.36E-05 |
| C14orf55 | -1.39651 | 0.000112 | 0.000795 |
| GOLGA8A | -1.39614 | 0.000215 | 0.001361 |
| PLEKHH2 | -1.3961 | 1.05E-08 | 4.33E-07 |
| TUBB1 | -1.39553 | 0.000287 | 0.001727 |
| SAMD3 | -1.39377 | 5.01E-08 | 1.58E-06 |
| PRTG | -1.39302 | 6.88E-07 | 1.3E-05 |
| GABRB2 | -1.39288 | 0.000113 | 0.000801 |
| C9orf170 | -1.39263 | 6.61E-05 | 0.000514 |
| INMT | -1.39185 | 1.01E-07 | 2.7E-06 |
| DPEP2 | -1.39143 | 9.18E-12 | 1.76E-09 |
| DLC1 | -1.39133 | 3.83E-08 | 1.27E-06 |
| KIAA1984 | -1.38857 | 2.13E-11 | 3.09E-09 |
| ROBO4 | -1.38736 | 7.53E-08 | 2.14E-06 |
| DUSP1 | -1.38703 | 3.22E-06 | 4.48E-05 |
| PCSK9 | -1.38666 | 1.62E-06 | 2.6E-05 |
| BET3L | -1.38423 | 1.25E-05 | 0.000132 |
| CETP | -1.3842 | 8.52E-07 | 1.55E-05 |
| LINGO4 | -1.3823 | 0.000696 | 0.003566 |
| ATHL1 | -1.38219 | 2.21E-05 | 0.000211 |
| PTPRR | -1.37976 | 0.000139 | 0.000946 |
| MSH4 | -1.37898 | 0.000139 | 0.000951 |
| BCYRN1 | -1.37851 | 0.000133 | 0.000912 |
| KLF6 | -1.37778 | 1.49E-07 | 3.76E-06 |
| MYL1 | -1.3774 | 0.000187 | 0.001204 |
| C7orf45 | -1.37726 | 0.006203 | 0.021065 |
| LOC441204 | -1.37718 | 1.42E-07 | 3.64E-06 |
| KLRC4 | -1.37491 | 5.96E-05 | 0.000472 |
| IMPG1 | -1.3749 | 5.95E-07 | 1.15E-05 |
| ARHGEF4 | -1.37488 | 3.36E-07 | 7.23E-06 |
| GHRHR | -1.37476 | 0.000663 | 0.003436 |
| KLRB1 | -1.37436 | 4.93E-08 | 1.56E-06 |
| HMSD | -1.37361 | 7.91E-06 | 9.17E-05 |
| BCRP2 | -1.37237 | 0.004751 | 0.016993 |
| COL6A6 | -1.37235 | 2.97E-06 | 4.19E-05 |
| MIR4257 | -1.37217 | 2.05E-05 | 0.000199 |
| LSAMP-AS3 | -1.36966 | 0.00906 | 0.028663 |
| SYN3 | -1.36881 | 2.16E-07 | 5.05E-06 |
| FREM2 | -1.36811 | 1.66E-09 | 9.71E-08 |
| C20orf202 | -1.36734 | 1.35E-06 | 2.24E-05 |
| TSPAN13 | -1.36712 | 9.17E-11 | 9.69E-09 |
| FAM70A | -1.36655 | 1.91E-05 | 0.000188 |
| C1orf198 | -1.3656 | 1.35E-11 | 2.29E-09 |
| LOC654433 | -1.3654 | 0.00266 | 0.010559 |
| PRAP1 | -1.36462 | 6.3E-08 | 1.87E-06 |
| PTN | -1.36421 | 1.55E-09 | 9.11E-08 |
| BOLL | -1.3625 | 0.000441 | 0.00245 |
| LOC257358 | -1.36198 | 5.83E-07 | 1.13E-05 |
| LOC388387 | -1.36083 | 1.2E-06 | 2.03E-05 |
| LOC100506585 | -1.36001 | 7.33E-08 | 2.12E-06 |
| LOC728537 | -1.35911 | 8.99E-06 | 0.000101 |
| CTSW | -1.35796 | 3.08E-05 | 0.000276 |
| HAS3 | -1.35783 | 1.15E-06 | 1.96E-05 |
| AMICA1 | -1.35709 | 1.55E-07 | 3.89E-06 |
| PRAM1 | -1.35532 | 3.16E-07 | 6.89E-06 |
| CABP4 | -1.35515 | 3.25E-09 | 1.65E-07 |
| MST1P2 | -1.35393 | 7.93E-08 | 2.23E-06 |
| SEMA3B | -1.352 | 2.35E-09 | 1.29E-07 |
| SOAT2 | -1.35111 | 0.000227 | 0.001424 |
| NAT8B | -1.3497 | 0.000138 | 0.000941 |
| KRTAP5-10 | -1.34908 | 0.000444 | 0.002462 |
| VEPH1 | -1.34816 | 1.12E-06 | 1.92E-05 |
| HSD3BP4 | -1.34643 | 9.57E-07 | 1.7E-05 |
| GBA3 | -1.3453 | 0.000453 | 0.002502 |
| L1TD1 | -1.34476 | 2.34E-06 | 3.46E-05 |
| LOC100507055 | -1.3441 | 1.76E-06 | 2.75E-05 |
| CYP4Z2P | -1.34402 | 2.97E-07 | 6.56E-06 |
| SLC26A7 | -1.34223 | 9.69E-05 | 0.000704 |
| SPRYD7 | -1.34042 | 2.81E-11 | 3.79E-09 |
| CNKSR2 | -1.34028 | 1.64E-07 | 4.07E-06 |
| COL4A4 | -1.34008 | 3.08E-08 | 1.05E-06 |
| LOC644100 | -1.33937 | 0.000452 | 0.002498 |
| KCNK7 | -1.33765 | 7.15E-05 | 0.00055 |
| ERAS | -1.33718 | 0.001026 | 0.004861 |
| S100A4 | -1.33425 | 8.03E-11 | 8.7E-09 |
| LAMC3 | -1.33393 | 8.94E-05 | 0.00066 |
| RIMS4 | -1.33236 | 3.01E-06 | 4.23E-05 |
| LOC100129722 | -1.3319 | 9.9E-08 | 2.67E-06 |
| PIGA | -1.33143 | 6.62E-07 | 1.26E-05 |
| LOC100506433 | -1.33135 | 0.001067 | 0.005023 |
| ACSBG2 | -1.33122 | 3.27E-08 | 1.11E-06 |
| FMO5 | -1.33042 | 1.08E-07 | 2.87E-06 |
| IDO2 | -1.32912 | 0.00057 | 0.003027 |
| WIF1 | -1.32887 | 3.41E-07 | 7.33E-06 |
| SLC22A6 | -1.32672 | 0.001637 | 0.007101 |
| MTRNR2L4 | -1.32655 | 0.00017 | 0.001117 |
| CALN1 | -1.32636 | 0.000159 | 0.001056 |
| RGL3 | -1.32633 | 1.96E-08 | 7.28E-07 |
| KIR3DL2 | -1.3262 | 0.003184 | 0.012224 |
| SFTPA2 | -1.32618 | 0.000169 | 0.00111 |
| LOC285359 | -1.32611 | 8.08E-05 | 0.00061 |
| CEACAM22P | -1.3257 | 0.000239 | 0.001486 |
| ZFP36 | -1.32528 | 9.45E-05 | 0.00069 |
| CCDC84 | -1.32505 | 4.38E-06 | 5.76E-05 |
| SFTPA1 | -1.32478 | 0.000107 | 0.000765 |
| CDKL2 | -1.32453 | 2.21E-09 | 1.23E-07 |
| ASMTL-AS1 | -1.32399 | 9.34E-05 | 0.000684 |
| ASMTL-AS1 | -1.32399 | 9.34E-05 | 0.000684 |
| MIR3682 | -1.32391 | 0.001531 | 0.006731 |
| SLC16A11 | -1.32279 | 1.75E-09 | 1.01E-07 |
| SLITRK5 | -1.32107 | 0.001956 | 0.008224 |
| KRT4 | -1.31986 | 0.000247 | 0.001528 |
| IFI44L | -1.31907 | 1.99E-05 | 0.000194 |
| GHRL | -1.31848 | 2.67E-05 | 0.000246 |
| ETV5 | -1.31835 | 2.15E-09 | 1.2E-07 |
| CYP3A7-CYP3AP1 | -1.31824 | 0.000705 | 0.003601 |
| GDPD5 | -1.31569 | 2.05E-09 | 1.16E-07 |
| CDH19 | -1.31497 | 2.03E-05 | 0.000197 |
| MIR3671 | -1.31384 | 4.32E-09 | 2.09E-07 |
| HBA1 | -1.31299 | 0.016238 | 0.045716 |
| FOXF1 | -1.31268 | 2.82E-06 | 4.01E-05 |
| CRX | -1.30708 | 0.000507 | 0.002762 |
| LOC100101266 | -1.30665 | 4.49E-05 | 0.000377 |
| FLJ42393 | -1.30661 | 7.6E-05 | 0.000579 |
| RAB17 | -1.30646 | 1.4E-11 | 2.34E-09 |
| SLC4A10 | -1.30561 | 0.000291 | 0.001752 |
| OCLN | -1.3056 | 2.58E-11 | 3.55E-09 |
| LINC00482 | -1.30544 | 5.7E-10 | 4.13E-08 |
| TRPM1 | -1.30385 | 0.004605 | 0.016561 |
| LCAT | -1.30299 | 1.77E-07 | 4.32E-06 |
| GPBAR1 | -1.30174 | 1.99E-08 | 7.35E-07 |
| NR4A2 | -1.30161 | 0.003637 | 0.013672 |
| JUND | -1.29983 | 7.21E-06 | 8.49E-05 |
| C20orf203 | -1.29922 | 0.000368 | 0.002118 |
| TNFSF10 | -1.29863 | 5.86E-08 | 1.77E-06 |
| LOC100268168 | -1.29797 | 3.19E-09 | 1.62E-07 |
| TCEAL2 | -1.29767 | 6.24E-07 | 1.2E-05 |
| UNC13D | -1.29691 | 5.42E-10 | 3.99E-08 |
| EPOR | -1.29591 | 1.15E-09 | 7.26E-08 |
| CYP4F12 | -1.2959 | 2.61E-06 | 3.77E-05 |
| ITGA2B | -1.29555 | 0.002719 | 0.010737 |
| SLC25A41 | -1.29443 | 0.006454 | 0.021746 |
| STAC | -1.29359 | 6.86E-06 | 8.17E-05 |
| PLCZ1 | -1.29113 | 0.000144 | 0.000977 |
| CAPN3 | -1.29058 | 1.73E-09 | 1E-07 |
| ALPP | -1.29056 | 3.75E-05 | 0.000325 |
| TEK | -1.28857 | 8.81E-08 | 2.43E-06 |
| ARGLU1 | -1.2875 | 1.95E-10 | 1.74E-08 |
| FRY | -1.28729 | 2.78E-10 | 2.28E-08 |
| RBM44 | -1.28706 | 3.35E-07 | 7.22E-06 |
| DKFZP434A062 | -1.28673 | 0.000152 | 0.001021 |
| LINC00261 | -1.28661 | 1.4E-08 | 5.41E-07 |
| KCTD16 | -1.28659 | 4.06E-05 | 0.000346 |
| ZNF300P1 | -1.28599 | 0.000254 | 0.001565 |
| TULP2 | -1.28571 | 5.74E-05 | 0.000459 |
| LOC100505933 | -1.28558 | 2.56E-05 | 0.000239 |
| LOC400794 | -1.28542 | 0.000486 | 0.002662 |
| CDO1 | -1.28539 | 1.36E-07 | 3.5E-06 |
| ZNF331 | -1.28361 | 9.06E-07 | 1.63E-05 |
| ESRG | -1.28339 | 5.41E-05 | 0.000437 |
| ITPK1-AS1 | -1.28302 | 7.96E-06 | 9.2E-05 |
| PON1 | -1.28281 | 4.67E-05 | 0.00039 |
| HLF | -1.28272 | 7.37E-07 | 1.38E-05 |
| LRRC2 | -1.28246 | 2.09E-05 | 0.000201 |
| TNNC2 | -1.28225 | 4.33E-06 | 5.71E-05 |
| ICAM4 | -1.28214 | 0.001098 | 0.005151 |
| B3GALT2 | -1.28176 | 5.09E-06 | 6.48E-05 |
| RTN1 | -1.28069 | 1.48E-08 | 5.7E-07 |
| ABCA9 | -1.28045 | 2.2E-07 | 5.13E-06 |
| FLJ13197 | -1.28032 | 9.42E-07 | 1.68E-05 |
| DUSP6 | -1.2802 | 9.1E-07 | 1.64E-05 |
| RPS16P5 | -1.27935 | 0.000112 | 0.000796 |
| ARHGEF26-AS1 | -1.27919 | 7.08E-06 | 8.37E-05 |
| MASP2 | -1.27823 | 2.95E-08 | 1.02E-06 |
| FASLG | -1.27769 | 2.34E-05 | 0.000221 |
| FCGR1B | -1.27757 | 9.31E-06 | 0.000104 |
| PRLR | -1.27732 | 1.87E-05 | 0.000185 |
| OR5E1P | -1.27705 | 0.002108 | 0.008741 |
| IFIT1 | -1.27622 | 2.8E-06 | 3.99E-05 |
| LOC283392 | -1.27619 | 1.26E-06 | 2.11E-05 |
| LOC100507589 | -1.27618 | 9.19E-09 | 3.86E-07 |
| C8orf46 | -1.27577 | 9.33E-06 | 0.000104 |
| GNG11 | -1.27474 | 2.74E-08 | 9.59E-07 |
| RADIL | -1.27428 | 6.4E-09 | 2.88E-07 |
| NOS1 | -1.27345 | 3.81E-05 | 0.000329 |
| MAP2 | -1.27317 | 7.24E-08 | 2.1E-06 |
| FAT3 | -1.27263 | 5.12E-06 | 6.51E-05 |
| PRSS48 | -1.2707 | 6.75E-05 | 0.000522 |
| KLRK1 | -1.26889 | 0.000416 | 0.002338 |
| EDN1 | -1.26762 | 5.97E-05 | 0.000473 |
| ZSCAN10 | -1.26686 | 0.000129 | 0.000891 |
| RND1 | -1.26653 | 0.016922 | 0.047157 |
| LOC728084 | -1.26416 | 0.00023 | 0.001441 |
| C9orf11 | -1.26379 | 7.73E-05 | 0.000586 |
| ZNF365 | -1.26329 | 7.2E-08 | 2.09E-06 |
| CD36 | -1.26323 | 1.77E-06 | 2.76E-05 |
| DDX25 | -1.26288 | 0.00053 | 0.002863 |
| CRYGS | -1.26284 | 2.07E-05 | 0.0002 |
| TXNDC3 | -1.2623 | 2.48E-05 | 0.000232 |
| DACT2 | -1.26087 | 0.000508 | 0.002769 |
| LOC100128071 | -1.26026 | 2.39E-06 | 3.52E-05 |
| FCGR3A | -1.25979 | 3.57E-05 | 0.000312 |
| LOC728643 | -1.25904 | 2.3E-06 | 3.41E-05 |
| LOC284379 | -1.25871 | 0.001445 | 0.006426 |
| MLXIPL | -1.25836 | 7.01E-06 | 8.31E-05 |
| BNIPL | -1.25792 | 1.48E-05 | 0.000153 |
| GGT3P | -1.25771 | 0.000985 | 0.004719 |
| C15orf63 | -1.25753 | 0.001171 | 0.00542 |
| CDKN2B | -1.25749 | 2.83E-07 | 6.35E-06 |
| COX4I2 | -1.25723 | 1.1E-07 | 2.9E-06 |
| LOC646576 | -1.2571 | 1.37E-06 | 2.26E-05 |
| ARHGAP29 | -1.25708 | 8.51E-10 | 5.7E-08 |
| SFTA3 | -1.25531 | 1.21E-08 | 4.9E-07 |
| C1orf115 | -1.25506 | 2.26E-10 | 1.96E-08 |
| LOC100288911 | -1.25459 | 1.69E-07 | 4.17E-06 |
| LOC285889 | -1.25442 | 0.000196 | 0.001257 |
| CCL4 | -1.25415 | 0.000111 | 0.000786 |
| SLFNL1 | -1.25408 | 7.45E-06 | 8.73E-05 |
| MAPK4 | -1.25381 | 5.01E-06 | 6.4E-05 |
| GBP7 | -1.25364 | 0.000304 | 0.001813 |
| PLAC8L1 | -1.25295 | 9.05E-05 | 0.000667 |
| CRIP3 | -1.25263 | 0.000402 | 0.002278 |
| CACNA2D3 | -1.25223 | 1.23E-06 | 2.06E-05 |
| MSLNL | -1.25196 | 0.007074 | 0.023404 |
| LAMP3 | -1.25186 | 3.85E-05 | 0.000332 |
| GDF10 | -1.25178 | 9.84E-06 | 0.000109 |
| RLBP1 | -1.2517 | 0.000704 | 0.003597 |
| CYP4F22 | -1.25162 | 0.000882 | 0.004308 |
| OR2A7 | -1.25111 | 0.000159 | 0.001056 |
| LINC00299 | -1.25018 | 9.89E-05 | 0.000716 |
| CLEC12A | -1.24977 | 1.78E-05 | 0.000178 |
| FMO2 | -1.24938 | 3.74E-06 | 5.07E-05 |
| NINJ2 | -1.24907 | 1.25E-09 | 7.69E-08 |
| CADM1 | -1.24905 | 4.33E-09 | 2.09E-07 |
| METTL21C | -1.24834 | 0.000413 | 0.002326 |
| HYMAI | -1.24762 | 0.000146 | 0.000985 |
| ADHFE1 | -1.24704 | 1.16E-08 | 4.71E-07 |
| CAPN9 | -1.24528 | 0.000428 | 0.002389 |
| GRIA4 | -1.24509 | 0.001283 | 0.005836 |
| CYP3A43 | -1.24396 | 0.000219 | 0.001383 |
| PAR1 | -1.2437 | 0.000371 | 0.00213 |
| DPEP3 | -1.24364 | 1.44E-05 | 0.000149 |
| FLT4 | -1.24261 | 1.71E-10 | 1.56E-08 |
| ANXA8L2 | -1.24199 | 8.85E-06 | 0.0001 |
| CCDC38 | -1.24123 | 1.92E-05 | 0.000189 |
| LOC100507117 | -1.24078 | 0.000242 | 0.001504 |
| TSIX | -1.24075 | 0.001424 | 0.006353 |
| MYH2 | -1.23948 | 0.012427 | 0.036853 |
| FRMD3 | -1.23895 | 2.38E-08 | 8.55E-07 |
| HERC2P4 | -1.23779 | 9.76E-07 | 1.72E-05 |
| TMEM72-AS1 | -1.23716 | 9.19E-06 | 0.000103 |
| ZDHHC8P1 | -1.23701 | 1.75E-05 | 0.000176 |
| RNF133 | -1.237 | 8.34E-05 | 0.000625 |
| PTGS2 | -1.23576 | 0.000948 | 0.004575 |
| SPN | -1.23564 | 1.45E-06 | 2.37E-05 |
| TEX29 | -1.23525 | 0.002464 | 0.009938 |
| MSR1 | -1.23487 | 8.72E-05 | 0.000646 |
| NFKBIA | -1.23421 | 3.67E-06 | 5.01E-05 |
| PRSS41 | -1.23143 | 0.002754 | 0.010833 |
| LOC643441 | -1.23105 | 5.54E-06 | 6.92E-05 |
| CDH5 | -1.23075 | 1.25E-07 | 3.27E-06 |
| DNM3 | -1.23014 | 2.58E-11 | 3.55E-09 |
| PAPSS2 | -1.2294 | 4.1E-08 | 1.34E-06 |
| PDZD9 | -1.22923 | 6.58E-06 | 7.9E-05 |
| MIR27A | -1.22828 | 0.005816 | 0.020032 |
| ITGAL | -1.22807 | 2.32E-08 | 8.39E-07 |
| FAM176A | -1.22762 | 1.58E-08 | 5.98E-07 |
| NPR3 | -1.22721 | 0.000126 | 0.000873 |
| RPGRIP1 | -1.22642 | 6.45E-09 | 2.89E-07 |
| PCOLCE2 | -1.22634 | 0.000604 | 0.00318 |
| SEZ6L | -1.22433 | 0.000695 | 0.003562 |
| KCNN2 | -1.22407 | 0.002514 | 0.010105 |
| AMELX | -1.22406 | 0.000911 | 0.004428 |
| SEMA3G | -1.22393 | 0.000885 | 0.004321 |
| MTMR9LP | -1.22272 | 5.53E-07 | 1.09E-05 |
| CABLES1 | -1.22271 | 5.6E-10 | 4.07E-08 |
| CLEC14A | -1.22126 | 8.45E-08 | 2.33E-06 |
| SOX7 | -1.22001 | 0.000139 | 0.000947 |
| CASKIN2 | -1.21995 | 5.04E-07 | 1.02E-05 |
| KIR3DX1 | -1.21927 | 0.001041 | 0.004918 |
| LEFTY2 | -1.21806 | 0.000198 | 0.001264 |
| FBXO39 | -1.21643 | 0.000344 | 0.002009 |
| CDHR5 | -1.2164 | 4.85E-05 | 0.000401 |
| SNORA75 | -1.21618 | 0.000275 | 0.001672 |
| HEATR8 | -1.21591 | 0.000519 | 0.002816 |
| EPAS1 | -1.21504 | 3.42E-07 | 7.34E-06 |
| P2RY13 | -1.21478 | 4.68E-06 | 6.05E-05 |
| FAM166A | -1.21406 | 0.000316 | 0.001875 |
| LOC100128573 | -1.21397 | 0.000121 | 0.000847 |
| LINC00092 | -1.21387 | 1.81E-07 | 4.39E-06 |
| SYT13 | -1.21382 | 0.005756 | 0.019863 |
| NEBL | -1.21315 | 1.69E-11 | 2.62E-09 |
| NEK3 | -1.2111 | 5.64E-07 | 1.1E-05 |
| DAPK1 | -1.21028 | 4.44E-12 | 1.12E-09 |
| DUSP8 | -1.21018 | 5.16E-06 | 6.54E-05 |
| IRX2 | -1.20943 | 3.51E-08 | 1.19E-06 |
| MIR320E | -1.20832 | 8.19E-05 | 0.000616 |
| CRYBB3 | -1.20807 | 0.002759 | 0.010849 |
| LGALSL | -1.20579 | 8.43E-08 | 2.33E-06 |
| PRB1 | -1.2055 | 0.002189 | 0.009022 |
| MIR210HG | -1.20521 | 2.45E-05 | 0.00023 |
| RGPD1 | -1.20518 | 0.006059 | 0.020705 |
| CHKB-CPT1B | -1.20476 | 6.59E-05 | 0.000513 |
| TXK | -1.20413 | 3.54E-08 | 1.19E-06 |
| AKR1CL1 | -1.20373 | 0.000904 | 0.004399 |
| ADCY4 | -1.2037 | 7.26E-09 | 3.18E-07 |
| LOC100128750 | -1.20317 | 4.91E-07 | 1E-05 |
| TMIGD2 | -1.20311 | 3.23E-05 | 0.000287 |
| PPP2R3B-AS1 | -1.20303 | 0.004025 | 0.01484 |
| IL27 | -1.203 | 0.00458 | 0.016498 |
| A4GNT | -1.20251 | 0.000179 | 0.001161 |
| ZNF404 | -1.20153 | 7.42E-06 | 8.7E-05 |
| LMAN1L | -1.20103 | 0.000652 | 0.003386 |
| NFKBIZ | -1.20066 | 2.33E-05 | 0.00022 |
| DIO3OS | -1.20065 | 9.56E-05 | 0.000697 |
| LOC100506474 | -1.19938 | 0.000151 | 0.001015 |
| DOCK4 | -1.19925 | 1.02E-11 | 1.9E-09 |
| RSPO2 | -1.19854 | 1.54E-05 | 0.000158 |
| LINC00260 | -1.19844 | 6.01E-06 | 7.36E-05 |
| NAALAD2 | -1.19816 | 1.37E-08 | 5.36E-07 |
| IFI27 | -1.19814 | 2.94E-05 | 0.000266 |
| ADTRP | -1.19784 | 7.06E-05 | 0.000544 |
| ESYT3 | -1.19742 | 3.15E-06 | 4.4E-05 |
| RASIP1 | -1.19727 | 6.91E-08 | 2.03E-06 |
| MMP28 | -1.19708 | 5.51E-09 | 2.56E-07 |
| KIAA1462 | -1.19701 | 2.56E-06 | 3.72E-05 |
| ILDR2 | -1.19635 | 1.08E-06 | 1.87E-05 |
| NRGN | -1.19633 | 1.52E-05 | 0.000156 |
| INHA | -1.19601 | 0.001974 | 0.008279 |
| NEDD4L | -1.19595 | 2.42E-11 | 3.44E-09 |
| C5orf56 | -1.19553 | 1.21E-10 | 1.21E-08 |
| WDR72 | -1.19358 | 0.000603 | 0.003175 |
| ID1 | -1.1934 | 7.92E-06 | 9.17E-05 |
| HSD17B7P2 | -1.1932 | 4.03E-06 | 5.39E-05 |
| DMRT2 | -1.19316 | 0.000567 | 0.003012 |
| TRIM71 | -1.193 | 0.00203 | 0.008461 |
| LINC00114 | -1.1911 | 0.001767 | 0.007568 |
| MIRLET7A1 | -1.19078 | 0.000464 | 0.002547 |
| UGT2B4 | -1.19004 | 0.009561 | 0.029957 |
| MLLT4-AS1 | -1.18773 | 0.000154 | 0.001031 |
| LOC100128568 | -1.1872 | 0.001793 | 0.00765 |
| LINC00514 | -1.18709 | 5.15E-05 | 0.00042 |
| RAPSN | -1.18699 | 0.002475 | 0.009973 |
| STX16-NPEPL1 | -1.18663 | 6.98E-08 | 2.04E-06 |
| IRS4 | -1.18567 | 0.003783 | 0.014118 |
| NKX2-8 | -1.18556 | 0.000193 | 0.001237 |
| KCNS1 | -1.18502 | 0.000527 | 0.002854 |
| CYP2E1 | -1.18404 | 1.58E-05 | 0.000162 |
| PRSS45 | -1.18395 | 0.00218 | 0.008998 |
| HCRTR1 | -1.18361 | 0.002356 | 0.009589 |
| SMA4 | -1.1826 | 9.24E-10 | 6.11E-08 |
| LOC100131434 | -1.18217 | 3.93E-06 | 5.28E-05 |
| MIR573 | -1.18194 | 0.000368 | 0.00212 |
| ESAM | -1.18072 | 1.96E-08 | 7.28E-07 |
| LOC100129148 | -1.18031 | 0.000153 | 0.001023 |
| FRAS1 | -1.17985 | 1.18E-06 | 2E-05 |
| TGM4 | -1.17981 | 0.005476 | 0.019058 |
| FZD5 | -1.17976 | 4.49E-08 | 1.43E-06 |
| ARAP2 | -1.17966 | 7.29E-12 | 1.52E-09 |
| ALDH8A1 | -1.17884 | 0.000112 | 0.000793 |
| SLC46A2 | -1.1787 | 0.000117 | 0.000821 |
| MARCO | -1.17812 | 0.000918 | 0.004458 |
| KANSL1-AS1 | -1.17807 | 3.59E-05 | 0.000314 |
| CALCRL | -1.17792 | 1.59E-05 | 0.000163 |
| AMY2B | -1.17785 | 1.25E-06 | 2.1E-05 |
| PSORS1C2 | -1.17776 | 0.012442 | 0.036887 |
| LOC100505678 | -1.17767 | 2.13E-05 | 0.000205 |
| ZNF704 | -1.17654 | 5.88E-10 | 4.24E-08 |
| LOC644714 | -1.17599 | 1.63E-06 | 2.6E-05 |
| LOC100302401 | -1.1756 | 2.26E-07 | 5.23E-06 |
| TCF15 | -1.17533 | 0.000382 | 0.00219 |
| CD52 | -1.17475 | 9.91E-06 | 0.000109 |
| TBC1D26 | -1.17429 | 0.000144 | 0.000974 |
| UNC13B | -1.17414 | 1.33E-13 | 7.69E-11 |
| SIGLEC11 | -1.17311 | 1.45E-05 | 0.00015 |
| MIR135A2 | -1.1726 | 0.014131 | 0.040807 |
| CHRNA10 | -1.17227 | 3.66E-05 | 0.000319 |
| OR52N4 | -1.17205 | 8.27E-05 | 0.00062 |
| MAS1L | -1.17187 | 0.010812 | 0.03299 |
| AKNAD1 | -1.17146 | 9.38E-05 | 0.000687 |
| SIRPD | -1.17141 | 0.0001 | 0.000724 |
| MIR605 | -1.17117 | 0.015651 | 0.044393 |
| APOL3 | -1.17102 | 3.15E-09 | 1.61E-07 |
| TM4SF4 | -1.16926 | 0.000508 | 0.002769 |
| SLC10A1 | -1.16874 | 0.001057 | 0.004982 |
| LRRN4 | -1.16854 | 0.00034 | 0.00199 |
| LOC100507203 | -1.16831 | 0.003492 | 0.013212 |
| CGN | -1.16777 | 1.96E-09 | 1.12E-07 |
| RASL10A | -1.16772 | 0.001159 | 0.005382 |
| CCL5 | -1.16728 | 2.29E-06 | 3.41E-05 |
| MIRLET7BHG | -1.16665 | 0.00011 | 0.000781 |
| FAM125B | -1.16516 | 8.05E-10 | 5.45E-08 |
| DLL4 | -1.16516 | 1.09E-05 | 0.000118 |
| ANKRD20A4 | -1.16439 | 0.001994 | 0.008337 |
| BMP3 | -1.16437 | 5.79E-06 | 7.17E-05 |
| ADAM1 | -1.16368 | 2.71E-09 | 1.44E-07 |
| LPA | -1.16306 | 0.006144 | 0.020921 |
| LOC285965 | -1.16301 | 2.29E-06 | 3.4E-05 |
| TREH | -1.16251 | 0.00088 | 0.004304 |
| RBP4 | -1.16132 | 0.002783 | 0.010926 |
| LOC100288974 | -1.16095 | 2.04E-06 | 3.08E-05 |
| GKN2 | -1.16051 | 0.000658 | 0.003416 |
| RHOXF1 | -1.16034 | 0.012483 | 0.036995 |
| GPR152 | -1.15935 | 0.000266 | 0.001626 |
| CD300LF | -1.15902 | 9.25E-06 | 0.000104 |
| GIMAP8 | -1.15855 | 1.62E-07 | 4.05E-06 |
| C5orf64 | -1.15849 | 0.002408 | 0.009765 |
| CTRL | -1.15682 | 1.4E-06 | 2.31E-05 |
| LGR5 | -1.15582 | 0.000704 | 0.003597 |
| PDE4C | -1.15503 | 8.63E-05 | 0.000641 |
| N4BP2L1 | -1.15449 | 6.26E-12 | 1.43E-09 |
| MANSC4 | -1.15373 | 0.000878 | 0.004297 |
| IL20RA | -1.15369 | 0.000179 | 0.001165 |
| CGNL1 | -1.15366 | 5.73E-09 | 2.63E-07 |
| UACA | -1.15359 | 2.66E-09 | 1.43E-07 |
| ASMT | -1.15353 | 0.001042 | 0.004921 |
| ASMT | -1.15353 | 0.001042 | 0.004921 |
| LOC100506795 | -1.15346 | 0.000305 | 0.001817 |
| NRBP2 | -1.15334 | 2.45E-11 | 3.45E-09 |
| LGALS2 | -1.1533 | 4.74E-05 | 0.000394 |
| PPP1R15A | -1.15325 | 4.68E-06 | 6.05E-05 |
| MAP4K2 | -1.15232 | 6.95E-12 | 1.48E-09 |
| KCNH6 | -1.15227 | 0.003041 | 0.011761 |
| LOC442132 | -1.15146 | 0.008093 | 0.026142 |
| GPR112 | -1.15072 | 0.015711 | 0.04451 |
| C6orf223 | -1.15047 | 0.0009 | 0.004381 |
| ARHGEF10 | -1.15047 | 7.35E-09 | 3.2E-07 |
| LOC100505658 | -1.15013 | 0.000883 | 0.004314 |
| REP15 | -1.14947 | 3.74E-08 | 1.24E-06 |
| LOC100506472 | -1.14901 | 2.82E-09 | 1.5E-07 |
| CCDC116 | -1.14859 | 6.37E-05 | 0.000499 |
| HPX | -1.1475 | 2.51E-05 | 0.000234 |
| CHRM5 | -1.14709 | 1.65E-05 | 0.000167 |
| HOXA4 | -1.14559 | 8.03E-08 | 2.25E-06 |
| ST7-AS1 | -1.14511 | 5.19E-07 | 1.04E-05 |
| TMEM14E | -1.14451 | 0.000606 | 0.003187 |
| ATOH8 | -1.14387 | 1.06E-05 | 0.000115 |
| PAPOLB | -1.14387 | 0.000616 | 0.003233 |
| PRRT4 | -1.14361 | 0.000637 | 0.003322 |
| DGKE | -1.14347 | 3.55E-11 | 4.65E-09 |
| ACVRL1 | -1.14331 | 8.03E-06 | 9.25E-05 |
| JAKMIP2 | -1.14295 | 5.86E-05 | 0.000466 |
| CYP7A1 | -1.14248 | 0.000749 | 0.003777 |
| LRIT3 | -1.14235 | 5.55E-05 | 0.000447 |
| C16orf78 | -1.14226 | 9.46E-05 | 0.000691 |
| SNORA28 | -1.14114 | 0.000113 | 0.000798 |
| LOC143666 | -1.14064 | 5.92E-08 | 1.78E-06 |
| BOK-AS1 | -1.14044 | 0.00044 | 0.002448 |
| CELF4 | -1.13958 | 4.94E-07 | 1E-05 |
| SLC25A2 | -1.13951 | 0.00025 | 0.001542 |
| GJC2 | -1.13836 | 4.5E-07 | 9.29E-06 |
| LY6G5B | -1.13821 | 6.57E-07 | 1.25E-05 |
| ANKRD44 | -1.13809 | 5.21E-10 | 3.88E-08 |
| SLC16A12 | -1.13762 | 0.000237 | 0.001477 |
| SAP25 | -1.13714 | 8.31E-05 | 0.000623 |
| ROBO3 | -1.13707 | 3.01E-08 | 1.03E-06 |
| IDI2-AS1 | -1.13677 | 2.3E-06 | 3.41E-05 |
| GYS2 | -1.13676 | 8.94E-05 | 0.00066 |
| LOC727896 | -1.13586 | 4.75E-06 | 6.12E-05 |
| CHRM4 | -1.13573 | 0.013408 | 0.039129 |
| IGSF23 | -1.13524 | 0.002727 | 0.01076 |
| LINC00028 | -1.13445 | 5.68E-05 | 0.000455 |
| ASB9 | -1.13437 | 1.33E-08 | 5.23E-07 |
| LOC100134015 | -1.13285 | 4.82E-05 | 0.000399 |
| IQSEC3 | -1.13247 | 8.48E-05 | 0.000632 |
| UBAP1L | -1.13231 | 1.72E-06 | 2.7E-05 |
| WFDC13 | -1.1314 | 0.000193 | 0.001236 |
| ZNF682 | -1.13127 | 2.81E-06 | 4.01E-05 |
| LOC441455 | -1.13063 | 7.69E-05 | 0.000584 |
| LOC100507424 | -1.12944 | 7.03E-06 | 8.32E-05 |
| THSD1 | -1.12916 | 8.96E-07 | 1.61E-05 |
| HCG4B | -1.1287 | 0.001549 | 0.0068 |
| MUC6 | -1.12709 | 0.008718 | 0.027775 |
| SLC2A12 | -1.12681 | 4.25E-07 | 8.86E-06 |
| LEAP2 | -1.12419 | 9.5E-06 | 0.000106 |
| SYNDIG1L | -1.12372 | 0.011321 | 0.034222 |
| P2RY1 | -1.12355 | 0.001118 | 0.005224 |
| RAB11FIP1 | -1.12281 | 9.39E-08 | 2.55E-06 |
| CLDN11 | -1.12272 | 2.85E-08 | 9.87E-07 |
| MYOZ1 | -1.12268 | 5.82E-06 | 7.19E-05 |
| SLC6A13 | -1.12084 | 2.33E-06 | 3.44E-05 |
| USP44 | -1.12061 | 9.16E-07 | 1.64E-05 |
| MYOC | -1.12042 | 0.012216 | 0.036343 |
| C9orf173 | -1.12034 | 0.000654 | 0.003395 |
| PAQR5 | -1.12 | 2.48E-09 | 1.34E-07 |
| ZEB2-AS1 | -1.1192 | 8.27E-06 | 9.46E-05 |
| DOCK6 | -1.11892 | 3.48E-10 | 2.73E-08 |
| HNF1B | -1.11786 | 1.82E-10 | 1.65E-08 |
| RNF138P1 | -1.11786 | 1.24E-05 | 0.000131 |
| LYZ | -1.11738 | 0.00078 | 0.003902 |
| FOXA2 | -1.11694 | 1.85E-07 | 4.48E-06 |
| FAM19A1 | -1.11693 | 4.75E-07 | 9.71E-06 |
| RGN | -1.11684 | 1.14E-08 | 4.67E-07 |
| PCDH15 | -1.11631 | 1.38E-05 | 0.000144 |
| NKX2-1 | -1.11587 | 4.34E-07 | 8.99E-06 |
| KLRC4-KLRK1 | -1.11563 | 0.001427 | 0.006363 |
| DOK7 | -1.11518 | 3.17E-05 | 0.000283 |
| PGPEP1L | -1.1146 | 0.00145 | 0.006443 |
| UPK1A | -1.11429 | 0.003539 | 0.013374 |
| CD247 | -1.11357 | 2.65E-08 | 9.31E-07 |
| LYPD6 | -1.11329 | 0.003617 | 0.013614 |
| CCIN | -1.11305 | 0.000528 | 0.002855 |
| LOC100131733 | -1.11303 | 1.54E-06 | 2.48E-05 |
| ITM2A | -1.11218 | 6.86E-07 | 1.29E-05 |
| SLC25A27 | -1.11165 | 7.56E-06 | 8.83E-05 |
| PPARG | -1.11078 | 4.19E-05 | 0.000356 |
| CD55 | -1.11006 | 3.64E-07 | 7.72E-06 |
| LOC439949 | -1.10956 | 3.67E-09 | 1.84E-07 |
| GPSM1 | -1.10834 | 2.9E-07 | 6.42E-06 |
| FLJ45513 | -1.10709 | 0.000122 | 0.00085 |
| CDH13 | -1.1064 | 7.63E-05 | 0.00058 |
| C8orf56 | -1.10633 | 0.001225 | 0.005622 |
| FAM46B | -1.10595 | 1.8E-05 | 0.000179 |
| MPL | -1.10586 | 8.55E-07 | 1.55E-05 |
| TBX3 | -1.10544 | 6.25E-07 | 1.2E-05 |
| CLEC1A | -1.10374 | 1.89E-07 | 4.53E-06 |
| GRM3 | -1.10362 | 0.000444 | 0.002464 |
| CRIPAK | -1.10307 | 1.98E-07 | 4.71E-06 |
| LOC100272228 | -1.10276 | 2.02E-06 | 3.07E-05 |
| HPN | -1.10268 | 0.001138 | 0.005298 |
| PCDH9 | -1.10127 | 0.003435 | 0.013031 |
| PI4KAP2 | -1.10126 | 1.01E-05 | 0.000111 |
| PER1 | -1.10042 | 2.39E-05 | 0.000225 |
| LOC338817 | -1.09964 | 0.001161 | 0.005387 |
| C9orf153 | -1.09943 | 0.000546 | 0.00293 |
| MIR17HG | -1.09935 | 3.36E-05 | 0.000296 |
| FZD8 | -1.0985 | 1.54E-09 | 9.07E-08 |
| LOC730102 | -1.09821 | 6.93E-09 | 3.06E-07 |
| SLC19A1 | -1.09708 | 7.9E-08 | 2.23E-06 |
| SIGLEC16 | -1.09707 | 8.87E-05 | 0.000656 |
| JPH4 | -1.0969 | 1.87E-07 | 4.49E-06 |
| LIMCH1 | -1.09673 | 1.82E-08 | 6.82E-07 |
| LOC100128054 | -1.09651 | 0.000791 | 0.003946 |
| RRAS | -1.09631 | 7.17E-08 | 2.09E-06 |
| LOC100507577 | -1.09606 | 1.06E-06 | 1.84E-05 |
| CDKN1C | -1.0955 | 2.1E-08 | 7.71E-07 |
| CTRC | -1.09498 | 0.004938 | 0.017527 |
| GZMM | -1.0949 | 0.000151 | 0.001015 |
| SP6 | -1.09425 | 0.000245 | 0.00152 |
| HSD17B3 | -1.09371 | 0.000595 | 0.003133 |
| XCL2 | -1.09365 | 0.000171 | 0.001121 |
| EMID2 | -1.09362 | 0.00021 | 0.00133 |
| CD274 | -1.09322 | 0.000134 | 0.00092 |
| NUDT9P1 | -1.0921 | 1.84E-05 | 0.000182 |
| IL23R | -1.09209 | 0.001949 | 0.008197 |
| C3orf27 | -1.09125 | 0.002737 | 0.010783 |
| TMEM125 | -1.09037 | 1.22E-08 | 4.93E-07 |
| DDX60L | -1.09037 | 1.79E-07 | 4.35E-06 |
| ALS2CR11 | -1.09037 | 3.22E-05 | 0.000287 |
| ICOS | -1.09014 | 0.000108 | 0.000767 |
| IFI44 | -1.08994 | 1.54E-06 | 2.49E-05 |
| CASP5 | -1.08994 | 0.000761 | 0.003827 |
| LCNL1 | -1.0899 | 0.0031 | 0.011951 |
| LOC100506025 | -1.08932 | 0.000924 | 0.004481 |
| CXADR | -1.08892 | 4.03E-09 | 1.97E-07 |
| AMIGO2 | -1.08868 | 1.49E-11 | 2.41E-09 |
| TEF | -1.08838 | 0.00061 | 0.003205 |
| CHRM3 | -1.08835 | 2.76E-07 | 6.23E-06 |
| ITIH2 | -1.0876 | 0.00211 | 0.008744 |
| SERPINE3 | -1.08754 | 0.000199 | 0.00127 |
| CCDC141 | -1.08749 | 0.001268 | 0.005779 |
| IFIT3 | -1.08731 | 4.37E-05 | 0.000369 |
| ATP8A1 | -1.08599 | 1.07E-08 | 4.41E-07 |
| C14orf80 | -1.08596 | 3.2E-05 | 0.000285 |
| SCARF1 | -1.08553 | 9.75E-11 | 1.02E-08 |
| MARCH1 | -1.08547 | 1.26E-08 | 5.05E-07 |
| ZNF705A | -1.08482 | 0.005916 | 0.020309 |
| ID4 | -1.08442 | 1.68E-06 | 2.66E-05 |
| RANBP17 | -1.08438 | 5.3E-08 | 1.65E-06 |
| CHKB | -1.08354 | 3.19E-05 | 0.000285 |
| MIR647 | -1.08344 | 0.000138 | 0.000942 |
| SEC14L4 | -1.08258 | 6.43E-06 | 7.78E-05 |
| OSCAR | -1.08249 | 0.000137 | 0.000936 |
| ADRA1D | -1.08212 | 6.18E-05 | 0.000487 |
| AOC4 | -1.08209 | 5.76E-05 | 0.000459 |
| HCG26 | -1.08141 | 4.23E-08 | 1.37E-06 |
| LOC285593 | -1.08125 | 4.51E-05 | 0.000379 |
| TMC7 | -1.08094 | 1.35E-07 | 3.49E-06 |
| NR0B2 | -1.08008 | 0.007157 | 0.023649 |
| KLHL33 | -1.07926 | 0.005838 | 0.020078 |
| TMPRSS5 | -1.07912 | 8.3E-06 | 9.49E-05 |
| PKIA | -1.07891 | 9.83E-09 | 4.09E-07 |
| CYP17A1 | -1.07888 | 0.000131 | 0.000901 |
| GAB1 | -1.07877 | 1.38E-09 | 8.27E-08 |
| EFNB2 | -1.0782 | 1.45E-07 | 3.69E-06 |
| CCBE1 | -1.07759 | 8.34E-05 | 0.000625 |
| SLC5A2 | -1.07746 | 3.67E-05 | 0.00032 |
| OR2W3 | -1.07744 | 0.006446 | 0.021729 |
| BEX5 | -1.07729 | 2.28E-05 | 0.000217 |
| LOC100505495 | -1.077 | 1.92E-06 | 2.94E-05 |
| CTNNAL1 | -1.07672 | 1.96E-07 | 4.68E-06 |
| RSPH6A | -1.07668 | 0.000387 | 0.002211 |
| RNF144B | -1.07659 | 2.4E-08 | 8.62E-07 |
| LRP2BP | -1.07653 | 1.81E-06 | 2.81E-05 |
| COL11A2 | -1.07643 | 8.88E-08 | 2.44E-06 |
| TSPYL6 | -1.07509 | 0.004668 | 0.016733 |
| LGALS17A | -1.07493 | 0.016713 | 0.04674 |
| TAS2R50 | -1.07481 | 4.79E-05 | 0.000398 |
| ZNF662 | -1.07457 | 2.23E-08 | 8.13E-07 |
| ZNF323 | -1.07452 | 4.54E-06 | 5.95E-05 |
| NKTR | -1.07413 | 6.94E-08 | 2.04E-06 |
| SSPO | -1.07377 | 1.67E-05 | 0.000169 |
| GTF2IRD2 | -1.07363 | 1.36E-06 | 2.25E-05 |
| ABCC6P1 | -1.07294 | 0.000566 | 0.003011 |
| LOC286467 | -1.0726 | 4.06E-05 | 0.000346 |
| C7orf41 | -1.07183 | 6.72E-09 | 2.99E-07 |
| LINC00176 | -1.0713 | 1.04E-05 | 0.000114 |
| LINC00189 | -1.07081 | 0.002348 | 0.009562 |
| C22orf24 | -1.07068 | 0.002228 | 0.009156 |
| SLAIN1 | -1.07051 | 2.1E-09 | 1.18E-07 |
| CAPN12 | -1.07004 | 3.32E-06 | 4.58E-05 |
| CPLX3 | -1.06995 | 0.012361 | 0.036682 |
| NOTUM | -1.06993 | 9.96E-05 | 0.000721 |
| SHH | -1.06942 | 6.72E-05 | 0.000521 |
| SNORD37 | -1.0692 | 0.000321 | 0.001899 |
| HLA-F-AS1 | -1.0685 | 5.43E-10 | 3.99E-08 |
| SGIP1 | -1.0685 | 1.09E-06 | 1.88E-05 |
| HLA-E | -1.06727 | 9.24E-08 | 2.52E-06 |
| CYB5R2 | -1.06687 | 8.39E-08 | 2.33E-06 |
| RNF148 | -1.06607 | 0.005914 | 0.020309 |
| HCAR2 | -1.06592 | 2.94E-05 | 0.000266 |
| THBD | -1.06528 | 3.02E-05 | 0.000272 |
| ZNF37BP | -1.06519 | 1.31E-08 | 5.18E-07 |
| PPP1R9A | -1.0649 | 6.91E-11 | 7.84E-09 |
| IDO1 | -1.06402 | 0.01754 | 0.048613 |
| NHSL1 | -1.06376 | 2.4E-10 | 2.05E-08 |
| PER2 | -1.06363 | 1.97E-07 | 4.68E-06 |
| ZDHHC19 | -1.06357 | 0.000899 | 0.004381 |
| DGCR10 | -1.06354 | 0.002442 | 0.009865 |
| GIMAP1 | -1.06352 | 2.13E-09 | 1.19E-07 |
| PPP4R4 | -1.06349 | 2.3E-05 | 0.000218 |
| LOC338758 | -1.06277 | 1E-09 | 6.48E-08 |
| GLT25D2 | -1.06242 | 9.47E-06 | 0.000105 |
| SLC7A9 | -1.06235 | 0.007547 | 0.024664 |
| LMOD2 | -1.06191 | 0.002296 | 0.009398 |
| SNORA38 | -1.06172 | 0.001727 | 0.00743 |
| FLJ31306 | -1.06152 | 2.86E-07 | 6.37E-06 |
| PTH2R | -1.06116 | 0.001469 | 0.006511 |
| CD69 | -1.06088 | 6.53E-05 | 0.00051 |
| LOC100131564 | -1.06072 | 8.83E-08 | 2.43E-06 |
| SEMA5B | -1.06018 | 0.006493 | 0.021833 |
| ZNF577 | -1.05998 | 3.55E-08 | 1.19E-06 |
| SNORA29 | -1.05903 | 0.000679 | 0.003497 |
| GUCY1A2 | -1.0571 | 0.000296 | 0.001774 |
| SLC22A1 | -1.05703 | 0.001162 | 0.00539 |
| CST6 | -1.05683 | 0.000121 | 0.000849 |
| ZNF596 | -1.05669 | 1.79E-06 | 2.79E-05 |
| GABBR1 | -1.05634 | 1.82E-06 | 2.82E-05 |
| TSSK3 | -1.05553 | 1.54E-09 | 9.07E-08 |
| POU5F1 | -1.05545 | 0.000629 | 0.003291 |
| GUCA1B | -1.05494 | 8.27E-05 | 0.00062 |
| MMEL1 | -1.05403 | 5.89E-06 | 7.26E-05 |
| ALOX5AP | -1.05389 | 5.43E-05 | 0.000439 |
| NCR3 | -1.05309 | 0.001191 | 0.005487 |
| SAMD9L | -1.05221 | 1.13E-07 | 2.96E-06 |
| HFM1 | -1.05007 | 1.7E-05 | 0.000171 |
| AFAP1L1 | -1.04998 | 1.78E-07 | 4.34E-06 |
| HBE1 | -1.04986 | 0.00523 | 0.018358 |
| LOC100507062 | -1.04964 | 2.19E-07 | 5.11E-06 |
| PAX6 | -1.04942 | 8.35E-08 | 2.32E-06 |
| SLC26A9 | -1.0493 | 0.001347 | 0.006067 |
| TAS2R10 | -1.04925 | 0.002773 | 0.010894 |
| CCNL1 | -1.04814 | 5.47E-06 | 6.85E-05 |
| PITPNM2 | -1.04737 | 6.02E-11 | 7.15E-09 |
| ACCS | -1.04711 | 2.03E-05 | 0.000197 |
| RAPGEF4 | -1.04656 | 2.15E-06 | 3.23E-05 |
| PRSS33 | -1.04623 | 0.013787 | 0.040012 |
| RASA4P | -1.04571 | 1.14E-06 | 1.95E-05 |
| LOC284865 | -1.04476 | 0.015395 | 0.04382 |
| PHOSPHO1 | -1.04408 | 2.49E-06 | 3.64E-05 |
| SLC27A3 | -1.04404 | 2.01E-07 | 4.74E-06 |
| LOC727915 | -1.04392 | 0.002701 | 0.010688 |
| MYH7B | -1.04307 | 2.9E-06 | 4.1E-05 |
| GSTM2 | -1.04157 | 7.61E-07 | 1.41E-05 |
| MAL | -1.04137 | 4.09E-06 | 5.45E-05 |
| OOEP | -1.04078 | 0.008131 | 0.026214 |
| SNORA77 | -1.0407 | 0.00061 | 0.003205 |
| GNRH1 | -1.04062 | 3.29E-06 | 4.56E-05 |
| NPNT | -1.04038 | 6E-08 | 1.8E-06 |
| CELA2B | -1.03898 | 0.000252 | 0.001555 |
| ODF3L2 | -1.03864 | 0.0068 | 0.022672 |
| TTN | -1.03861 | 9.99E-06 | 0.00011 |
| TSPAN7 | -1.0386 | 8.12E-09 | 3.49E-07 |
| PLEKHA1 | -1.03844 | 1.01E-10 | 1.04E-08 |
| DUXA | -1.03694 | 0.003842 | 0.014296 |
| TEX12 | -1.0367 | 0.000533 | 0.002878 |
| RSAD2 | -1.03639 | 2.7E-05 | 0.000248 |
| CMTM5 | -1.03605 | 0.00417 | 0.015262 |
| ASGR1 | -1.03585 | 0.000244 | 0.001516 |
| ZNF493 | -1.03564 | 2.84E-07 | 6.36E-06 |
| LEFTY1 | -1.03548 | 0.001578 | 0.006901 |
| ASB9P1 | -1.0354 | 2E-06 | 3.04E-05 |
| CORO6 | -1.0354 | 1.59E-05 | 0.000162 |
| AKR1E2 | -1.03516 | 1.77E-07 | 4.32E-06 |
| MGC27382 | -1.03452 | 0.004776 | 0.017063 |
| BGLAP | -1.03421 | 0.00826 | 0.026564 |
| C5orf20 | -1.03386 | 0.001317 | 0.005962 |
| LY6G6D | -1.03336 | 0.005218 | 0.018324 |
| CCDC42 | -1.03314 | 0.000802 | 0.00399 |
| SPRY4 | -1.03295 | 0.0004 | 0.002269 |
| MIR26B | -1.0327 | 0.001338 | 0.006039 |
| GZMA | -1.0327 | 0.000128 | 0.000888 |
| TGM1 | -1.03257 | 0.000254 | 0.001561 |
| ITGA10 | -1.03246 | 6.36E-06 | 7.7E-05 |
| C5orf38 | -1.03229 | 5.29E-06 | 6.68E-05 |
| AMBP | -1.03223 | 0.000566 | 0.003009 |
| TMEM191C | -1.0321 | 0.003032 | 0.01174 |
| CTAGE4 | -1.03072 | 0.012315 | 0.036592 |
| MTERFD3 | -1.0303 | 1.23E-05 | 0.00013 |
| RPL13AP17 | -1.02965 | 0.008044 | 0.026009 |
| MIR548A2 | -1.02956 | 0.005467 | 0.019044 |
| YPEL1 | -1.02929 | 2.38E-11 | 3.4E-09 |
| ZNF503-AS1 | -1.0288 | 8.89E-05 | 0.000657 |
| GPR133 | -1.02711 | 5.22E-07 | 1.05E-05 |
| AMT | -1.02695 | 1.49E-05 | 0.000154 |
| MS4A7 | -1.02694 | 2.86E-06 | 4.06E-05 |
| MIR3065 | -1.02608 | 0.005484 | 0.019076 |
| CEACAM21 | -1.02588 | 0.002347 | 0.009562 |
| KIAA1274 | -1.02513 | 2.85E-07 | 6.37E-06 |
| LOC388942 | -1.02492 | 0.005174 | 0.018204 |
| FABP5 | -1.0249 | 1.84E-05 | 0.000182 |
| MIR24-2 | -1.02424 | 0.018105 | 0.049852 |
| GP6 | -1.02418 | 0.001548 | 0.0068 |
| C19orf18 | -1.02402 | 0.006334 | 0.021423 |
| ALOX5 | -1.02395 | 7.92E-06 | 9.17E-05 |
| KLHDC1 | -1.02335 | 4.14E-07 | 8.66E-06 |
| C14orf166B | -1.02325 | 0.000127 | 0.000883 |
| TIE1 | -1.02275 | 5.39E-09 | 2.52E-07 |
| KLRAP1 | -1.02275 | 6.64E-07 | 1.26E-05 |
| CITED2 | -1.02265 | 4.11E-08 | 1.34E-06 |
| LOC283888 | -1.02204 | 0.002607 | 0.010393 |
| SLC22A3 | -1.02122 | 3.95E-07 | 8.32E-06 |
| LINC00304 | -1.02093 | 0.001966 | 0.008255 |
| MUC15 | -1.02087 | 0.001125 | 0.005253 |
| LOC100507410 | -1.02077 | 0.001732 | 0.007447 |
| NPHP3 | -1.02058 | 2.58E-06 | 3.73E-05 |
| GTF2IRD2B | -1.01979 | 3.02E-07 | 6.62E-06 |
| TRPM5 | -1.01958 | 0.01524 | 0.043429 |
| SLC7A14 | -1.01878 | 0.002525 | 0.010135 |
| ECHDC2 | -1.01844 | 6.72E-07 | 1.27E-05 |
| STARD8 | -1.01792 | 9.67E-07 | 1.71E-05 |
| LOC729080 | -1.01791 | 0.00256 | 0.010259 |
| CTNNBIP1 | -1.01779 | 8.92E-10 | 5.96E-08 |
| LOC728218 | -1.0176 | 0.008649 | 0.027605 |
| ARHGAP6 | -1.01752 | 1.84E-09 | 1.05E-07 |
| FTCD | -1.01748 | 0.017683 | 0.048947 |
| PHACTR1 | -1.01659 | 5.36E-05 | 0.000433 |
| INMT-FAM188B | -1.01608 | 0.00135 | 0.006079 |
| FLJ44511 | -1.01602 | 0.000117 | 0.000822 |
| SLC6A16 | -1.0151 | 3.24E-06 | 4.5E-05 |
| CSAD | -1.01403 | 3.81E-06 | 5.16E-05 |
| L3MBTL4 | -1.01402 | 1.45E-12 | 4.63E-10 |
| MIR223 | -1.01367 | 0.001462 | 0.006482 |
| STK31 | -1.0136 | 2.16E-05 | 0.000207 |
| LOC148696 | -1.01342 | 6E-06 | 7.36E-05 |
| EDA | -1.01326 | 5.95E-06 | 7.31E-05 |
| TPRG1 | -1.01306 | 3.91E-09 | 1.92E-07 |
| SULT1C4 | -1.01271 | 5.33E-05 | 0.000432 |
| HGFAC | -1.0126 | 0.004686 | 0.016787 |
| SEC1 | -1.01247 | 2.65E-05 | 0.000245 |
| LOC100506195 | -1.01246 | 0.001966 | 0.008255 |
| PRKCQ | -1.012 | 4.2E-07 | 8.78E-06 |
| SNORD97 | -1.01186 | 0.000205 | 0.001306 |
| DGCR9 | -1.01182 | 0.010466 | 0.032126 |
| ATG16L2 | -1.01029 | 2.55E-08 | 9.03E-07 |
| PDGFB | -1.00989 | 0.000496 | 0.002711 |
| KRT27 | -1.00947 | 0.002725 | 0.010755 |
| FAM132A | -1.00928 | 0.001254 | 0.00573 |
| FZD4 | -1.00924 | 7.86E-06 | 9.12E-05 |
| GPD1L | -1.00911 | 6.85E-12 | 1.47E-09 |
| NRN1 | -1.00895 | 4.39E-08 | 1.42E-06 |
| LOC100505696 | -1.00814 | 4.88E-09 | 2.3E-07 |
| PURG | -1.00741 | 0.000995 | 0.00475 |
| CFLAR-AS1 | -1.00722 | 1.62E-07 | 4.05E-06 |
| TDH | -1.00701 | 0.000378 | 0.002166 |
| NR2E3 | -1.00688 | 0.000613 | 0.003218 |
| PABPN1L | -1.00659 | 0.001582 | 0.006917 |
| TBX2 | -1.00618 | 1.05E-07 | 2.78E-06 |
| FAM211B | -1.00544 | 5.5E-07 | 1.08E-05 |
| ULBP1 | -1.00498 | 0.003919 | 0.014521 |
| REPS2 | -1.00388 | 8.04E-08 | 2.25E-06 |
| C2orf54 | -1.00339 | 1.29E-05 | 0.000136 |
| TRIB1 | -1.0032 | 0.000405 | 0.002291 |
| PKNOX2 | -1.00306 | 3.81E-05 | 0.00033 |
| RAPGEF3 | -1.00251 | 6.68E-07 | 1.27E-05 |
| FGD5 | -1.00242 | 9.44E-10 | 6.23E-08 |
| SGK1 | -1.00228 | 1.09E-06 | 1.88E-05 |
| ODF3 | -1.00227 | 0.000421 | 0.002359 |
| DHRS12 | -1.00218 | 2.99E-09 | 1.55E-07 |
| C7orf53 | -1.0018 | 0.000147 | 0.000991 |
